# Copper-Catalyzed Enantioselective Borylative Allyl–Allyl Coupling of Allenes and Allylic *gem*-Dichlorides

**DOI:** 10.1021/acscatal.3c00536

**Published:** 2023-04-10

**Authors:** Martín Piñeiro-Suárez, Andrés M. Álvarez-Constantino, Martín Fañanás-Mastral

**Affiliations:** Centro Singular de Investigación en Química Biolóxica e Materiais Moleculares (CiQUS), Universidade de Santiago de Compostela, 15782 Santiago de Compostela, Spain

**Keywords:** copper, allylboration, asymmetric catalysis, allenes, multifunctional compounds, noncovalent interactions

## Abstract

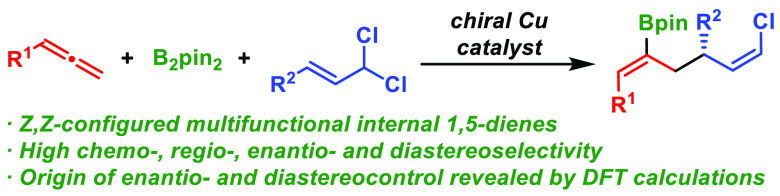

A catalytic asymmetric reaction between allenes, bis(pinacolato)diboron, and allylic *gem*-dichlorides is reported. The method involves the coupling of a catalytically generated allyl copper species with the allylic *gem*-dichloride and provides chiral internal 1,5-dienes featuring (*Z*)-configured alkenyl boronate and alkenyl chloride units with high levels of chemo-, regio-, enantio-, and diastereoselectivity. The synthetic utility of the products is demonstrated with the synthesis of a range of optically active compounds. DFT calculations reveal key noncovalent substrate–ligand interactions that account for the enantioselectivity outcome and the diastereoselective formation of the (*Z*)-alkenyl chloride.

Catalytic asymmetric transformations that provide control over several stereochemistry elements in a single operation are among the most important challenges in modern organic chemistry.^1^ Asymmetric allyl–allyl coupling of unsymmetrical allyl compounds represents an especially challenging type of reaction since, besides control over the enantioselectivity, many different regio- and stereoisomers can originate from coupling at either the α or γ position of both allylic reagents (Scheme 1a). Enantioselective allyl–allyl coupling has been achieved through Pd-,^2^ Cu-,^3^ and Ir-catalyzed^4^ coupling of stoichiometric allyl metal reagents with several allylic electrophiles. However, these methods are typically confined to the introduction of simple allyl fragments from allyl metal reagents, and are limited to the synthesis of terminal 1,5-dienes (Scheme 1b).^5,6^

**Scheme 1:**
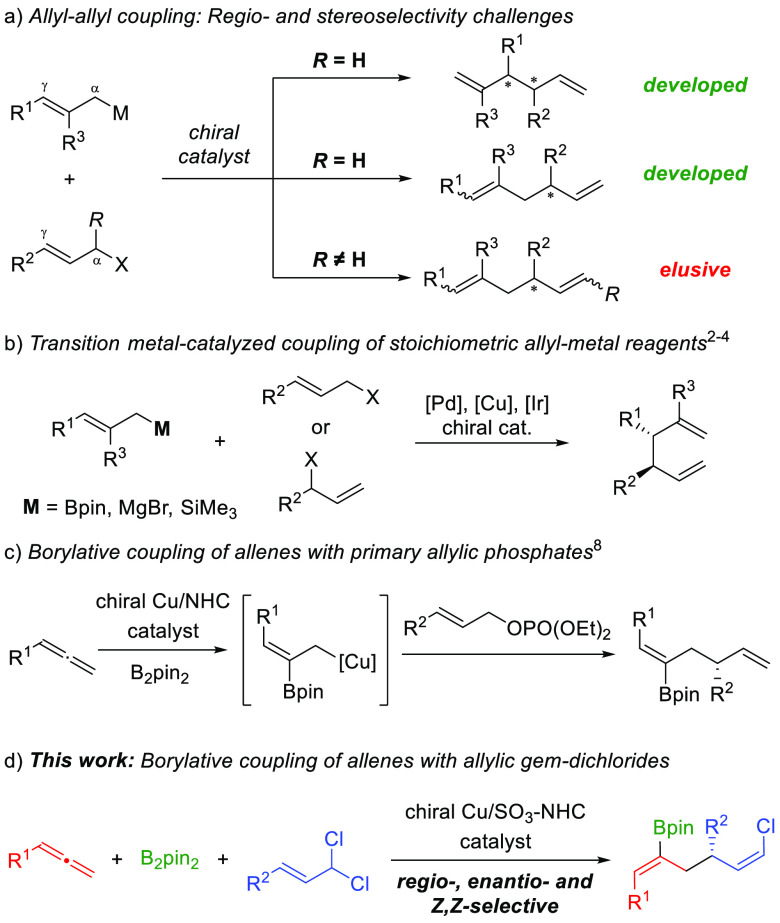
Enantioselective Allyl–Allyl Cross-Coupling

An interesting alternative to the stoichiometric use of allyl metal reagents in cross-coupling reactions is the catalytic generation of an allyl copper nucleophile by allene element-cupration.^7^ By using this strategy, Hoveyda et al. reported a regio- and enantioselective borylative allyl–allyl coupling between allenes and primary allylic phosphates (Scheme 1c).^8^ Additionally, Xiong et al.^9^ and Hoveyda et al.^10^ described the reductive version of this coupling by using copper hydride catalysis. Despite these efforts, the use of secondary 1,3-disubstituted allyl electrophiles in enantioselective allyl–allyl cross-coupling, which imposes an extra selectivity challenge since the geometry of the resulting internal double bond must be controlled, is elusive.^5^ As far as we are aware, only one racemic example of a borylative allyl–allyl coupling involving a 1,3-disubstituted allyl phosphate has been reported.^11^

Within our ongoing interest in developing enantioselective multicomponent couplings of allylic *gem*-dichlorides,^12,13^ we envisioned that an enantioselective allene allylboration based on this type of allylic substrates would afford an unprecedented chiral 1,5-diene structure bearing two orthogonal stereodefined functionalities (Scheme 1d). This high degree of functionalization would provide a useful tool to significantly expand the range of accessible molecules bearing the important and synthetically versatile 1,5-diene core.^14^ Moreover, it would represent a new synthetic application among the few catalytic methods available for the stereoselective synthesis of acyclic alkenyl chlorides.^15^

The envisioned transformation is highly demanding since it requires a chemo-, regio-, and stereoselective Cu-Bpin addition to the allene, followed by a regio- and enantioselective coupling of the resulting allylcopper species with the allylic *gem*-dichloride in a process where the geometry of the alkenyl chloride must be also controlled. Here, we report the successful realization of this idea through an enantioselective copper-catalyzed borylative allyl–allyl cross-coupling between allenes and allylic *gem*-dichlorides that efficiently provides chiral, densely functionalized internal 1,5-dienes bearing both (*Z*)-configured alkenyl chloride and alkenyl boronate units with high levels of chemo-, regio-, and stereoselectivity.

We began our study by surveying the reaction between cyclohexylallene **1**, (*E*)-(3,3-dichloroprop-1-en-1-yl)benzene **2** and B_2_pin_2_ (Table 1).

**Table 1:**
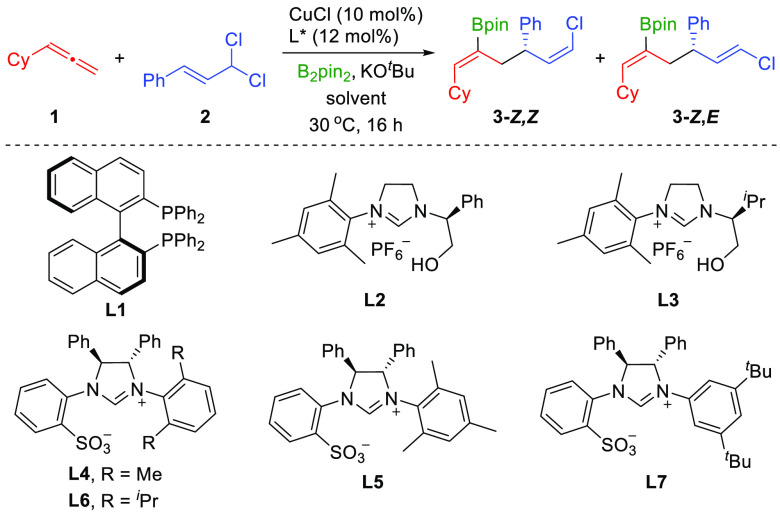
Optimization Studies

entry^a^	L*	solvent	conversion (%)^b^	*Z*,*Z*/*Z*,*E* ratio^c^	**3** yield (%)^d^	**3**-**(*****Z*****,*****Z*****/*****Z*****,*****E*****)** er^e^
1	**L1**	THF	72	2.2:1	26	51:49/52:48
2	**L2**	THF	85	1.3:1	21	39:61/45:55
3	**L3**	THF	75	1.5:1	15	56:44/46:54
4	**L4**	THF	80	>20:1	34	93:7/-
5	**L5**	THF	90	>20:1	32	91:9/-
6	**L6**	THF	88	>20:1	24	52:48/-
7	**L7**	THF	70	-	-	-
8	**L4**	dioxane	full	>20:1	48	96:4/-
9^f^,^g^	**L4**	dioxane	full	>20:1	62	96:4/-
10^f^,^g^	**L5**	dioxane	full	>20:1	68	96:4/-
11^f^,^g^	**L5**	toluene	full	>20:1	71	96:4/-
12^f^,^g^,^h^	**L5**	toluene	full	20:1	36	90:10/72:28
13^f^,^g^,^i^	**L5**	toluene	full	5:1	32	77:23/56:44

a Reaction conditions: **1** (0.3 mmol), **2** (0.2 mmol), B_2_pin_2_ (0.32 mmol), CuCl (10 mol %), ligand (12 mol %), KO^*t*^Bu (0.3 mmol), and solvent (2.0 mL) at 30 °C.

b Conversion (**2** consumption) was determined by ^1^H NMR analysis using an internal standard.

c Determined by ^1^H NMR analysis of reaction crude.

d Yield of isolated product.

e Enantioselectivity determined by SFC analysis.

f Using 0.4 mmol of KO^*t*^Bu.

g Using 0.4 mmol of **1**.

h NaO^*t*^Bu used instead of KO^*t*^Bu.

i LiO^*t*^Bu used instead of KO^*t*^Bu.

Initial chiral ligand screening revealed that the use of catalysts bearing a chiral bisphosphine (entry 1) or amino-alcohol-derived *N*-heterocyclic carbene (NHC) ligands (entries 2–3) led to nearly equimolar mixtures of stereoisomers **3-*Z,Z*** and **3**-***Z,E*** in low yield with almost negligible enantioselectivity. A significant improvement in stereoselectivity was observed when sulfonate-bearing NHC ligands^16^ were employed. Chiral copper complexes derived from **L4** and **L5**, which feature a 2,6-dimethylphenyl and a mesityl group, respectively, proved to be the most efficient catalysts and provided 1,5-diene **3** as a single (*Z*,*Z*)-isomer with very good enantiomeric ratio (entries 4 and 5). Changes on the *N*-aryl group had a negative impact since bulkier substituents at the ortho position led to diminished enantioselectivity (entry 6), while a 3,5-disubstitution pattern produced a complete loss of efficiency (entry 7). Further evaluation of different solvents and reaction stoichiometry (entries 8–11) provided the optimal set of conditions, which comprises the use of **L5** as ligand, KO^*t*^Bu as base, and toluene as solvent. Under these conditions, product **3** was obtained as a single isomer in 71% yield with 96:4 er (entry 11). Notably, no formation of other isomers arising either from coupling at the γ position of the allylcopper intermediate or from S_N_2-type substitution of the allylic *gem*-dichloride could be detected. Interestingly, in sharp contrast with our previously reported borylative coupling between alkynes and allylic *gem*-dichlorides,^12^ the use of alkoxide bases bearing smaller metal cations led to a significant decrease of both efficiency and stereoselectivity (entries 12–13).

Once the optimized conditions were identified, the scope of the reaction was explored (Scheme 2). The method proved to be remarkably effective for a wide range of allenes and provided solely the corresponding (*Z*,*Z*)-configured 1,5-dienes in good yield with excellent levels of chemo-, regio-, and enantioselectivity. Allenes bearing simple alkyl groups (**3**,**4**), or aliphatic chains bearing functionalities, such as aryl rings (**5**,**6**), silyl ether (**7**), ester (**8**), ether (**9**), or carbamate (**10**), were well tolerated. Heterocyclic-substituted allenes were also amenable for this transformation, as illustrated with the synthesis of piperidine and tetrahydropyran derivatives **11** and **12**. 1-Phenylallene still provided the corresponding product **13** as a single (*Z*,*Z*)-isomer with perfect regioselectivity, albeit with slightly diminished enantiomeric ratio.^17^ Importantly, scalability of the reaction was demonstrated through the 1 mmol-scale synthesis of **3** using lower catalyst loading (5 mol %).

**Scheme 2:**
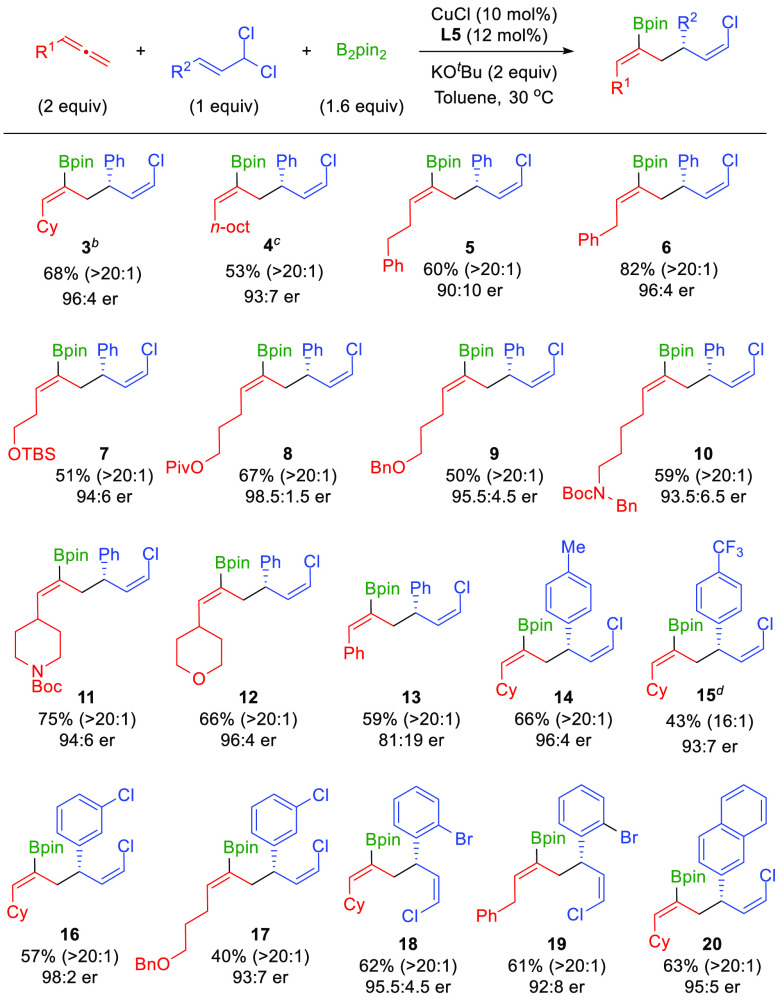
Scope of the Enantioselective Borylative Coupling of Allenes and Allylic *gem*-Dichlorides Unless otherwise noted, all reactions were performed on a 0.2 mmol scale under optimized conditions (Table 1, entry 11). Yield values refer to isolated products. *Z*,*Z*/*Z*,*E* selectivity is reported in brackets. Reaction run on a 1 mmol scale using 5 mol % of the catalyst. Reaction run at 40 °C over 48 h. Reaction run at 60 °C.

Different allylic *gem*-dichlorides were also evaluated for this enantioselective borylative allyl–allyl cross-coupling. Substrates with aryl rings bearing alkyl (**14**) or trifluoromethyl (**15**) groups proved to be efficient for this transformation. Halogenated aromatic substrates also furnished the corresponding 1,5-dienes (**16**–**19**) with good yield and excellent selectivities. Remarkably, the use of a hindered *ortho*-bromo-substituted substrate was well tolerated and provided products **18** and **19** without selectivity erosion. A substrate featuring another sterically demanding unit, such as a naphthalene group, could also be used with very high selectivity (**20**). Aliphatic *gem*-dichlorides were not suitable for the described enantioselective borylative allyl–allyl coupling.^18^ Absolute stereochemistry of the products was determined by X-ray diffraction analysis of product **11**, which revealed the configuration of the carbon stereogenic center to be (*S*), and confirmed the (*Z*)-configuration of both double bonds.

The combination of the high level of functionality of the products and their stereochemistry makes them versatile building blocks for the synthesis of a range of chiral structures (Scheme 3). Stereodefined 1,5-dienyl chlorides **21** and **22** could be accessed by chemoselective intermolecular Suzuki–Miyaura cross-coupling. Because of the remaining alkenyl chloride, the 1,5-diene structure can be further functionalized as shown with the formation of **23**. The presence of an *ortho*-bromo-substituted aryl ring in products **18** and **19** provides an extra handle to perform an intramolecular coupling, as illustrated with the synthesis of indane derivative **24**. Notably, the alkenyl boronate and the alkenyl chloride functionalities could be efficiently combined through intramolecular Pd-catalyzed cross-coupling to provide chiral cyclopentene **25**. Oxidation of the alkenyl boronate unit allows for the preparation of chiral alkenyl chloride-containing ketones (i.e., **26**–**30**). Finally, chiral borylated 1,5-enyne **31** could be obtained with full stereochemical retention by LDA-mediated dehydrochlorination.

**Scheme 3:**
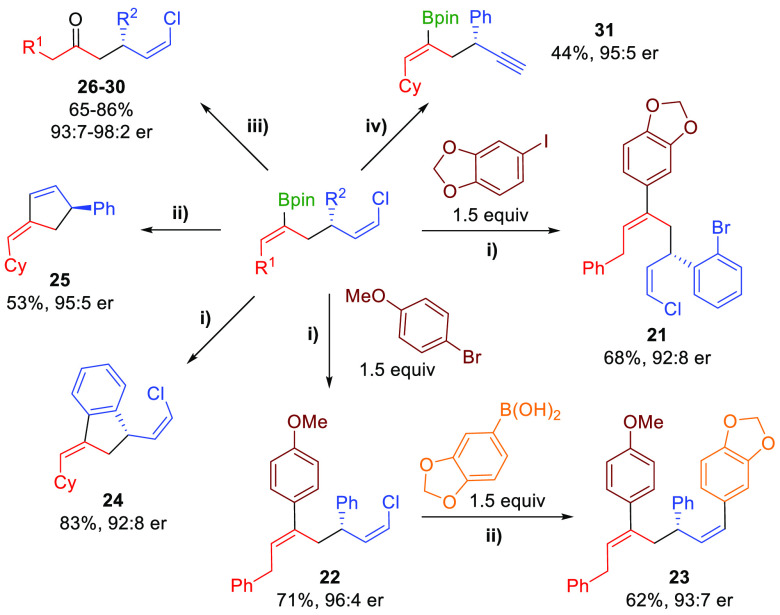
Synthetic Modifications of Products Conditions: (i) Pd(PPh_3_)_4_ (10 mol %), NaOH 2M, dioxane, 100 °C; (ii) Pd_2_(dba)_3_ (5 mol %), XPhos (10 mol %), CsF (3 equiv), dioxane, 100 °C; (iii) NaBO_3_·4H_2_O (5 equiv), THF:H_2_O, rt; (iv) LDA (2.5 equiv), THF, −78 °C, 5 min.

To analyze the factors that control the stereoselectivity of this enantioselective borylative allyl–allyl coupling, DFT calculations were performed (see the Supporting Information for details). Two lowest-energy structures (**Ia** and **Ib**), which rapidly interconvert (Δ*G*_sol_ = 0.5 kcal·mol^–1^), were found for the σ-allyl-Cu(I) intermediate. Among the different investigated coordination modes for the stereodetermining oxidative addition step in the formation of **3-*****Z*****,*****Z***,^19^ the one featuring the allyl *gem*-dichloride opposite to the *N*-arylsulfonate group (i.e., coordination to **Ia**), led to the most favorable pathway to the major (*S*)-enantiomer, while the preferred pathway to the minor (*R*)-enantiomer arises from coordination to **Ib** where the allyl substrate is opposite to the *N*-mesityl (NMes) unit (Figure 1a). The relative energy for transition state **TS**_**OA**_**-*****Z***,***S*** was 2.7 kcal·mol^–1^ lower than for **TS**_**OA**_**-*****Z***,***R***, which is in good agreement with the observed 96:4 er. In order to account for this energy difference, analysis of non-covalent interactions (NCI) was performed. NCI plots of both transition state structures showed no significant differences in terms of repulsive interactions (see the Supporting Information). However, analysis of the attractive interactions revealed that the coordination mode in **TS**_**OA**_**-*****Z***,***S*** allows for the establishment of a double potassium cation bridge interaction between the NHC’s sulfonate group and both chloride units. In contrast, only one K···Cl interaction is present in **TS**_**OA**_**-*****Z***,***R*** (Figure 1b). This difference in attractive interactions may suggest that the origin of enantioselectivity arises from the extra stabilization in **TS**_**OA**_**-*****Z***,***S***, which originates from the double substrate–ligand cation bridge interaction. In this line, analysis of the optimized structures for the transition states using a lithium cation instead of potassium showed that the extra stabilization given by the second cation bridge interaction of the second chlorine atom is missing in **TS**_**OA**_**-*****Z***,***S*****-Li** (Figure 1c). This leads to a higher-energy transition state and, thus, to a smaller energy difference with **TS**_**OA**_**-*****Z***,***R*****-Li**.^20^ The lack of the second stabilizing interaction may be due to the smaller size of the Li cation and might explain the lower enantioselectivity observed when LiO^*t*^Bu is used instead of KO^*t*^Bu (see Table 1, entries 11 vs 13).

**Figure 1:**
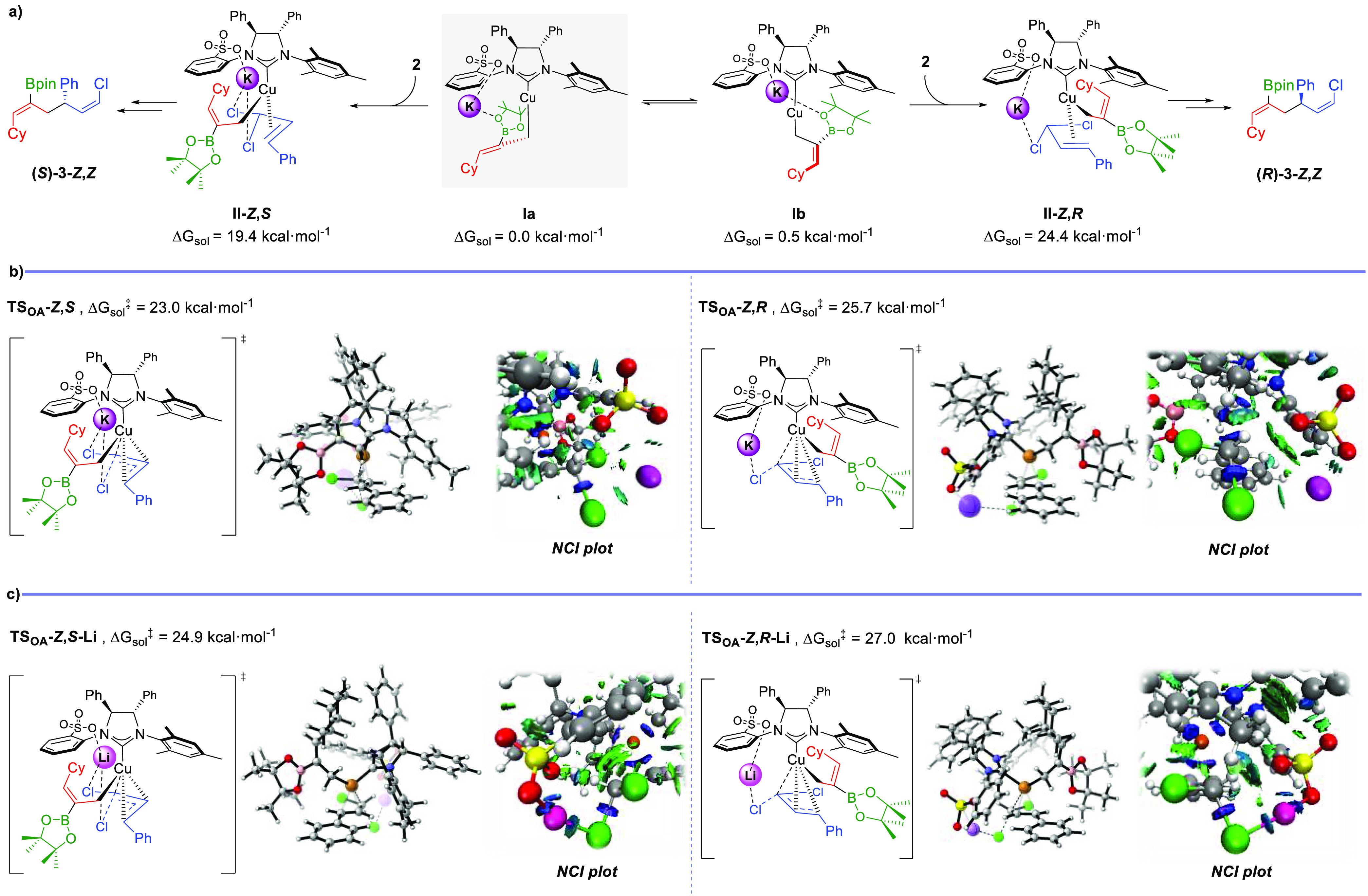
Optimized structures and energies obtained from DFT calculations performed at the ωB97XD/*def2*-TZVP/*def2*-QZVP (Cu) (scrf = smd, toluene)//ωB97XD/6-31G(d,p)/SDD+f (Cu) level for (a) σ-allyl-Cu(I) intermediates **Ia** and **Ib** and their most favorable π-olefin Cu(I) complexes with allylic *gem*-dichloride **2** and for (b) the stereochemistry-determining oxidative-addition transition states associated with the most favored pathways leading to **(*****S*****)-3,*****Z***,***Z*** and **(*****R*****)-3,*****Z***,***Z*** using KO^*t*^Bu and (c) using LiO^*t*^Bu.

To evaluate the factors that govern the diastereoselective formation of the (*Z*)-alkenyl chloride, we calculated the optimized structure for transition state **TS**_**OA**_**-*****E***,***S*** that leads to the formation of diastereomer **3-*****Z***,***E***.^21^ In accordance with the experimental results, **TS**_**OA**_**-*****E***,***S*** resulted in higher energy than the preferred **TS**_**OA**_**-*****Z***,***S*** (Δ*G*^‡^ = 30.7 kcal·mol^–1^ vs Δ*G*^‡^ = 23.0 kcal·mol^–1^). NCI plot analysis revealed that a new repulsive interaction between the Bpin unit and the *N*-arylsulfonate group appears in **TS**_**OA**_**-*****E***,***S*** when compared with **TS**_**OA**_**-*****Z***,***S*** (Figure 2). Moreover, repulsive interactions between the cyclohexyl ring and the phenyl and NMes units of the NHC ligand become larger in **TS**_**OA**_**-*****E***,***S***. Analysis of **TS**_**OA**_**-*****E***,***S*** geometry shows that in order to establish the stabilizing double-cation bridge interaction the system has to adopt a structural reorganization, which involves a decrease on the dihedral angle displayed between NHC carbenic carbon and C6 of the arylsulfonate ring (60.5° in **TS**_**OA**_**-*****E***,***S*** vs 68.2° in **TS**_**OA**_**-*****Z***,***S***). This angle modification pushes the *N*-arylsulfonate ring closer to the Bpin unit, thereby engendering the new repulsive interaction. Consequently, the σ-allyl substituent adopts a new spatial disposition that enhances the repulsive interactions between the cyclohexyl group and the NHC’s phenyl and mesityl rings. This set of repulsive interactions would result in a higher-energy transition state and would explain the origin of the (*Z*)-selectivity.

**Figure 2:**
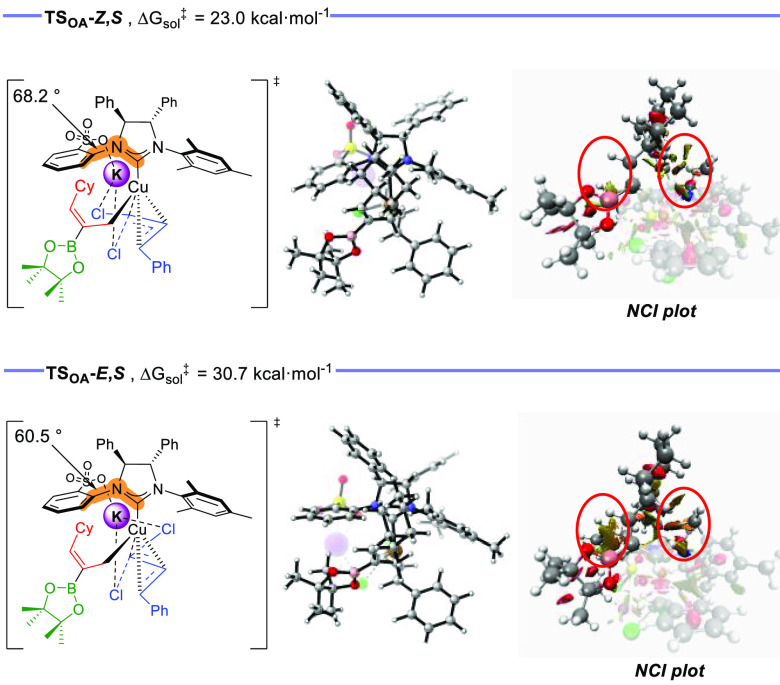
Optimized structures and energies obtained from DFT calculations performed at the ωB97XD/*def2*-TZVP/*def2*-QZVP (Cu) (scrf = smd, toluene)//ωB97XD/6-31G(d,p)/SDD+f (Cu) level for stereochemistry-determining oxidative-addition transition states associated with the most favored pathways leading to **(*****S*****)-3,*****Z***,***Z*** and **(*****S*****)-3,*****Z***,***E***.

In summary, we have disclosed an enantioselective borylative allyl–allyl cross-coupling between allenes and allylic *gem*-dichlorides to provide optically active (*Z*,*Z*)-configured internal 1,5-dienes. Remarkable features of the method are the observed excellent chemo-, regio-, and enantioselectivity, the stereoselective formation of two orthogonally functionalized alkenyl units, and the synthetic versatility of the resulting chiral borylated chlorodienes. Intrinsic stereochemical features were analyzed by DFT calculations, which revealed key noncovalent interactions that allow rationalization of the observed high levels of enantio- and diastereoselectivity.

## Supporting Information Available

List of starting materials and ligands, experimental procedures, compound characterization data, NMR spectra, X-ray crystallographic data for **11**, details for DFT calculations, NCI plots, Cartesian coordinates, imaginary frequencies, and absolute energies in hartrees for all optimized geometries (PDF)
CCDC 2222779 (CIF)

## Supplementary Material

cs3c00536_si_001.pdf

cs3c00536_si_002.cif

## Accession Codes

CCDC 2222779 contains the supplementary crystallographic data for this paper. These data can be obtained free of charge via www.ccdc.cam.ac.uk/data_request/cif, or by emailing data_request@ccdc.cam.ac.uk, or by contacting The Cambridge Crystallographic Data Centre, 12 Union Road, Cambridge CB2 1EZ, UK; fax: + 44 1223 336033.
