# Supplementary material for: Copper-Catalyzed Enantioselective Borylative Allyl–Allyl Coupling of Allenes and Allylic gem-Dichlorides
Source: ACS Catal. 2023 Apr 10;13(8):5578–83. doi: 10.1021/acscatal.3c00536 (PMC10127276; doi:10.1021/acscatal.3c00536)
Supplement: Supplementary file 1 — cs3c00536_si_001.pdf [file cs3c00536_si_001.pdf]

Supporting Information

Copper-Catalyzed Enantioselective Borylative Allyl-Allyl Coupling of Allenes and Allylic *gem*-Dichlorides

Martín Piñeiro-Suárez, Andrés M. Álvarez-Constantino and Martín Fañanás-Mastral*

Centro Singular de Investigación en Química Biolóxica e Materiais Moleculares (CiQUS), Universidade de Santiago de Compostela, 15782 Santiago de Compostela, Spain

Correspondence to: martin.fananas@usc.es

Table of contents

1. General methods 3
2. List of starting materials and chiral ligands 4
3. Synthesis of chiral ligand **L6** 5
4. Synthesis of allenes **38** and **40** 7
5. General procedure for the Cu-catalyzed enantioselective borylative coupling of allenes with allylic *gem*-dichlorides 8
6. Product characterization 9
7. Product derivatizations 19
    7.1. Suzuki-Miyaura cross-couplings 19
        7.1.1. Intermolecular Suzuki-Miyaura cross-couplings 19
        7.1.2. Intramolecular Suzuki-Miyaura cross-couplings 22
    7.2. Oxidation of alkenyl boronates 24
    7.3. Transformation of alkenyl chloride **3** into terminal alkyne **31** 27
8. Chemoselectivity versus other unsaturated hydrocarbons 28
9. Unsuccessful substrates 29
10. ^{1}H-NMR and ^{13}C-NMR spectra 30
11. X-ray diffraction analysis data of product **11** 62
12. DFT Calculations 64
    12.1. Basis set and functional benchmarking to the experimental value 64
    12.2. Energy profiles and optimized structures 67
        12.2.1. Energy profile for the pathways associated to the formation of products (***S***)-**3**-***Z*,*Z*** and (***R***)-**3**-***Z*,*Z*** using K as metal cation 67
        12.2.2. Energy profile for the pathways associated to the formation of products (***S***)-**3**-***Z*,*E*** and (***R***)-**3**-***Z*,*E*** using K as metal cation 70
        12.2.3. Energy profile for the pathways associated to the formation of products (***S***)-**3**-***Z*,*Z*** and (***R***)-**3**-***Z*,*Z*** using Li as metal cation 71
    12.3. Non-Covalent Interactions (NCI) plots 73
        12.3.1. NCI plots for **TS$_{OA}$-*Z*,*S*** and **TS$_{OA}$-*Z*,*R*** 73
        12.3.2. NCI plots for **TS$_{OA}$-*Z*,*S*-Li** and **TS$_{OA}$-*Z*,*R*-Li** 75
        12.3.3. NCI plots for **TS$_{OA}$-*Z*,*S*** and **TS$_{OA}$-*Z*,S-Li** 76
        12.3.4. NCI plots for **TS$_{OA}$-*Z*,*S*** and **TS$_{OA}$-*E*,S** 78
    12.4. Cartesian coordinates 80
13. References 187

1. General methods

• All reactions were performed under argon atmosphere using oven dried glassware and using standard Schlenk techniques. Solvents were dried using an MBraun SPS 800 system. All chemicals were purchased from Acros Organics Ltd., Aldrich Chemical Co. Ltd., Alfa Aesar, Apollo, Strem Chemicals Inc., Fluorochem Ltd. or TCI Europe N.V. chemical companies and used without further purification, unless otherwise noted.

• Analytical thin layer chromatography was carried out on silica-coated aluminum plates (silica gel 60 F254 Merck) and components were visualized by UV light and $KMnO_4$ staining. Flash column chromatography was performed on silica gel 60 (Merck, 230-400 mesh) without previous deactivation, unless otherwise stated.

• High Resolution Mass spectrometry was carried out on a Bruker microTOF spectrometer using ESI or APCI.

• ^{1}H, ^{13}C, ^{11}B and ^{19}F NMR experiments were carried out using a Bruker AVIII 500MHz or a Varian Mercury 300MHz NMR spectrometer. Chemical shift values are reported in ppm with the solvent resonance as the internal standard ($CHCl_3$: δ 7.26 for ^{1}H, δ 77.16 for ^{13}C). Coupling constants (J) are given in Hertz (Hz). Multiplicities are reported as follows: s = singlet, d = doublet, t = triplet, q = quartet, m = multiplet or as a combination of them.

• Because of quadrupolar relaxation, in all cases the carbon directly attached to the boron atom was not detected by ^{13}C NMR technique.

• Melting points were determined using a Buchi-M565 apparatus.

• Optical rotation was determined in a Jasco P-2000 Polarimeter.

• Enantiomeric ratios were determined by Supercritical Fluid Chromatography (SFC) analysis in a Jasco Series 4000 instrument or by High Performance Liquid Chromatography (HPLC) analysis using a WATERS ACQUITY Arc System, consisting of a quaternary pump, column oven and autosampler coupled with a 2998 PDA detector.

• In order to preclude side protoboration reactions, commercial B_2pin_2 was dried over Na_2SO_4 prior to being used.

2. List of starting materials and chiral ligands

Allene **1** and chiral ligand **L1** were purchased from commercial sources. Allenes **32-34**[1], **35**[2], **36**[3], **37**[4], **39**[5], **41**[6], allylic *gem*-dichlorides **2**[7], **42**[8], **43-46**[9] and chiral ligands **L2**[10], **L3**[11], **L4**[12], **L5**[13], **L7**[14] were prepared according to literature procedures.

- Allenes:

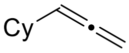

1

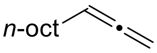

32

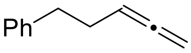

33

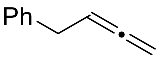

34

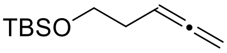

35

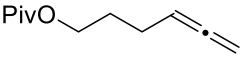

36

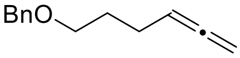

37

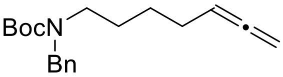

38

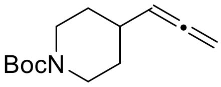

39

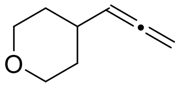

40

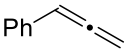

41

- Allylic *gem*-dichlorides:

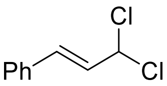

2

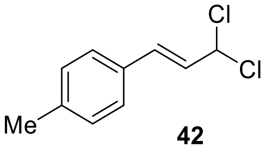

42

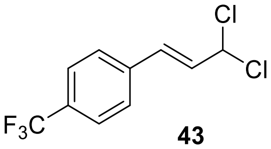

43

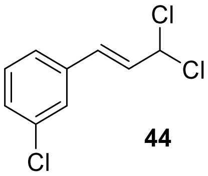

44

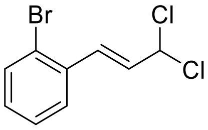

45

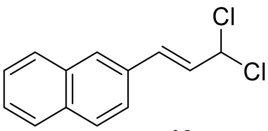

46

- Chiral ligands:

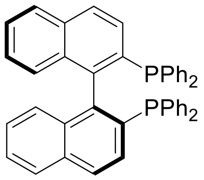

L1

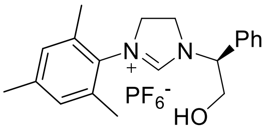

L2

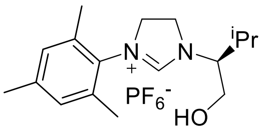

L3

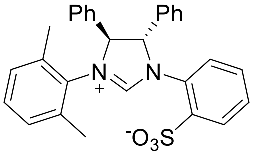

L4

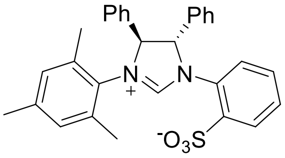

L5

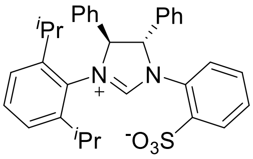

L6

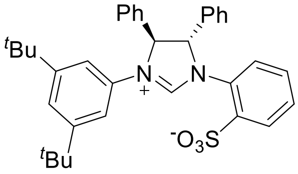

L7

3. Synthesis of chiral ligand **L6**

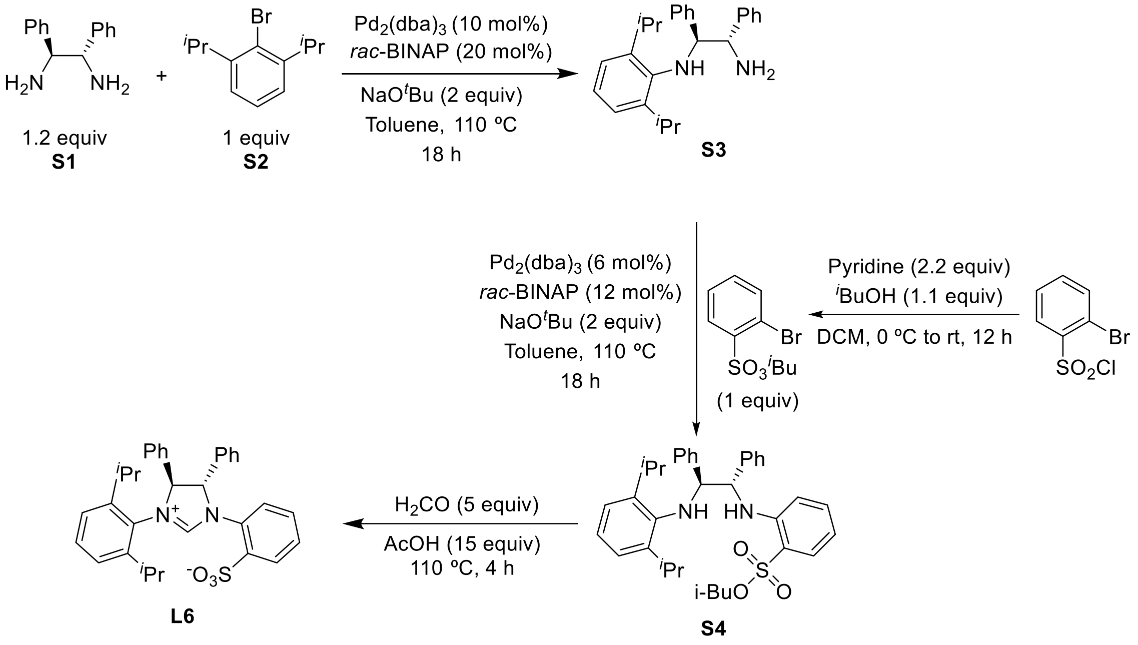

- **First step:** A flame-dried round-bottom flask equipped with reflux condenser was charged with (-)-(*S*,*S*) -1,2-Diphenylethylenediamine (1.2 equiv, 7.2 mmol), $Pd_2(dba)_3$ (10 mol%, 0.6 mmol), *rac*-BINAP (20 mol%, 1.2 mmol) and NaO^tBu (2 equiv, 12 mmol) under a dry argon atmosphere. A solution of the bromobenzene **S2** (1 equiv, 6.0 mmol) in toluene [0.1 M] was added through a syringe. The resulting mixture was stirred at 110 ^{o}C during 18 h. After that time, the mixture was allowed to cool to room temperature, loaded directly on top of a column containing silica gel and purified by silica gel column chromatography (Hexane:AcOEt, 95:5 to 80:20) to afford the corresponding diamine **S3** as a red oil in 70% yield.

- **Second step:** $Pd_2(dba)_3$ (6 mol%, 0.42 mmol), *rac*-BINAP (12 mol%, 0.84 mmol), and NaO^tBu (2 equiv, 8.4 mmol) were weighed into an oven-dried 100 mL round bottom flask inside a glove box. The flask was removed from the glove box and fitted with a reflux condenser. A solution of the corresponding diamine **S3** (1 equiv, 4.2 mmol) in toluene [0.1 M] and a solution of the isobutyl-2-bromobenzenesulfonate (1 equiv, 4.2 mmol) in toluene [0.1 M] were sequentially added to the mixture which was stirred at 110 ^oC during 18 h. After that time, the mixture was cooled down to room temperature, loaded directly on top of a column containing silica gel and purified by silica gel column chromatography (Hexane:AcOEt, 90:10) to afford the corresponding diamine **S4** as a red oil in 74% yield.

- **Third step:** The corresponding diamine **S4** (1 equiv, 3.1 mmol) was weighed into a 25 mL round bottom flask. The flask was fitted with a reflux condenser. Acetic acid (15 equiv, 46.5 mmol), followed by formaldehyde (5 equiv, 15.5 mmol) were added through a syringe and stirred at 110 ^oC during 4 h. Then, the mixture was cooled to room temperature and diluted with CH_2Cl_2 (10 mL). The reaction was neutralized by the slow addition of a saturated $NaHCO_3$ aq. solution until gas evolution ceased. The mixture was washed with saturated aqueous solution of NH_4Cl (2x10 mL). Then, the aqueous layer was extracted with CH_2Cl_2 (10 mL). Combined organic layers were dried over anhydrous Na_2SO_4, filtered and solvent was removed under reduced pressure. Crude product was purified by silica gel column chromatography (AcOEt:MeOH, 100:0 to 90:10) to afford the corresponding imidazolium salt **L6** as a white solid in 28% yield.

2-((4*S*,5*S*)-3-(2,6-Diisopropylphenyl)-4,5-diphenyl-4,5-dihydro-1H-imidazol-3-ium-1-yl)benzenesulfonate (L6)

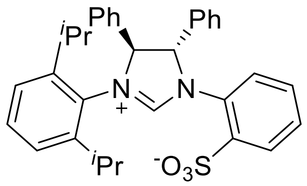

^{1}H-NMR (300 MHz, $CDCl_3$) δ 8.80 (s, 1H), 8.24 (d, *J* = 7.8 Hz, 1H), 7.65 (m, 2H), 7.51 – 7.23 (m, 11H), 7.16 – 7.01 (m, 2H), 6.78 (d, *J* = 8.0 Hz, 1H), 6.61 (d, *J* = 11.9 Hz, 1H), 5.39 (d, *J* = 11.9 Hz, 1H), 3.27 (m, 2H), 1.55 (d, *J* = 6.8 Hz, 3H), 1.44 (d, *J* = 6.7 Hz, 3H), 1.10 (d, *J* = 6.5 Hz, 3H), 0.31 (d, *J* = 6.7 Hz, 3H). **^{13}C-NMR** (75 MHz, $CDCl_3$) δ 159.0, 149.5, 145.9, 143.8, 134.8, 131.3, 131.1, 130.7, 135.0, 130.4, 130.3, 130.0, 129.8, 129.8, 129.4, 128.9, 128.4, 127.5, 125.5, 124.6, 78.4, 74.4, 29.3, 28.6, 26.4, 25.4, 24.7, 22.7. **HRMS (APCI)** Calc. for $C_{33}H_{35}N_2O_3S$ [$M+H^+$] 539.2370, found 539.2363. **Optical rotation:** $[\alpha]_D^{22}$ + 26.4 (c=0.99, $CHCl_3$). **Mp (^oC):** 193-196.

4. Synthesis of allenes **38** and **40**

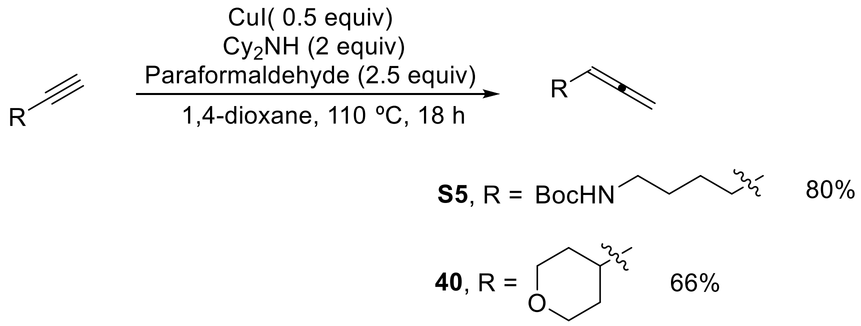

A flame-dried two-necked 100 ml round-bottom flask equipped with a magnetic stirring bar, was charged with paraformaldehyde (2.5 equiv) and backfilled with argon gas. Dry 1,4-dioxane (1 M concentration regarding the acetylene derivative) added as solvent and dicyclohexylamine (2 equiv) by syringe. The mixture was stirred for 10 minutes at room temperature and then copper(I) iodide (0.5 equiv) was added in one portion and the resulting mixture was stirred for 5 minutes, while the white slurry mixture turns green. Under argon atmosphere, the acetylene derivative was added by syringe in one portion, and the reaction mixture was boiled gently (at 110 °C) over night (at the beginning the reaction turns quickly to yellow and as it evolves to dark brown). Once the reaction reached completion, the mixture was cooled down to room temperature, and diethyl ether was added. The slurry mixture was filtered through a pad of celite and a pad of silica. The filtered yellow solution was evaporated to obtain a crude oil, which was purified by column chromatography using silica gel.

4-(Propa-1,2-dien-1-yl)tetrahydro-2*H*-pyran (40)

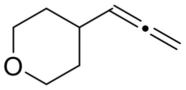

Synthesized from 4-ethynyltetrahydro-2*H*-pyran. Colorless oil obtained after column chromatography (Pentane:CH_2Cl_2, 80:20) in 66% yield. **^{1}H NMR** (500 MHz, $CDCl_3$) δ 5.11 (q, *J* = 6.4 Hz, 1H), 4.73 (dd, *J* = 6.7, 3.3 Hz, 2H), 3.94 (dt, *J* = 11.6, 3.3 Hz, 2H), 3.42 (td, *J* = 11.5, 2.3 Hz, 2H), 2.27 – 2.17 (m, 1H), 1.69 – 1.64 (m, 2H), 1.46 (dtd, *J* = 13.8, 11.3, 4.4 Hz, 2H). **^{13}C NMR** (126 MHz, $CDCl_3$) δ 207.7, 94.8, 76.3, 67.8, 33.9, 32.7. **HRMS (APCI)** Calc. For $C_8H_{13}O$ [M+H$^+$] 125.0961, found 125.0963.

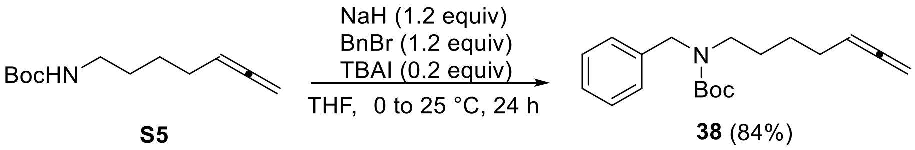

A flame-dried 100 ml round-bottom flask equipped with a magnetic stirring bar, was charged with sodium hydride (60 m/m% in mineral oil, 1.2 equiv, 7.2 mmol, 288 mg). Under argon atmosphere, the solid was washed with dry hexane (3x1 ml) and dried in vacuum. Then, THF (4 ml) was added and the resulting mixture was cooled to 0 °C using an ice/water bath. Then a solution of *tert*-Butyl hepta-5,6-dien-1-ylcarbamate **S5** (1.0 equiv, 6 mmol) in THF (6 mL) was added dropwise by syringe over 10 minutes (CAUTION: H_2 gas formation). The resulting brown mixture was stirred for 10 minutes at 0 °C, then the cooling bath was removed and to the mixture was warmed to room temperature over 30 minutes. Then, a suspension of tetrabutylammonium

iodide (0.2 equiv, 1.2 mmol, 443 mg) and benzyl bromide (98%, d=1.438, 1.2 equiv, 7.2 mmol, 1257 mg, 0.874 ml) in THF (2 mL) was added in one portion and the resulting mixture was stirred overnight at room temperature. The reaction mixture was filtered through a plug of celite, diluted with dry diethyl ether and quenched by the careful addition of water while being stirred for 15 minutes. The mixture was then extracted with diethyl ether (20 mL), and the organic phase was washed with water (3x10mL) and brine (3x10mL). and dried over $MgSO_4$. The filtered yellow solution was evaporated to obtain the crude product, which was purified by silica gel column chromatography using a 20:1 mixture of hexane: ethyl acetate as eluent. The final product was obtained in 84% yield as a colorless oil.

tert-Butyl benzyl(hepta-5,6-dien-1-yl)carbamate (38)

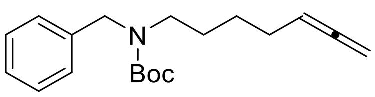

^{1}H NMR (300 MHz, DMSO) δ 7.36 – 7.20 (m, 5H), 5.13 (p, J = 6.7 Hz, 1H), 4.71 (dt, J = 6.6, 3.3 Hz, 2H), 4.36 (s, 2H), 3.12 (s, 2H), 1.98 – 1.88 (m, 2H), 1.52 – 1.25 (m, 13H). **^{13}C NMR** (75 MHz, DMSO) δ 207.8, 138.7, 128.3, 127.1, 126.9, 89.5, 78.6, 75.2, 49.1, 45.9, 28.0, 27.1, 25.6. **HRMS (APCI)** Calc. for $C_{19}H_{28}NO_2$ [M+H$^+$] 302.2115, found 302.2104.

5. General procedure for the Cu-catalyzed enantioselective borylative coupling of allenes with allylic *gem*-dichlorides

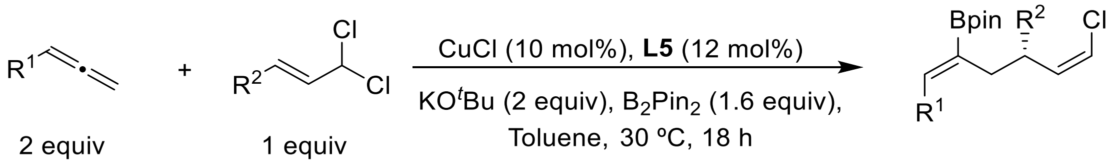

General procedure A:

A flame-dried Schlenk tube equipped with a magnetic stirring bar was charged with CuCl (10 mol%, 0.02 mmol) and KO^{t}Bu (2 equiv, 0.4 mmol) in a glovebox. The Schlenk tube was removed from the glovebox, ligand **L5** (12 mol%, 0.024 mmol) was added, and the mixture was dissolved in dry toluene (0.5 mL) and stirred during 30 min at room temperature. B_2Pin_2 (1.6 equiv, 0.32 mmol) was added to the Schlenk tube and the mixture was stirred during 15 min. The corresponding allene (2.0 equiv, 0.4 mmol) and allylic *gem*-dichloride (1.0 equiv, 0.2 mmol) were dissolved in dry toluene (each substrate in 0.5 mL) and added to the Schlenk tube. The resulting mixture was stirred over 18 h at 30 °C. Then, the mixture was diluted with CH_2Cl_2 (5 ml) and washed with saturated aqueous solution of NH_4Cl (2x5 mL). The aqueous layer was extracted with CH_2Cl_2 (5 mL). Combined organic layers were dried over anhydrous Na_2SO_4, filtered and solvent was removed under reduced pressure. Crude product was purified through flash column chromatography using the indicated mixture of solvents as eluent.

Note: Racemic products were synthesized running the reaction at 30 °C in toluene using SIMes as ligand and KO^{t}Bu as base. In cases where chiral separation of racemic *Z* and *E* isomers was not possible, racemic **L5** ligand was used instead of SIMes.

6. Product characterization

2-((*S*,1*Z*,5*Z*)-6-Chloro-1-cyclohexyl-4-phenylhexa-1,5-dien-2-yl)-4,4,5,5-tetramethyl-1,3,2-dioxaborolane (3)

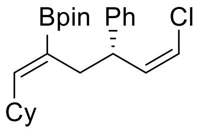

Synthesized from **1**, **2** and B_2Pin_2 according to general procedure A. Yellow oil obtained in 71% yield with 96:4 er after column chromatography (hexane:CH_2Cl_2, 95:5 to 85:15). **^{1}H NMR** (300 MHz, $CDCl_3$) δ 7.30 – 7.14 (m, 5H), 6.12 (d, *J* = 9.9 Hz, 1H), 6.04 – 5.93 (m, 2H), 4.09 – 3.98 (m, 1H), 2.61 – 2.50 (m, 2H), 2.32 (m, 1H), 1.78 – 1.55 (m, 5H), 1.44 – 0.93 (m, 17H). **^{13}C NMR** (75 MHz, $CDCl_3$) δ 153.3, 143.7, 135.3, 128.5, 127.8, 126.4, 117.7, 83.2, 44.6, 38.1, 35.1, 32.7, 32.6, 26.1, 26.0, 26.0, 25.0, 24.8. **^{11}B NMR** (160 MHz, $CDCl_3$) δ 32.0. **HRMS (APCI)** Calc. for $C_{24}H_{35}BClO_2$ [M+H$^+$] 401.2418, found 401.2413. **Optical rotation:** $[\alpha]_D^{22}$ + 46.3 (c=1.01, $CHCl_3$).

Enantiomeric purity was determined by chiral SFC analysis [Lux Cellulose-1, 100 bar, T_{oven}: 40 °C, Flow: 2.2 mL/min; 1% MeOH, λ =220 nm, major enantiomer t_R = 19.02 min, minor enantiomer t_R = 20.40 min].

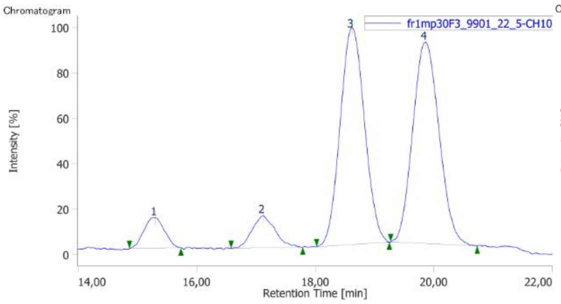

Peak Information

#	Peak Name	CH	tR [min]	Area [μV·sec]	Area%
1	Unknown	10	15.307	94781	5.280
2	Unknown	10	17.123	110068	6.132
3	Unknown	10	18.620	793521	44.205
4	Unknown	10	19.857	796728	44.384

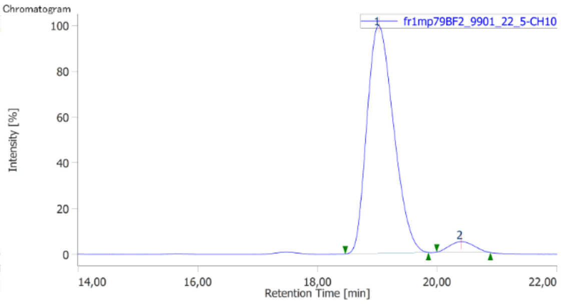

Peak Information

#	Peak Name	CH	tR [min]	Area [μV·sec]	Area%
1	Unknown	10	19.020	19388049	95.883
2	Unknown	10	20.400	832500	4.117

Note: Chromatogram of racemic compound shows peaks for *Z,E*-isomer (15.31 and 17.12 min) and *Z,Z*-isomer (18.62 and 19.86 min).

2-((*S*,1*Z*,5*Z*)-1-Chloro-3-phenyltetradeca-1,5-dien-5-yl)-4,4,5,5-tetramethyl-1,3,2-dioxaborolane (4)

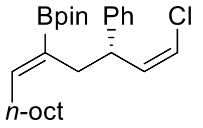

Synthesized from **32**, **2** and B_2Pin_2 according to general procedure A at 40 °C (48 h). Yellow oil obtained in 53% yield with 93:7 er after column chromatography (hexane:CH_2Cl_2, 90:10 to 80:20). **^{1}H NMR** (300 MHz, $CDCl_3$) δ 7.28 – 7.26 (m, 4H), 7.23 – 7.13 (m, 1H), 6.34 (t, *J* = 7.3 Hz, 1H), 6.04 – 5.92 (m, 2H), 4.05 (q, *J* = 8.3 Hz, 1H), 2.55 (d, *J* = 7.7 Hz, 2H), 2.09 (t, *J* = 7.3 Hz, 2H), 1.22 (d, *J* = 2.8 Hz, 24H), 0.88 (t, *J* = 6.2 Hz, 3H). **^{13}C NMR** (75 MHz, $CDCl_3$) δ 148.2, 143.7, 135.1, 128.4, 127.6, 126.2, 117.7, 83.1, 43.9, 34.8, 31.9, 29.6, 29.5, 29.2, 29.1, 29.0, 24.8, 24.7, 22.7, 14.0. **^{11}B NMR** (160 MHz, $CDCl_3$) δ 31.1. **HRMS (APCI)** Calc. for $C_{26}H_{41}BClO_2$ [M+H$^+$] 431.2883, found 431.2883. **Optical rotation:** $[\alpha]_D^{21}$ + 53.7 (c=0.78, $CHCl_3$).

Enantiomeric purity was determined by chiral SFC analysis [Lux Cellulose-1, 100 bar, T_{oven}: 40 °C, Flow: 2.2 mL/min; 1% MeOH, λ =220 nm, major enantiomer t_R = 19.03 min, minor enantiomer t_R = 21.07 min].

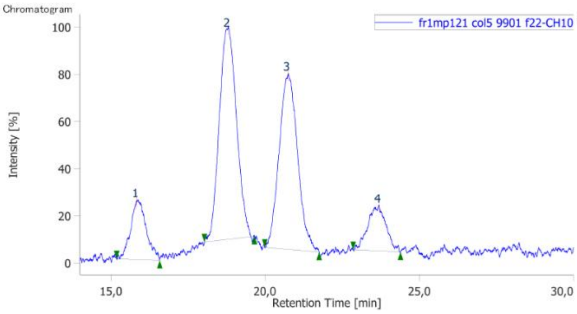

Peak Information

#	Peak Name	CH	tR [min]	Area [μV·sec]	Area%
1	Unknown	10	15.847	103152	10.705
2	Unknown	10	18.810	407247	42.264
3	Unknown	10	20.757	361344	37.500
4	Unknown	10	23.687	91837	9.531

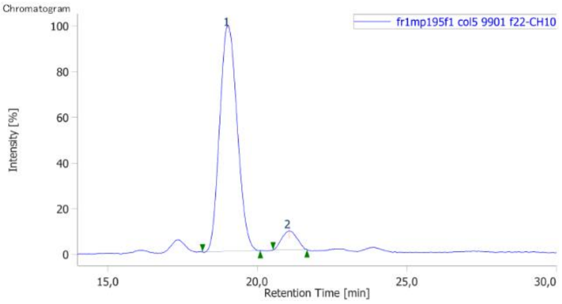

Peak Information

#	Peak Name	CH	tR [min]	Area [μV·sec]	Area%
1	Unknown	10	19.033	6706604	93.098
2	Unknown	10	21.070	497216	6.902

Note: Chromatogram of racemic compound shows peaks for *Z*,*E*-isomer (15.85 and 23.69 min) and *Z*,*Z*-isomer (18.81 and 20.76 min).

2-((*S*,3*Z*,7*Z*)-8-Chloro-1,6-diphenylocta-3,7-dien-4-yl)-4,4,5,5-tetramethyl-1,3,2-dioxaborolane (5)

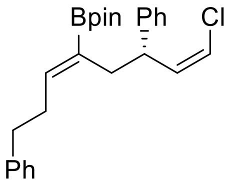

Synthesized from **33**, **2** and B_2Pin_2 according to general procedure A. Yellow oil obtained in 60% yield with 90:10 er after column chromatography (hexane:CH_2Cl_2, 90:10 to 80:20). This compound was isolated together with 9% of the dechlorinated product. **^{1}H NMR** (500 MHz, $CDCl_3$) δ 7.23 – 7.15 (m, 5H), 7.13 – 7.08 (m, 5H), 6.34 (t, *J* = 7.1 Hz, 1H), 5.93 (d, *J* = 7.1 Hz, 1H), 5.85 (dd, *J* = 9.7, 7.1 Hz, 1H), 4.00 (dt, *J* = 9.6, 7.7 Hz, 1H), 2.62 – 2.52 (m, 2H), 2.48 (d, *J* = 7.7 Hz, 2H), 2.34 (q, *J* = 7.8 Hz, 2H), 1.17 (s, 6H), 1.16 (s, 6H). **^{13}C NMR** (126 MHz, $CDCl_3$) δ 146.8, 143.7, 142.2, 135.0, 128.5, 128.5, 128.5, 127.7, 126.4, 126.0, 117.9, 83.3, 43.9, 35.5, 35.0, 31.2, 25.0, 24.8. **^{11}B NMR** (160 MHz, $CDCl_3$) δ 30.7. **HRMS (APCI)** Calc. for $C_{26}H_{33}BClO_2$ [M+H$^+$] 423.2257, found 423.2262. **Optical rotation:** $[\alpha]_D^{21}$ + 44.1 (c=0.71, $CHCl_3$).

Enantiomeric purity was determined by chiral SFC analysis [Lux Cellulose-1, 100 bar, T_{oven}: 40 °C, Flow: 1 mL/min; 3% MeOH, λ =220 nm, major enantiomer t_R = 43.03 min, minor enantiomer t_R = 47.84 min].

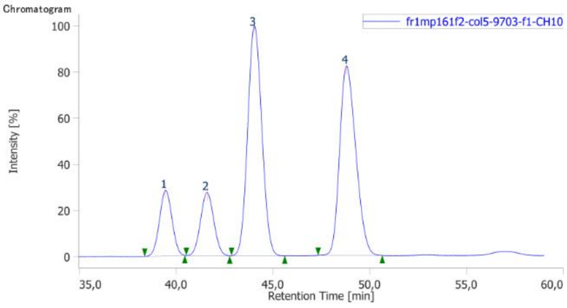

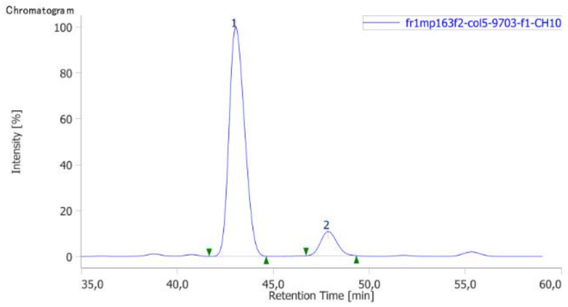

Peak Information

#	Peak Name	CH	tR [min]	Area [μV·sec]	Area%
1	Unknown	10	39.453	2549968	10.253
2	Unknown	10	41.610	2616921	10.523
3	Unknown	10	44.047	10128576	40.727
4	Unknown	10	48.810	9573840	38.497

Peak Information

#	Peak Name	CH	tR [min]	Area [μV·sec]	Area%
1	Unknown	10	43.033	41879122	90.013
2	Unknown	10	47.840	4646676	9.987

Note: Chromatogram of racemic compound shows peaks for *Z,E*-isomer (39.45 and 41.61 min) and *Z,Z*-isomer (44.05 and 48.81 min).

2-((*S*,2*Z*,6*Z*)-7-Chloro-1,5-diphenylhepta-2,6-dien-3-yl)-4,4,5,5-tetramethyl-1,3,2-dioxaborolane (6)

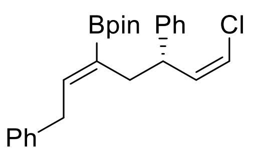

Synthesized from **34**, **2** and B_2Pin_2 according to general procedure A. Yellow oil obtained in 82% yield with 96:4 er after column chromatography (hexane:CH_2Cl_2, 90:10 to 80:20). **^{1}H NMR** (500 MHz, $CDCl_3$) δ 7.30 – 7.20 (m, 6H), 7.20 – 7.14 (m, 2H), 7.11 (d, *J* = 7.6 Hz, 2H), 6.47 (t, *J* = 7.2 Hz, 1H), 6.04 – 5.95 (m, 2H), 4.13 (q, *J* = 8.2 Hz, 1H), 3.42 (dd, *J* = 7.2, 2.9 Hz, 2H), 2.67 (d, *J* = 7.8 Hz, 2H), 1.20 (s, 6H), 1.19 (s, 6H). **^{13}C NMR** (126 MHz, $CDCl_3$) δ 145.7, 143.6, 140.3, 135.0, 128.8, 128.6, 128.5, 127.7, 126.5, 126.1, 118.0, 83.4, 44.0, 35.2, 35.1, 25.0, 24.8. **^{11}B NMR** (160 MHz, $CDCl_3$) δ 30.3. **HRMS (APCI)** Calc. For $C_{25}H_{31}BClO_2$ [M+H$^+$] 409.2100, found 409.2103. **Optical rotation:** $[\alpha]_D^{22}$ + 49.1 (c=0.51, $CHCl_3$).

Enantiomeric purity was determined on Suzuki cross-coupling product **22** (see section 7.1.1).

***tert*-Butyl(((*S*,3*Z*,7*Z*)-8-chloro-6-phenyl-4-(4,4,5,5-tetramethyl-1,3,2-dioxaborolan-2-yl)octa-3,7-dien-1-yl)oxy)dimethylsilane (7)**

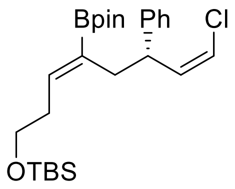

Synthesized from **35**, **2** and B_2Pin_2 according to general procedure A. Yellow oil obtained in 51% yield with 94:6 er after column chromatography (hexane:CH_2Cl_2, 80:20 to 60:40). **^{1}H NMR** (300 MHz, $CDCl_3$) δ 7.30 – 7.23 (m, 4H), 7.18 (td, *J* = 6.1, 3.6 Hz, 1H), 6.31 (t, *J* = 7.2 Hz, 1H), 6.02 (d, *J* = 7.1 Hz, 1H), 5.95 (dd, *J* = 9.4, 7.1 Hz, 1H), 4.08 (dt, *J* = 9.3, 7.7 Hz, 1H), 3.61 (t, *J* = 7.3 Hz, 2H), 2.58 (d, *J* = 7.7 Hz, 2H), 2.36 (q, *J* = 7.2 Hz, 2H), 1.23 (s, 6H), 1.22 (s, 6H), 0.89 (s, 9H), 0.05 (s, 6H). **^{13}C NMR** (75 MHz, $CDCl_3$) δ 143.7, 143.5, 135.1, 128.6, 127.7, 126.4, 117.9, 83.3, 62.7, 43.9, 35.1, 32.9, 29.9, 26.1, 25.0, 24.8, -5.1. **^{11}B NMR** (160 MHz, $CDCl_3$) δ 30.1. **HRMS (APCI)** Calc. For $C_{26}H_{43}BClO_3Si$ [M+H$^+$] 477.2758, found 477.2765. **Optical rotation:** $[\alpha]_D^{22}$ + 33.0 (c=0.37, $CHCl_3$).

Enantiomeric purity was determined by chiral SFC analysis [Lux Cellulose-1, 100 bar, T_{oven}: 40 °C, Flow: 1.0 mL/min; 1% MeOH, λ =220 nm, major enantiomer t_R = 26.01 min, minor enantiomer t_R = 27.52 min].

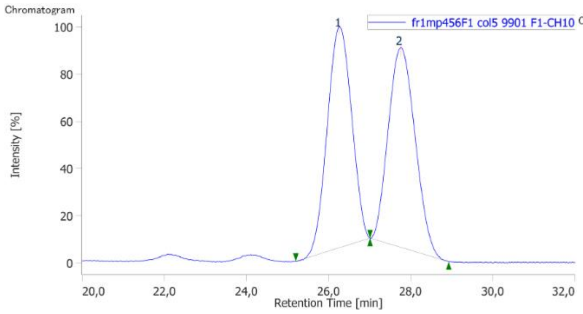

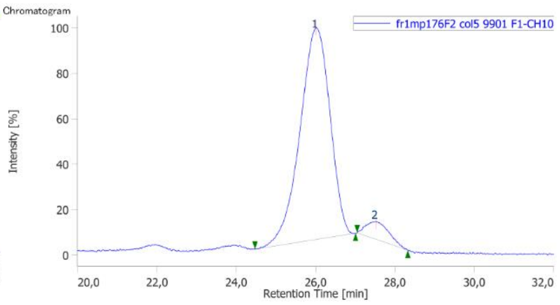

Peak Information

#	Peak Name	CH	tR [min]	Area [µV·sec]	Area%
1	Unknown	10	26,270	3171164	50,116
2	Unknown	10	27,760	3156436	49,884

Peak Information

#	Peak Name	CH	tR [min]	Area [µV·sec]	Area%
1	Unknown	10	26,013	2148097	93,912
2	Unknown	10	27,523	139247	6,088

(*S*,4*Z*,8*Z*)-9-Chloro-7-phenyl-5-(4,4,5,5-tetramethyl-1,3,2-dioxaborolan-2-yl)nona-4,8-dien-1-yl pivalate (8)

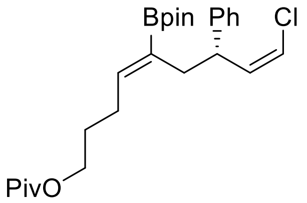

Synthesized from **36**, **2** and B_2Pin_2 according to general procedure A. Yellow oil obtained in 67% yield with 98.5:1.5 er after column chromatography (hexane:CH_2Cl_2, 80:20 to 50:50). **^{1}H NMR** (500 MHz, $CDCl_3$) δ 7.31 – 7.21 (m, 4H), 7.18 (dt, *J* = 6.1, 1.4 Hz, 1H), 6.32 (t, *J* = 7.2 Hz, 1H), 6.02 (dd, *J* = 7.2, 0.8 Hz, 1H), 5.95 (dd, *J* = 9.7, 7.1 Hz, 1H), 4.10 – 3.95 (m, 3H), 2.58 – 2.53 (m, 2H), 2.21 – 2.15 (m, 2H), 1.71 – 1.62 (m, 2H), 1.23 (s, 6H), 1.22 (s, 6H), 1.18 (s, 9H). **^{13}C NMR** (126 MHz, $CDCl_3$) δ 178.7, 146.4, 143.6, 135.0, 128.5, 127.7, 126.4, 117.9, 83.3, 64.1, 43.9, 38.8, 35.0, 28.2, 27.3, 25.5, 25.0, 24.8. **^{11}B NMR** (160 MHz, $CDCl_3$) δ 29.9. **HRMS (APCI)** Calc. For $C_{26}H_{39}BClO_4$ [M+H$^+$] 461.2624, found 461.2631. **Optical rotation:** $[\alpha]_D^{22}$ + 57.3 (c=0.69, $CHCl_3$).

Enantiomeric purity was determined by chiral uHPLC analysis [Lux i-Cellulose-5, T_{oven}: 40 °C, Flow: 0.3 mL/min; 99.5% hexane, λ =209.5 nm, major enantiomer t_R = 19.40 min, minor enantiomer t_R = 21.64 min].

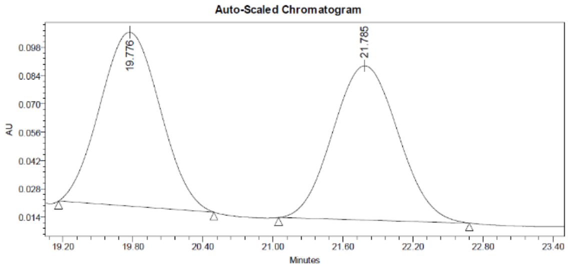

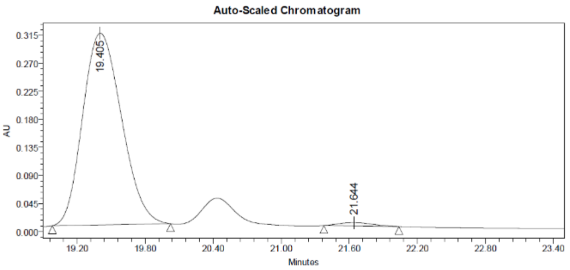

Unknown Peak Results

	RT	Area	% Area	Height
1	19.776	3080670	50.87	86030
2	21.785	2975842	49.13	76351

Unknown Peak Results

	RT	Area	% Area	Height
1	19.405	7327831	98.51	307804
2	21.644	111202	1.49	5280

2-((*S*,1*Z*,5*Z*)-9-(Benzyloxy)-1-chloro-3-phenylnona-1,5-dien-5-yl)-4,4,5,5-tetramethyl-1,3,2-dioxaborolane (9)

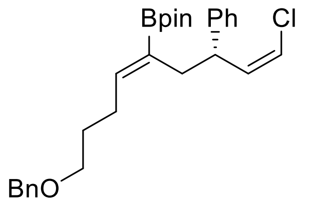

Synthesized from **37**, **2** and B_2Pin_2 according to general procedure A. Yellow oil obtained in 50% yield with 95.5:4.5 er after column chromatography (hexane:AcOEt, 99:1 to 94:6). This compound was isolated together with 7% of the dechlorinated product. **^{1}H NMR** (500 MHz, $CDCl_3$) δ 7.39-7.36 (m, 4H), 7.32-7.29 (m, 5H), 7.26 – 7.19 (m, 1H), 6.39 (t, *J* = 7.2 Hz, 1H), 6.05 (d, *J* = 7.0 Hz, 1H), 6.00 (dd, *J* = 9.2, 7.4 Hz, 1H), 4.53 (s, 2H), 4.12 (q, *J* = 8.2 Hz, 1H), 3.51 (t, *J* = 7.0 Hz, 2H), 2.62 (d, *J* = 7.8 Hz, 2H), 2.30 – 2.23 (m, 2H), 1.76 – 1.67 (m, 2H), 1.28 (s, 6H), 1.27 (s, 6H). **^{13}C NMR** (126 MHz, $CDCl_3$) δ 147.2, 143.7, 138.8, 135.1, 128.5, 128.4, 127.7, 127.7, 127.6, 126.4, 117.9, 83.2, 72.9, 70.0, 44.0, 35.0, 29.2, 25.6, 25.0, 24.8. **^{11}B NMR** (160 MHz, $CDCl_3$) δ 30.7. **HRMS (APCI)** Calc. For $C_{28}H_{37}BClO_3$ [M+H$^+$] 467.2519, found 467.2522. **Optical rotation:** $[\alpha]_D^{21}$ + 35.9 (c=0.70, $CHCl_3$).

Enantiomeric purity was determined on oxidation product **26** (see section 7.2).

***tert*-Butyl benzyl((*S*,5*Z*,9*Z*)-10-chloro-8-phenyl-6-(4,4,5,5-tetramethyl-1,3,2-dioxaborolan-2-yl)deca-5,9-dien-1-yl)carbamate (10)**

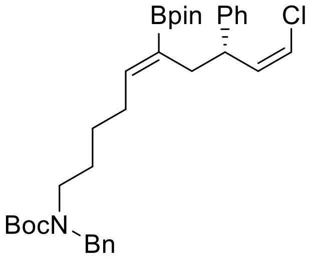

Synthesized from **38**, **2** and B_2Pin_2 according to general procedure A. Yellow oil obtained in 59% yield with 93.5:6.5 er after column chromatography (hexane:AcOEt, 98:2 to 94:6). This compound was isolated together with 6% of the dechlorinated product. **^{1}H NMR** (300 MHz, $CDCl_3$) δ 7.38 – 7.25 (m, 4H), 7.25 – 7.14 (m, 6H), 6.31 (t, *J* = 7.0 Hz, 1H), 6.02 (d, *J* = 7.1 Hz, 1H), 5.96 (dd, *J* = 9.5, 7.0 Hz, 1H), 4.35-4.47 (m, 4H), 4.06 (q, *J* = 8.1 Hz, 1H), 3.29 – 2.98 (m, 4H), 2.55 (d, *J* = 7.6 Hz, 2H), 2.11 (p, *J* = 7.2 Hz, 2H), 1.47 (s, 9H), 1.24 (s, 6H), 1.23 (s, 6H). **^{13}C NMR** (126 MHz, $CDCl_3$) δ 147.5, 143.6, 135.1, 128.5, 128.5, 128.5, 128.3, 128.0, 127.7, 127.2, 126.4, 117.8, 83.2, 79.6, 44.0, 34.9, 28.6, 26.4, 25.0, 24.9, 24.9, 24.8, 24.8, 24.7. **^{11}B NMR** (160 MHz, $CDCl_3$) δ 30.9. **HRMS (APCI)** Calc. For $C_{34}H_{48}BClNO_2$ [M+H$^+$] 580.3359, found 580.3368. **Optical rotation:** $[\alpha]_D^{22}$ + 20.9 (c=0.43, $CHCl_3$).

Enantiomeric purity was determined by chiral uHPLC analysis [Lux i-amylose-3, T_{oven}: 40 °C, Flow: 1 mL/min; 99% hexane, λ =209.5 nm, minor enantiomer t_R = 9.49 min, major enantiomer t_R = 10.06 min].

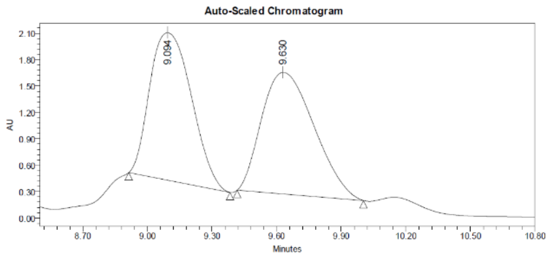

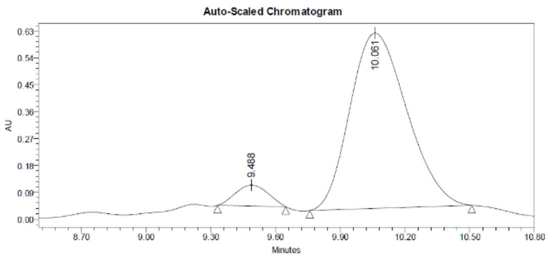

Unknown Peak Results

	RT	Area	% Area	Height
1	9.094	22656938	49.14	1678502
2	9.630	23450761	50.86	1382203

Unknown Peak Results

	RT	Area	% Area	Height
1	9.488	732187	6.38	69633
2	10.061	10747819	93.62	586601

tert-Butyl 4-((*S*,1*Z*,5*Z*)-6-chloro-4-phenyl-2-(4,4,5,5-tetramethyl-1,3,2-dioxaborolan-2-yl)hexa-1,5-dien-1-yl)piperidine-1-carboxylate (11)

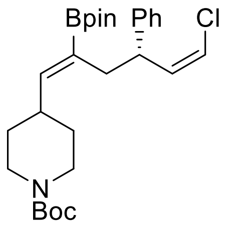

Synthesized from **39**, **2** and B_2Pin_2 according to general procedure A. White solid obtained in 75% yield with 94:6 er after column chromatography (hexane:AcOEt, 98:2 to 94:6). **^{1}H NMR** (500 MHz, $CDCl_3$) δ 7.23 – 7.15 (m, 4H), 7.13 – 7.08 (m, 1H), 5.99 (d, *J* = 9.8 Hz, 1H), 5.96 (d, *J* = 7.1 Hz, 1H), 5.92 (dd, *J* = 9.4, 7.0 Hz, 1H), 3.97 – 3.91 (m, 3H), 2.64 – 2.44 (m, 4H), 2.36 – 2.29 (m, 1H), 1.38 (s, 9H), 1.19 – 1.15 (m, 16H). **^{13}C NMR** (126 MHz, $CDCl_3$) δ 155.0, 150.6, 143.3, 135.0, 128.5, 127.8, 126.5, 117.8, 83.4, 79.4, 44.5, 36.1, 35.2, 31.3, 29.8, 28.6, 25.0, 24.8. **^{11}B NMR** (160 MHz, $CDCl_3$) δ 30.5. **HRMS (APCI)** Calc. for $C_{28}H_{42}BClNO_4$ [M+H$^+$] 502.2890, found 502.2893. **Optical rotation:** $[\alpha]^{22}_{D}$ + 20.2 (c=0.66, $CHCl_3$). **Mp (ºC):** 25-30.

Enantiomeric purity was determined by chiral SFC analysis [Lux Cellulose-5, 100 bar, T_{oven}: 40 °C, Flow: 0.6 mL/min; 5% MeOH, λ =220 nm, major enantiomer t_R = 46.93 min, minor enantiomer t_R = 49.82 min].

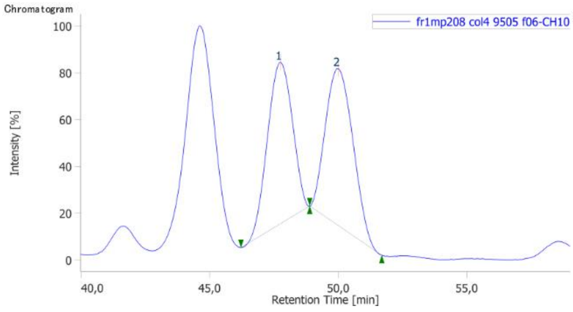

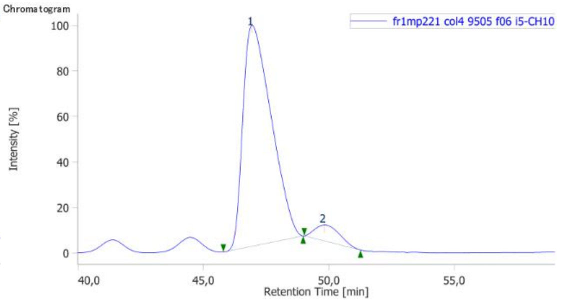

Peak Information

#	Peak Name	CH	tR [min]	Area [μV·sec]	Area%
1	Unknown	10	47.753	4503443	48.211
2	Unknown	10	49.997	4837630	51.789

Peak Information

#	Peak Name	CH	tR [min]	Area [μV·sec]	Area%
1	Unknown	10	46.933	33636508	93.951
2	Unknown	10	49.817	2165851	6.049

Note: Chromatogram of racemic compound shows peaks for *Z*,*E*-isomer (not separated) and *Z*,*Z*-isomer (47.75 and 50.00 min).

2-((*S*,1*Z*,5*Z*)-6-Chloro-4-phenyl-1-(tetrahydro-2H-pyran-4-yl)hexa-1,5-dien-2-yl)-4,4,5,5-tetramethyl-1,3,2-dioxaborolane (12)

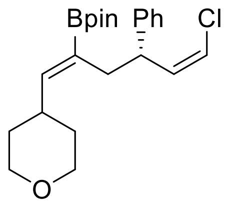

Synthesized from **40**, **2** and B_2Pin_2 according to general procedure A. Yellow oil obtained in 66% yield with 96:4 er after column chromatography (hexane:AcOEt, 99:1 to 94:6). **^{1}H NMR** (500 MHz, $CDCl_3$) δ 7.31 – 7.23 (m, 4H), 7.20 – 7.16 (m, 1H), 6.09 (d, *J* = 9.8 Hz, 1H), 6.03 (d, *J* = 7.2 Hz, 1H), 5.99 (dd, *J* = 9.5, 7.1 Hz, 1H), 4.02 (dt, *J* = 9.6, 7.6 Hz, 1H), 3.98 – 3.86 (m, 2H), 3.37 (dtd, *J* = 28.3, 11.6, 3.0 Hz, 2H), 2.65 – 2.48 (m, 3H), 1.54 – 1.34 (m, 4H), 1.23 (s, 6H), 1.23 (s, 6H). **^{13}C NMR** (126 MHz, $CDCl_3$) δ 150.8, 143.4, 135.1, 128.6, 127.8, 126.5, 117.9, 83.4, 67.7, 67.6, 44.6, 35.2, 35.1, 32.3, 32.1, 25.0, 24.8. **^{11}B NMR** (160 MHz, $CDCl_3$) δ 30.9. **HRMS (APCI)** Calc. for $C_{23}H_{33}BClO_3$ [M+H$^+$] 403.2206, found 403.2206. **Optical rotation:** $[\alpha]^{22}_{D}$ + 59.1 (c=0.54, $CHCl_3$).

Enantiomeric purity was determined by chiral SFC analysis [Lux Cellulose-1, 100 bar, T_{oven}: 40 °C, Flow: 0.8 mL/min; 2% MeOH, λ =220 nm, major enantiomer t_R = 60.49 min, minor enantiomer t_R = 62.71 min].

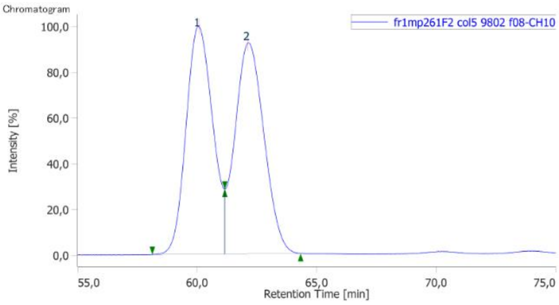

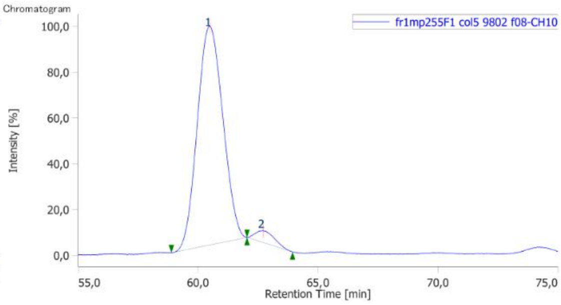

Peak Information

#	Peak Name	CH	tR [min]	Area [µV·sec]	Area%
1	Unknown	10	60.070	13203523	49.745
2	Unknown	10	62.140	13339033	50.255

Peak Information

#	Peak Name	CH	tR [min]	Area [µV·sec]	Area%
1	Unknown	10	60.490	6503180	96.054
2	Unknown	10	62.713	267156	3.946

2-((*S*,1*Z*,5*Z*)-6-Chloro-1,4-diphenylhexa-1,5-dien-2-yl)-4,4,5,5-tetramethyl-1,3,2-dioxaborolane (13)

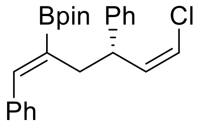

Synthesized from **41**, **2** and B_2Pin_2 according to general procedure A. Yellow oil obtained in 59% yield with 81:19 er after column chromatography (hexane:CH_2Cl_2, 90:10 to 80:20). **^{1}H NMR** (300 MHz, $CDCl_3$) δ 7.39 – 7.23 (m, 8H), 7.22 – 7.14 (m, 3H), 6.02 (d, *J* = 7.0 Hz, 1H), 5.85 (dd, *J* = 9.4, 7.3 Hz, 1H), 4.25 (q, *J* = 8.4 Hz, 1H), 2.91 – 2.82 (m, 2H), 1.31 (d, *J* = 8.0 Hz, 12H). **^{13}C NMR** (75 MHz, $CDCl_3$) δ 144.2, 143.6, 138.1, 135.1, 129.1, 128.5, 128.3, 127.7, 127.2, 126.4, 118.0, 83.7, 43.5, 35.3, 25.1, 24.9. **^{11}B NMR** (160 MHz, $CDCl_3$) δ 30.9. **HRMS (APCI)** Calc. for $C_{24}H_{29}BClO_2$ [M+H$^+$] 395.1944, found 395.1946. **Optical rotation:** $[\alpha]_D^{20}$ + 60.4 (c=0.75, $CHCl_3$).

Enantiomeric purity was determined by chiral SFC analysis [Lux Cellulose-1, 100 bar, T_{oven}: 40 °C, Flow: 1.4 mL/min; 1% MeOH, λ =220 nm, major enantiomer t_R = 65.72 min, minor enantiomer t_R = 68.83 min].

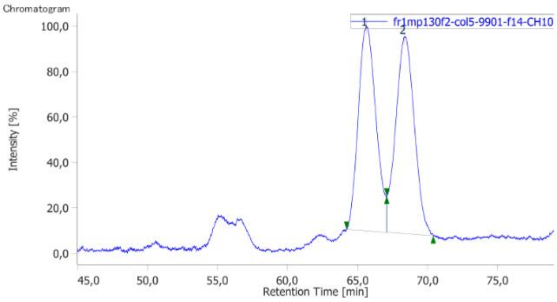

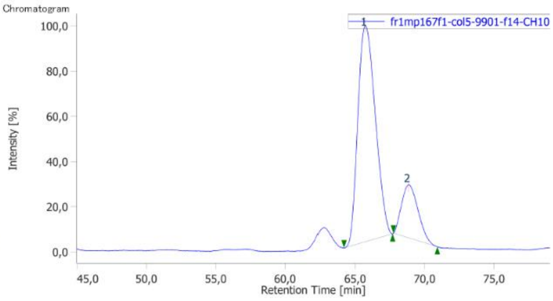

Peak Information

#	Peak Name	CH	tR [min]	Area [µV·sec]	Area%
1	Unknown	10	65.577	791935	49.651
2	Unknown	10	68.317	803073	50.349

Peak Information

#	Peak Name	CH	tR [min]	Area [µV·sec]	Area%
1	Unknown	10	65.720	7129443	81.151
2	Unknown	10	68.827	1655988	18.849

Note: Chromatogram of racemic compound shows peaks for *Z*,*E*-isomer (not separated) and *Z*,*Z*-isomer (65.58 and 68.32 min).

2-((*S*,1*Z*,5*Z*)-6-Chloro-1-cyclohexyl-4-(p-tolyl)hexa-1,5-dien-2-yl)-4,4,5,5-tetramethyl-1,3,2-dioxaborolane (14)

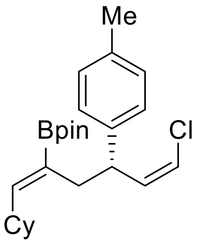

Synthesized from **1**, **42** and B_2Pin_2 according to general procedure A. Yellow oil obtained in 66% yield with 96:4 er after column chromatography (hexane:CH_2Cl_2, 90:10 to 80:20). This compound was isolated together with 7% of the dechlorinated product. **^{1}H NMR** (500 MHz, $CDCl_3$) δ 7.16 (d, *J* = 8.0 Hz, 2H), 7.10 (d, *J* = 7.6 Hz, 2H), 6.13 (d, *J* = 9.9 Hz, 1H), 6.01 (d, *J* = 7.1 Hz, 1H), 5.96 (dd, *J* = 9.6, 7.1 Hz, 1H), 4.04 – 3.96 (m, 1H), 2.56 – 2.51 (m, 2H), 2.35 (m, 1H), 2.31 (s, 3H), 1.76 – 1.57 (m, 6H), 1.23 (s, 6H), 1.22 (s, 6H), 1.17 – 0.99 (m, 4H). **^{13}C NMR** (126 MHz, $CDCl_3$) δ 153.2, 140.7, 135.8, 135.4, 129.2, 127.6, 117.5, 83.2, 44.2, 38.0, 35.1, 32.7, 32.6, 26.2, 26.0, 25.9, 25.0, 24.8, 21.1. **^{11}B NMR** (160 MHz, $CDCl_3$) δ 30.50. **HRMS (APCI)** Calc. For $C_{25}H_{37}BClO_2$ [$M+H^+$] 415.2570, found 415.2568. **Optical rotation:** $[\alpha]_D^{22}$ + 64.7 (c=0.65, $CHCl_3$).

Enantiomeric purity was determined by chiral SFC analysis [Lux Cellulose-1, 100 bar, T_{oven}: 40 °C, Flow: 1.2 mL/min; 1% MeOH, λ =220 nm, major enantiomer t_R = 33.39 min, minor enantiomer t_R = 35.88 min].

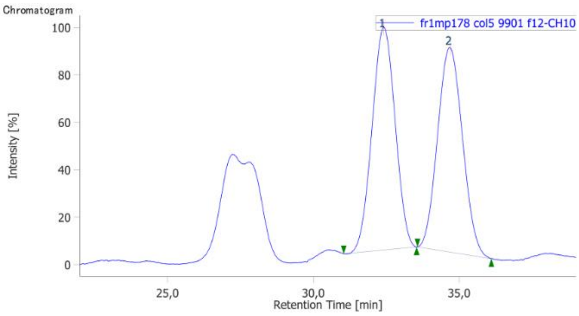

Peak Information

#	Peak Name	CH	tR [min]	Area [µV·sec]	Area%
1	Unknown	10	32.410	2840124	50.204
2	Unknown	10	34.687	2817047	49.796

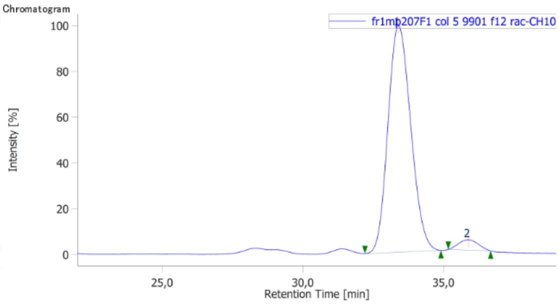

Peak Information

#	Peak Name	CH	tR [min]	Area [µV·sec]	Area%
1	Unknown	10	33.390	13467691	96.173
2	Unknown	10	35.883	535948	3.827

Note: Chromatogram of racemic compound shows peaks for *Z*,*E*-isomer (not separated) and *Z*,*Z*-isomer (32.41 and 34.69 min).

2-((*S*,1*Z*,5*Z*)-6-Chloro-1-cyclohexyl-4-(4-(trifluoromethyl)phenyl)hexa-1,5-dien-2-yl)-4,4,5,5-tetramethyl-1,3,2-dioxaborolane (15)

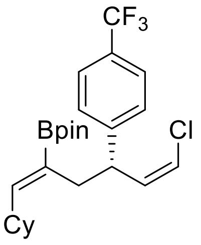

Synthesized from **1**, **43** and B_2Pin_2 according to general procedure A at 60 °C (18 h). Yellow oil obtained in 43% yield with 93:7 er after column chromatography (hexane:CH_2Cl_2, 90:10 to 85:15). **^{1}H NMR** (500 MHz, $CDCl_3$) δ 7.53 (d, *J* = 8.1 Hz, 2H), 7.36 (d, *J* = 8.0 Hz, 2H), 6.14 (d, *J* = 10.0 Hz, 1H), 6.08 (d, *J* = 7.1 Hz, 1H), 5.99 (dd, *J* = 9.6, 7.0 Hz, 1H), 4.08 (dt, *J* = 9.7, 7.7 Hz, 1H), 2.55 (d, *J* = 7.6 Hz, 2H), 2.34 – 2.24 (m, 1H), 1.76 – 1.55 (m, 4H), 1.20 (m, 18H). **^{13}C NMR** (126 MHz, $CDCl_3$) δ 153.8, 147.7, 134.3, 128.6, 128.3, 125.4 (q, *J* = 3.9 Hz), 118.6, 83.3, 44.6, 38.1, 35.1, 32.7, 32.5, 26.1, 26.0, 25.9, 25.0, 24.7. *Quaternary C heterocoupled with F could not be seen. **^{11}B NMR** (160 MHz, $CDCl_3$) δ 30.1. **^{19}F NMR** (282 MHz, $CDCl_3$) δ -62.45. **HRMS (APCI)** Calc. for $C_{25}H_{34}BClF_3O_2$ [$M+H^+$] 469.2287, found 469.2295. **Optical rotation:** $[\alpha]_D^{21}$ + 30.2 (c=0.52, $CHCl_3$).

Enantiomeric purity was determined on oxidation product **27** (see section 7.2).

2-((*S*,1*Z*,5*Z*)-6-Chloro-4-(3-chlorophenyl)-1-cyclohexylhexa-1,5-dien-2-yl)-4,4,5,5-tetramethyl-1,3,2-dioxaborolane (16)

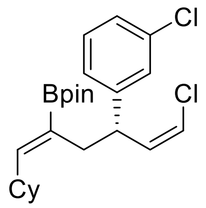

Synthesized from **1**, **44** and B_2Pin_2 according to general procedure A. Yellow oil obtained in 57% yield with 98:2 er after column chromatography (hexane:CH_2Cl_2, 90:10 to 80:20). **^{1}H NMR** (500 MHz, $CDCl_3$) δ 7.25 (t, *J* = 1.9 Hz, 1H), 7.19 (d, *J* = 7.6 Hz, 1H), 7.16 (t, *J* = 1.7 Hz, 1H), 7.12 (dt, *J* = 7.4, 1.5 Hz, 1H), 6.13 (d, *J* = 9.9 Hz, 1H), 6.05 (d, *J* = 7.1 Hz, 1H), 5.94 (dd, *J* = 9.6, 7.1 Hz, 1H), 4.02 (dt, *J* = 9.8, 7.8 Hz, 1H), 2.59 – 2.47 (m, 2H), 2.36 – 2.23 (m, 1H), 1.77 – 1.52 (m, 4H), 1.29 – 1.13 (m, 18H). **^{13}C NMR** (126 MHz, $CDCl_3$) δ 152.50, 144.55, 133.39, 133.12, 128.54, 126.72, 125.40, 125.16, 117.20, 82.10, 43.12, 36.94, 33.81, 31.53, 31.44, 24.99, 24.84, 24.82, 23.85, 23.62. **^{11}B NMR** (160 MHz, $CDCl_3$) δ 29.6. **HRMS (APCI)** Calc. For $C_{24}H_{34}BCl_2O_2$ [M+H$^+$] 435.2023, found 435.2030. **Optical rotation:** $[\alpha]^{21}_{D}$ + 52.6 (c=0.71, $CHCl_3$).

Enantiomeric purity was determined on oxidation product **28** (see section 7.2).

2-((*S*,1*Z*,5*Z*)-9-(Benzyloxy)-1-chloro-3-(3-chlorophenyl)nona-1,5-dien-5-yl)-4,4,5,5-tetramethyl-1,3,2-dioxaborolane (17)

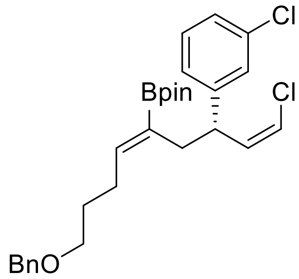

Synthesized from **37**, **44** and B_2Pin_2 according to general procedure A. Yellow oil obtained in 40% yield with 93:7 er after column chromatography (hexane:AcOEt, 99:1 to 94:6). **^{1}H NMR** (300 MHz, $CDCl_3$) δ 7.40 – 7.25 (m, 6H), 7.23 – 7.11 (m, 3H), 6.38 (t, *J* = 7.1 Hz, 1H), 6.06 (d, *J* = 7.1 Hz, 1H), 6.00 – 5.88 (m, 1H), 4.53 (s, 2H), 4.08 (q, *J* = 8.2 Hz, 1H), 3.57 – 3.44 (m, 2H), 2.57 (d, *J* = 7.6 Hz, 2H), 2.25 (q, *J* = 7.5 Hz, 2H), 1.71 (ddd, *J* = 13.8, 9.1, 5.3 Hz, 2H), 1.26 (s, 12H). **^{13}C NMR** (75 MHz, $CDCl_3$) δ 147.6, 145.8, 138.8, 134.4, 134.3, 129.7, 128.5, 127.9, 127.7, 127.6, 126.6, 126.1, 118.6, 83.4, 73.0, 70.0, 43.7, 34.8, 29.2, 25.7, 25.0, 24.8. **^{11}B NMR** (160 MHz, $CDCl_3$) δ 30.1. **HRMS (APCI)** Calc. for $C_{28}H_{36}BCl_2O_3$ [M+H$^+$] 501.2129, found 501.2132. **Optical rotation:** $[\alpha]^{21}_{D}$ + 44.0 (c=0.75, $CHCl_3$).

Enantiomeric purity was determined on oxidation product **29** (see section 7.2).

2-((*S*,1*Z*,5*Z*)-4-(2-Bromophenyl)-6-chloro-1-cyclohexylhexa-1,5-dien-2-yl)-4,4,5,5-tetramethyl-1,3,2-dioxaborolane (18)

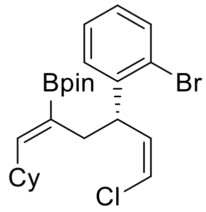

Synthesized from **1**, **45** and B_2Pin_2 according to general procedure A. Yellow oil obtained in 62% yield with 95.5:4.5 er after column chromatography (hexane:CH_2Cl_2, 90:10 to 80:20). **^{1}H NMR** (500 MHz, $CDCl_3$) δ 7.45 (dd, *J* = 8.0, 1.3 Hz, 1H), 7.24 (dd, *J* = 7.8, 1.8 Hz, 1H), 7.20 – 7.17 (m, 1H), 6.96 (td, *J* = 7.6, 1.8 Hz, 1H), 6.08 (d, *J* = 10.0 Hz, 1H), 5.99 – 5.95 (m, 2H), 4.41 (m, 1H), 2.55 – 2.46 (m, 1H), 2.43 – 2.31 (m, 2H), 1.60 (m, 4H), 1.52 – 1.47 (m, 2H), 1.18 (s, 6H), 1.17 (s, 6H), 1.11 – 0.92 (m, 4H). **^{13}C NMR** (126 MHz, $CDCl_3$) δ 154.14, 143.32, 134.23, 133.17, 129.23, 127.76, 127.60, 124.49, 118.82, 83.27, 43.80, 38.00, 34.60, 32.63, 26.18, 26.06, 26.02, 25.06, 24.87. **^{11}B NMR** (160 MHz, $CDCl_3$) δ 30.4. **HRMS (APCI)** Calc. for $C_{24}H_{34}BBrClO_2$ [M+H$^+$] 479.1518, found 479.1525. **Optical rotation:** $[\alpha]^{21}_{D}$ + 20.5 (c=0.70, $CHCl_3$).

Enantiomeric purity was determined on oxidation product **30** (see section 7.2).

2-((*S*,2*Z*,6*Z*)-5-(2-Bromophenyl)-7-chloro-1-phenylhepta-2,6-dien-3-yl)-4,4,5,5-tetramethyl-1,3,2-dioxaborolane (19)

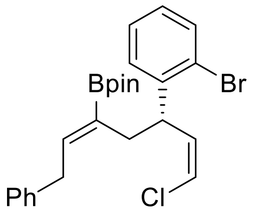

Synthesized from **34**, **45** and B_2Pin_2 according to general procedure A. Yellow oil obtained in 61 % yield with 92:8 er after column chromatography (hexane:CH_2Cl_2, 90:10 to 80:20). **1H NMR** (500 MHz, Chloroform-d) δ 7.46 (dd, J = 8.1, 1.3 Hz, 1H), 7.26 (dd, J = 7.8, 1.7 Hz, 1H), 7.19 (ddt, J = 7.2, 5.0, 2.7 Hz, 3H), 7.14 – 7.06 (m, 3H), 6.97 (td, J = 7.6, 1.7 Hz, 1H), 6.44 (t, J = 7.2 Hz, 1H), 6.01 – 5.96 (m, 2H), 4.54 (dtd, J = 8.5, 6.6, 1.7 Hz, 1H), 3.43 (dd, J = 7.2, 4.4 Hz, 2H), 2.63 (dd, J = 13.0, 6.7 Hz, 1H), 2.57 (dd, J = 12.9, 8.8 Hz, 1H), 1.16 (s, 6H), 1.15 (s, 6H). **^{13}C NMR** (126 MHz, $CDCl_3$) δ 146.4, 143.0, 140.3, 133.8, 133.1, 129.1, 128.7, 128.4, 127.8, 127.6, 126.0, 124.4, 119.0, 83.3, 43.1, 35.2, 34.4, 24.9, 24.8. **^{11}B NMR** (160 MHz, $CDCl_3$) δ 32.7. **HRMS (APCI)** Calc. for $C_{25}H_{30}BBrClO_2$ [M+H$^+$] 487.1205, found 487.1211. **Optical rotation:** $[\alpha]_D^{22}$ + 20.8 (c=0.51, $CHCl_3$).

Enantiomeric purity was determined on Suzuki cross-coupling product **21** (see section 7.1.1).

2-((*S*,1*Z*,5*Z*)-6-Chloro-1-cyclohexyl-4-(naphthalen-2-yl)hexa-1,5-dien-2-yl)-4,4,5,5-tetramethyl-1,3,2-dioxaborolane (20)

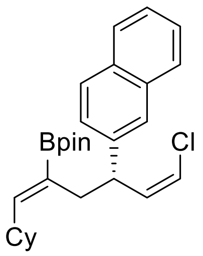

Synthesized from **1**, **46** and B_2Pin_2 according to general procedure A. Yellow oil obtained in 63% yield with 95:5 er after column chromatography (hexane:CH_2Cl_2, 95:5 to 85:15). **^{1}H NMR** (500 MHz, $CDCl_3$) δ 7.79 (td, *J* = 8.8, 7.7, 4.6 Hz, 3H), 7.69 (d, *J* = 1.7 Hz, 1H), 7.47 – 7.37 (m, 3H), 6.15 (d, *J* = 9.9 Hz, 1H), 6.12 – 6.05 (m, 2H), 4.28 – 4.16 (m, 1H), 2.66 (m, 2H), 2.37 (m, 1H), 1.78 – 1.60 (m, 4H), 1.18 (m, 18H). **^{13}C NMR** (126 MHz, $CDCl_3$) δ 153.4, 141.1, 135.1, 133.7, 132.5, 128.1, 127.8, 127.6, 126.6, 126.0, 125.9, 125.4, 118.0, 83.2, 44.7, 38.1, 35.1, 32.7, 32.6, 26.2, 26.0, 25.9, 25.0, 24.7. **^{11}B NMR** (160 MHz, $CDCl_3$) δ 30.3. **HRMS (APCI)** Calc. For $C_{28}H_{37}BClO_2$ [M+H$^+$] 451.2570, found 451.2582. **Optical rotation:** $[\alpha]_D^{21}$ + 67.4 (c=0.68, $CHCl_3$).

Enantiomeric purity was determined by chiral SFC analysis [Lux Cellulose-1, 100 bar, T_{oven}: 40 °C, Flow: 2 mL/min; 2% MeOH, λ =220 nm, major enantiomer t_R = 33.97 min, minor enantiomer t_R = 37.86 min].

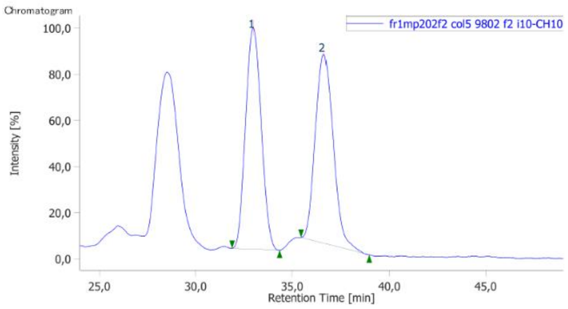

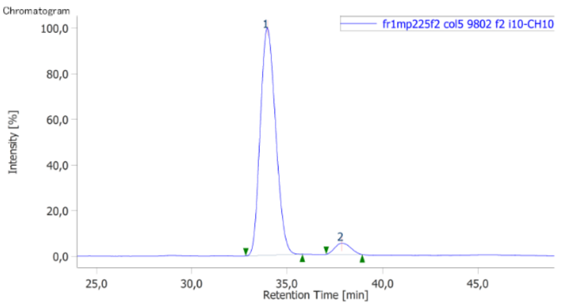

Peak Information

#	Peak Name	CH	tR [min]	Area [μV·sec]	Area%
1	Unknown	10	32.980	11723459	51.234
2	Unknown	10	36.617	11158736	48.766

Peak Information

#	Peak Name	CH	tR [min]	Area [μV·sec]	Area%
1	Unknown	10	33.970	50222077	95.170
2	Unknown	10	37.857	2548577	4.830

Note: Chromatogram of racemic compound shows peaks for *Z*,*E*-isomer (not separated) and *Z*,*Z*-isomer (32.98 and 36.62 min).

7. Product derivatizations

7.1. Suzuki-Miyaura cross-couplings

7.1.1. Intermolecular Suzuki-Miyaura cross-couplings

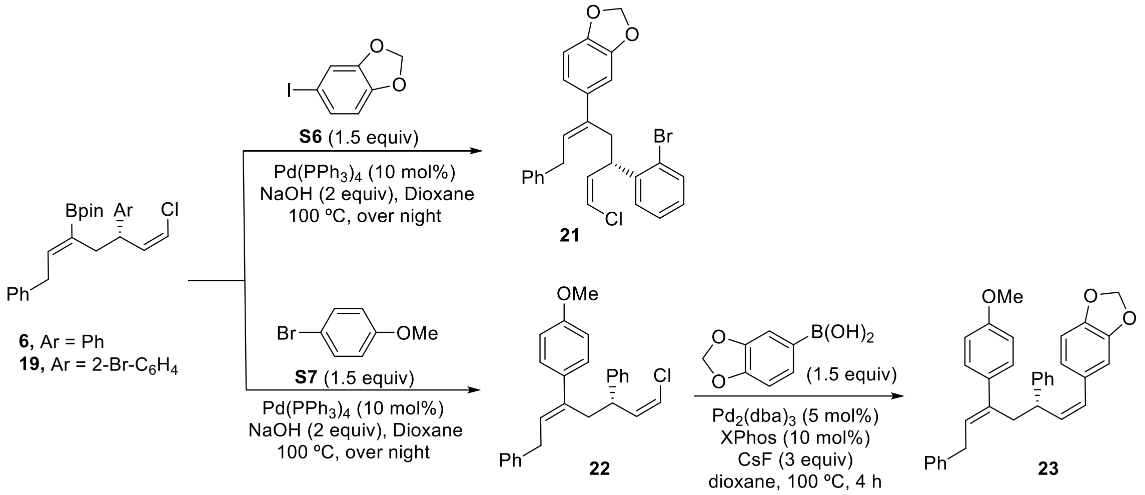

$Pd(PPh_3)_4$ (10 mol%, 0.01 mmol) was added to a Schlenk flask. Then, the corresponding alkenylboronate **6** or **19** (1.0 equiv, 0.1 mmol) was added in 1,4-dioxane (1.0 mL), followed by the addition of the corresponding aryl halide **S6** or **S7** (1.5 equiv, 0.15 mmol) and aq. NaOH (2.0 M, 2.0 equiv, 0.2 mmol). The reaction was stirred at 100 °C for 18 h. After this time, the mixture was diluted with CH_2Cl_2 (5 ml) and washed with saturated aqueous solution of NH_4Cl (2x5 mL). Then, the aqueous layer was extracted with CH_2Cl_2 (5 mL). Combined organic layer were dried over anhydrous Na_2SO_4, filtered and solvent was removed under reduced pressure. Crude product was purified through flash column chromatography using the indicated mixture of solvents as eluent to afford the corresponding product **21** or **22**.

5-((*S*,2*E*,6*Z*)-5-(2-Bromophenyl)-7-chloro-1-phenylhepta-2,6-dien-3-yl)benzo[d][1,3]dioxole (21)

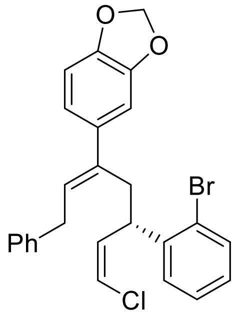

Synthesized from **19** and **S6**. Yellow oil obtained in 68% yield with 92:8 er after column chromatography (hexane:CH_2Cl_2, 90:10 to 80:20).**^{1}H NMR** (300 MHz, $CDCl_3$) δ 7.51 (d, *J* = 7.3 Hz, 1H), 7.32 – 7.24 (m, 2H), 7.22 – 7.15 (m, 4H), 7.09 – 6.95 (m, 2H), 6.85 – 6.80 (m, 2H), 6.74 (d, *J* = 8.5 Hz, 1H), 6.04 – 5.97 (m, 2H), 5.95 (s, 2H), 5.76 (t, *J* = 7.4 Hz, 1H), 4.50 (q, *J* = 8.3 Hz, 1H), 3.51 (d, *J* = 7.4 Hz, 2H), 3.04 (dd, *J* = 14.0, 6.8 Hz, 1H), 2.94 (dd, *J* = 14.0, 8.5 Hz, 1H). **^{13}C NMR** (75 MHz, $CDCl_3$) δ 147.60, 146.69, 142.41, 140.98, 138.03, 137.19, 133.35, 133.12, 129.29, 129.21, 128.60, 128.57, 128.08, 127.64, 126.16, 124.26, 120.47, 119.50, 108.03, 107.77, 101.26, 101.04, 42.13, 35.81, 34.96, 29.86. **HRMS (APCI)** Calc. For $C_{26}H_{23}BrClO_2$ [M+H$^+$] 481.0564, found 481.0549 **Optical rotation:** $[\alpha]_D^{20}$ - 9.8 (c=1.1, $CHCl_3$).

Enantiomeric purity was determined by chiral SFC analysis [Lux Cellulose-1, 100 bar, T_{oven}: 40 °C, Flow: 2 mL/min; 10% MeOH, λ =220 nm, minor enantiomer t_R = 28.91 min, major enantiomer t_R = 29.85 min].

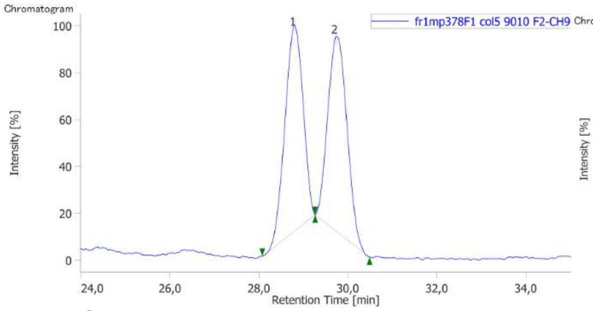

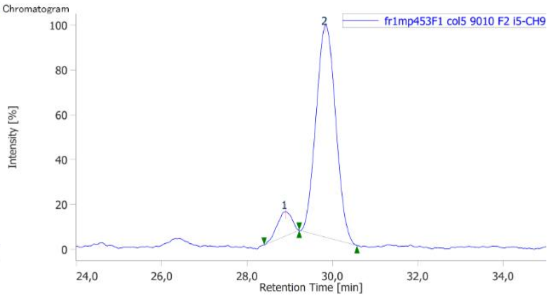

Peak Information

#	Peak Name	CH	tR [min]	Area [μV·sec]	Area%
1	Unknown	9	28,797	1225924	49,931
2	Unknown	9	29,733	1229318	50,069

Peak Information

#	Peak Name	CH	tR [min]	Area [μV·sec]	Area%
1	Unknown	9	28,910	151162	7,916
2	Unknown	9	29,847	1758519	92,084

((*S*,2*E*,6*Z*)-7-Chloro-3-(4-methoxyphenyl)hepta-2,6-diene-1,5-diyl)dibenzene (22)

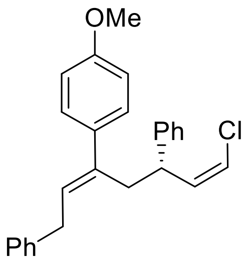

Synthesized from **6** and **S7**. Yellow oil obtained in 71% yield with 96:4 er after column chromatography (hexane:CH_2Cl_2, 80:20 to 70:30).

^{1}H NMR (300 MHz, $CDCl_3$) δ 7.26 (d, *J* = 12.8 Hz, 6H), 7.18 (t, *J* = 8.1 Hz, 4H), 7.08 (d, *J* = 7.3 Hz, 2H), 6.90 – 6.83 (m, 2H), 6.04 – 5.91 (m, 2H), 5.71 (t, *J* = 7.3 Hz, 1H), 4.00 (q, *J* = 7.9 Hz, 1H), 3.83 (s, 3H), 3.39 (qd, *J* = 16.2, 7.4 Hz, 2H), 3.01 (d, *J* = 7.5 Hz, 2H). **^{13}C NMR** (75 MHz, $CDCl_3$) δ 158.9, 143.0, 141.1, 137.9, 135.3, 134.5, 128.6, 128.6, 128.5, 128.3, 128.0, 127.7, 126.7, 126.1, 118.3, 113.7, 55.4, 42.5, 36.5, 34.9. **HRMS (APCI)** Calc. For $C_{26}H_{26}ClO$ [M+H$^+$] 389.1667, found 389.1656. **Optical rotation:** $[\alpha]_D^{21}$ + 1.7 (c=1.15, $CHCl_3$).

Enantiomeric purity was determined by chiral SFC analysis [Lux Amilose-1, 100 bar, T_{oven}: 40 °C, Flow: 1 mL/min; 3% MeOH, λ =220 nm, major enantiomer t_R = 81.94 min, minor enantiomer t_R = 87.26 min].

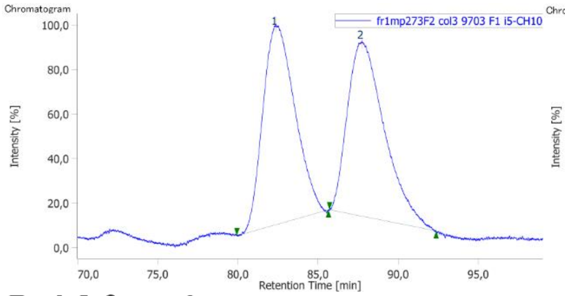

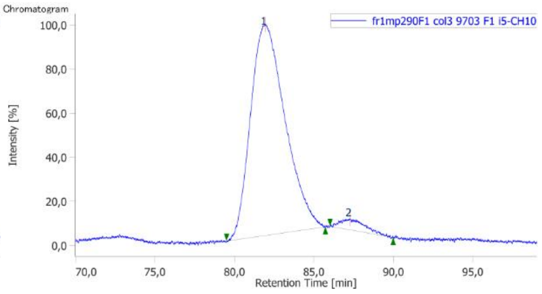

Peak Information

#	Peak Name	CH	tR [min]	Area [μV·sec]	Area%
1	Unknown	10	82,373	2414574	49,709
2	Unknown	10	87,733	2442892	50,291

Peak Information

#	Peak Name	CH	tR [min]	Area [μV·sec]	Area%
1	Unknown	10	81,940	2577304	96,313
2	Unknown	10	87,263	98668	3,687

5-((*S*,1*Z*,5*E*)-5-(4-Methoxyphenyl)-3,7-diphenylhepta-1,5-dien-1-yl)benzo[d][1,3]dioxole (23)

A dry Schlenk flask under argon atmosphere was charged with dicyclohexylphosphino-2',4',6'-triisopropylbiphenyl (XPhos) (10 mol%, 0.01 mmol), tris(dibenzylideneacetone)dipalladium(0) (5 mol%, 0.005mmol) and cesium fluoride (3 equiv, 0.3 mmol). Then, a solution of **21** (1.0 equiv, 0.1 mmol) in 1,4 dioxane (1 ml) was added, followed by the addition of benzo[d][1,3]dioxol-5-ylboronic acid (1.5 equiv, 0.15 mmol). The reaction was stirred at 100 °C for 4 h. After this time, the mixture was diluted with CH_2Cl_2 (5 ml) and washed with saturated aqueous solution of NH_4Cl (2x5 mL). Then, the aqueous layer was extracted with CH_2Cl_2 (5 mL). The combined organic layers were dried over anhydrous Na_2SO_4, filtered and solvent was removed under reduced pressure. Crude product was purified through flash column chromatography (hexane:CH_2Cl_2, 80:20 to 60:40) affording product **23** as a yellow oil in 62% yield with 93:7 er.

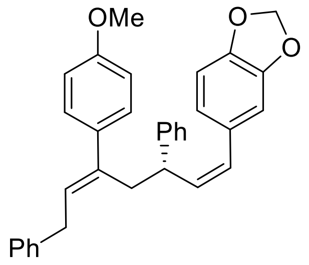

^{1}H NMR (300 MHz, $CDCl_3$) δ 7.33 – 7.25 (m, 4H), 7.22 (t, *J* = 7.0 Hz, 4H), 7.17 – 7.04 (m, 4H), 6.76 (d, *J* = 8.4 Hz, 2H), 6.64 (d, *J* = 8.4 Hz, 1H), 6.53 (s, 1H), 6.35 (d, *J* = 11.5 Hz, 1H), 5.92 (s, 2H), 5.81 (t, *J* = 11.1 Hz, 1H), 5.68 (t, *J* = 7.2 Hz, 1H), 3.96 (dt, *J* = 10.4, 7.3 Hz, 1H), 3.81 (s, 3H), 3.51 – 3.24 (m, 2H), 3.02 – 2.96 (m, 2H). **^{13}C NMR** (75 MHz, $CDCl_3$) δ 158.6, 147.4, 146.3, 144.7, 141.2, 138.2, 135.3, 134.2, 131.4, 128.9, 128.7, 128.6, 128.5, 127.9, 127.6, 126.4, 126.0, 122.3, 113.9, 113.6, 109.0, 108.0, 100.9, 55.3, 42.5, 38.2, 34.9. **HRMS (APCI)** Calc. For $C_{33}H_{31}O_3$ [M+H$^+$] 475.2268, found 475.2259. **Optical rotation:** $[\alpha]_D^{21}$ + 41.0 (c=0.81, $CHCl_3$).

Enantiomeric purity was determined by chiral uHPLC analysis [Lux i-amilose-3, T_{oven}: 40 °C, Flow: 0.5 mL/min; 97% hexane, λ =250nm, major enantiomer t_R = 11.58 min, minor enantiomer t_R = 12.09 min].

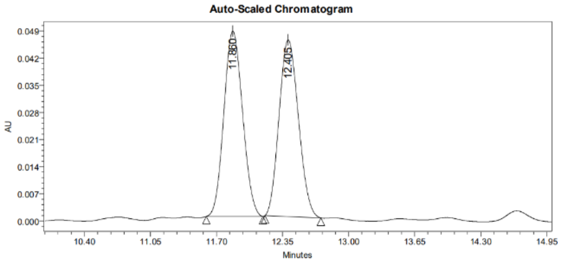

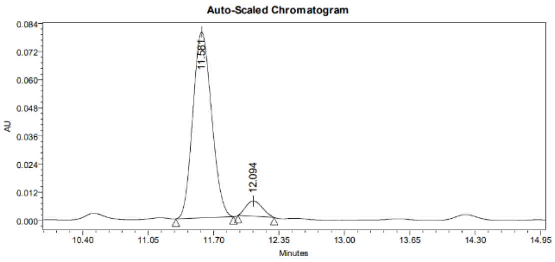

Unknown Peak Results

	RT	Area	% Area	Height
1	11.860	611577	50.34	47828
2	12.405	603375	49.66	45586

Unknown Peak Results

	RT	Area	% Area	Height
1	11.581	1025918	93.24	79275
2	12.094	74364	6.76	6522

7.1.2. Intramolecular Suzuki-Miyaura cross-couplings

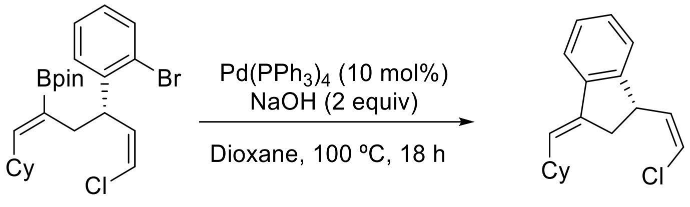

18 **24**

$Pd(PPh_3)_4$ (10 mol%, 0.02 mmol) was added to a Schlenk flask. Then, **18** (1.0 equiv, 0.2 mmol) was added in 1,4-dioxane (4.0 mL), followed by the addition of aq. NaOH (2.0 M, 2.0 equiv, 0.4 mmol). The reaction was stirred at 100 °C for 5 h. After this time, the mixture was diluted with CH_2Cl_2 (5 ml) and washed with saturated aqueous solution of NH_4Cl (2x5 mL). Then, the aqueous layer was extracted with CH_2Cl_2 (5 mL). Combined organic layer were dried over anhydrous Na_2SO_4, filtered and solvent was removed under reduced pressure. Crude product was purified through flash column chromatography (hexane) affording the desired product as a yellow oil in 83% yield and with 92:8 er. This compound was isolated together with 8% of the dechlorinated product.

(*S*,*E*)-1-((*Z*)-2-Chlorovinyl)-3-(cyclohexylmethylene)-2,3-dihydro-1*H*-indene (24)

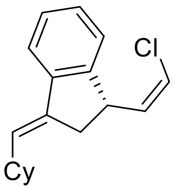

^{1}H NMR (300 MHz, $CDCl_3$) δ 7.45 – 7.41 (m, 1H), 7.21 – 7.17 (m, 3H), 6.16 (d, *J* = 7.0 Hz, 1H), 5.82 (td, *J* = 6.7, 3.4 Hz, 2H), 4.46 (td, *J* = 8.9, 5.6 Hz, 1H), 3.15 (ddd, *J* = 16.7, 8.8, 2.4 Hz, 1H), 2.46 (ddd, *J* = 16.4, 5.7, 2.6 Hz, 1H), 2.24 (q, *J* = 10.4 Hz, 1H), 1.84 – 1.65 (m, 4H), 1.38 – 1.12 (m,5H). **^{13}C NMR** (75 MHz, $CDCl_3$) δ 146.5, 141.7, 138.3, 135.3, 127.8, 127.3, 126.4, 125.2, 120.0, 118.1, 40.8, 38.9, 35.3, 33.2, 33.0, 26.3, 26.2. **HRMS (APCI)** Calc. For $C_{18}H_{22}Cl$ [M+H$^+$] 273.1405, found 273.1394. **Optical rotation:** $[\alpha]_D^{21}$ + 75.0 (c=0.88, $CHCl_3$).

Enantiomeric purity was determined by chiral uHPLC analysis [Lux i-amylose-3, T_{oven}: 40 °C, Flow: 0.5 mL/min; 99.5% hexane, λ =220 nm, minor enantiomer t_R = 6.84 min, major enantiomer t_R = 7.02 min].

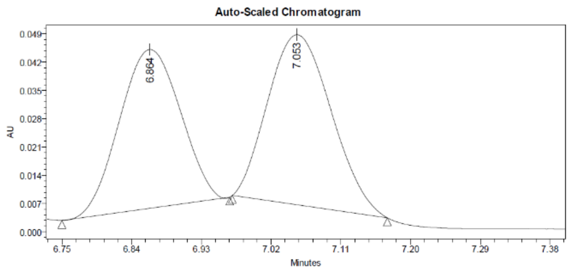

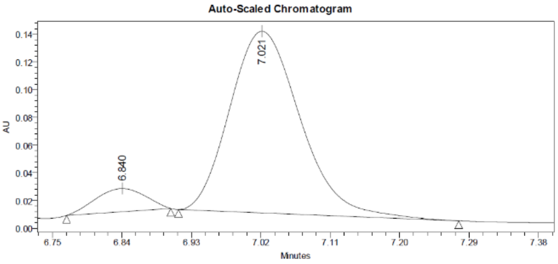

Unknown Peak Results

	RT	Area	% Area	Height
1	6.864	214667	47.14	39291
2	7.053	240731	52.86	42082

Unknown Peak Results

	RT	Area	% Area	Height
1	6.840	75298	8.30	16628
2	7.021	831744	91.70	131079

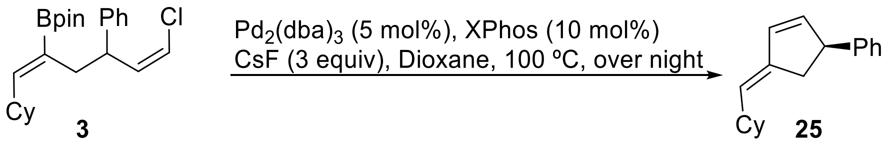

A dry Schlenk flask under argon atmosphere was charged with dicyclohexylphosphino-2',4',6'-triisopropylbiphenyl (XPhos) (10 mol%, 0.01 mmol), tris(dibenzylideneacetone)dipalladium(0) (5 mol%, 0.005mmol) and cesium fluoride (3 equiv, 0.3 mmol). Then, **3** (1.0 equiv, 0.1 mmol) was added in 1,4- dioxane (2 ml) and the reaction was stirred at 100 °C for 18 h. After this time, the mixture was diluted with CH_2Cl_2 (5 ml) and washed with saturated aqueous solution of NH_4Cl (2x5 mL). Then, the aqueous layer was extracted with CH_2Cl_2 (5 mL). Combined organic layers were dried over anhydrous Na_2SO_4, filtered and solvent was removed under reduced pressure. Crude product was purified through flash column chromatography (hexane) affording product **25** as a yellow oil in 53% yield and with 95:5 er.

(*S*,*E*)-(4-(Cyclohexylmethylene)cyclopent-2-en-1-yl)benzene (25)

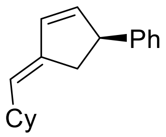

^{1}H NMR (500 MHz, $CDCl_3$) δ 7.25 – 7.19 (m, 2H), 7.15 – 7.07 (m, 3H), 6.16 (dd, *J* = 5.3, 2.3 Hz, 1H), 5.91 (dd, *J* = 5.5, 2.6 Hz, 1H), 5.18 (dd, *J* = 9.1, 2.6 Hz, 1H), 3.99 – 3.93 (m, 1H), 2.97 (ddd, *J* = 16.8, 8.7, 2.3 Hz, 1H), 2.32 (dt, *J* = 16.9, 3.1 Hz, 1H), 2.02 – 1.89 (m, 1H), 1.67 – 1.51 (m, 6H), 1.23 – 1.07 (m, 4H). **^{13}C NMR** (126 MHz, $CDCl_3$) δ 146.2, 144.1, 138.7, 135.4, 128.6, 127.3, 126.9, 126.3, 50.6, 39.0, 36.7, 33.2, 33.1, 26.3, 26.2, 26.2. **HRMS (APCI)** Calc. For $C_{18}H_{23}$ [M+H$^+$] 239.1794, found 239.2. **Optical rotation:** $[\alpha]^{21}_{D}$ – 33.7 (c=1.07, $CHCl_3$).

Enantiomeric purity was determined by chiral uHPLC analysis [Lux i-amilose-3, T_{oven}: 40 °C, Flow: 1 mL/min; 100% hexane, λ =209.5 nm, major enantiomer t_R = 3.71 min, minor enantiomer t_R = 4.17min].

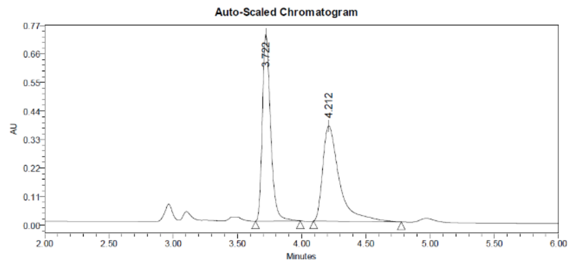

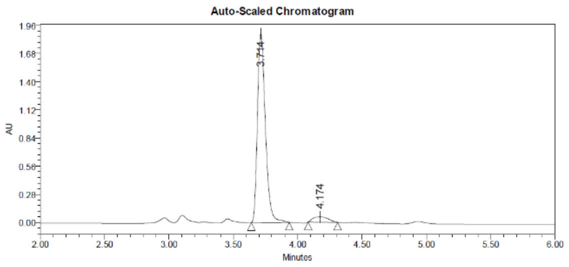

Unknown Peak Results

	RT	Area	% Area	Height
1	3.722	3212266	49.34	720211
2	4.212	3298581	50.66	367748

Unknown Peak Results

	RT	Area	% Area	Height
1	3.714	8042955	94.83	1880434
2	4.174	438647	5.17	53308

7.2. Oxidation of alkenyl boronates

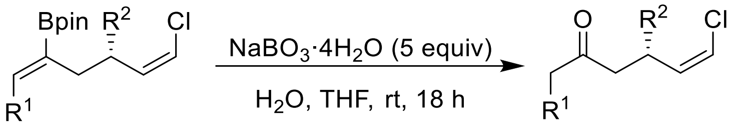

General procedure B:

A solution of $NaBO_3{\cdot}4H_2O$ (5 equiv) in H_2O [1M] was added to a solution of the alkenyl boronate in THF [0.2 M]. The reaction mixture was then stirred at room temperature over 18 h. After this time, the mixture was diluted with CH_2Cl_2 (5 ml) and washed with saturated aqueous solution of NH_4Cl (2x5 mL). Then, the aqueous layer was extracted with CH_2Cl_2 (5 mL). Combined organic layers were dried over anhydrous Na_2SO_4, filtered and solvent was removed under reduced pressure. Crude product was purified through flash column chromatography using the indicated mixture of solvents as eluent.

(*S*,*Z*)-9-(Benzyloxy)-1-chloro-3-phenylnon-1-en-5-one (26)

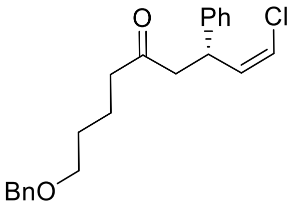

Synthesized from **9** according to general procedure B. Yellow oil obtained in 65% yield with 95.5:4.5 er after column chromatography (hexane:AcOEt, 95:5 to 90:10). **^{1}H NMR** (300 MHz, $CDCl_3$) δ 7.27 – 7.22 (m, 5H), 7.20 – 7.15 (m, 4H), 6.01 (d, *J* = 7.2 Hz, 1H), 5.84 (dd, *J* = 9.4, 7.2 Hz, 1H), 4.41 (s, 2H), 4.36 – 4.33 (m, 1H), 3.36 (t, *J* = 6.1 Hz, 2H), 2.83 (dd, *J* = 16.0, 7.1 Hz, 1H), 2.71 (dd, *J* = 16.0, 7.6 Hz, 1H), 2.32 (t, *J* = 7.0 Hz, 2H), 1.60 – 1.44 (m, 4H). **^{13}C NMR** (75 MHz, $CDCl_3$) δ 208.4, 142.3, 138.7, 133.9, 128.9, 128.5, 127.8, 127.7, 127.4, 126.9, 118.6, 73.0, 70.1, 48.4, 42.9, 39.1, 29.2, 20.5. **HRMS (APCI)** Calc. For $C_{22}H_{26}ClO_2$ [M+H$^+$] 357.1616, found 357.1624. **Optical rotation:** $[\alpha]^{21}_{D}$ + 64.9 (c=0.64, $CHCl_3$).

Enantiomeric purity was determined by chiral uHPLC analysis [Lux i-celulose-5, T_{oven}: 40 °C, Flow: 0.5 mL/min; 97% hexane, λ =209.5 nm, minor enantiomer t_R = 20.59 min, major enantiomer t_R = 21.09 min].

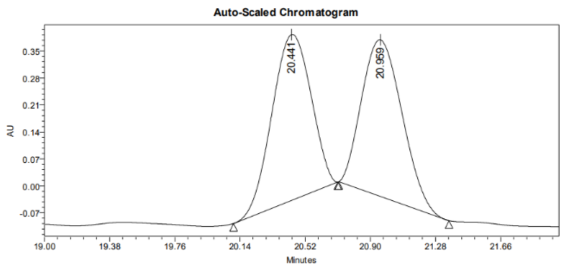

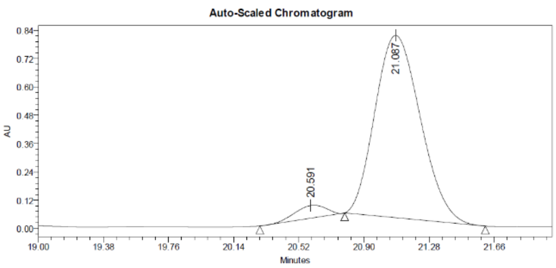

Unknown Peak Results

	RT	Area	% Area	Height
1	20.441	6803238	50.49	430735
2	20.959	6672291	49.51	406640

Unknown Peak Results

	RT	Area	% Area	Height
1	20.591	674750	4.52	53462
2	21.087	14247484	95.48	773259

(*S*,*Z*)-6-Chloro-1-cyclohexyl-4-(4-(trifluoromethyl)phenyl)hex-5-en-2-one (27)

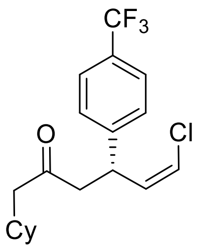

Synthesized from **15** according to general procedure B. Yellow oil obtained in 82% yield with 93:7 er after column chromatography (hexane:CH_2Cl_2, 80:20 to 70:30), as a 16:1 *Z*:*E* mixture of diastereomers. NMR data is only given for the major diastereomer. This compound was isolated together with 14% of the dechlorinated product. **^{1}H NMR** (500 MHz, $CDCl_3$) δ 7.55 (d, *J* = 8.0 Hz, 2H), 7.37 (d, *J* = 8.1 Hz, 2H), 6.12 (d, *J* = 7.1 Hz, 1H), 5.91 (dd, *J* = 9.3, 7.1 Hz, 1H), 4.53 (dt, *J* = 9.3, 7.2 Hz, 1H), 2.90 (dd, *J* = 16.7, 7.5 Hz, 1H), 2.80 (dd, *J* = 16.7, 6.8 Hz, 1H), 2.23 (d, *J* = 6.9 Hz, 2H), 1.81 – 1.70 (m, 1H), 1.66 – 1.59 (m, 3H), 1.59 – 1.51 (m, 2H), 1.25 – 1.17 (m, 2H), 1.16 – 1.04 (m, 1H), 0.91 – 0.80 (m, 2H). **^{13}C NMR** (126 MHz, $CDCl_3$) δ 207.7, 146.5, 133.0, 129.2 (q, *J* = 32.8 Hz), 127.9, 125.8 (q, *J* = 3.7 Hz), 119.6, 51.0, 48.6, 38.7, 33.9, 33.3, 33.3, 26.3, 26.2, 26.2. *Quaternary C heterocoupled with F could not be seen. **^{19}F NMR** (471 MHz, $CDCl_3$) δ -62.50. **HRMS (APCI)** Calc. For $C_{19}H_{23}ClF_3O$ [M+H$^+$] 359.1384, found 359.1386. **Optical rotation:** $[\alpha]_D^{21}$ + 68.0 (c=1.04, $CHCl_3$).

Enantiomeric purity was determined by chiral SFC analysis [Lux Cellulose-1, 100 bar, T_{oven}: 40 °C, Flow: 2 mL/min; 1% MeOH, λ =220 nm, major enantiomer t_R = 17.58 min, minor enantiomer t_R = 19.15 min].

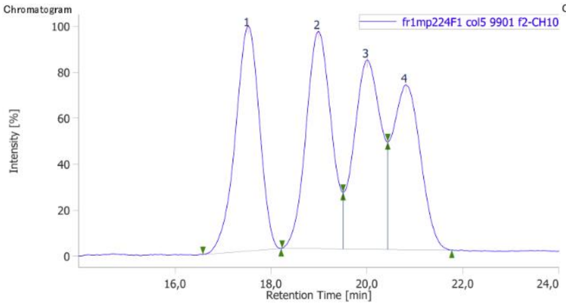

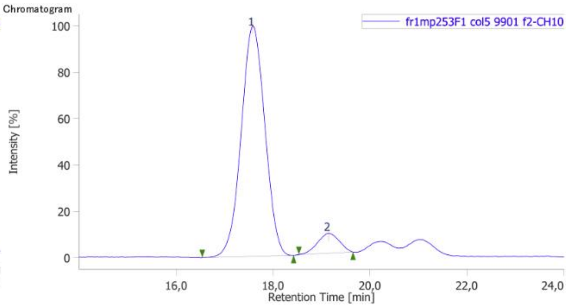

Peak Information

#	Peak Name	CH	tR [min]	Area [μV·sec]	Area%
1	Unknown	10	17,527	3196718	26,227
2	Unknown	10	18,990	3247836	26,647
3	Unknown	10	20,003	3037941	24,925
4	Unknown	10	20,810	2706011	22,201

Peak Information

#	Peak Name	CH	tR [min]	Area [μV·sec]	Area%
1	Unknown	10	17,580	4965592	92,730
2	Unknown	10	19,150	389318	7,270

Note: Chromatogram of racemic compound shows peaks for *Z,Z*-isomer (17.53 and 18.99 min) and *Z,E*-isomer (20.00 and 20.81 min).

(*S*,*Z*)-6-Chloro-4-(3-chlorophenyl)-1-cyclohexylhex-5-en-2-one (28)

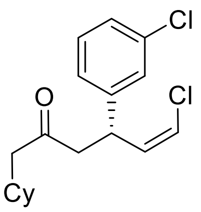

Synthesized from **16** according to general procedure B. Yellow oil obtained in 80% yield with 98:2 er after column chromatography (hexane:CH_2Cl_2, 80:20 to 70:30). This compound was isolated together with 10% of the dechlorinated product. **^{1}H NMR** (500 MHz $CDCl_3$) δ 7.23 – 7.21 (m, 2H), 7.20 – 7.17 (m, 1H), 7.14 (dd, *J* = 7.4, 1.6 Hz, 1H), 6.10 (d, *J* = 7.1 Hz, 1H), 5.87 (dd, *J* = 9.4, 7.1 Hz, 1H), 4.44 (dt, *J* = 9.3, 7.3 Hz, 1H), 2.87 (dd, *J* = 16.5, 7.3 Hz, 1H), 2.76 (dd, *J* = 16.6, 7.1 Hz, 1H), 2.23 (d, *J* = 6.9 Hz, 2H), 1.77 (m, 1H), 1.67 – 1.59 (m, 4H), 1.28 – 1.17 (m, 3H), 1.11 (m, 1H), 0.86 (m, 2H). **^{13}C NMR** (126 MHz, $CDCl_3$) δ 207.8, 144.4, 134.6, 133.2, 130.1, 127.6, 127.1, 125.9, 119.3, 51.0, 48.7, 38.6, 33.9, 33.3, 33.3, 26.3, 26.2. **HRMS (APCI)** Calc. For $C_{18}H_{23}ClO_2$ [M+H$^+$] 325.1120, found 325.1125. **Optical rotation:** $[\alpha]_D^{21}$ + 91.9 (c=1.24, $CHCl_3$).

Enantiomeric purity was determined by chiral uHPLC analysis [Lux i-amilose-3, T_{oven}: 40 °C, Flow: 0.7 mL/min; 99% hexane, λ =209.5 nm, minor enantiomer t_R = 10.81 min, major enantiomer t_R = 11.55 min].

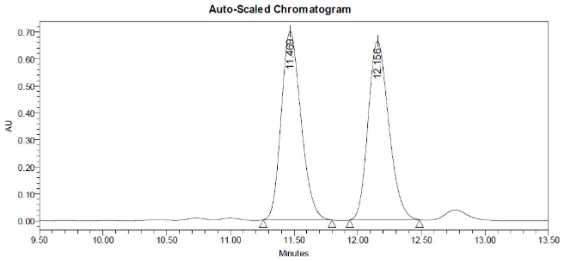

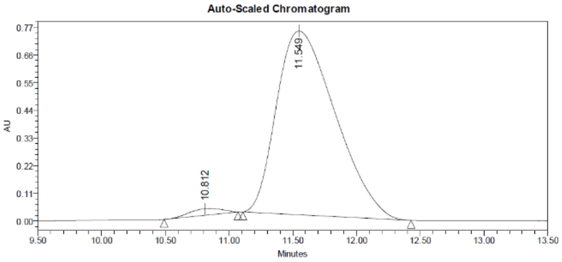

Unknown Peak Results

	RT	Area	% Area	Height
1	11.469	7525883	50.19	698587
2	12.156	7470011	49.81	662707

Unknown Peak Results

	RT	Area	% Area	Height
1	10.812	479993	2.00	26717
2	11.549	23480086	98.00	730648

(*S*,*Z*)-9-(Benzyloxy)-1-chloro-3-(3-chlorophenyl)non-1-en-5-one (29)

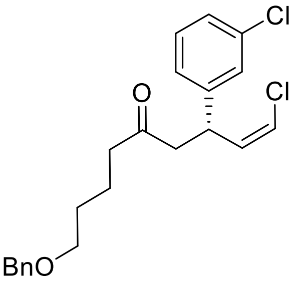

Synthesized from **17** according to general procedure B. Yellow oil obtained in 86% yield with 93:7 er after column chromatography (hexane:AcOEt, 99:1 to 94:6). **^{1}H NMR** (300 MHz, $CDCl_3$) δ 7.38 – 7.32 (m, 4H), 7.30 – 7.20 (m,4H), 7.19 – 7.10 (m, 1H), 6.11 (d, *J* = 7.2 Hz, 1H), 5.88 (dd, *J* = 9.5, 7.0 Hz, 1H), 4.50 (s, 2H), 4.51 – 4.39 (m, 1H), 3.46 (t, *J* = 5.0 Hz, 2H), 2.89 (dd, *J* = 16.9, 7.7 Hz, 1H), 2.78 (dd, *J* = 15.9, 6.8 Hz, 1H), 2.41 (t, *J* = 7.1 Hz, 2H), 1.69 – 1.55 (m, 4H). **^{13}C NMR** (75 MHz, $CDCl_3$) δ 207.9, 144.4, 138.7, 134.6, 133.2, 130.1, 128.5, 127.8, 127.7, 127.6, 127.1, 125.8, 119.3, 73.1, 70.1, 48.1, 42.9, 38.64, 29.2, 20.5. **HRMS (APCI)** Calc. For $C_{22}H_{25}Cl_2O_2$ [M+H$^+$] 391.1226, found 391.1230. **Optical rotation:** $[\alpha]_D^{21}$ + 59.9 (c=0.70, $CHCl_3$).

Enantiomeric purity was determined by chiral uHPLC analysis [CHIRALPAK IB N-3, T_{oven}: 40 °C, Flow: 1 mL/min; 99% hexane, λ =209.5 nm, major enantiomer t_R = 9.61 min, minor enantiomer t_R = 10.67 min].

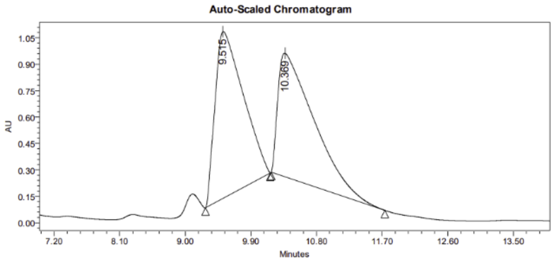

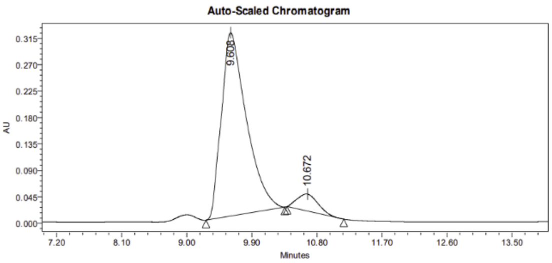

Unknown Peak Results

	RT	Area	% Area	Height
1	9.515	25228062	50.97	946681
2	10.369	24266120	49.03	701082

Unknown Peak Results

	RT	Area	% Area	Height
1	9.608	7515328	92.87	312355
2	10.672	576980	7.13	28909

(*S*,*Z*)-4-(2-Bromophenyl)-6-chloro-1-cyclohexylhex-5-en-2-one (30)

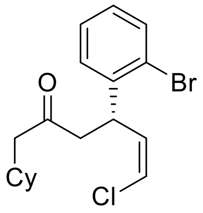

Synthesized from **18** according to general procedure B. Yellow oil obtained in 84% yield with 95.5:4.5 er after column chromatography (hexane:CH_2Cl_2, 80:20 to 70:30). **^{1}H NMR** (500 MHz, $CDCl_3$) δ 7.54 (d, *J* = 8.3 Hz, 1H), 7.26 – 7.22 (m, 2H), 7.06 (ddd, *J* = 8.7, 6.3, 2.8 Hz, 1H), 6.13 (d, *J* = 7.1 Hz, 1H), 6.01 (dd, *J* = 8.7, 7.1 Hz, 1H), 4.82 – 4.76 (m, 1H), 2.91 (dd, *J* = 16.1, 6.1 Hz, 1H), 2.73 (dd, *J* = 16.1, 8.2 Hz, 1H), 2.26 (d, *J* = 7.3 Hz, 2H), 1.85 – 1.74 (m, 1H), 1.67 – 1.57 (m, 5H), 1.27 – 1.17 (m, 2H), 1.15 – 1.05 (m, 1H), 0.92 – 0.81 (m, 2H). **^{13}C NMR** (126 MHz, $CDCl_3$) δ 208.2, 141.5, 133.5, 132.5, 129.0, 128.4, 127.8, 124.1, 120.0, 50.6, 48.3, 38.9, 33.9, 33.3, 26.3, 26.2. **HRMS (APCI)** Calc. For $C_{18}H_{23}BrClO$ [M+H^{+}] 369.0615, found 369.0620. **Optical rotation:** $[\alpha]_{D}^{21}$ + 21.4 (c=1.24, $CHCl_3$).

Enantiomeric purity was determined by chiral uHPLC analysis [Lux i-amilose-3, T_{oven}: 40 °C, Flow: 0.7 mL/min; 99% hexane, λ =209.5 nm, minor enantiomer t_R = 12.63 min, major enantiomer t_R = 13.24 min].

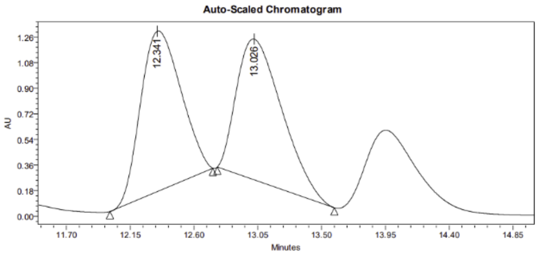

Unknown Peak Results

	RT	Area	% Area	Height
1	12.341	22299998	50.16	1128509
2	13.026	22160824	49.84	990571

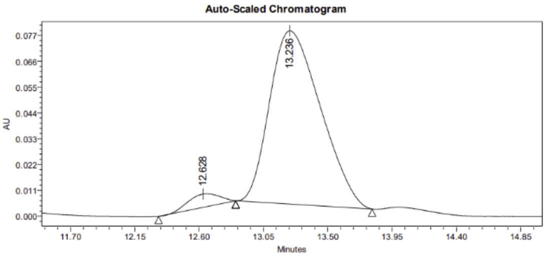

Unknown Peak Results

	RT	Area	% Area	Height
1	12.628	87271	4.55	5710
2	13.236	1829498	95.45	73807

Note: Chromatogram of racemic compound shows peaks for *Z*,*E*-isomer (not separated) and *Z*,*Z*-isomer (12.34 and 13.03 min).

7.3. Transformation of alkenyl chloride 3 into terminal alkyne 31

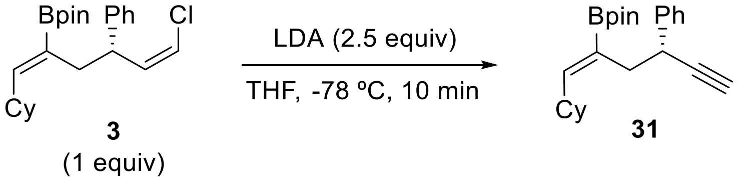

A solution of *n*-BuLi (2.5 M in hexanes) (0.2 mL, 0.45 mmol) was added to a solution of $^{i}Pr_2NH$ (49.5 mg, 0.49 mmol) in THF (0.9 mL) at -78 °C, and the mixture was stirred at -78 °C for 20 min. Then, a solution of **3** (82 mg, 0.18 mmol) in THF (0.9 mL) was added to the resulting solution at -78 °C, and the mixture was stirred at that temperature for 10 min. Then, the reaction was quenched with saturated aqueous solution of NH_4Cl (5 mL), and the mixture was extracted with CH_2Cl_2 (2x5 mL). The combined organic layers were dried over Na_2SO_4, filtered, and concentrated under reduced pressure. Crude product was purified through flash column chromatography

(hexane:CH_2Cl_2, 90:10 to 80:20) affording the desired product as a yellow oil in 44% yield and with 95:5 er.

(*S*,*Z*)-2-(1-Cyclohexyl-4-phenylhex-1-en-5-yn-2-yl)-4,4,5,5-tetramethyl-1,3,2-dioxaborolane (31)

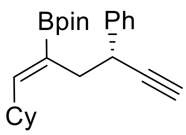

^{1}H NMR (300 MHz, $CDCl_3$) δ 7.39 – 7.34 (m, 1H), 7.32 – 7.25 (m, 3H), 7.23 – 7.16 (m, 1H), 6.14 (d, *J* = 9.8 Hz, 1H), 3.82 (td, *J* = 7.7, 2.5 Hz, 1H), 2.69 (dd, *J* = 12.9, 7.8 Hz, 1H), 2.55 (dd, *J* = 12.9, 7.5 Hz, 1H), 2.25-2.17 (m, 2H), 1.72 – 1.57 (m, 4H), 1.25 (d, *J* = 2.7 Hz, 12H), 1.21 – 1.01 (m, 4H), 1.02 – 0.81 (m, 2H). **^{13}C NMR** (75 MHz, $CDCl_3$) δ 154.0, 141.8, 128.4, 127.9, 126.7, 86.9, 83.3, 70.6, 55.3, 38.2, 37.9, 37.8, 32.7, 32.4, 26.2, 25.9, 25.0, 24.9. **^{11}B NMR** (160 MHz, $CDCl_3$) δ 30.3. **HRMS (APCI)** Calc. For $C_{24}H_{34}BO_2$ [M+H$^+$] 365.2646, found 365.2654. **Optical rotation:** $[\alpha]_D^{21}$ - 9.1 (c=0.47, $CHCl_3$).

Enantiomeric purity was determined by chiral uHPLC analysis [Lux i-amylose-3, T_{oven}: 40 °C, Flow: 0.5 mL/min; 99% hexane, λ =209.5 nm, minor enantiomer t_R = 6.35 min, major enantiomer t_R = 6.68 min].

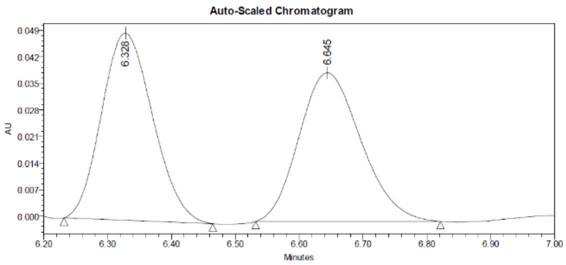

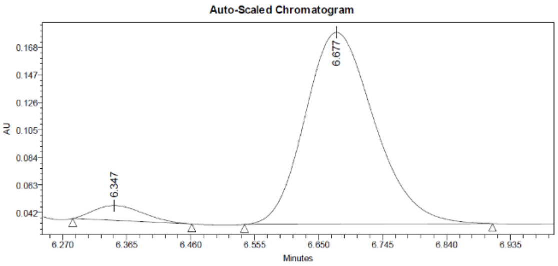

Unknown Peak Results

	RT	Area	% Area	Height
1	6.328	275654	51.03	49346
2	6.645	264533	48.97	39217

Unknown Peak Results

	RT	Area	% Area	Height
1	6.347	57653	5.22	11319
2	6.677	1047676	94.78	146244

8. Chemoselectivity versus other unsaturated hydrocarbons

To evaluate the chemoselectivity of the reaction towards the presence of other unsaturated hydrocarbons, we ran the reaction in the presence of either alkyne or an alkene. The reaction was totally chemoselective in both cases observing the exclusive formation of the allene allylboration product without erosion in yield or selectivity. The competing unsaturated hydrocarbon was recovered unreacted in both cases.

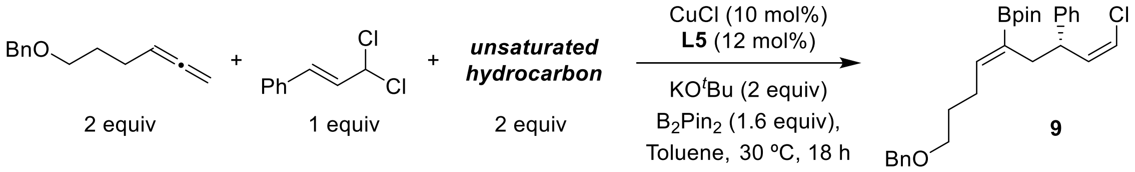

9

Entry[a]	Unsaturated compound	*Z,Z*/*Z,E* ratio[b]	Yield [%][c]	9 er[d]
**1**	-	>20:1	50	95.5:4.5
**2**	Ph—≡—Me	>20:1	53	95.5:4.5
**3**	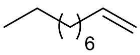	>20:1	51	95.5:4.5

[a] Run on a 0.2 mmol scale under optimized conditions. [b] Determined by ^{1}H-NMR analysis of reaction crude. [c] Yield of isolated product. [d] Determined by uHPLC analysis of the oxidized product.

9. Unsuccessful substrates

Although the transformation showed a broad functional group tolerance as well as the possibility to include different substitution patterns, aliphatic *gem*-dichlorides were poorly reactive and non-enantioselective under standard reaction conditions.
It is important to note that these substrates were used as a 0.8:1 mixture of 1,1 and 1,3-allylic dichlorides. In contrast to our previous reaction with alkynes,[9] the 1,3-isomer is fully consumed in the reaction with allenes. This fact could affect the enantioselectivity of the process since the 1,3-isomer may lead to the formation of different enantiomeric mixtures than the 1,1-isomer.

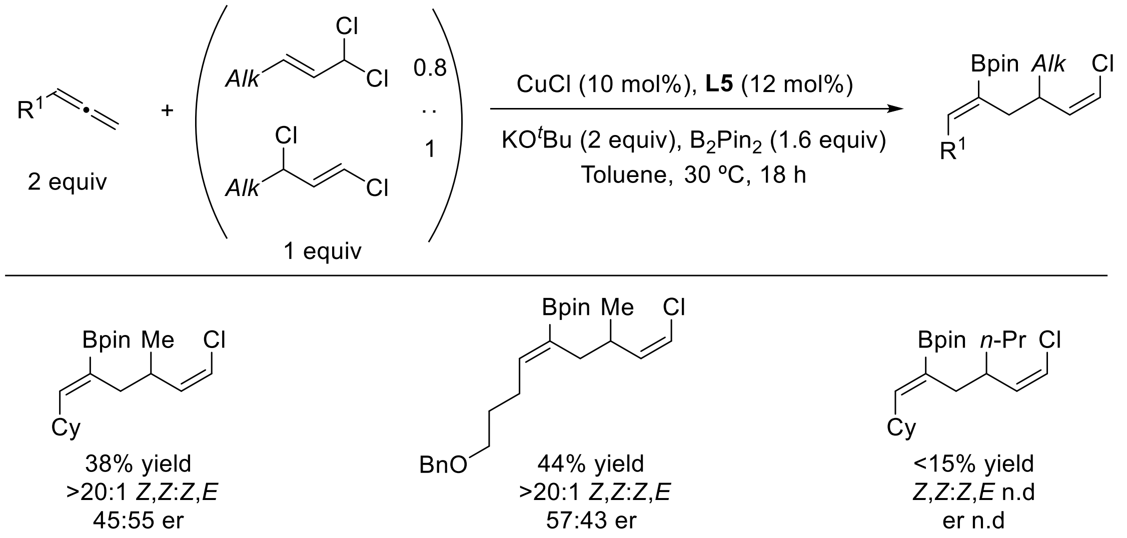

Disubstituted allylic dichlorides such as (3,3-dichloro-2-methylprop-1-en-1-yl)benzene and (4,4-dichlorobut-2-en-2-yl)benzene did not lead to any tractable amount of product.

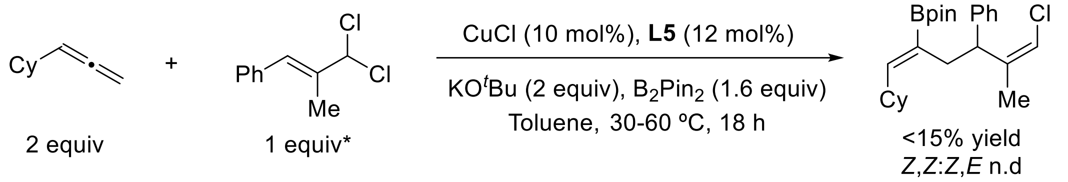

* *used as a 1:5 mixture of 1,1- and 1,3-dichloride*

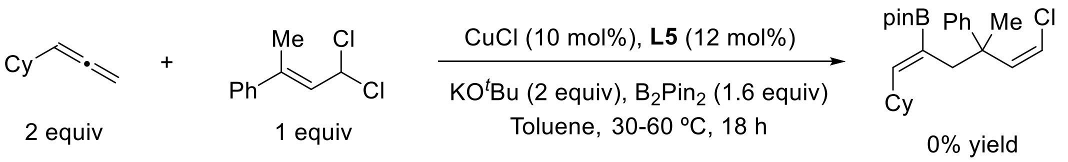

A 1,1-disubstituted allene such as 1,1-dimethylallene was not efficient under standard reaction conditions.

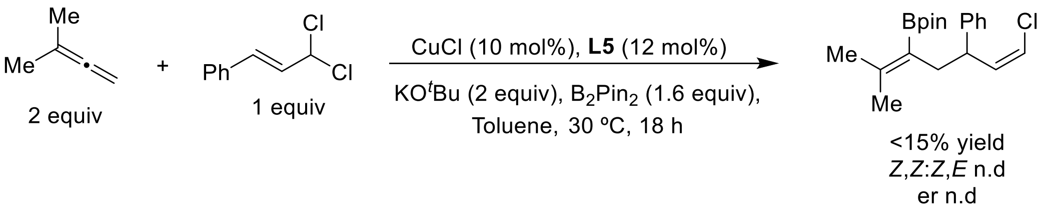

10. ^{1}H-NMR and ^{13}C-NMR spectra

1H NMR (CDCl3, 300 MHz)

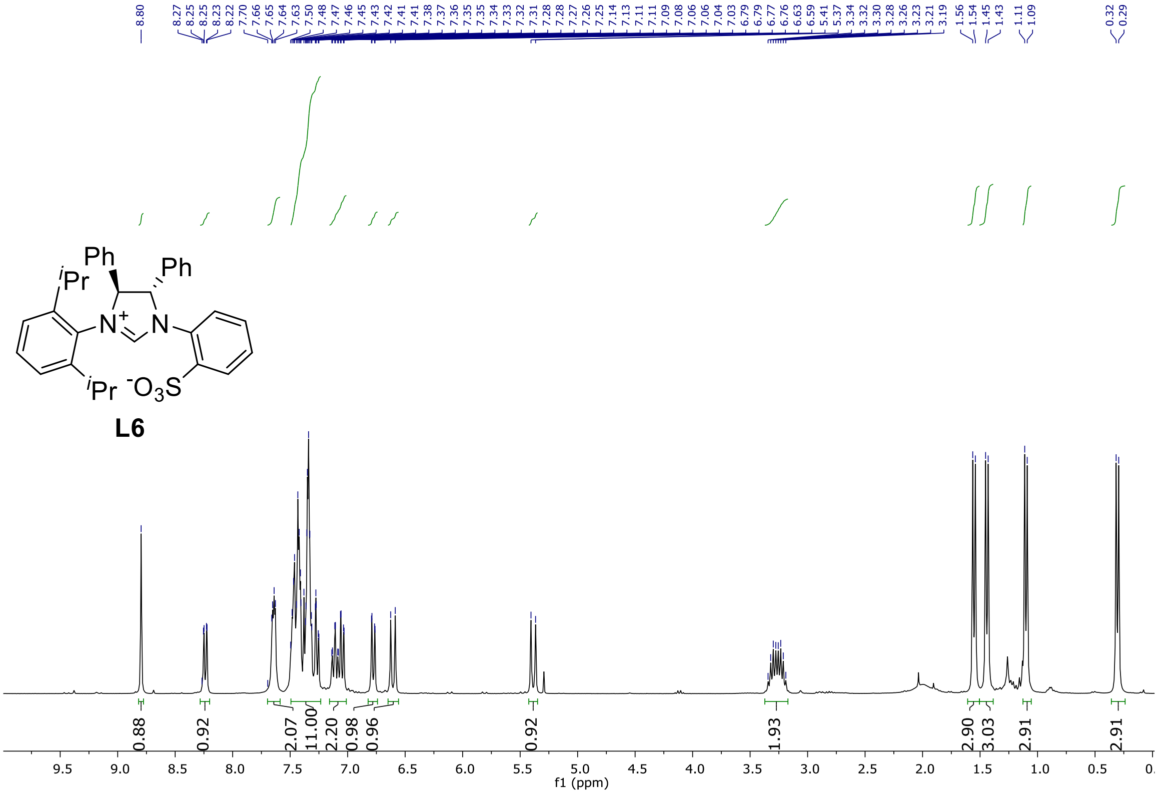

13C NMR (CDCl3, 75 MHz)

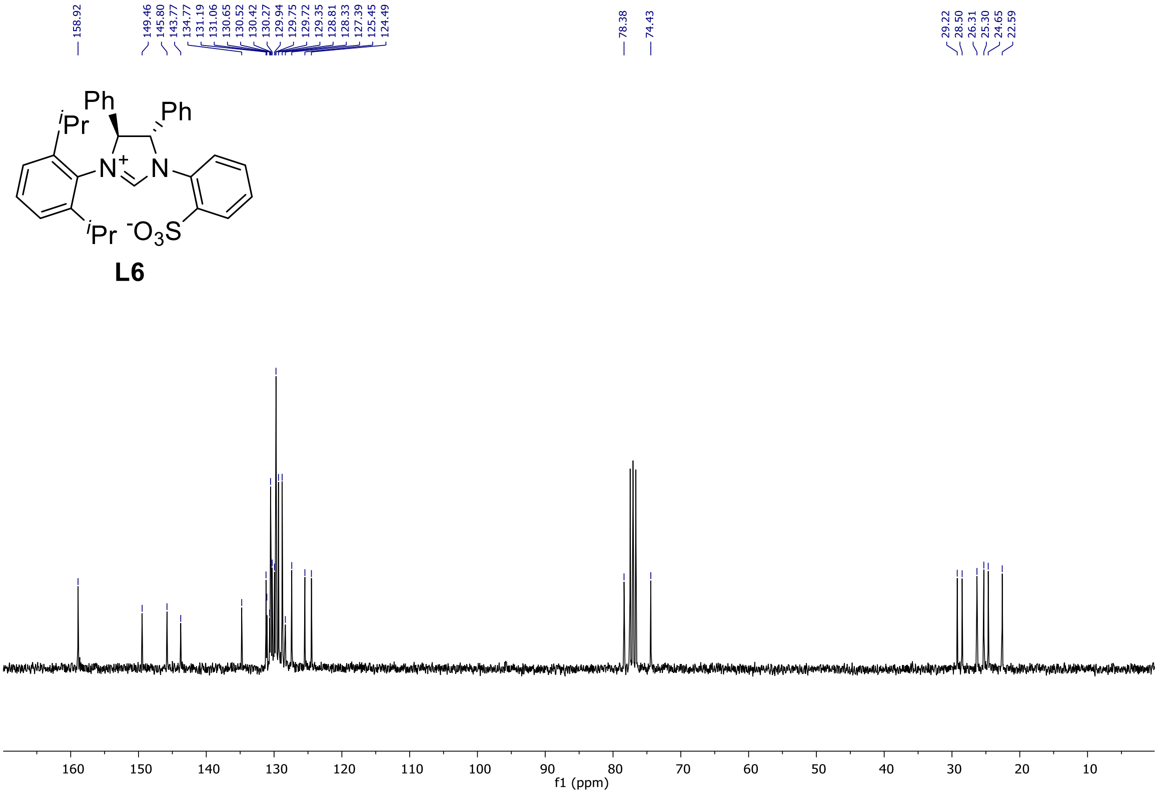

1H NMR (DMSO, 300 MHz)

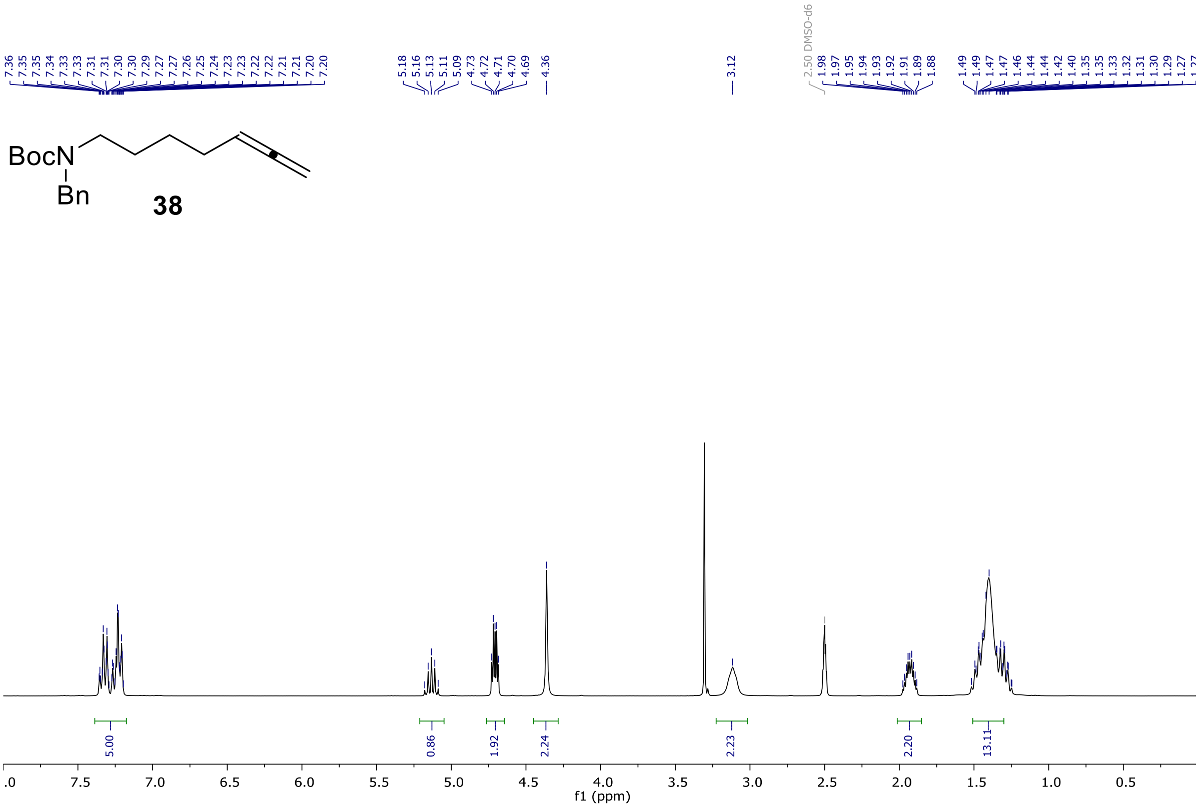

38

13C NMR (DMSO, 75 MHz)

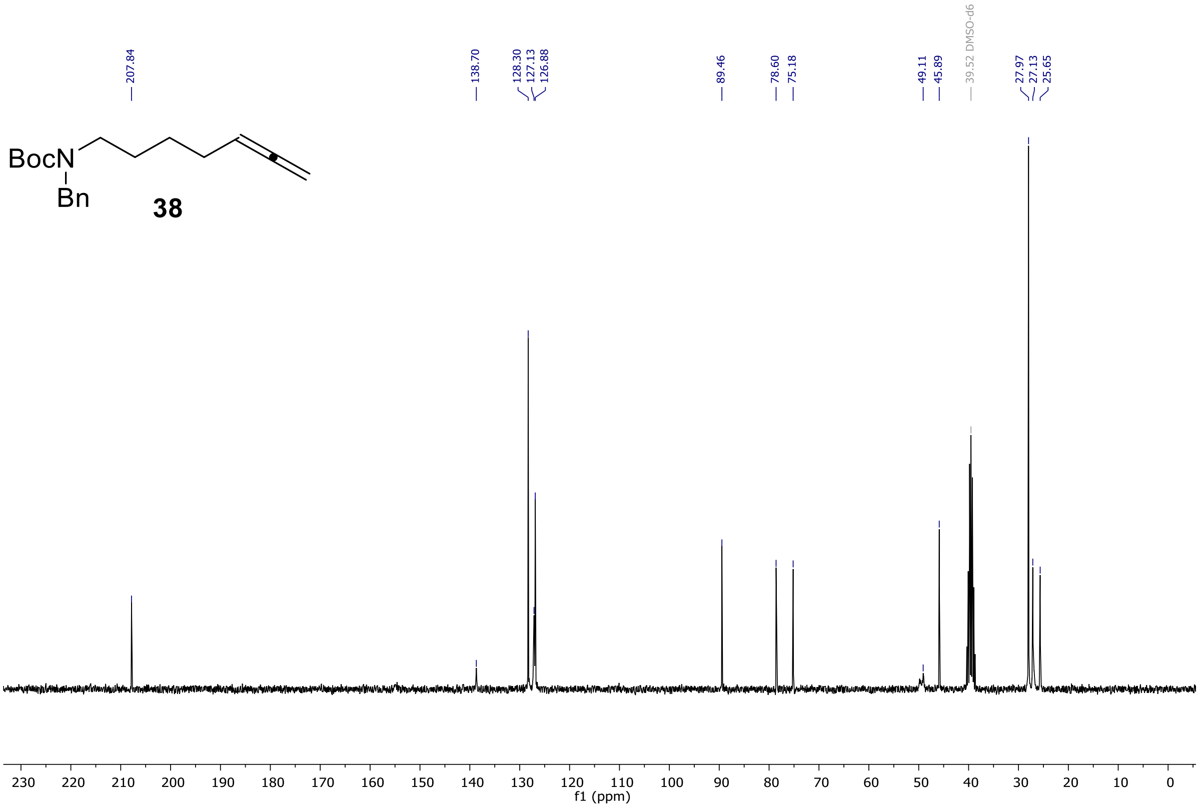

38

1H NMR (CDCl3, 500 MHz)

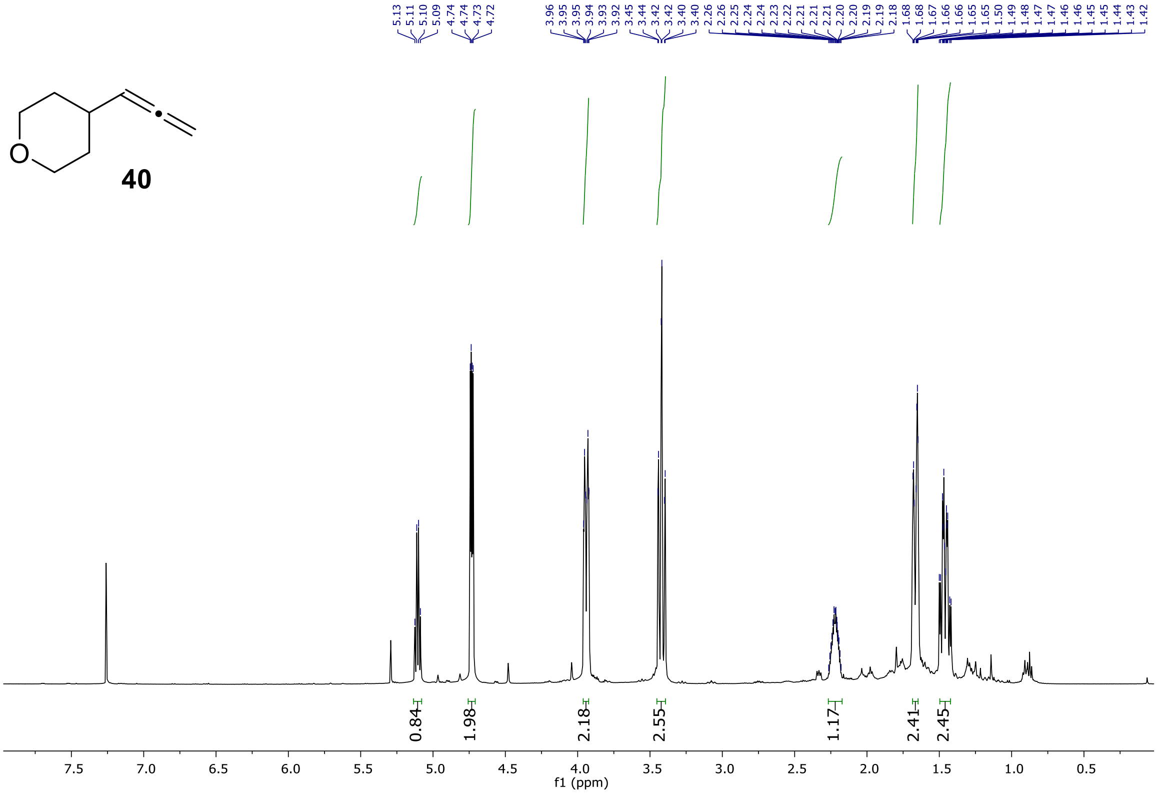

13C NMR (CDCl3, 126 MHz)

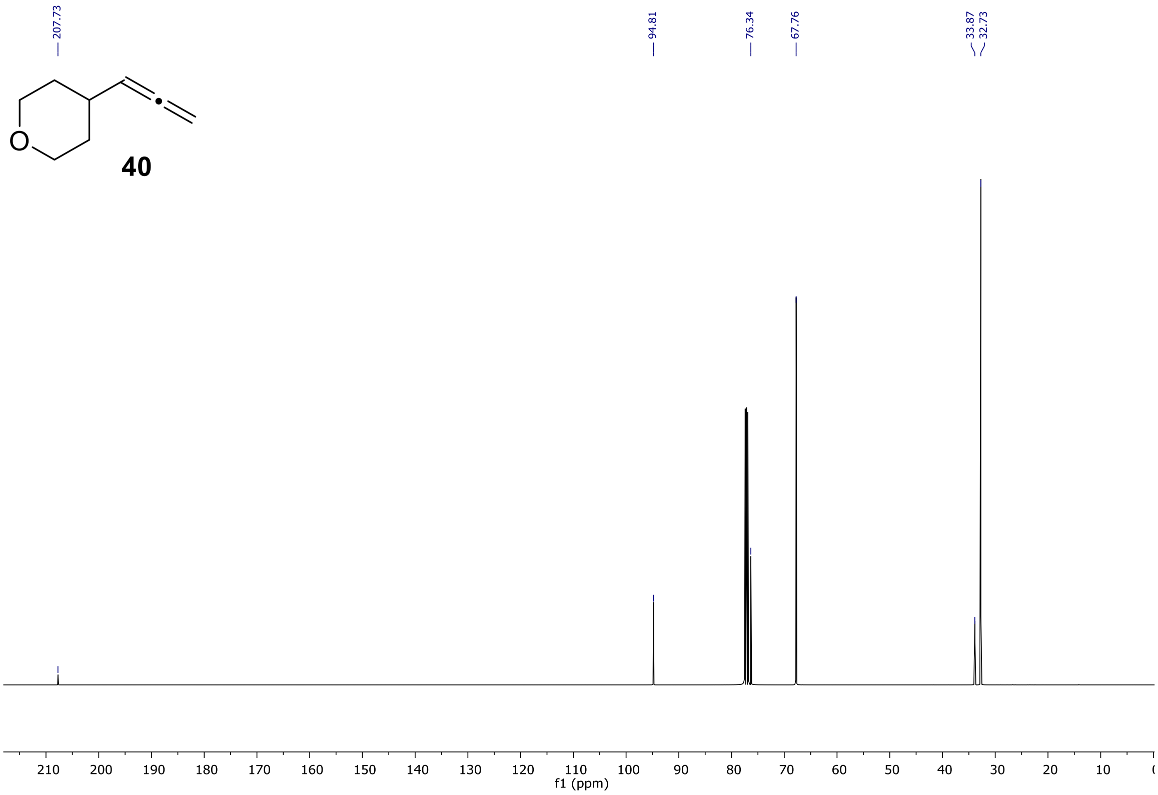

1H NMR (CDCl3, 300 MHz)

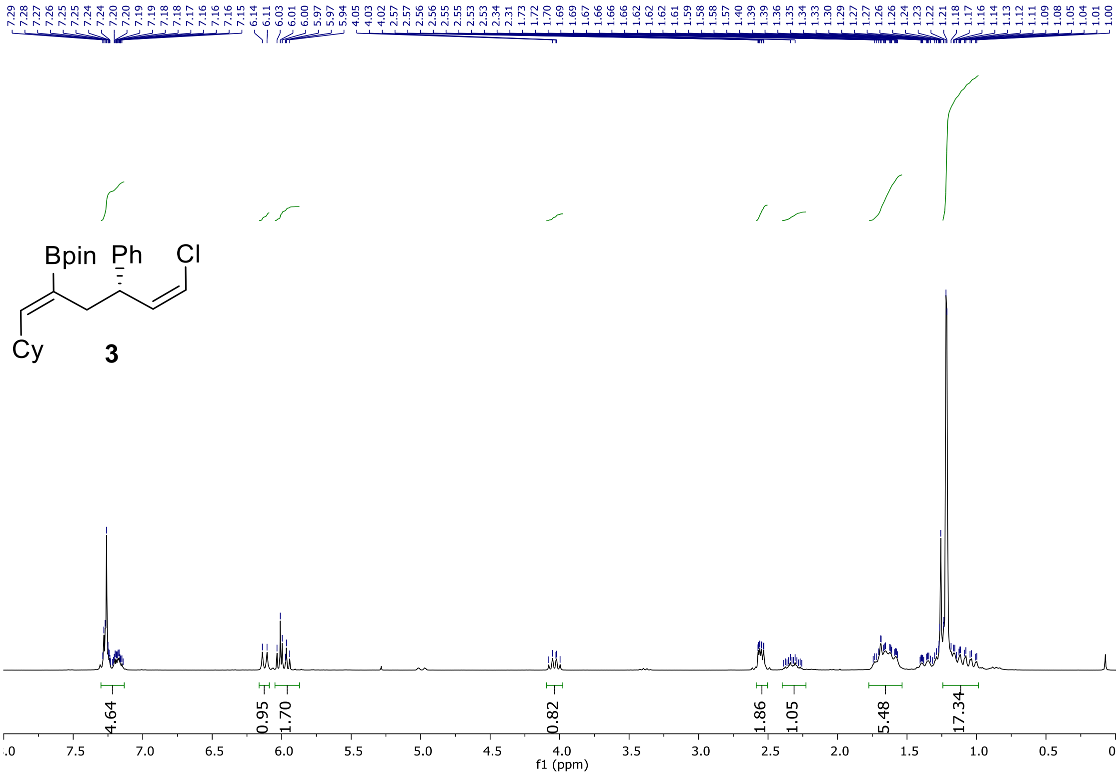

13C NMR (CDCl3, 75 MHz)

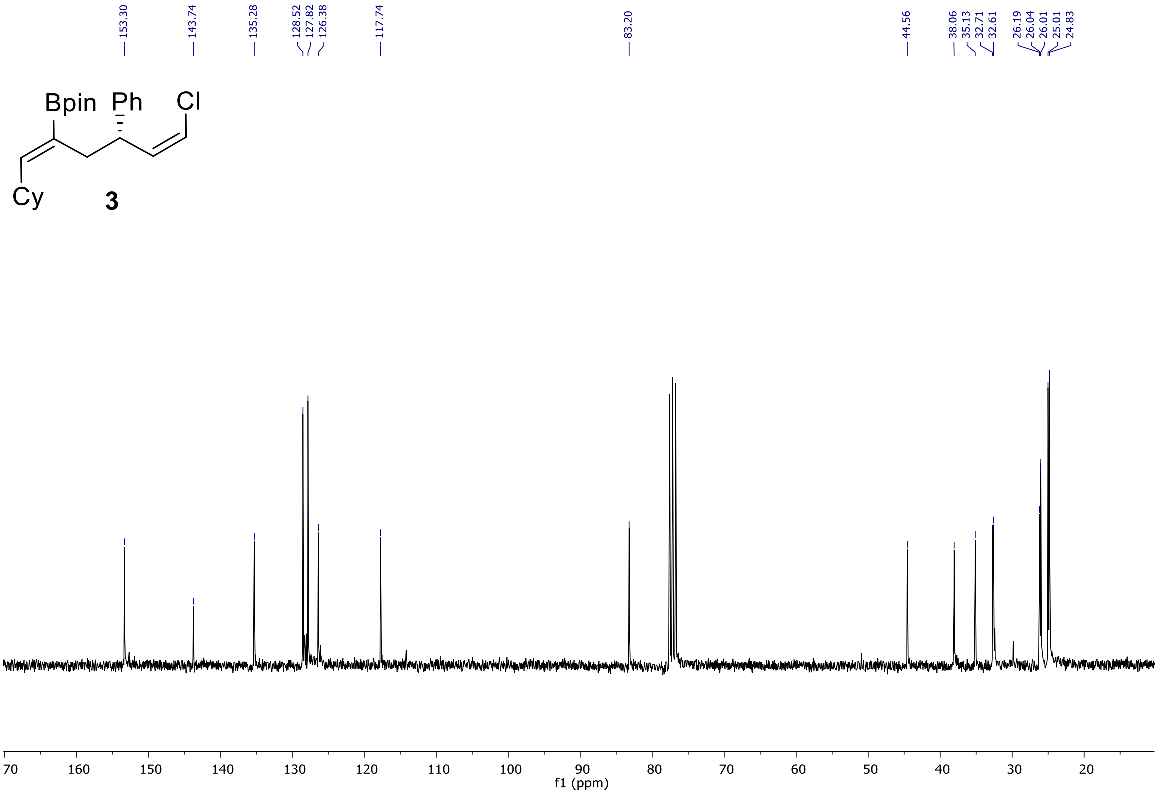

1H NMR (CDCl3, 300 MHz)

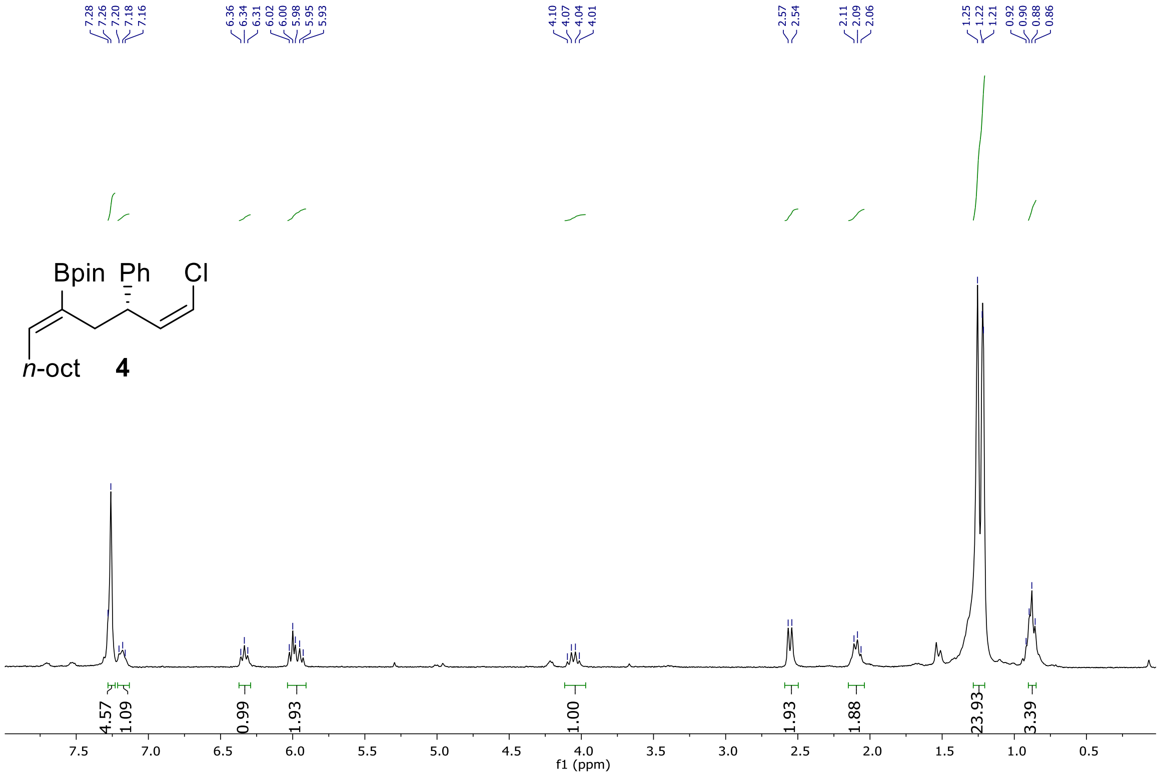

13C NMR (CDCl3, 75 MHz)

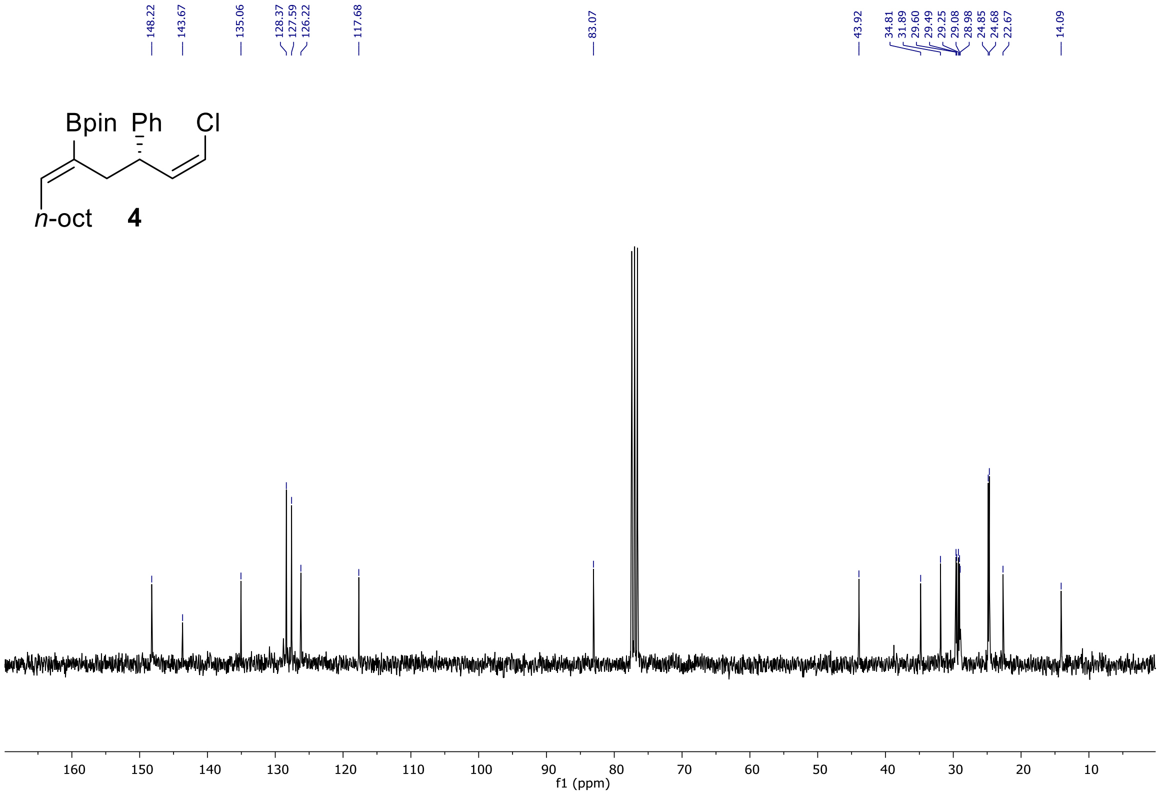

1H NMR (CDCl3, 500 MHz)

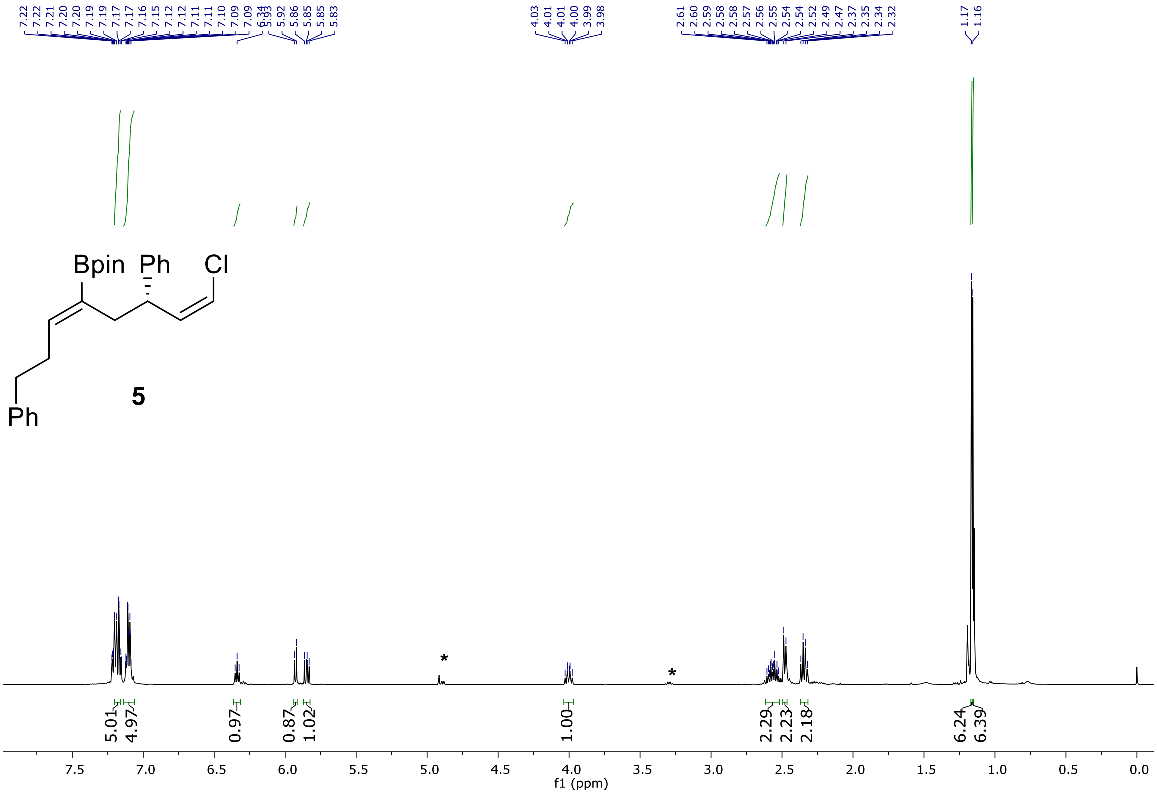

* These signals belong to the dechlorinated product.

13C NMR (CDCl3, 126 MHz)

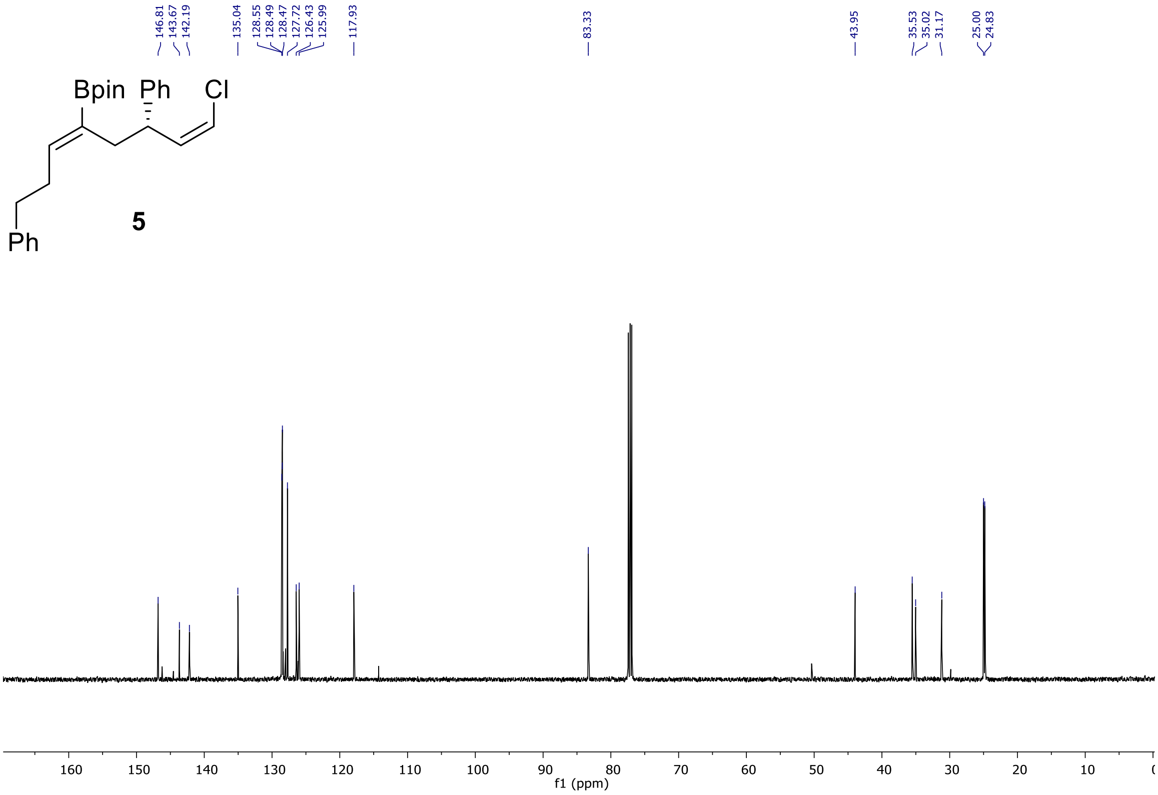

1H NMR (CDCl3, 500 MHz)

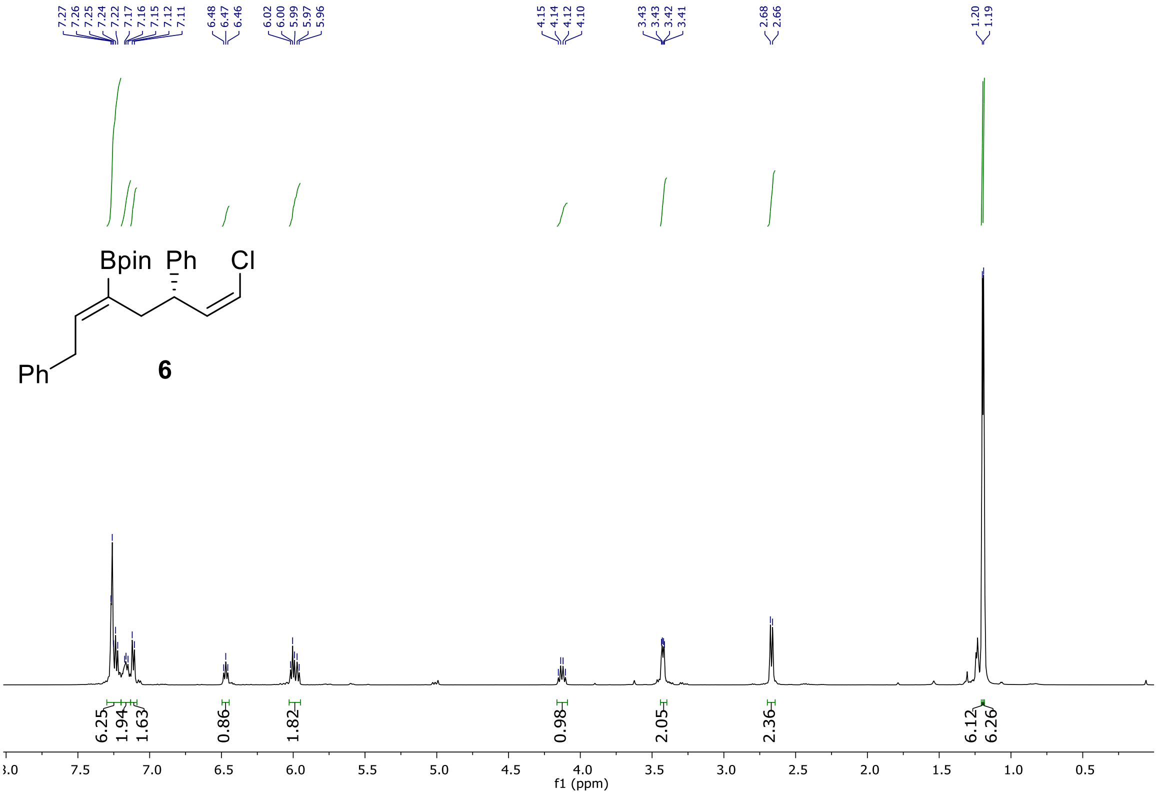

13C NRM (CDCl3, 126 MHz)

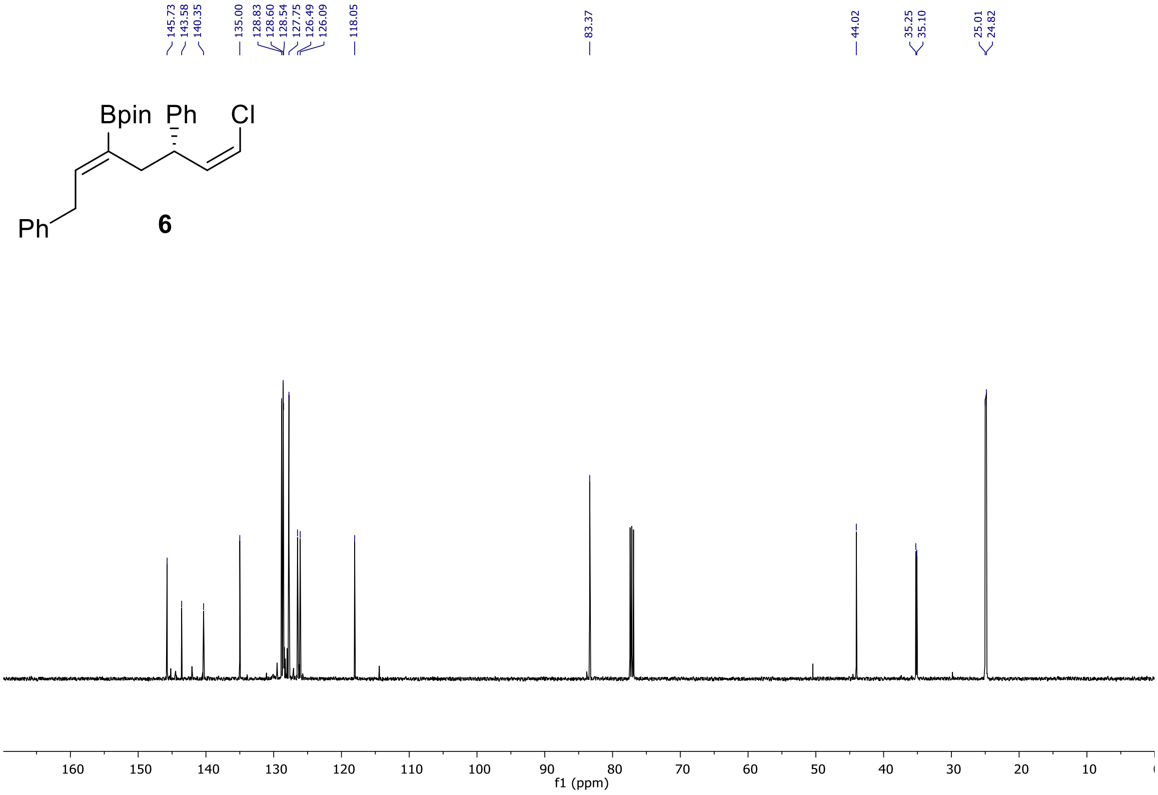

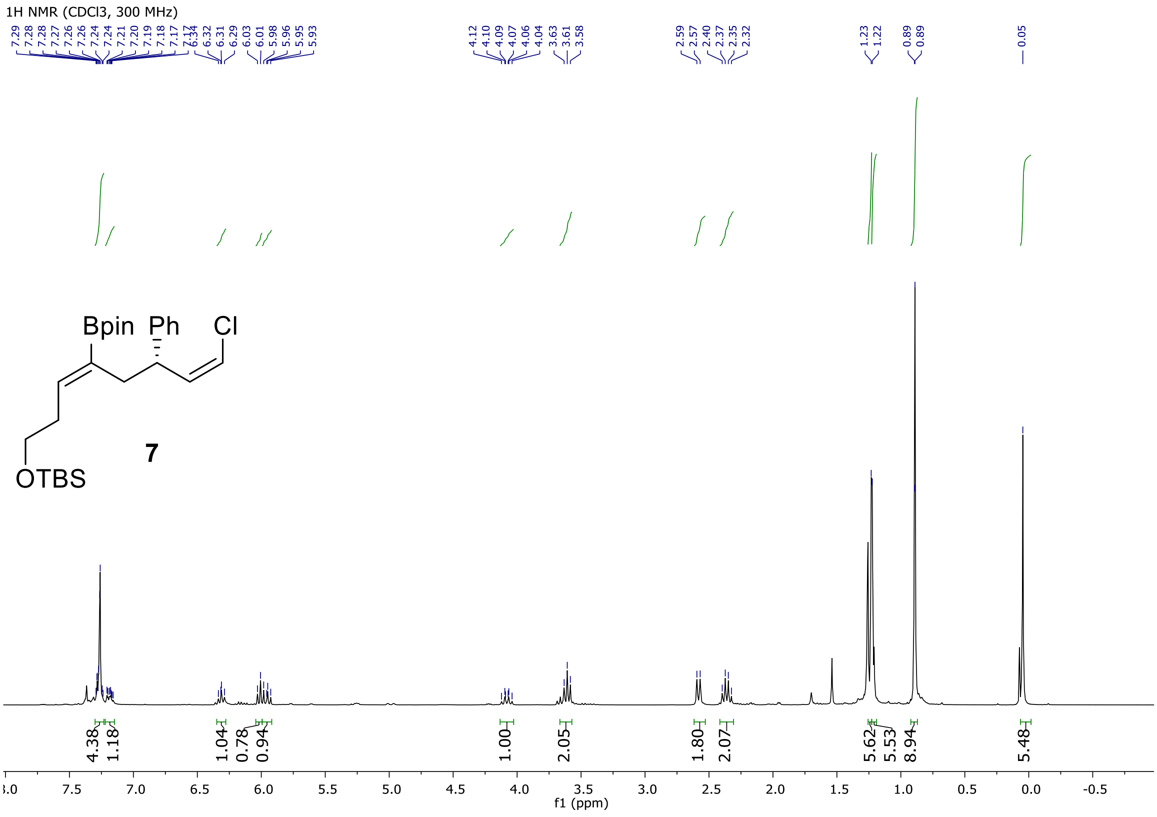
1H NMR (CDCl3, 300 MHz)
7.29 7.28 7.28 7.27 7.26 7.26 7.24 7.24 7.21 7.20 7.19 7.18 7.17 7.17 6.34 6.32 6.31 6.29 6.03 6.01 5.98 5.96 5.95 5.93
4.12 4.10 4.09 4.07 4.06 4.04 3.63 3.61 3.58
2.59 2.57 2.40 2.37 2.35 2.32
1.23 1.22 0.89 0.89
0.05
Bpin Ph Cl
7
OTBS
4.38 1.18 1.04 0.78 0.94 1.00 2.05 1.80 2.07 5.62 5.53 8.94 5.48
f1 (ppm)

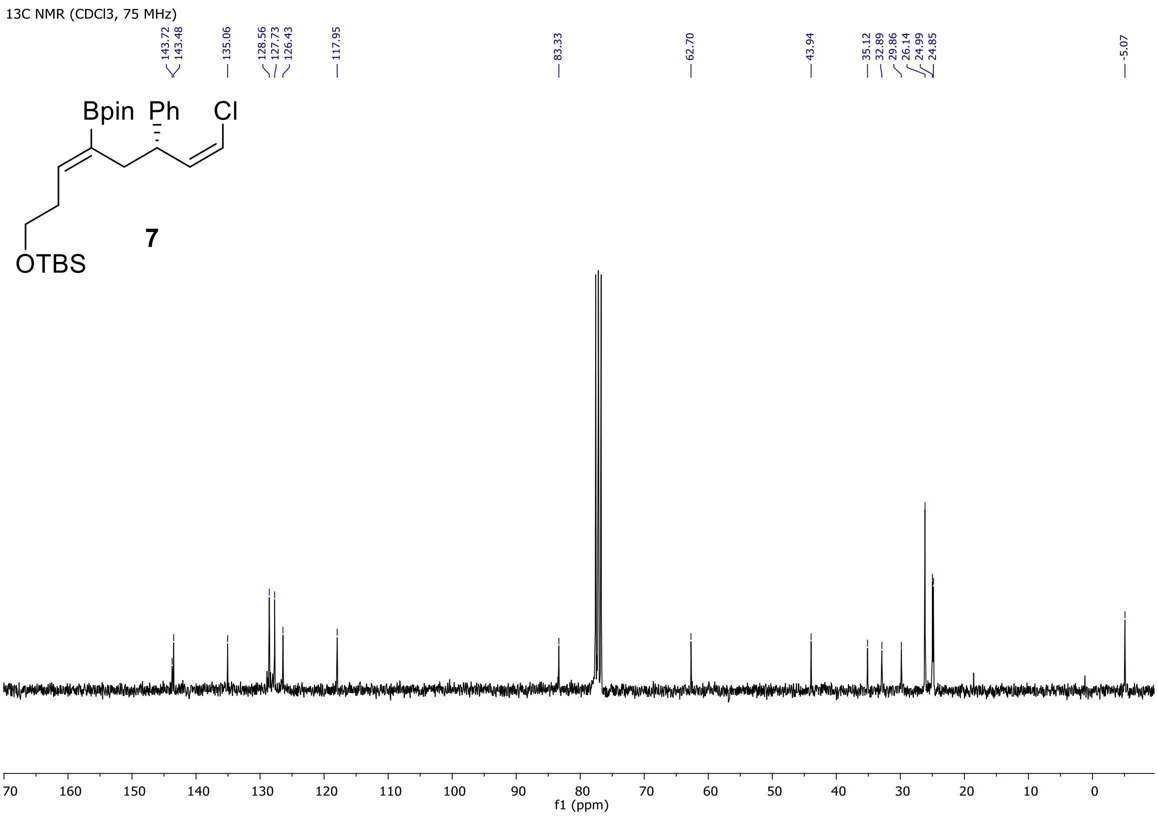
13C NMR (CDCl3, 75 MHz)
143.72 143.48 135.06 128.56 127.73 126.43 117.95 83.33 62.70 43.94 35.12 32.89 29.86 26.14 24.99 24.85 -5.07
Bpin Ph Cl
7
OTBS
f1 (ppm)

1H NMR (CDCl3, 500 MHz)

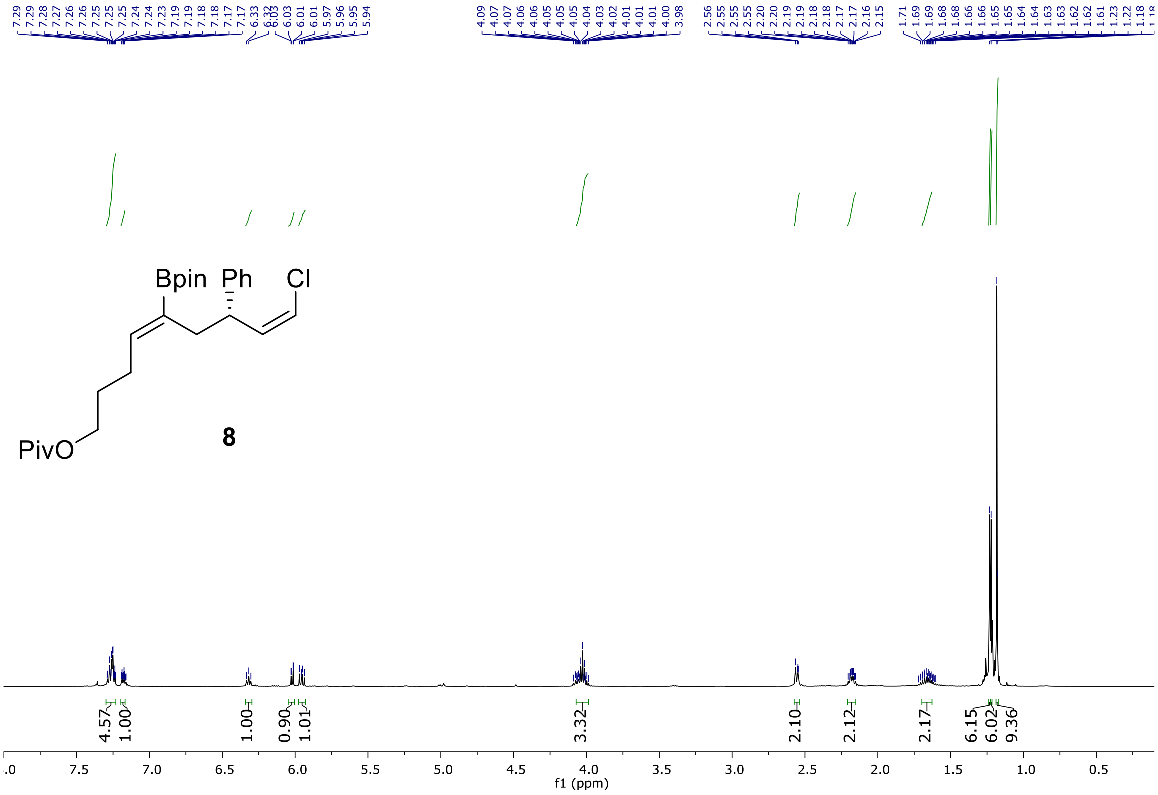

13C NMR (CDCl3, 126 MHz)

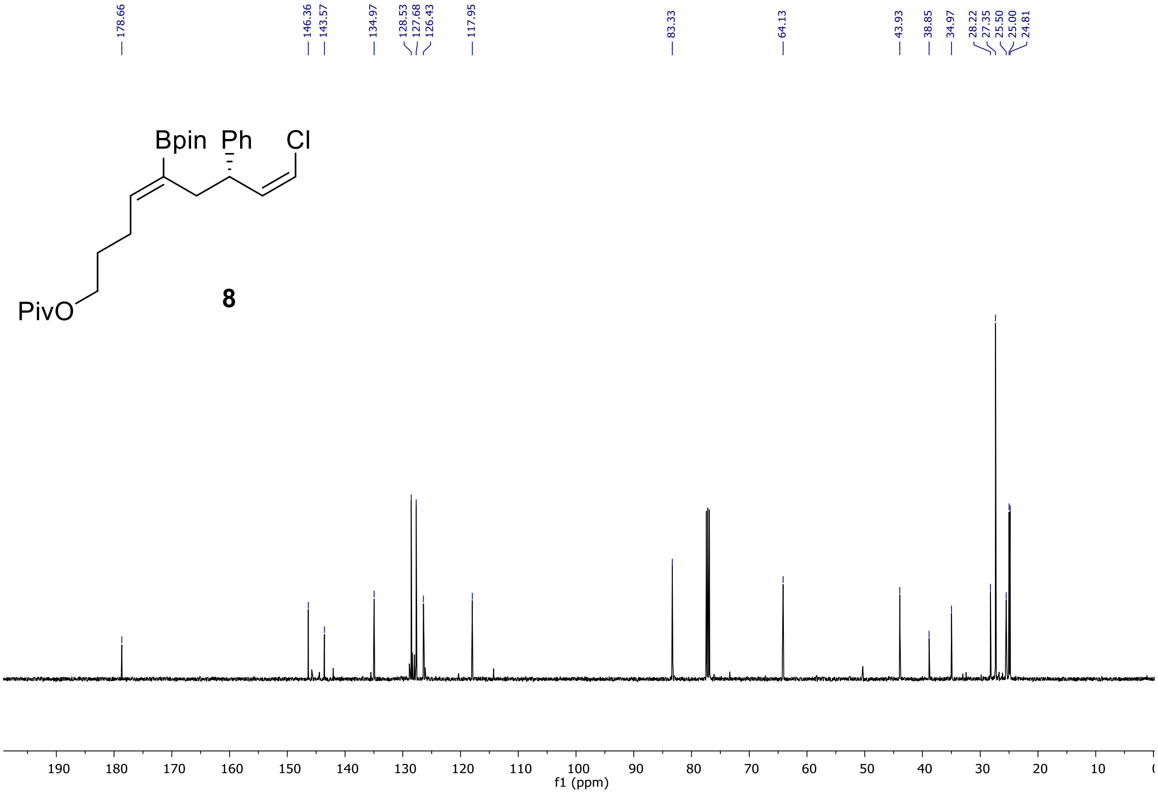

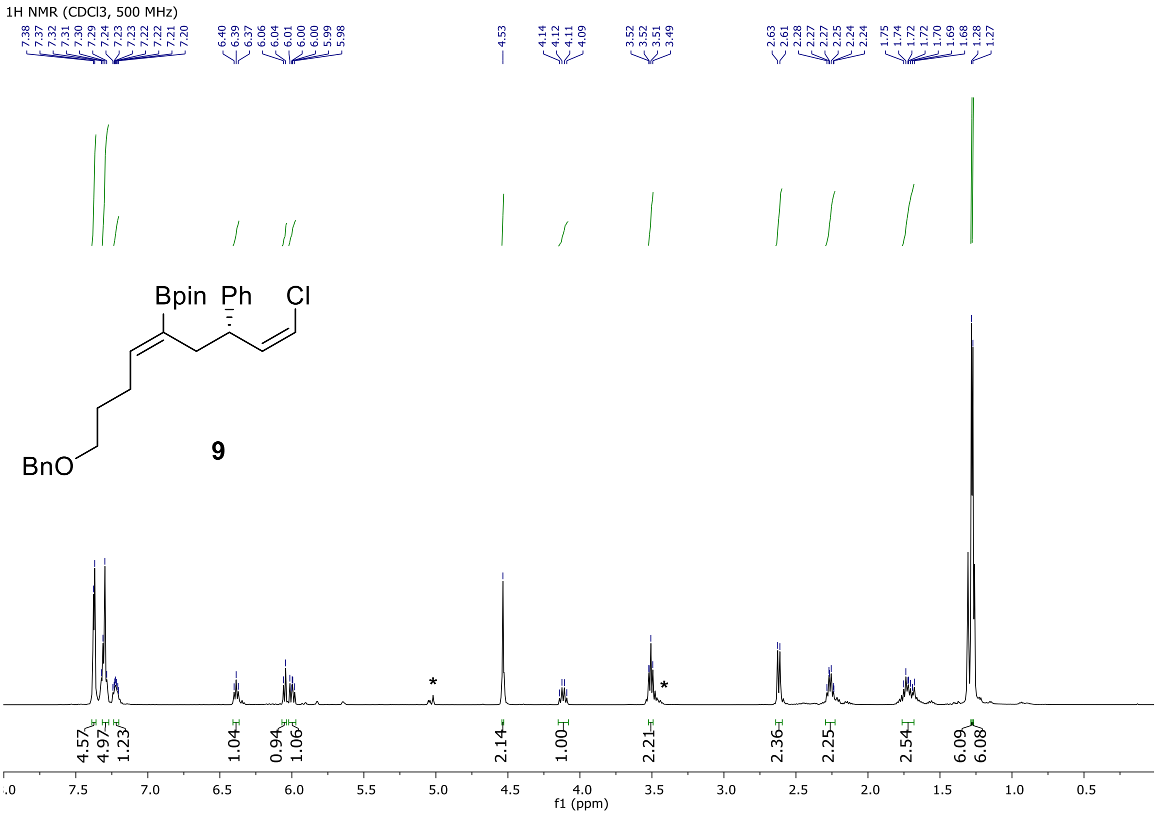

* These signals belong to the dechlorinated product.

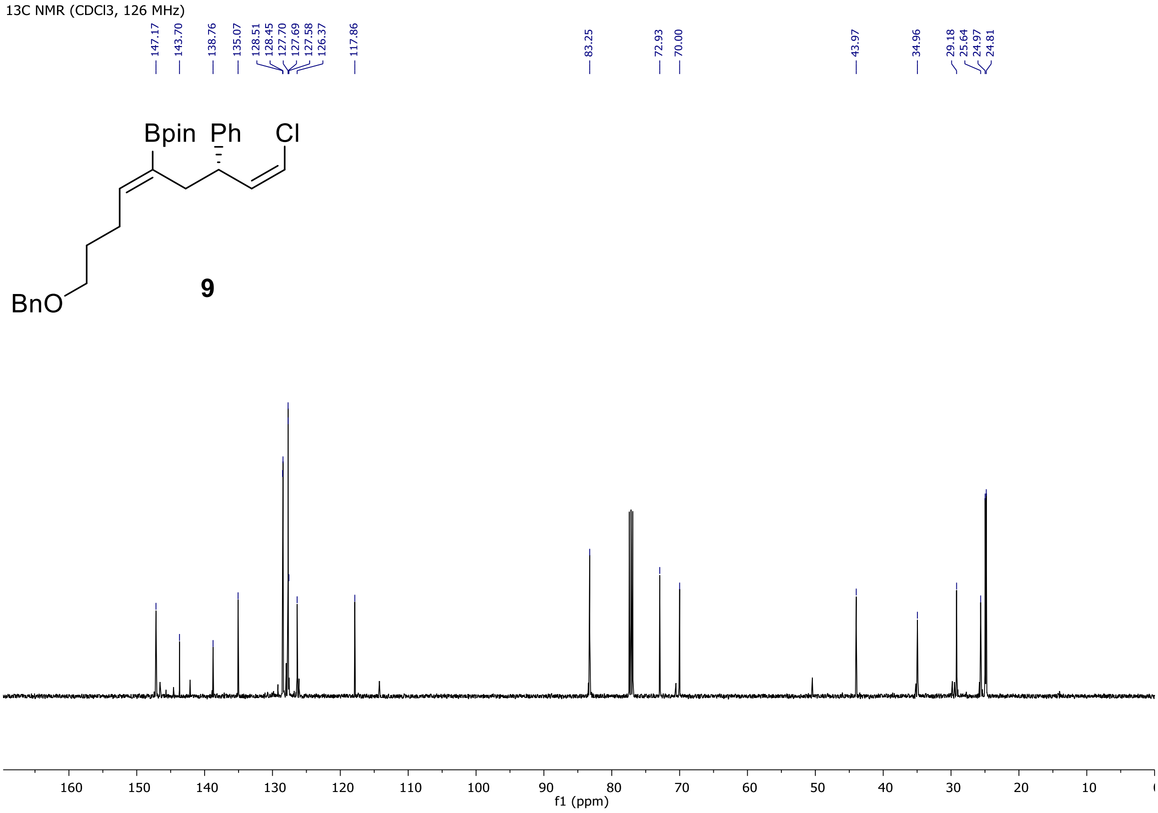

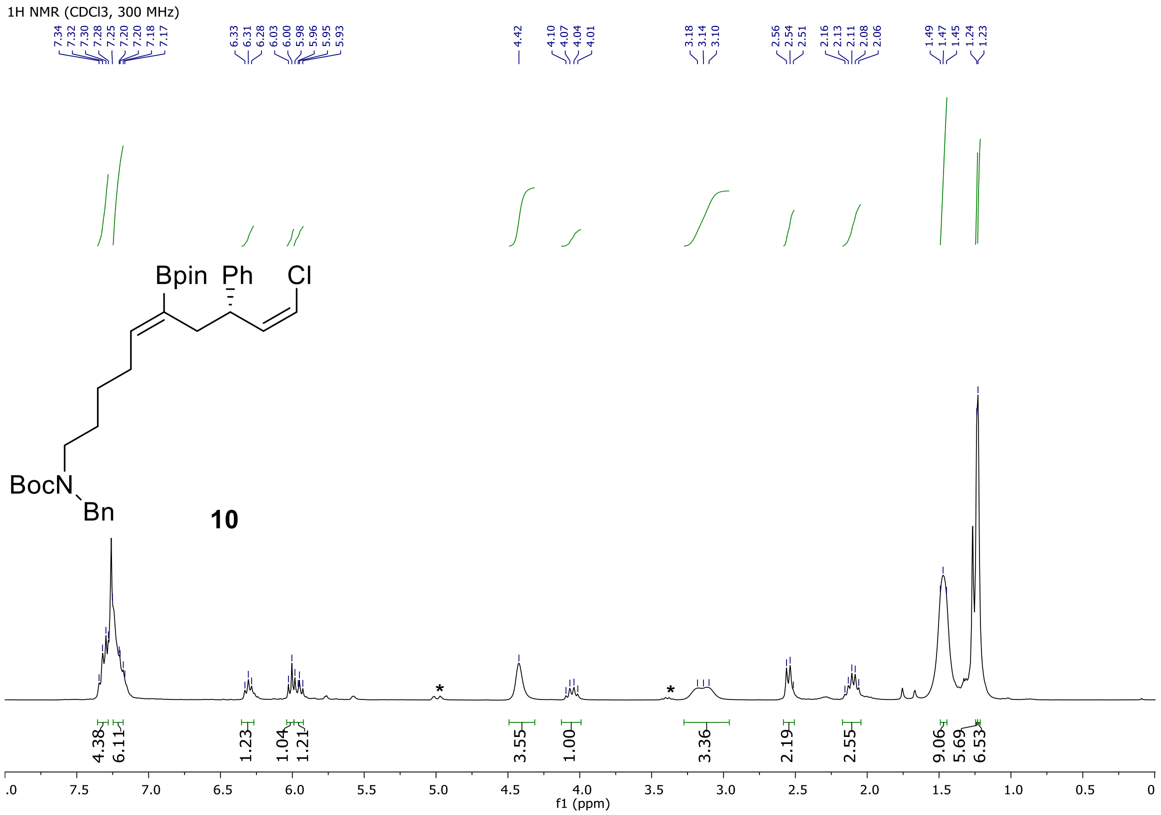

* Characteristic signals of the dechlorinated product.

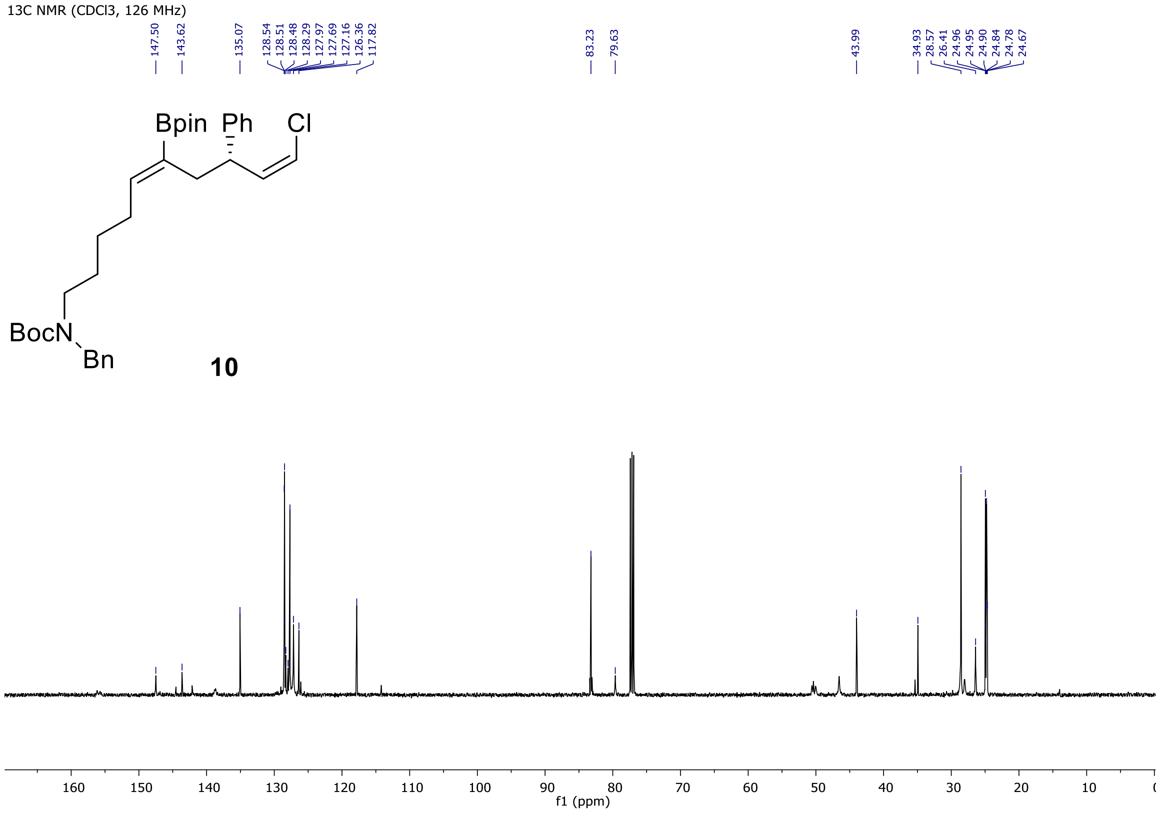

1H NMR (CDCl3, 500 MHz)

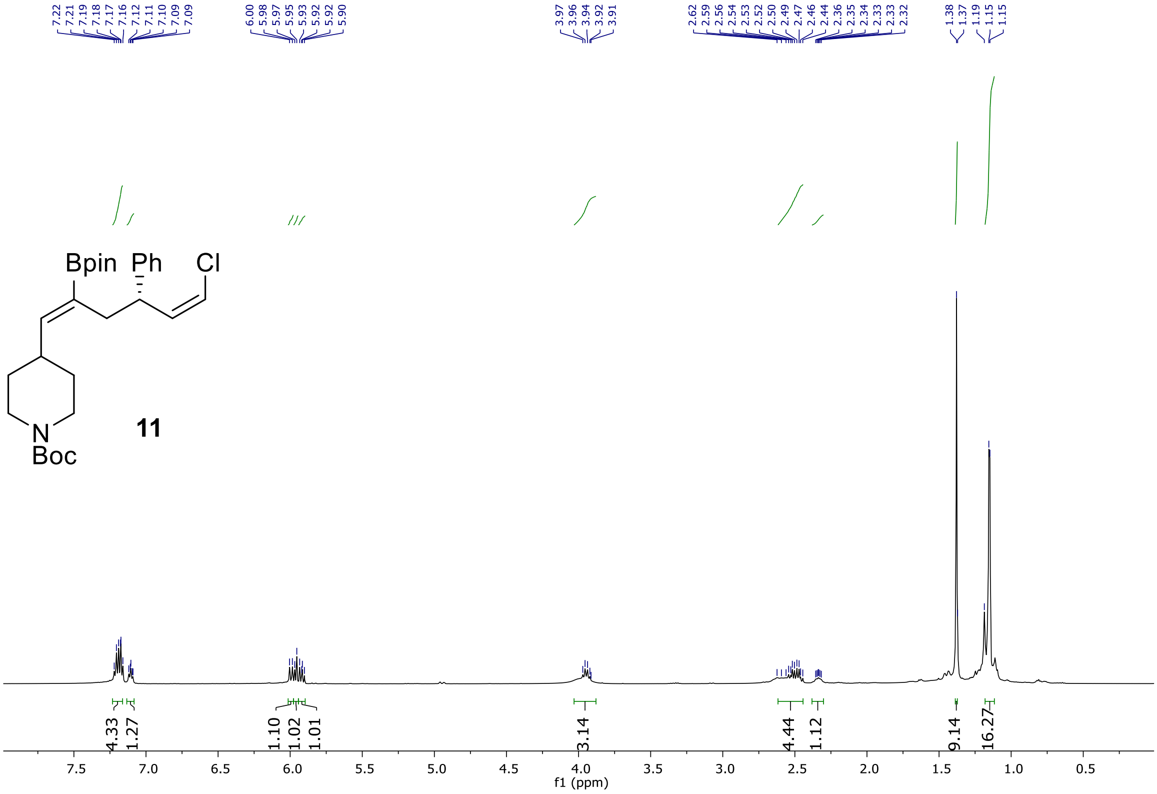

13C NMR (CDCl3, 126 MHz)

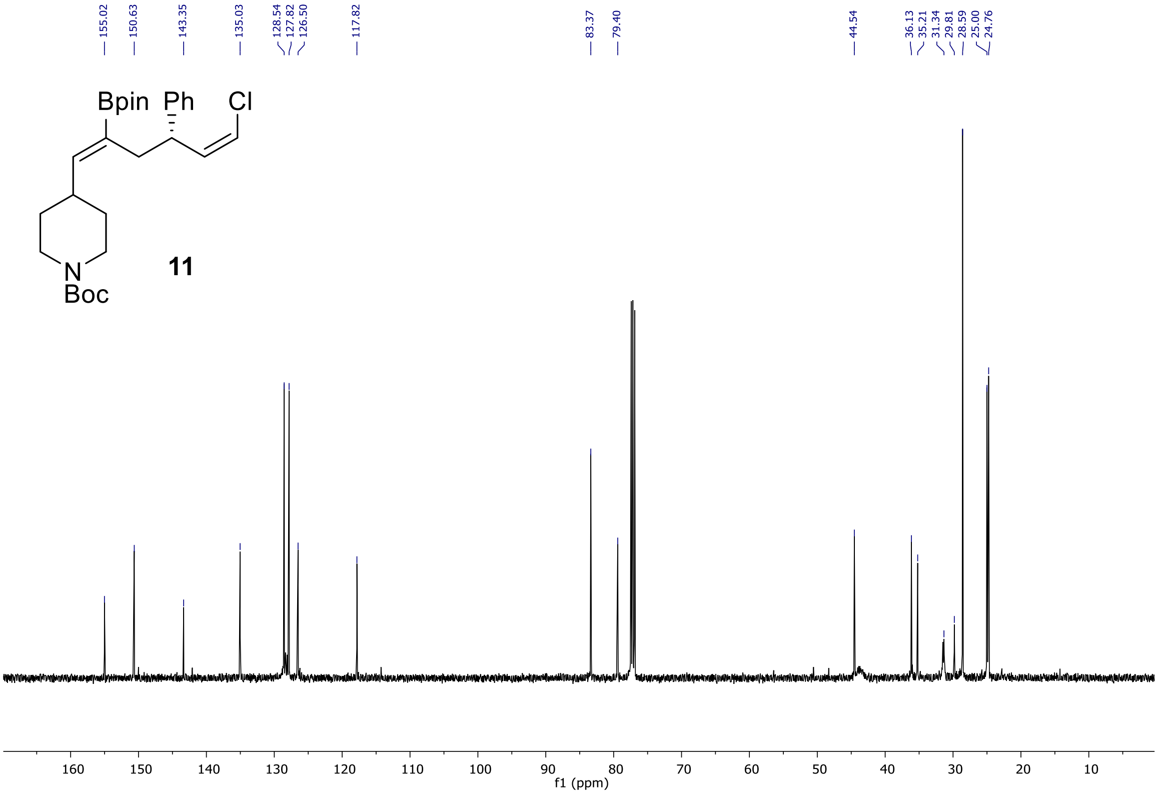

1H NMR (CDCl3, 500 MHz)

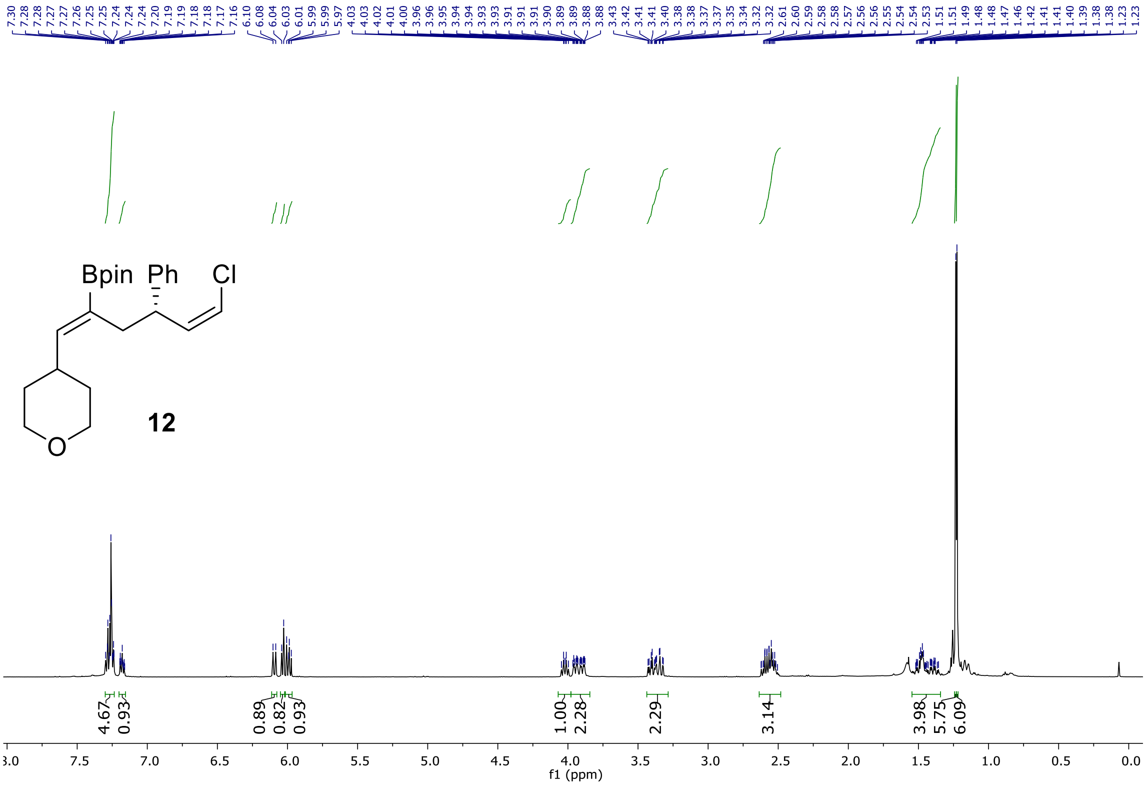

13C NMR (CDCl3, 126 MHz)

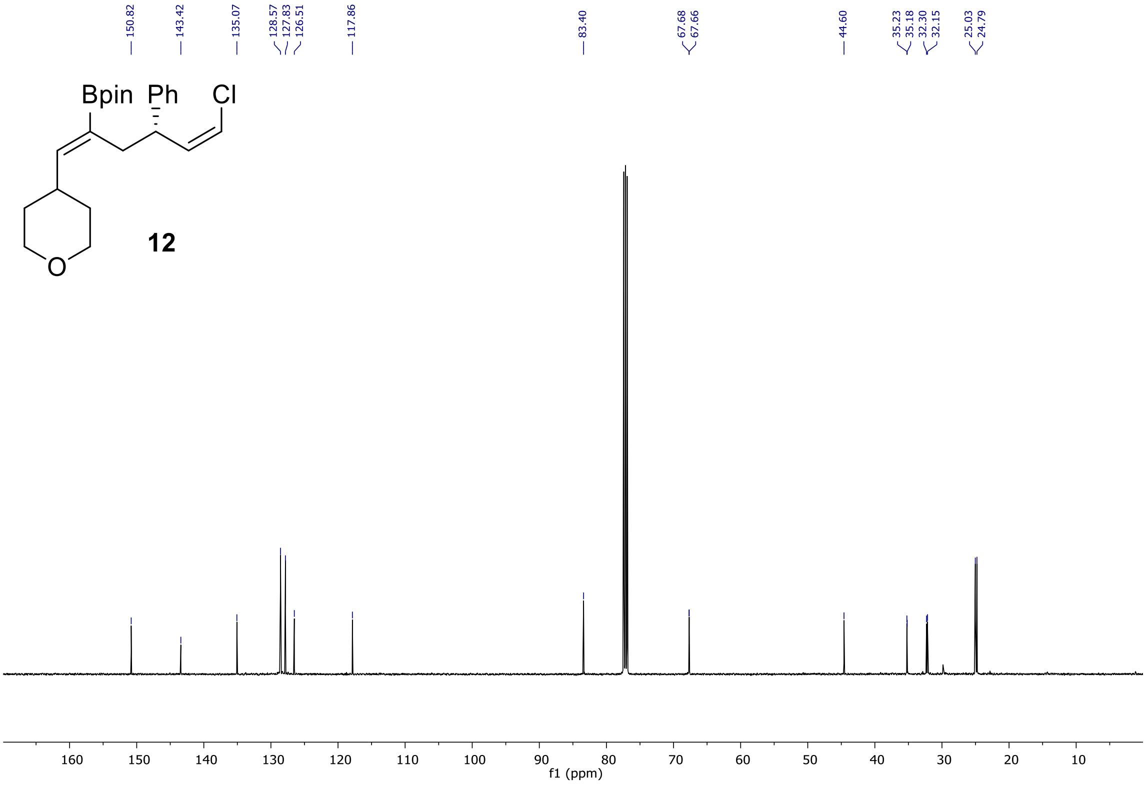

1H NMR (CDCl3, 300 MHz)

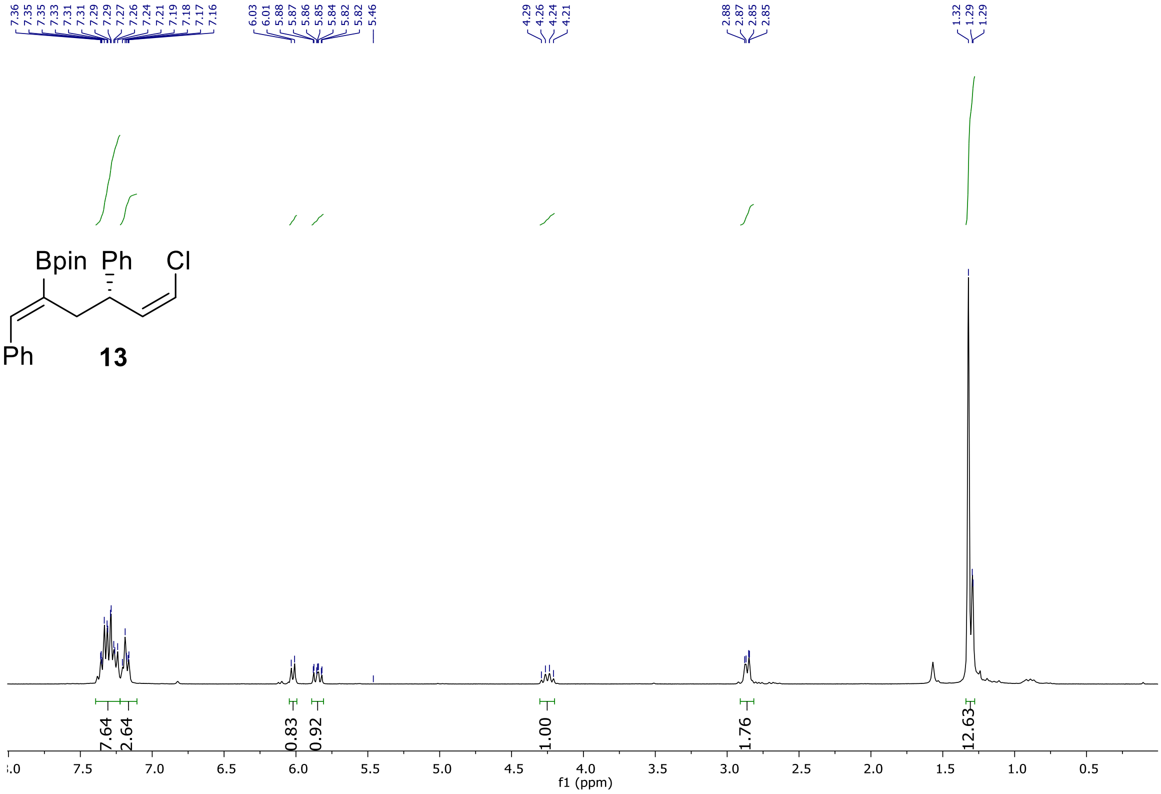

13C NMR (CDCl3, 75 MHz)

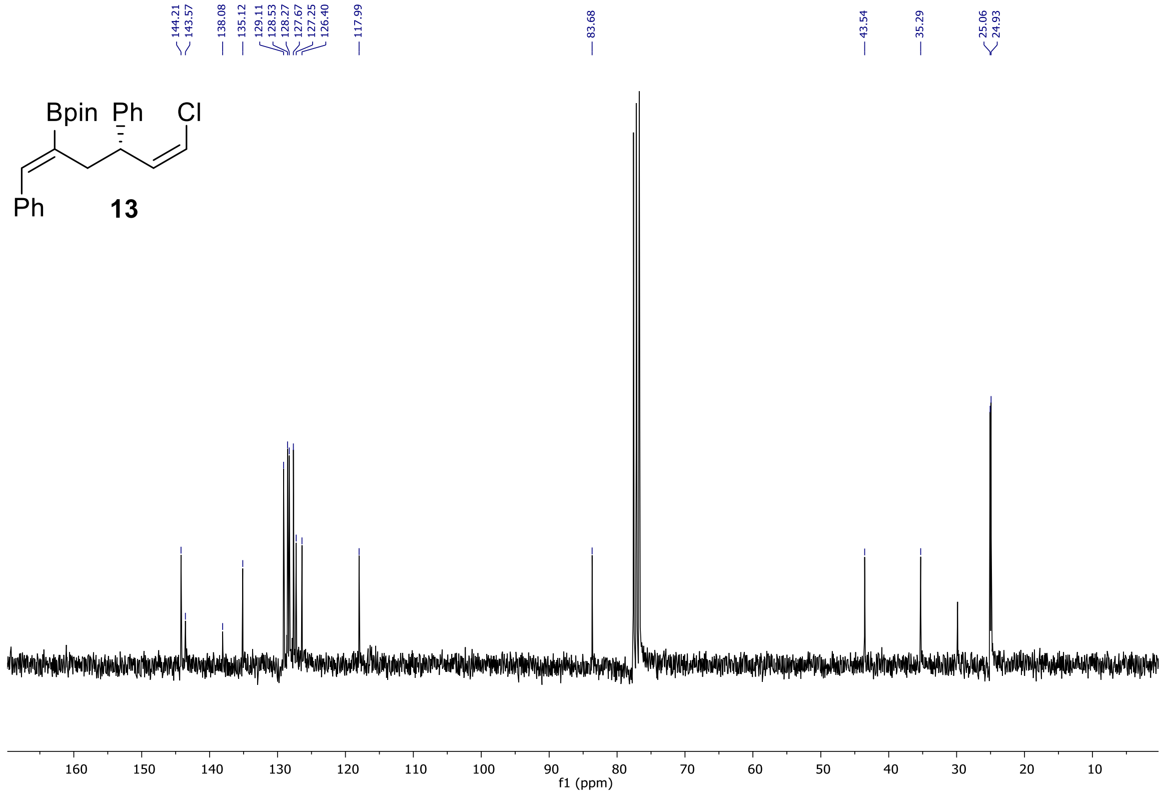

1H NMR (CDCl3, 500 MHz)

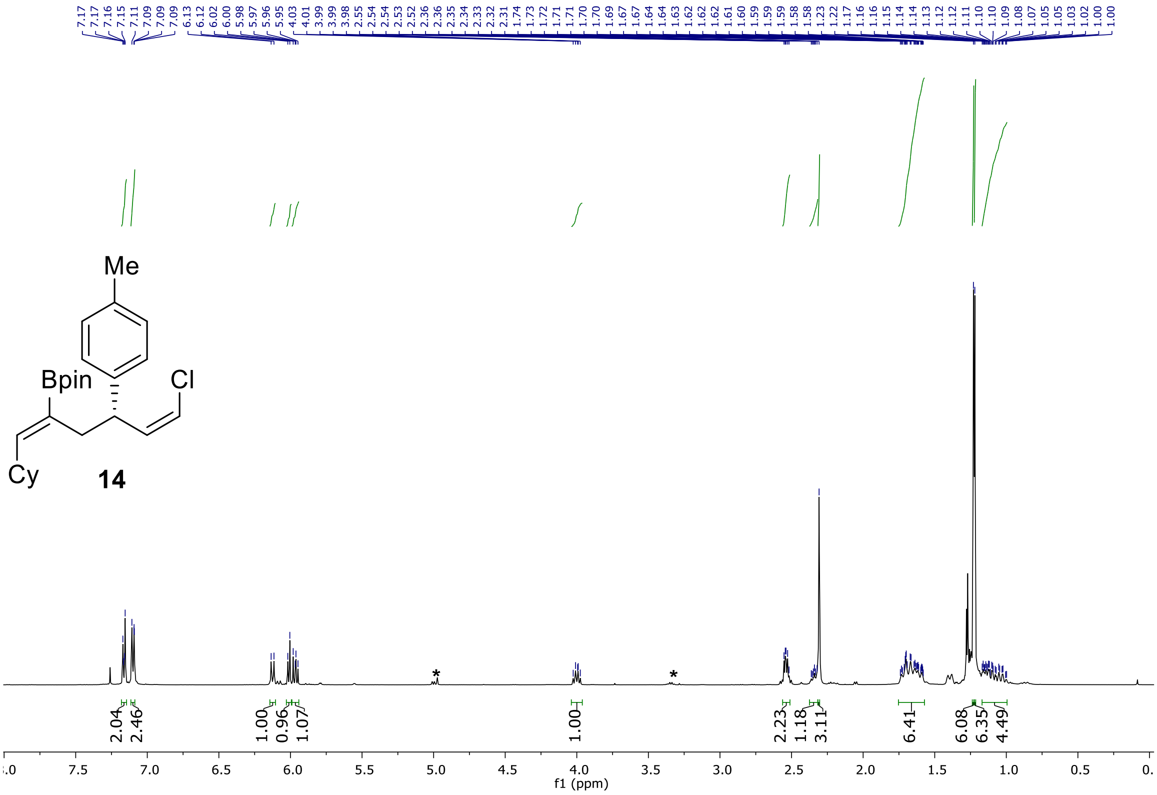

* These signals belong to the dechlorinated product.

13C NMR (CDCl3, 126 MHz)

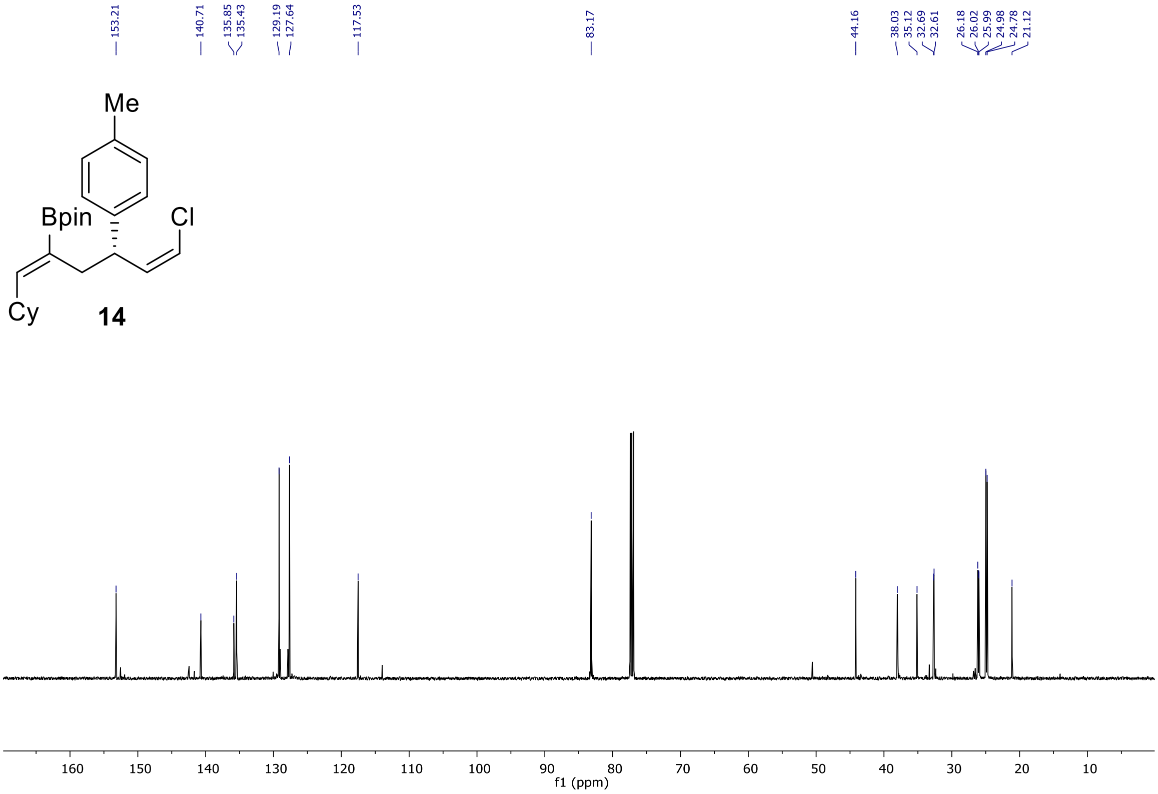

1H NMR (CDCl3, 500 MHz)

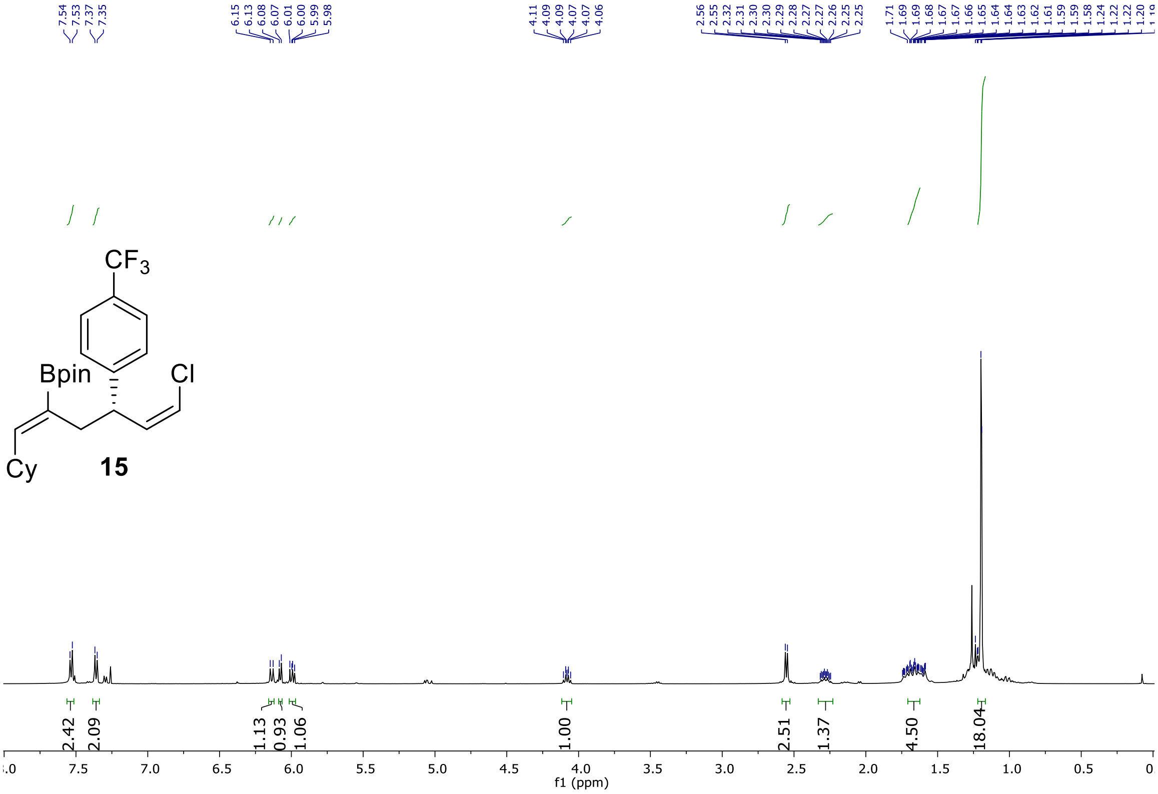

13C NMR (CDCl3, 126 MHz)

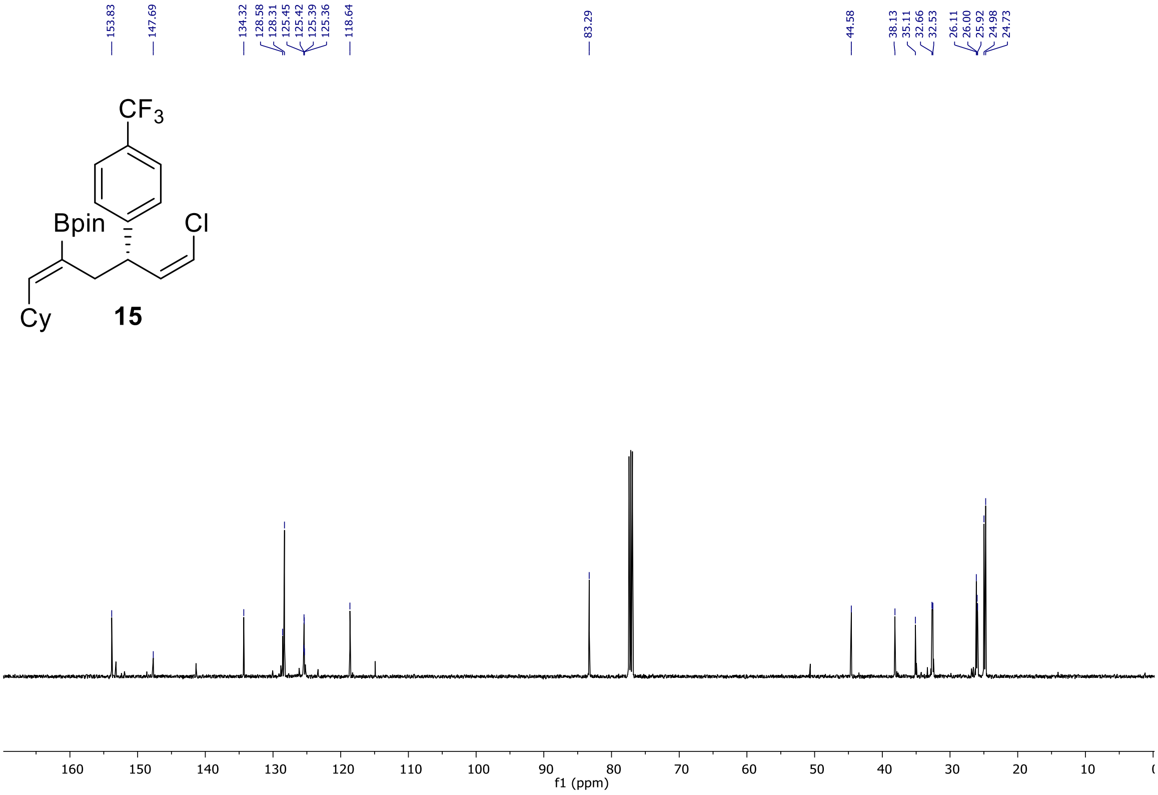

1H NMR (CDCl3, 500 MHz)

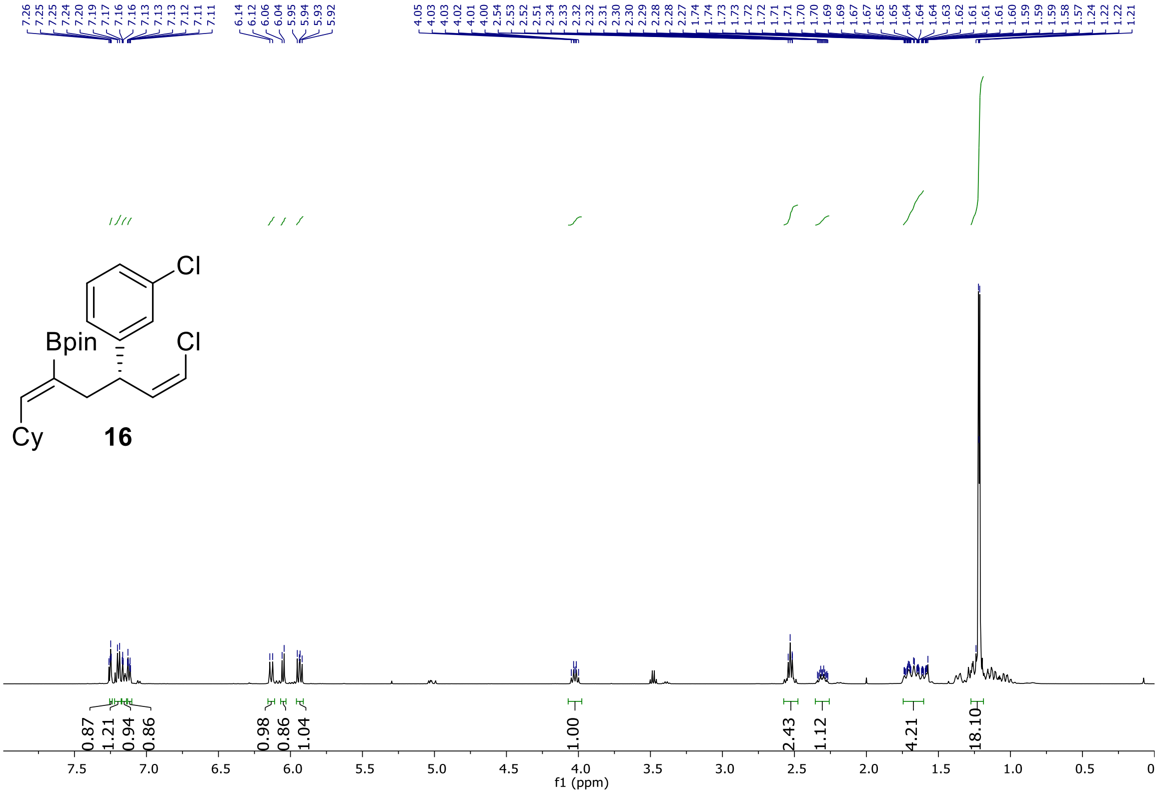

13C NMR (CDCl3, 126 MHz)

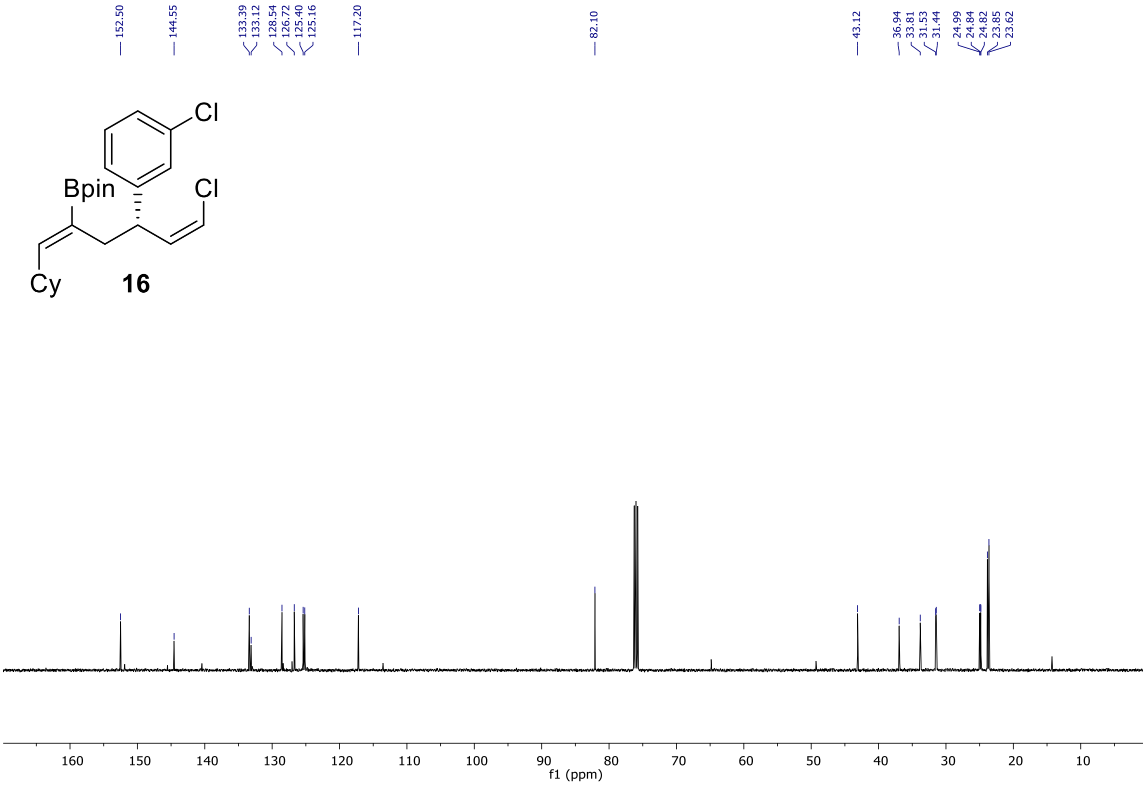

1H NMR (CDCl3, 500 MHz)

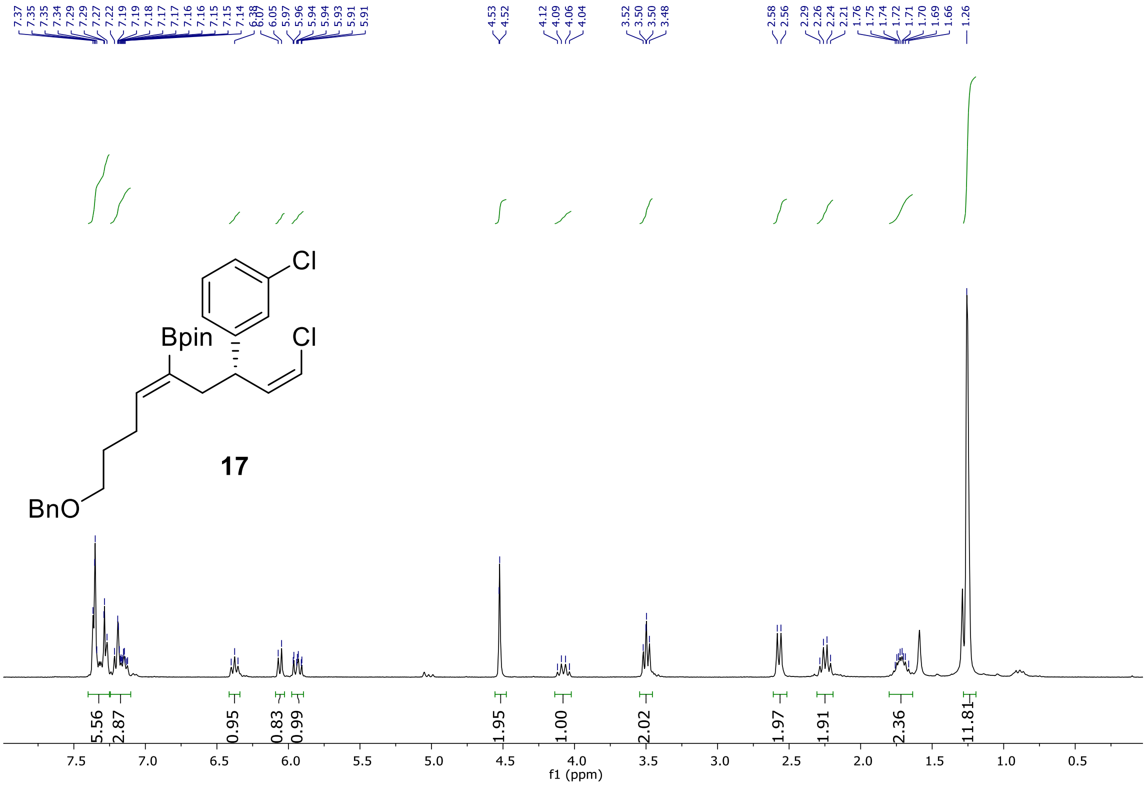

13C NMR (CDCl3, 75 MHz)

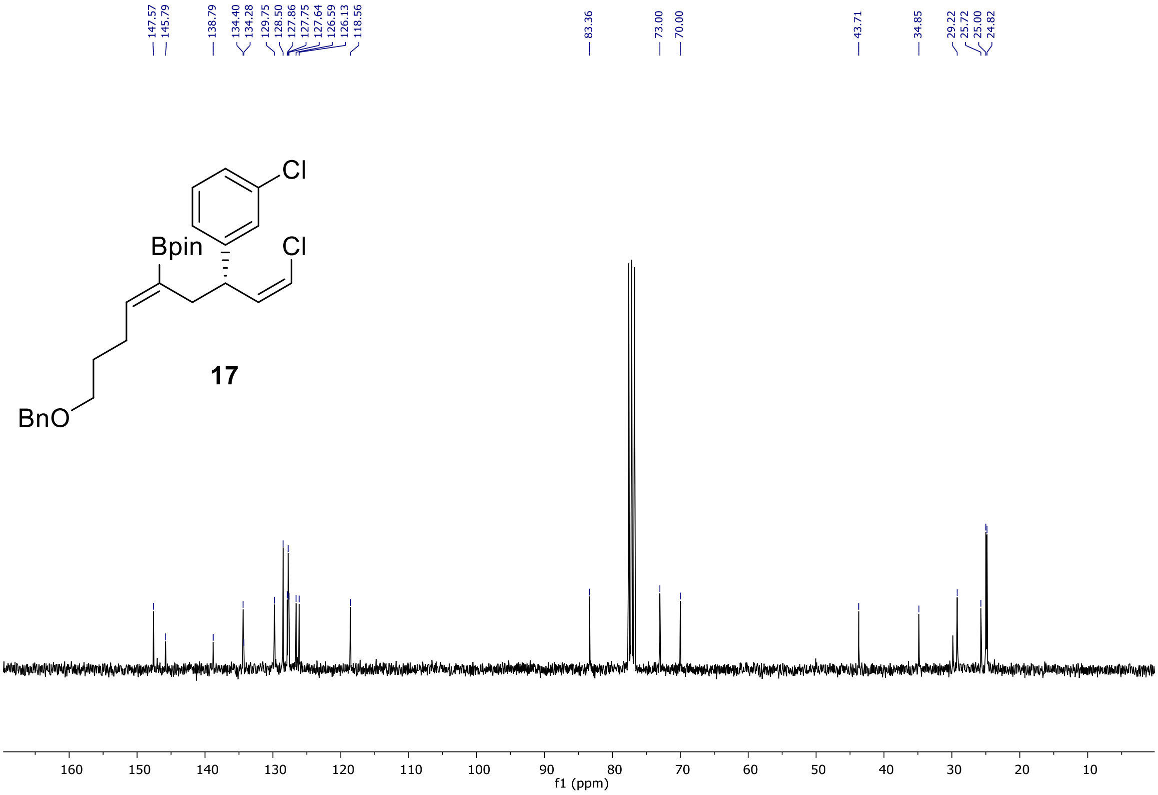

1H NMR (CDCl3, 500 MHz)

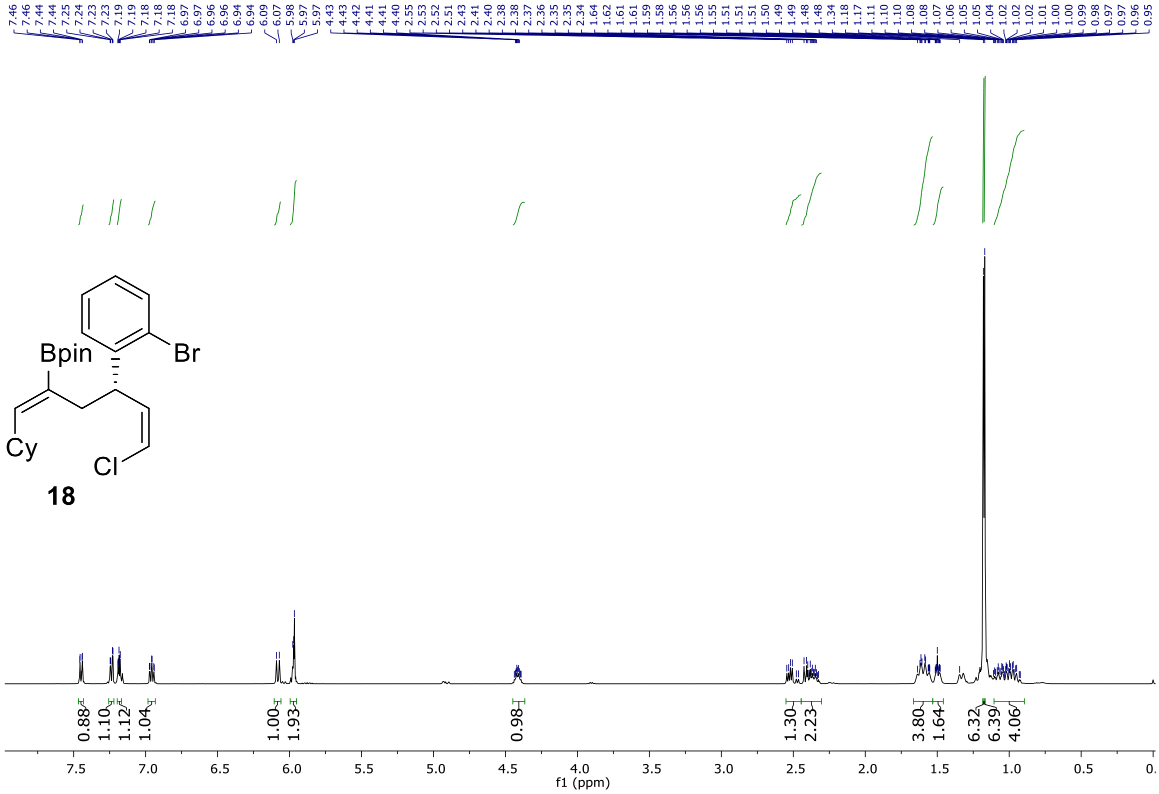

13C NMR (CDCl3, 126 MHz)

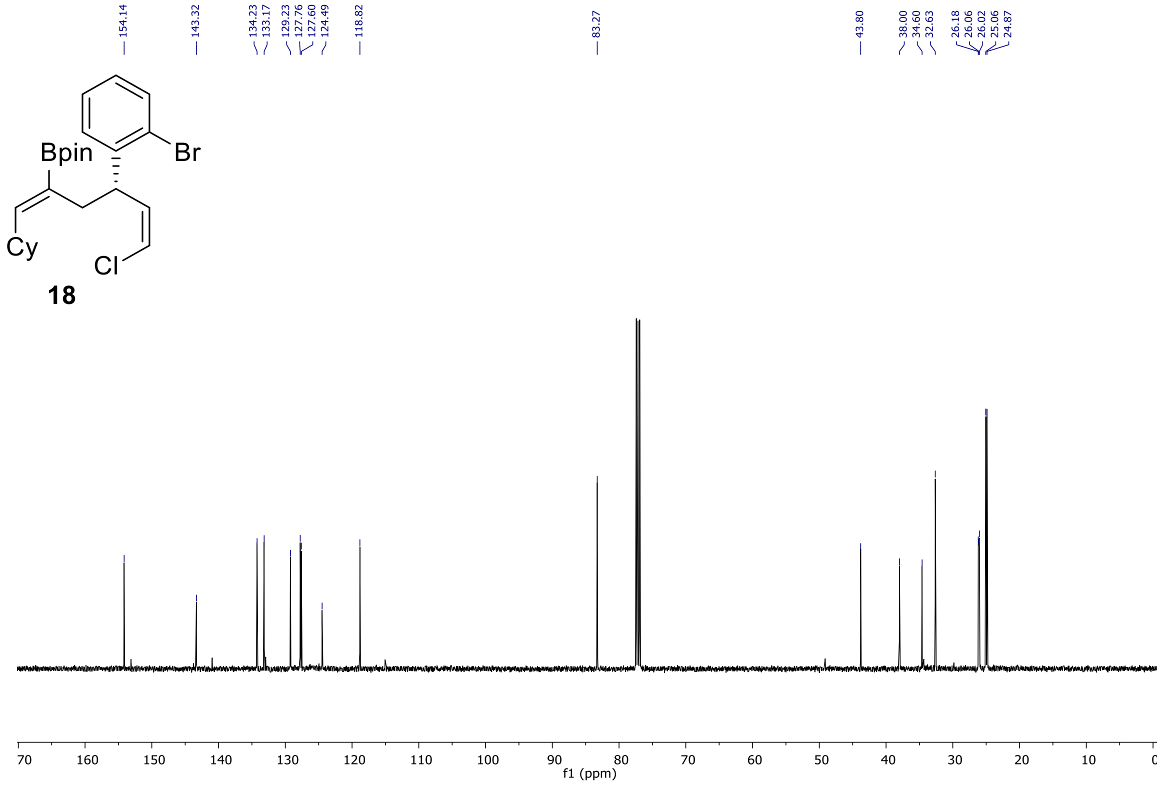

1H NMR (CDCl3, 500 MHz)

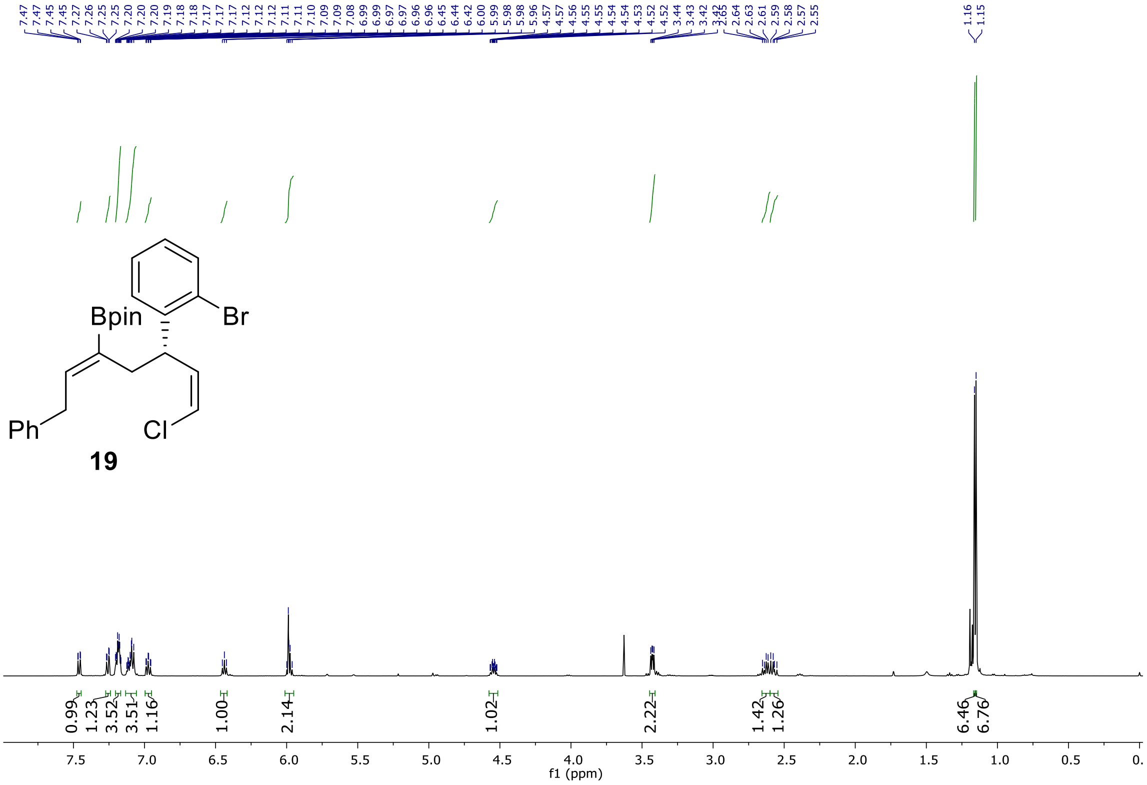

13C NMR (CDCl3, 126 MHz)

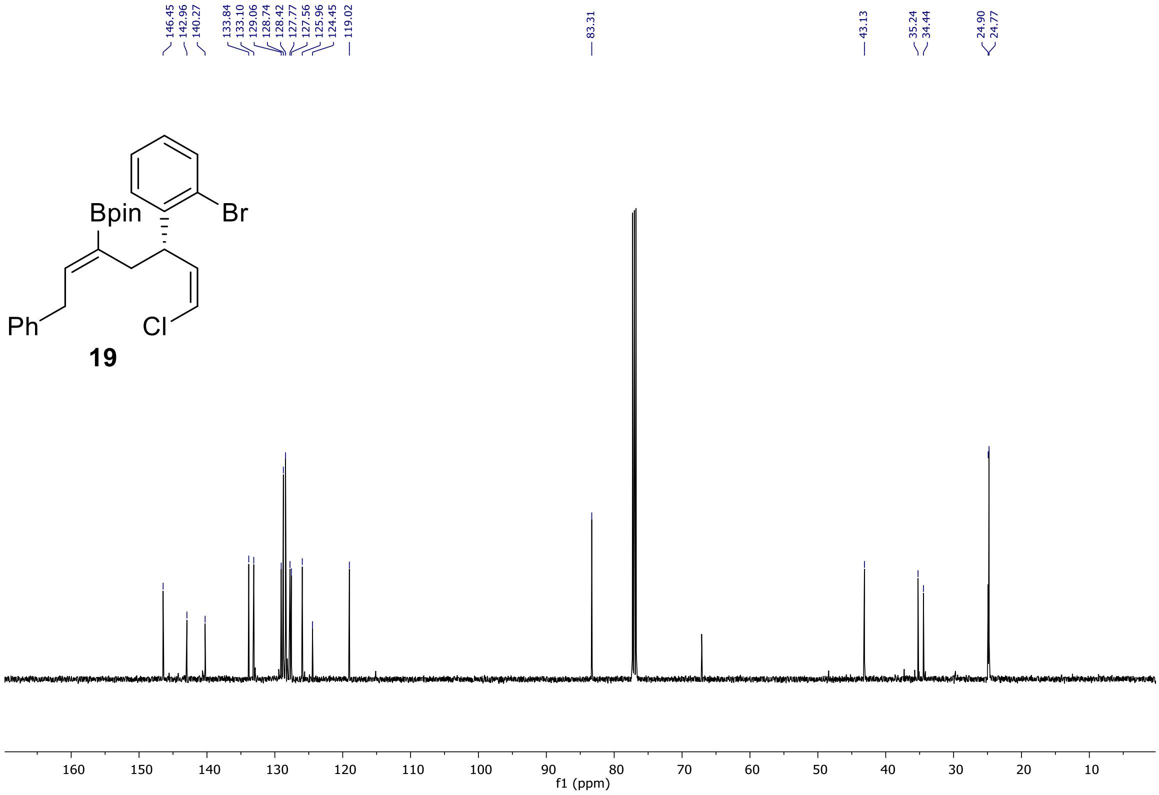

1H NMR (CDCl3, 500 MHz)

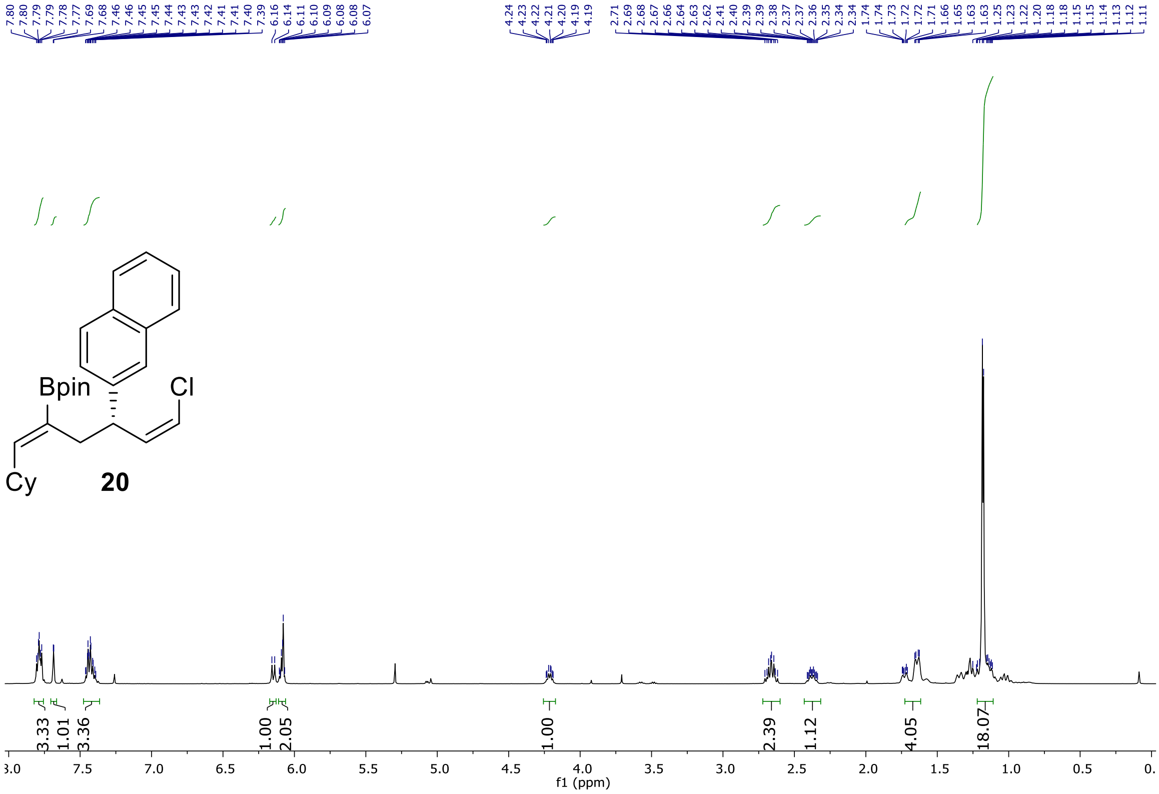

13C NMR (CDCl3, 126 MHz)

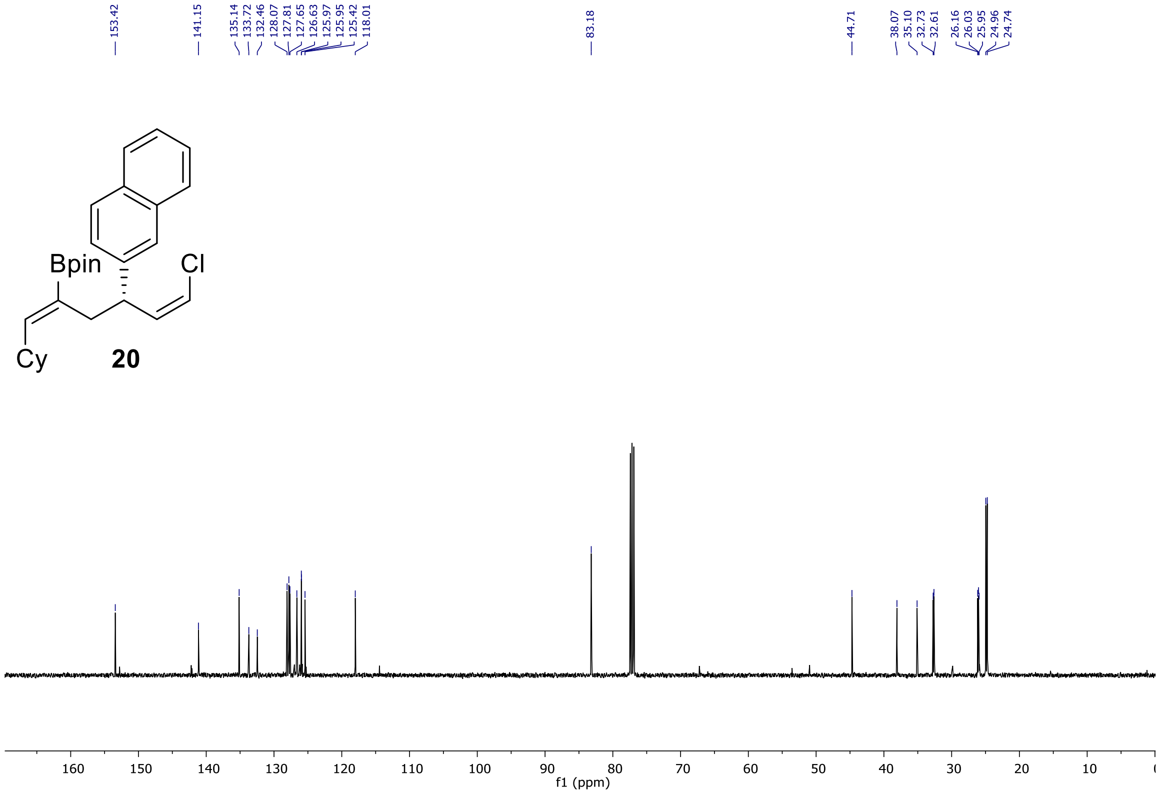

1H NMR (CDCl3, 300 MHz)

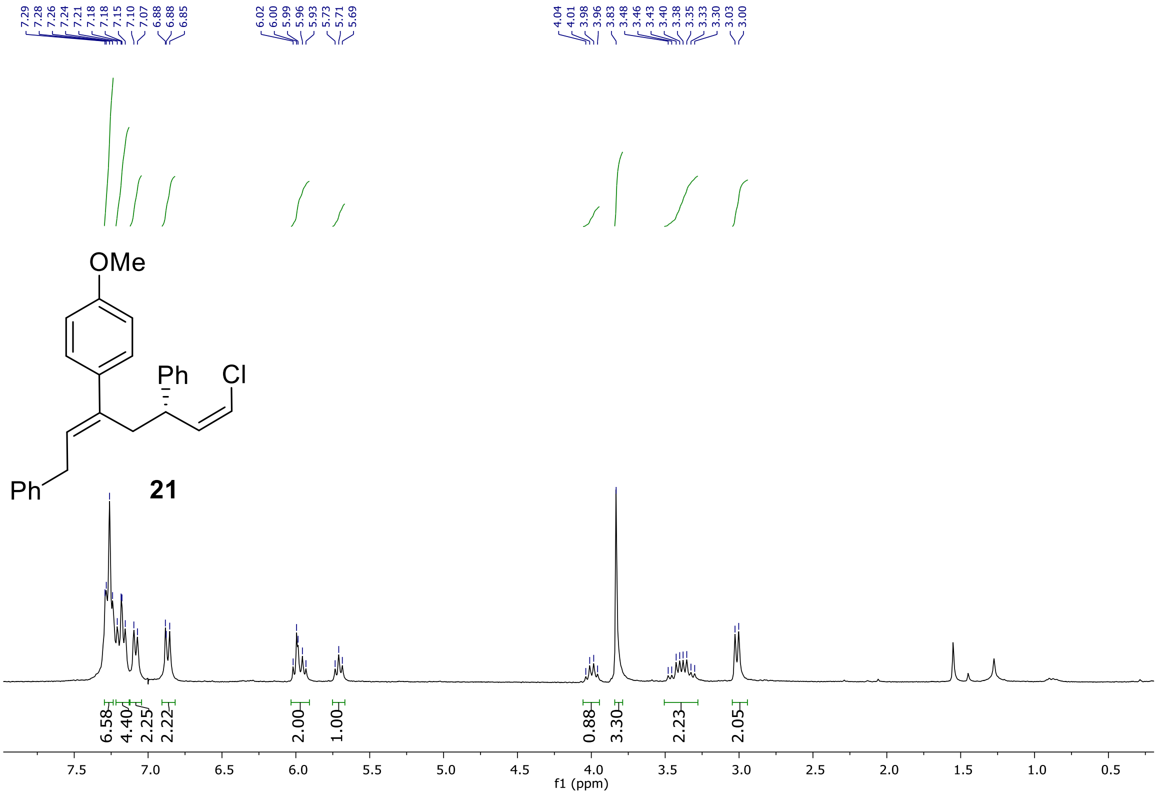

21

13C NMR (CDCl3, 75 MHz)

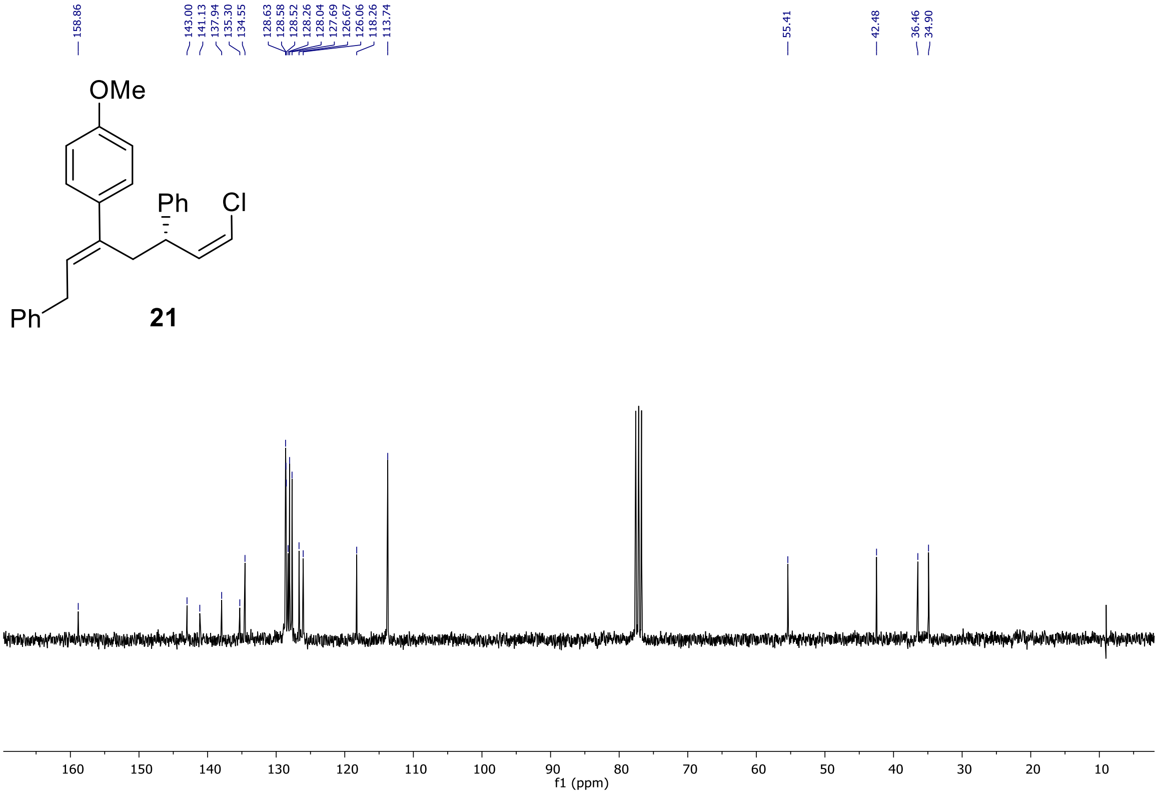

21

1H NMR (CDCl3, 300 MHz)

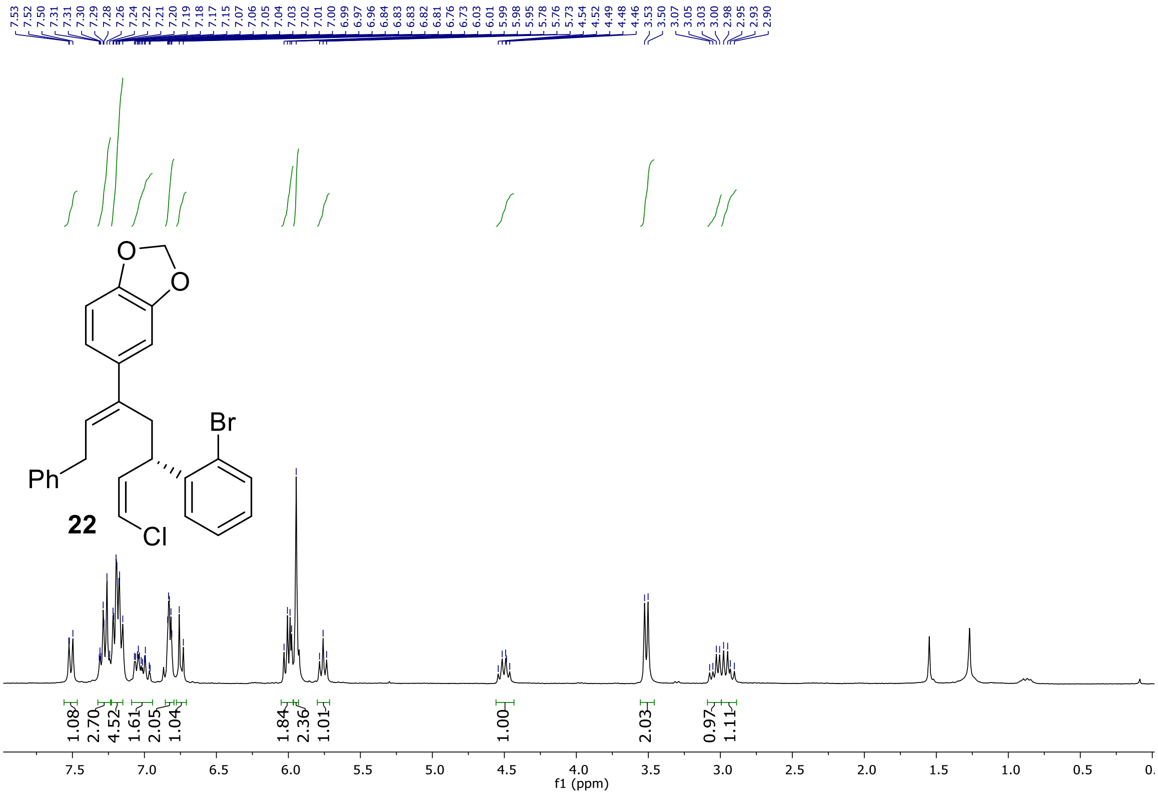

13C NMR (CDCl3, 75 MHz)

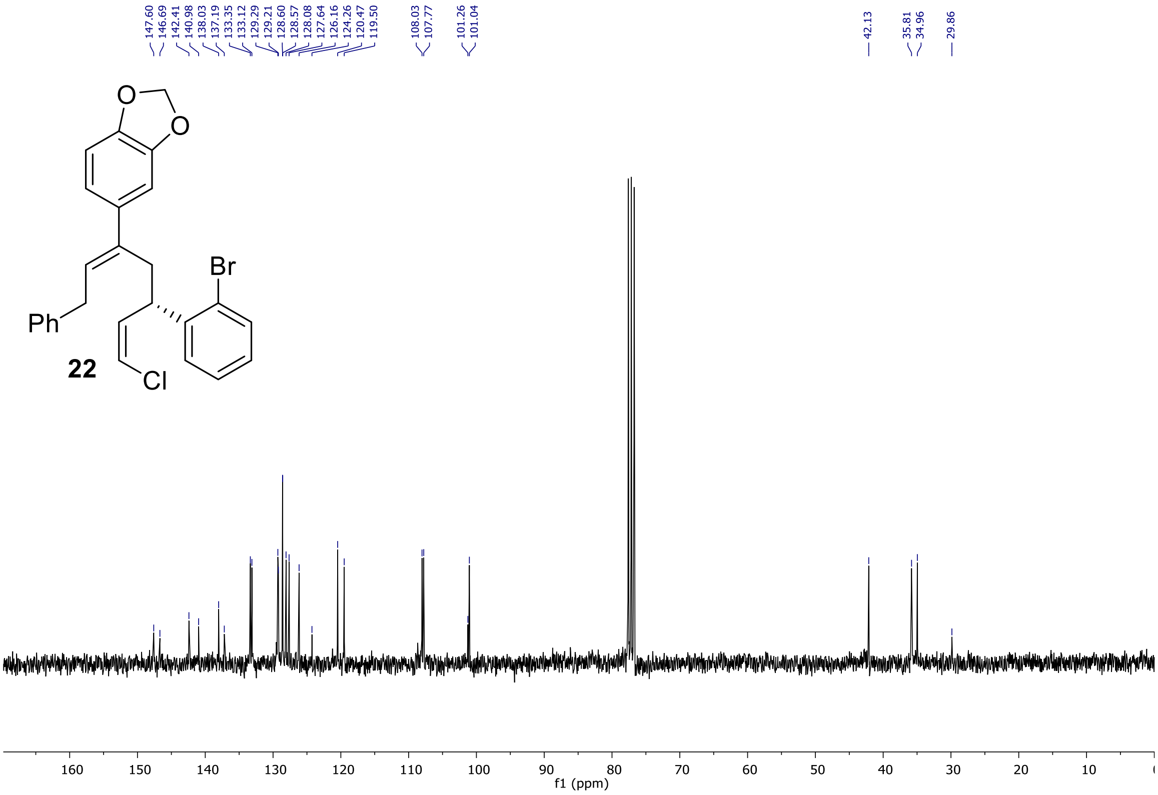

1H NMR (CDCl3, 300 MHz)

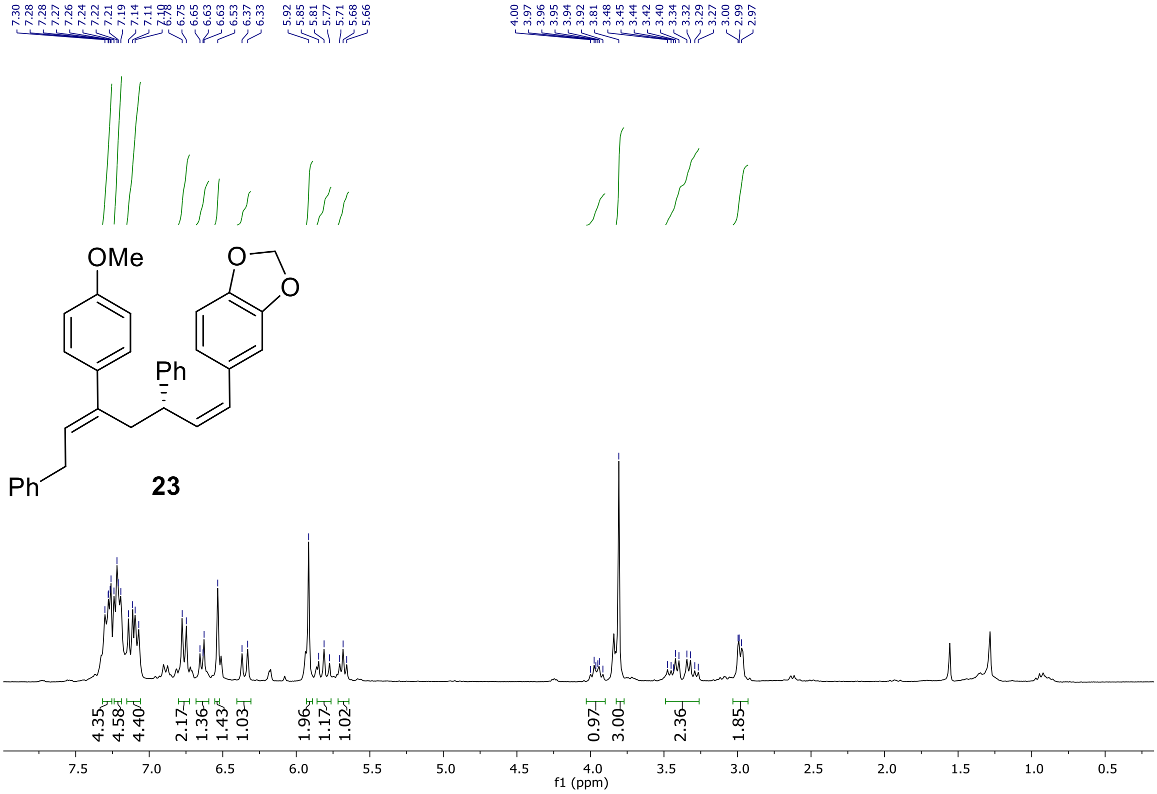

13C NMR (CDCl3, 75 MHz)

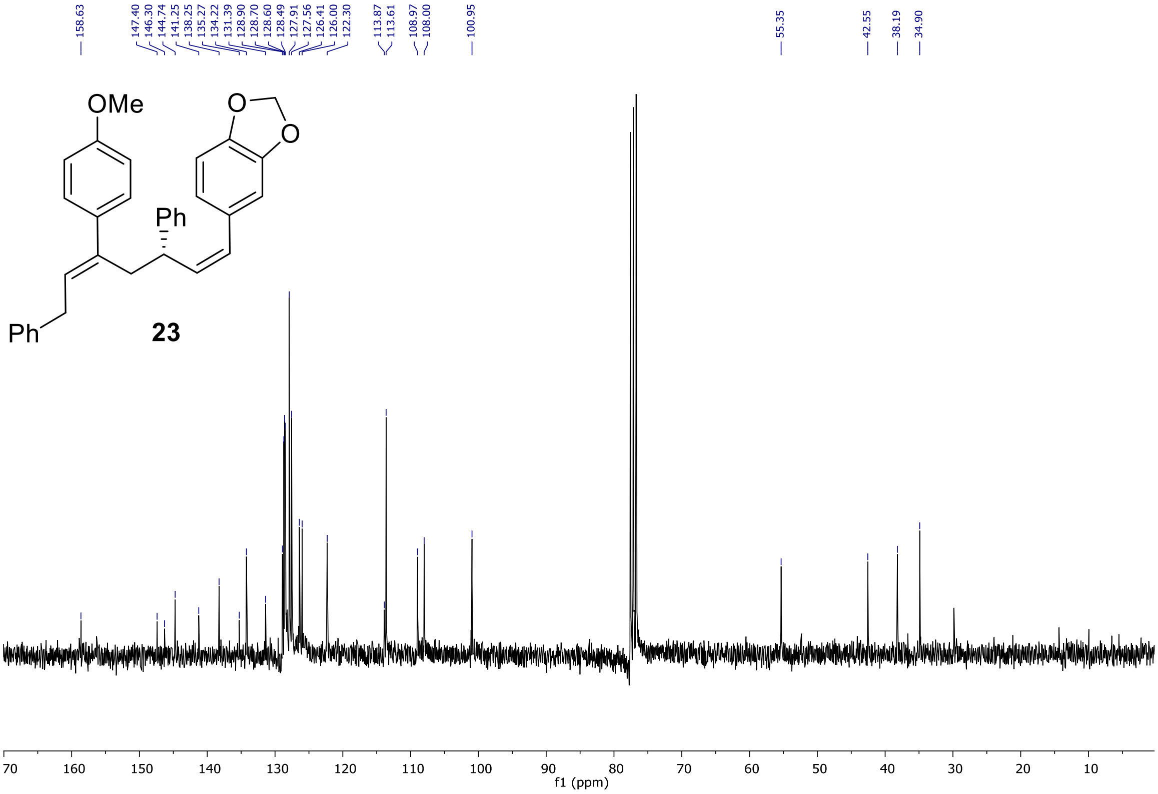

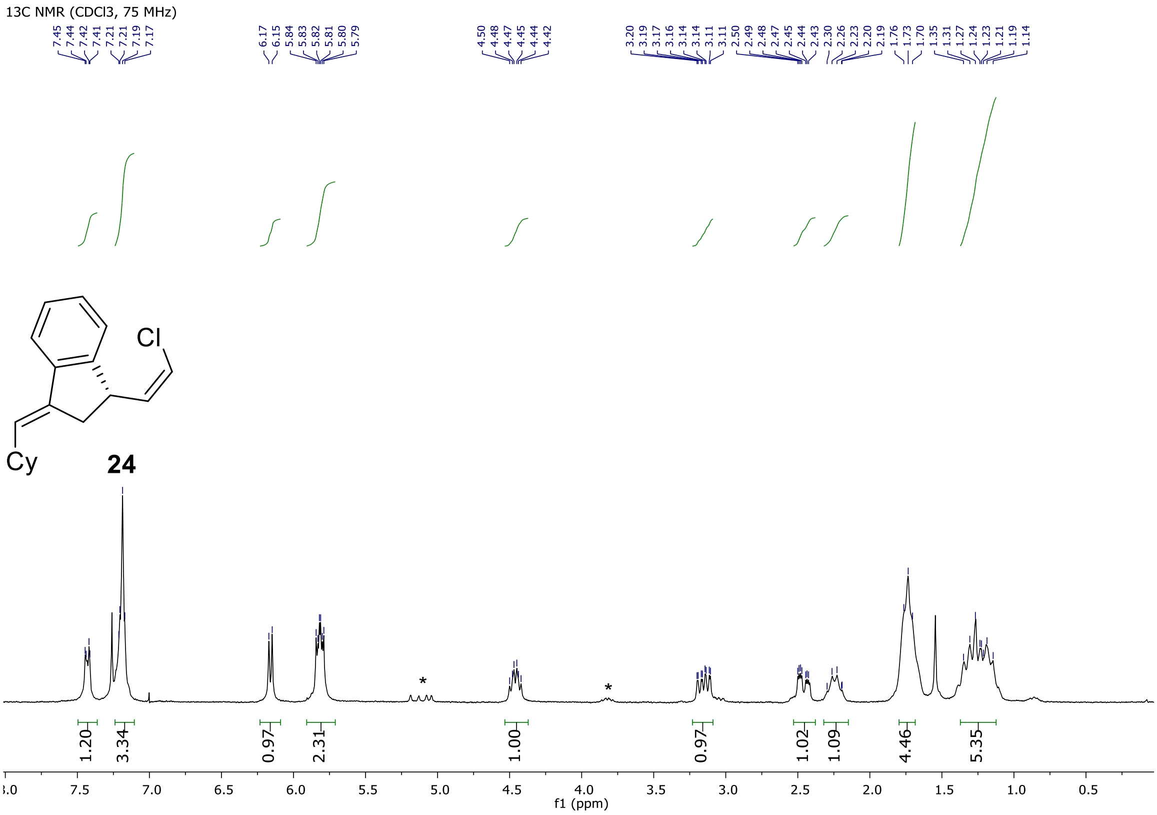

* These signals belong to the dechlorinated product.

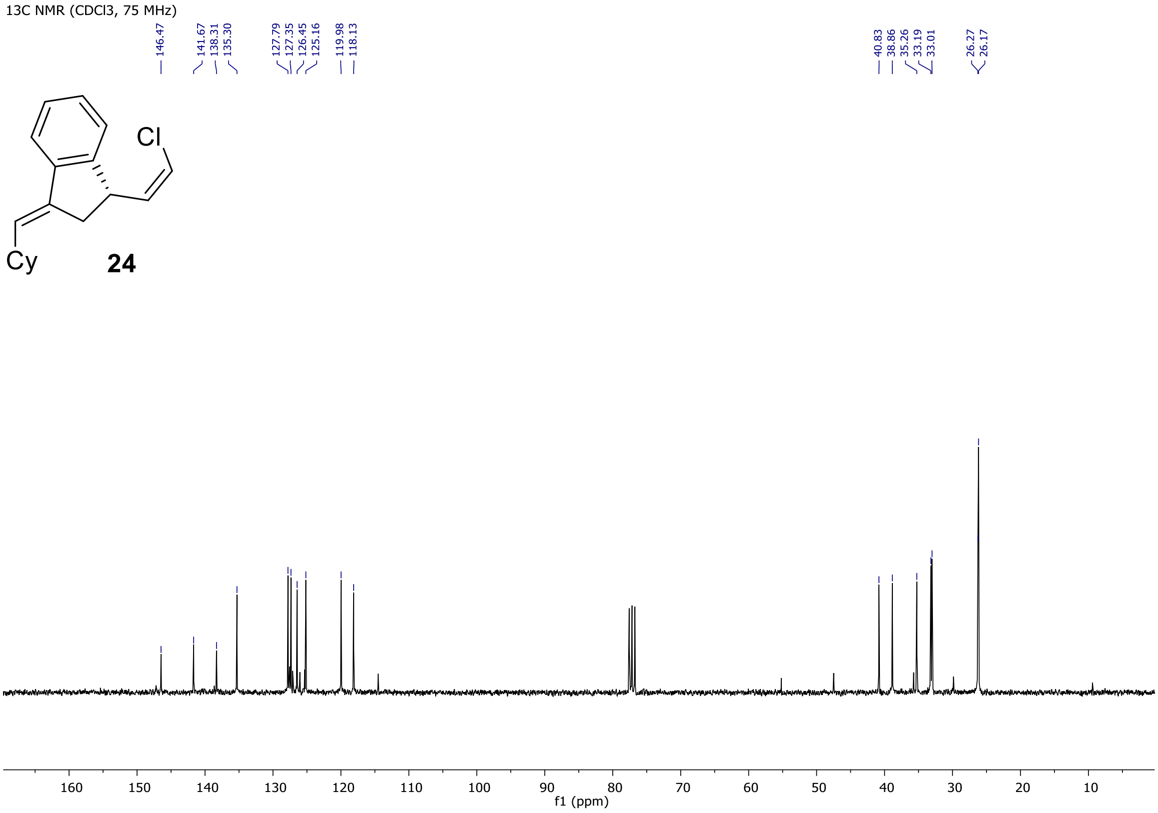

1H NMR (CDCl3, 500 MHz)

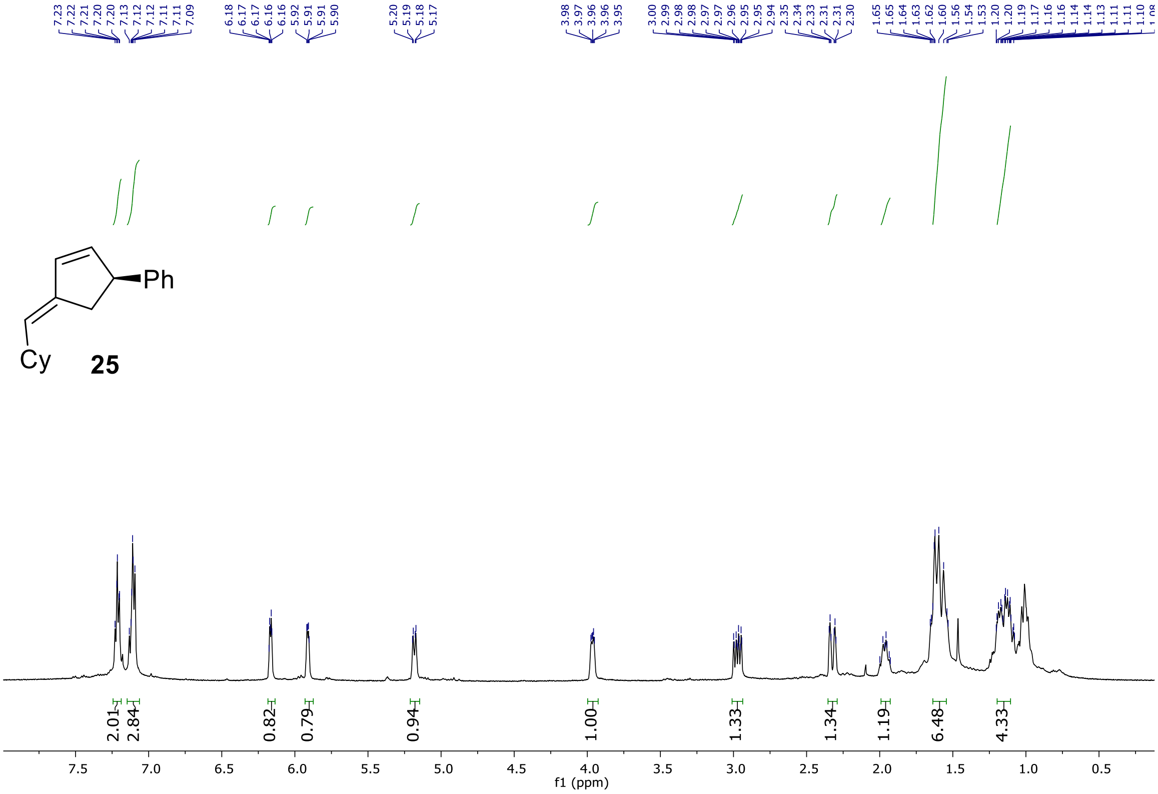

13C NMR (CDCl3, 126 MHz)

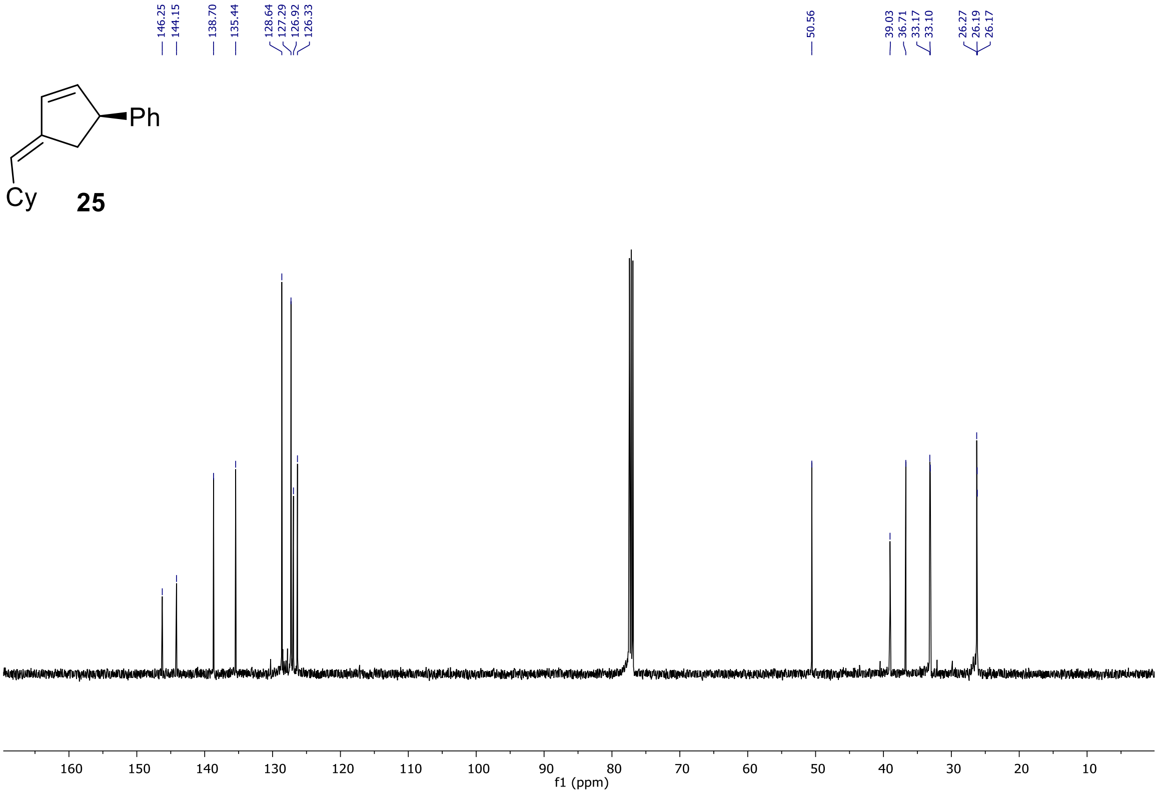

1H NMR (CDCl3, 500 MHz)

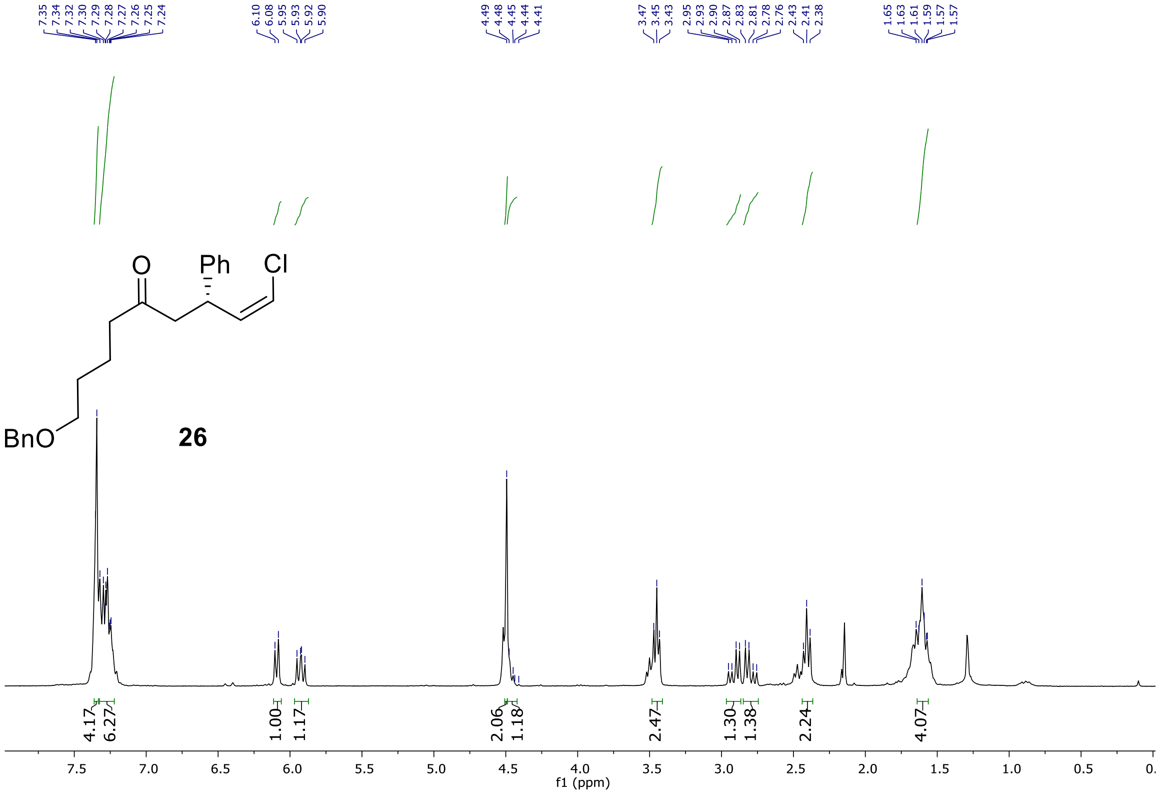

13C NMR (CDCl3, 75 MHz)

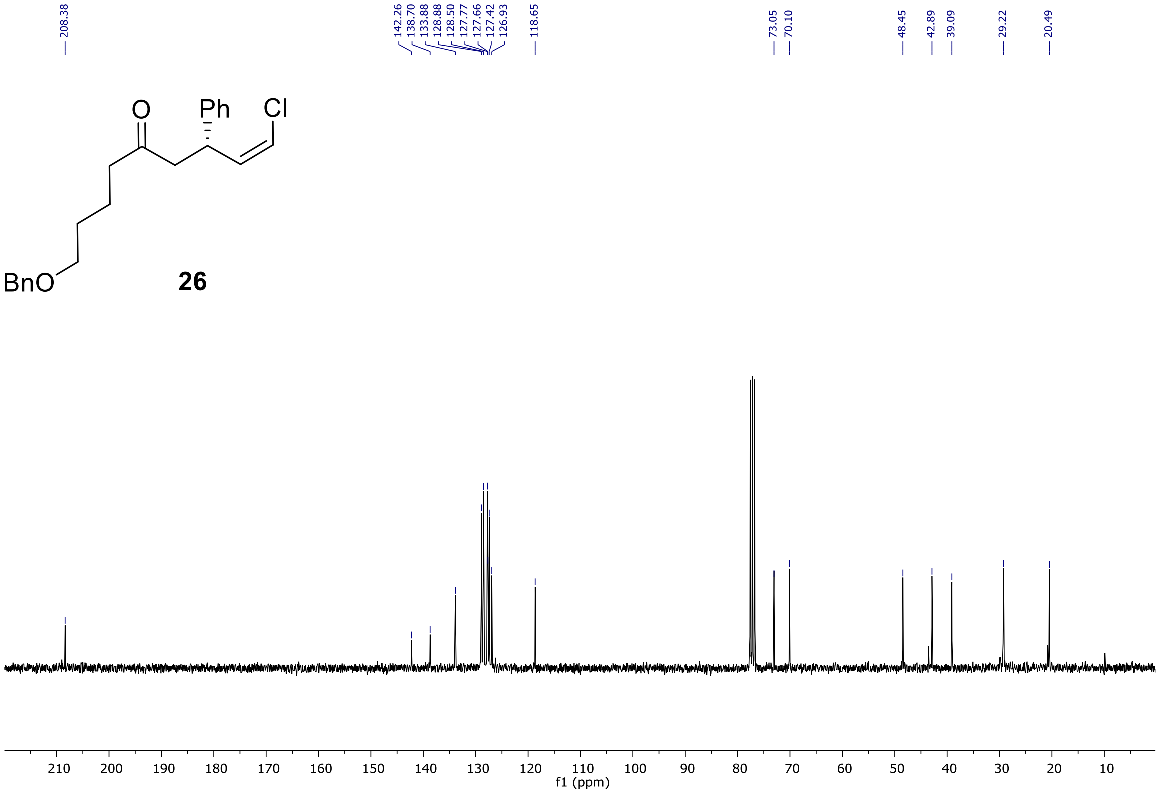

1H NMR (CDCl3, 500 MHz)

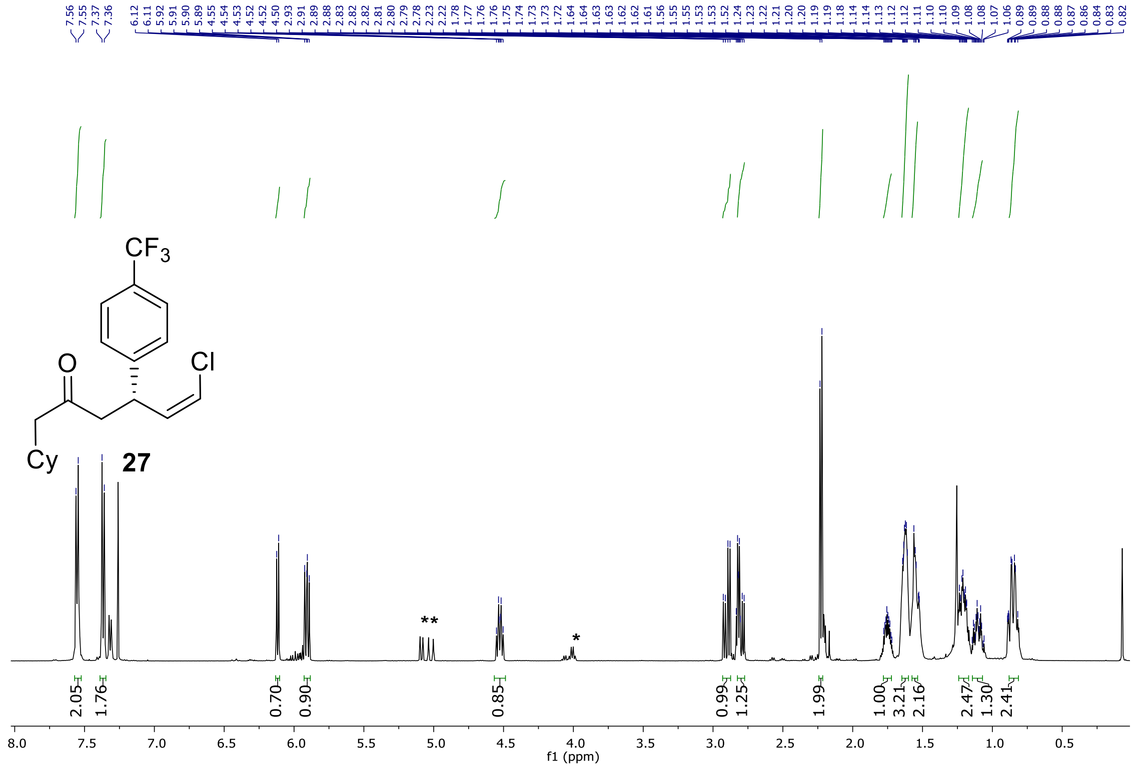

* These signals belong to the dechlorinated product.

13C NMR (CDCl3, 126 MHz)

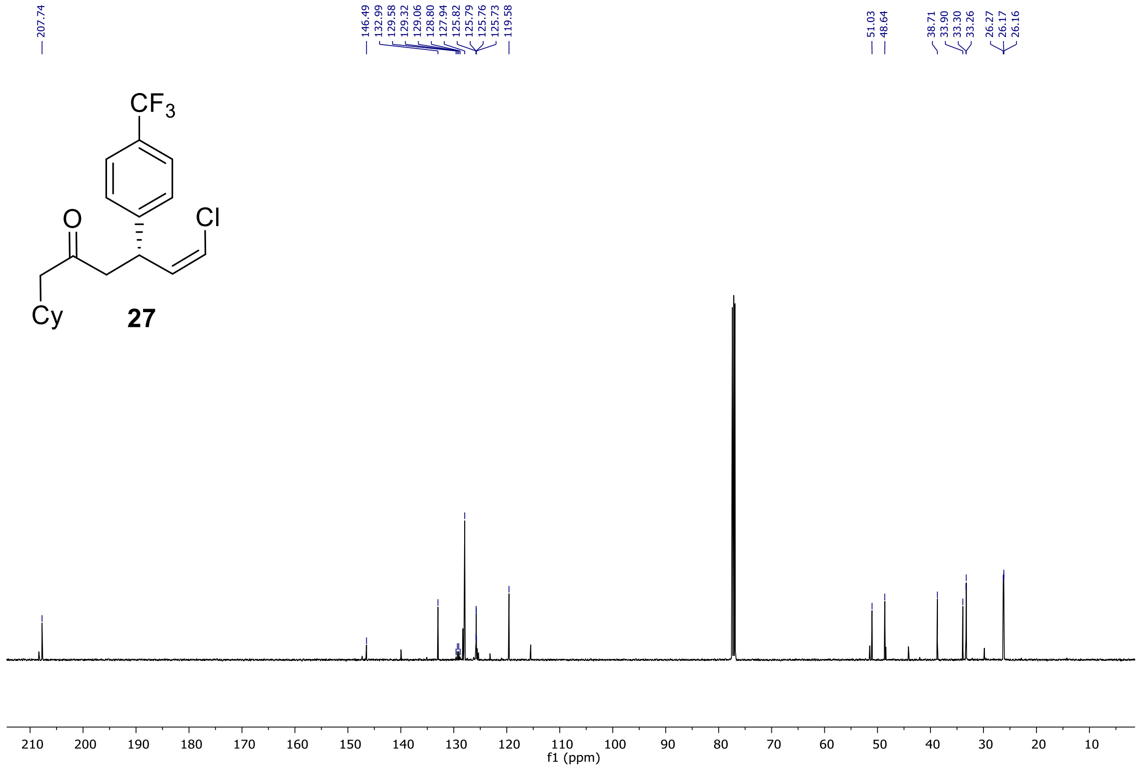

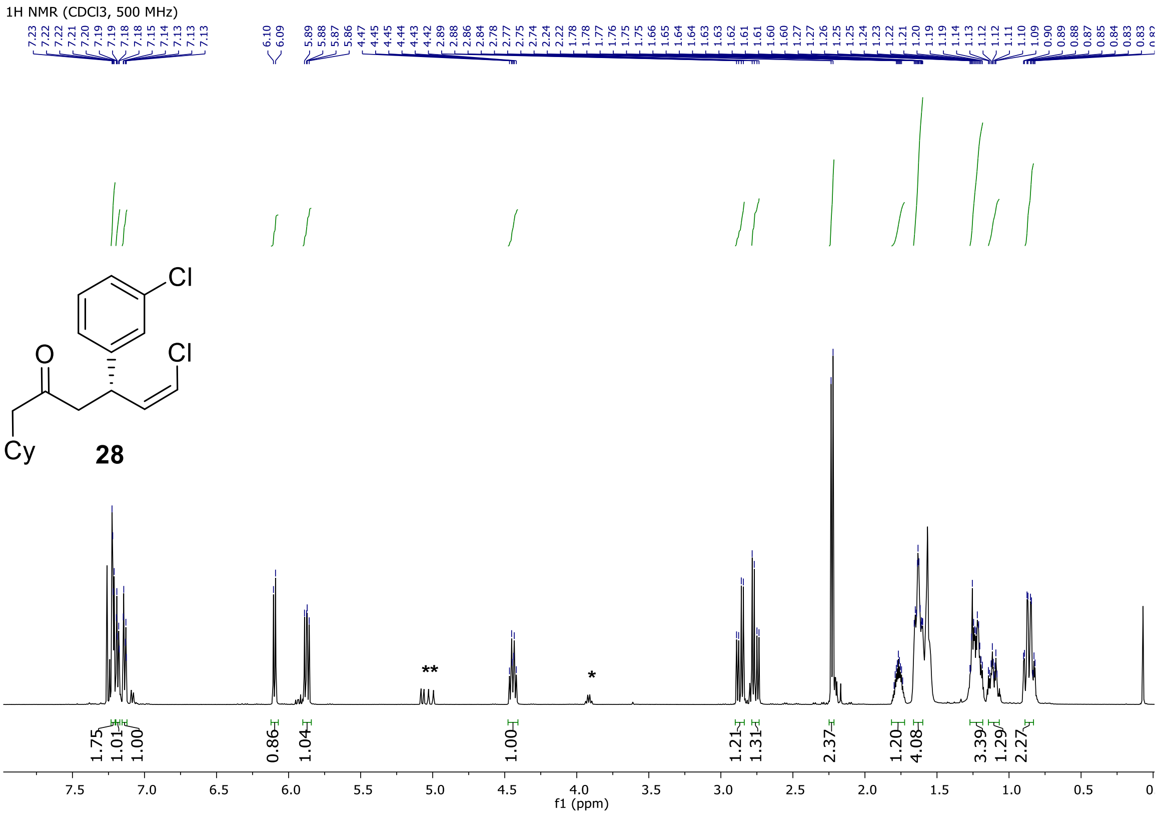

* These signals belong to the dechlorinated product.

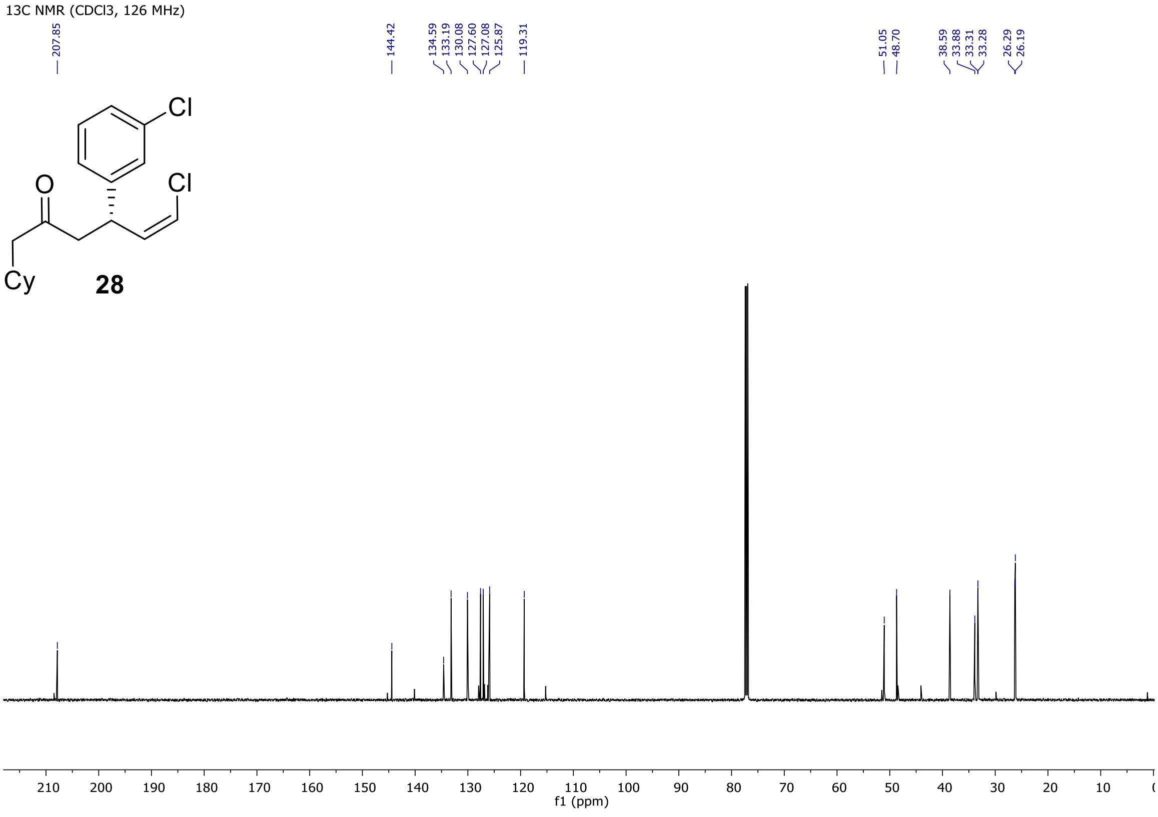

1H NMR (CDCl3, 300 MHz)

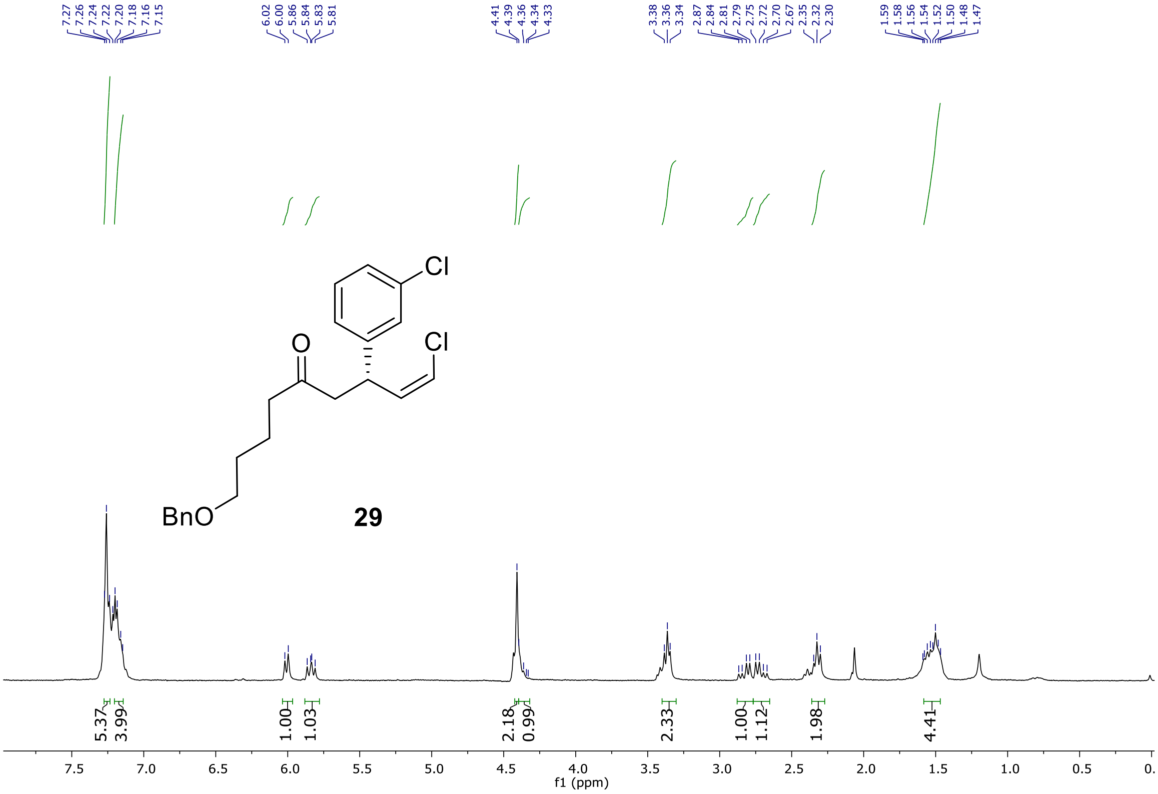

13C NMR (CDCl3, 75 MHz)

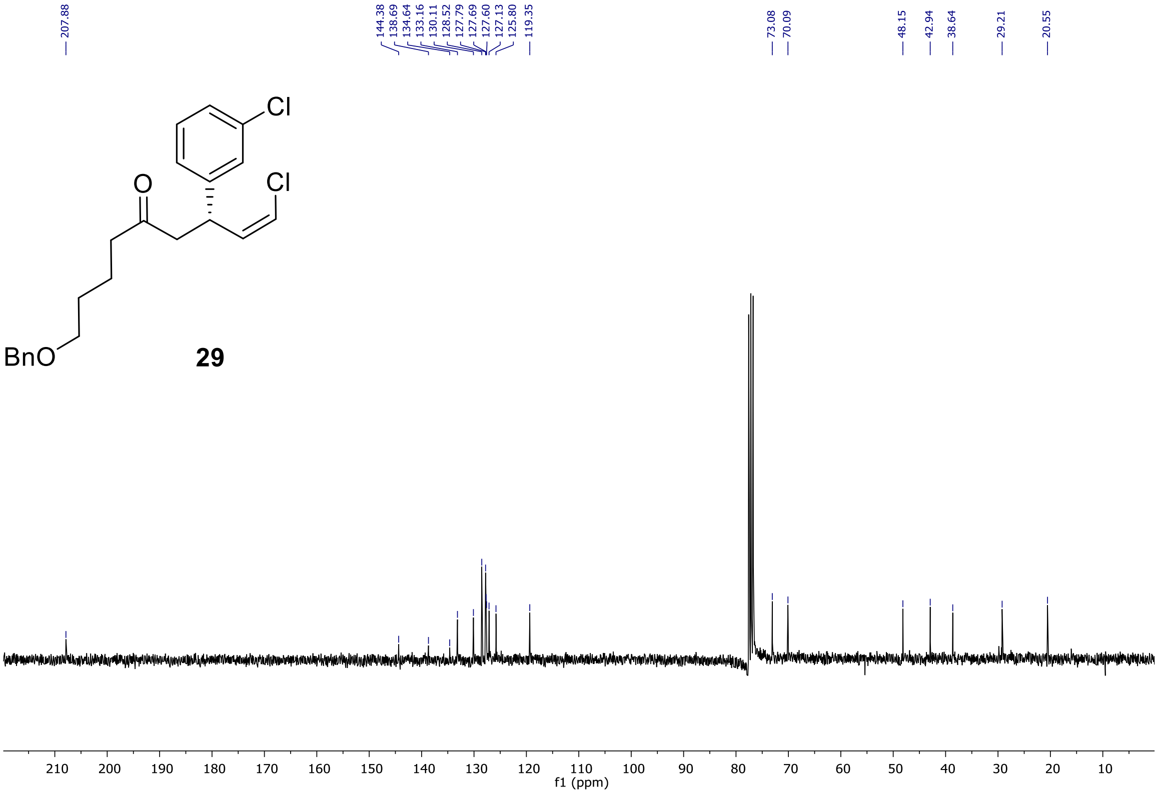

1H NMR (CDCl3, 500 MHz)

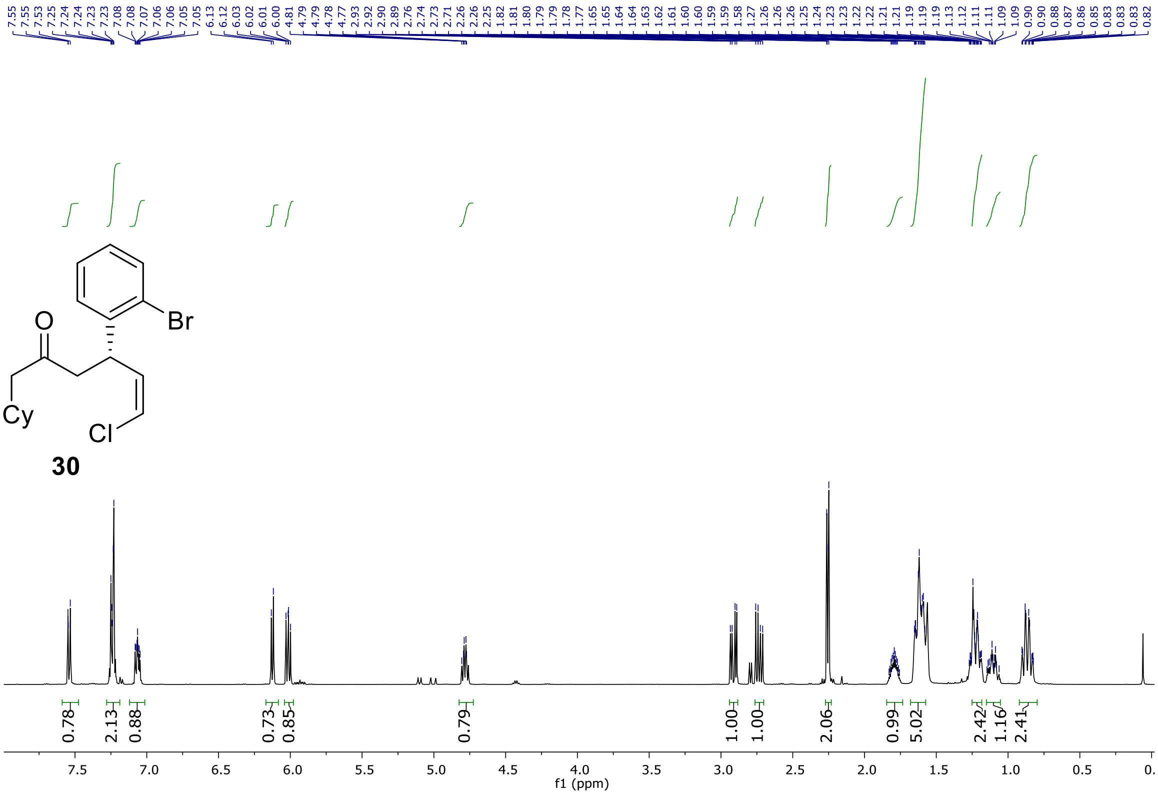

13C NMR (CDCl3, 126 MHz)

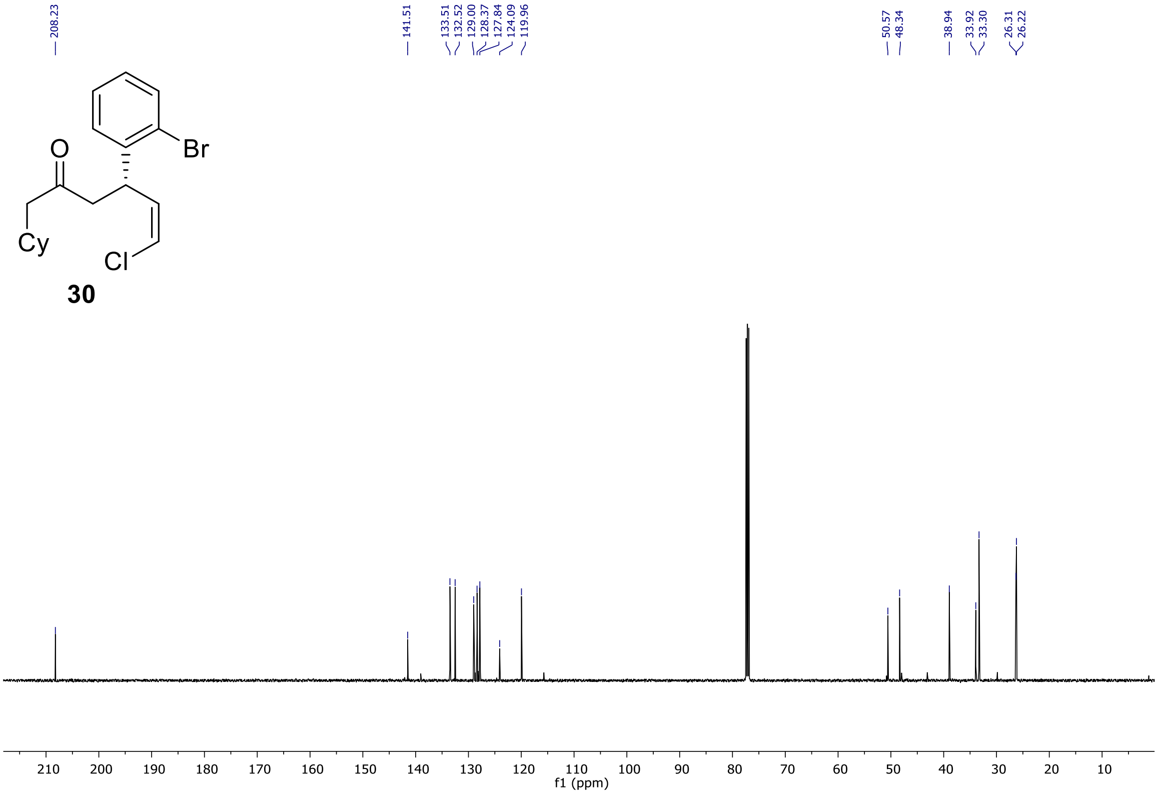

1H NMR (CDCl3, 300 MHz)

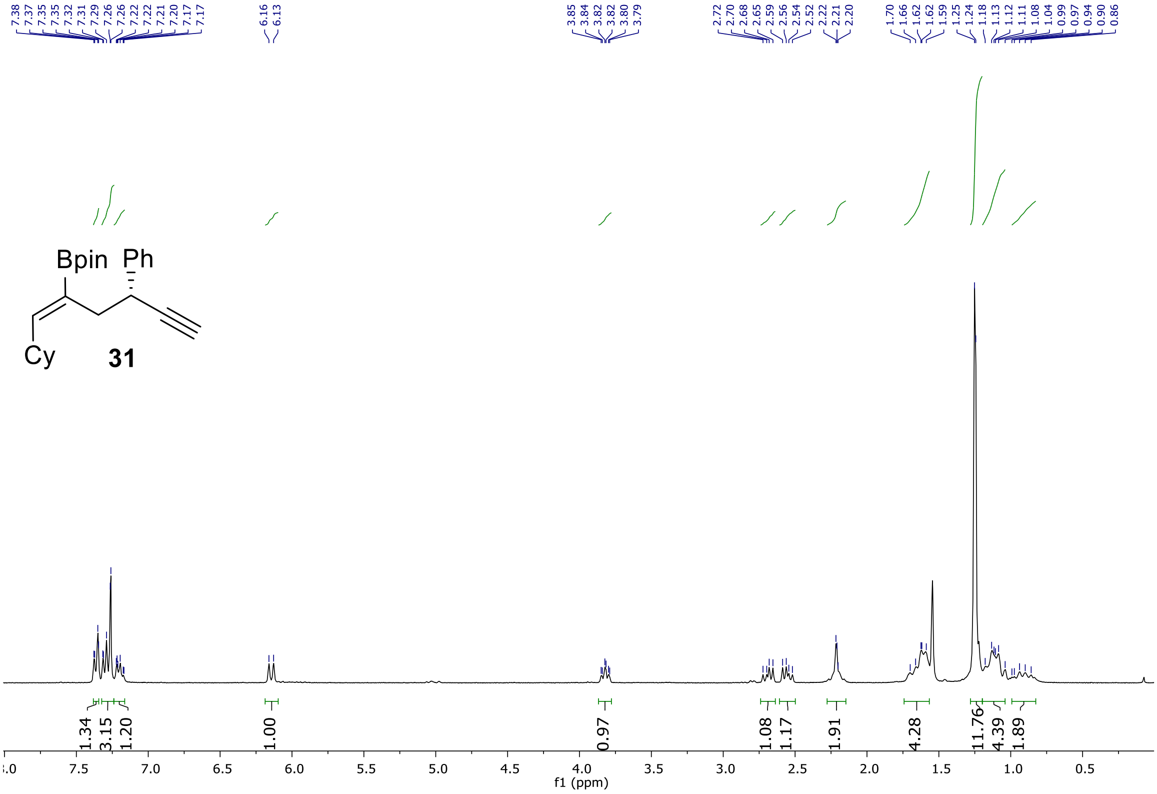

13C NMR (CDCl3, 75 MHz)

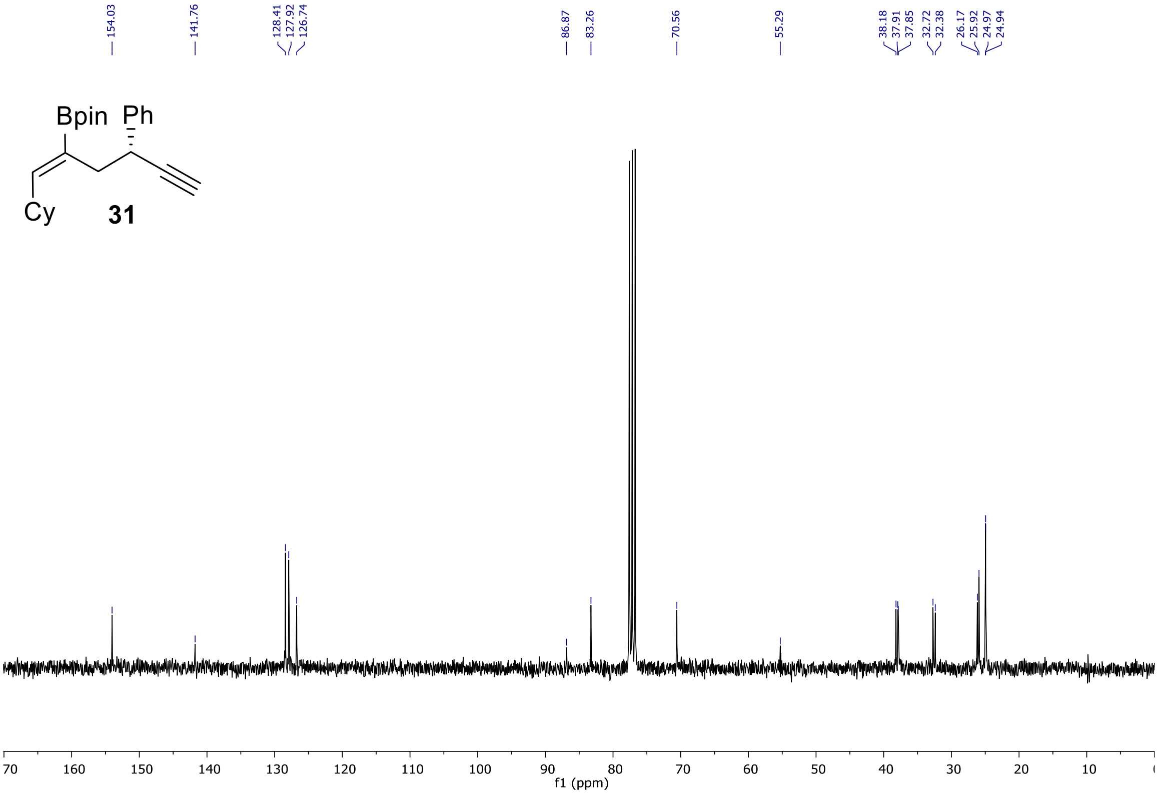

11. X-ray diffraction analysis data of product **11**

Product **11** was dissolved in CH_2Cl_2 and left to slow evaporation overnight to yield block, clear colorless type of crystals of an approximate dimensions 0.020 mm x 0.040 mm x 0.200 mm. The X-ray intensity data were measured on a Bruker D8 VENTURE PHOTON-III C14 κ-geometry diffractometer system equipped with a Incoatec IμS 3.0 microfocus sealed tube (Cu Kα, λ = 1.54178 Å) and a multilayer mirror monochromator. The structure was solved and refined using the Bruker SHELXTL Software Package.

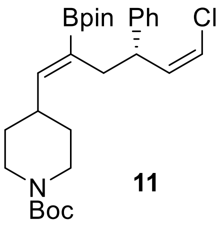

11

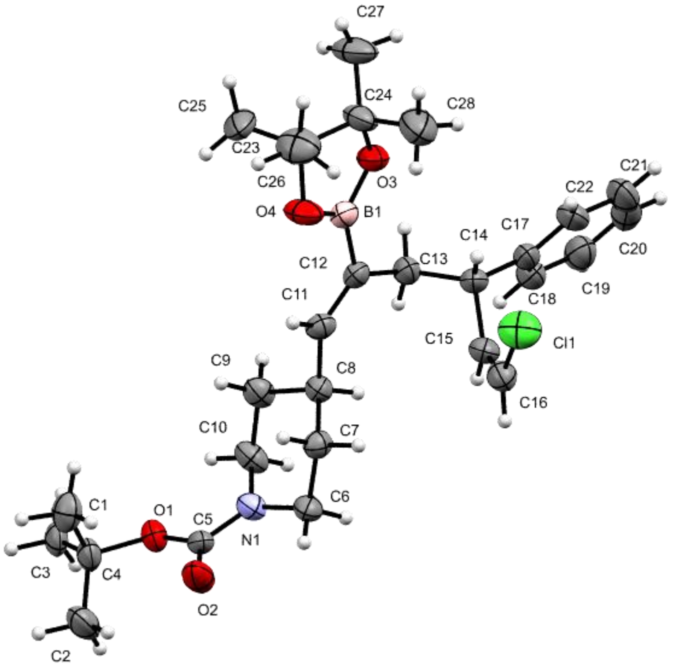

Figure S1. ORTEP plot of **11** with 30% ellipsoids. Selected bond distances (Å) and angles (°): C12-B1 (1.548), C11-C12 (1.346), C12-C13 (1.526), C14-C15 (1.507), C15-C16 (1.316) Cl1-C16 (1.710), C11-C12-B (117.6), C15-C16-Cl1 (121.1).

- **Crystal data**

Chemical formula	$C_{28}H_{41}BClNO_4$	
Formula Weight	501.88	
Temperature	100(2) K	
Wavelength	1.54178 Å	
Cristal size	0.020 mm x 0.040 mm x 0.200 mm	
Cristal habit	clear colorless block	
Cristal system	Monoclinic	
Space group	P 1 21 1	
Unit cell dimensions	*a* = 11.5921 (8) Å	*α* = 90°
	b = 6.5413 (5) Å	*β* = 104.210 (5)°
	c = 19.4131 (14) Å	*γ* = 90°
Volume	1427.00 (18) $Å^3$	
Z	2	
Density (calculated)	1.168 g/cm^3	
Absorption coefficient	1.43 mm^{-1}	
F(000)	540	

- **Data collection and structure refinement**

Diffractometer	Bruker D8 Venture Photon III-14	
Radiation source	Incoatec IµS 3.0 microfocus sealed tube	
Theta range for data collection	2.35 to 58.92°	
Reflections collected	8297	
Independent reflections	3644 [R(int) = 0.0969]	
Coverage of independent reflections	95.1%	
Absorption correction	Multi-Scan	
Max. and min. transmission	0.9720. and 0.7630	
Structure solution technique	direct methods	
Structure solution program	SHELXT 2018/2 (Sheldrick, 2015)	
Refinement method	Full-matrix least-squares on F^2	
Refinement program	SHELXL-2019/1 (Sheldrick, 2019)	
Function minimized	$\Sigma w(F_o^2 - F_c^2)^2$	
Data / restraints / parameters	3644 / 25 / 359	
Goodness-of-fit on F_2	1.163	
Final R indices	2328 data; I>2σ(I)	R1 = 0.0972, wR2 = 0.2549
	all data	R1 = 0.1583, wR2 = 0.3223
Weighting scheme	$w=1/[\sigma^2(F_o^2)+(0.1895P)^2+0.1571P]$ where $P=(F_o^2+2F_c^2)/3$	
Largest diff. peak and hole	0.295 and -0.539 e $Å^{-3}$	
R.M.S. deviation from mean	0.069 eÅ	

12. DFT Calculations

Theoretical calculations were performed at DFT level of theory using Gaussian16 software[15] at the CESGA facilities.

12.1. Basis set and functional benchmarking to the experimental value

In other to find a suitable computational level for the system, we first carried out the optimization of the structures and single-point energy calculation of the transition states related to the oxidative addition step in the formation of products **(*S*)-3-*Z*,*Z*** and **(*R*)-3-*Z*,*Z*** using two different calculation levels (**Lv1** and **Lv2**).

Table S1. Calculated single-point energies for benchmarking the BS1 to the experimental value

Calculation Level	TS_{OA}-*Z*,*S*	TS_{OA}-*Z*,*R*	$\Delta\Delta G^{\ddagger}_{sol}$ / kcal·mol^{-1}
	G_{SOL} / Hartree	G_{SOL} / Hartree	
**Lv1**	-6163.585434	-6163.57637	5.69
**Lv2**	-6163.604391	-6163.600132	2.67

- **Lv1** consists in the combination of ωB97XD/*def2*-TZVP/*def2*-QZVP (Cu) (scrf=smd, toluene)//ωB97XD/6-31G(d,p).
- **Lv2** consists in the combination of ωB97XD/*def2*-TZVP/*def2*-QZVP (Cu) (scrf=smd, toluene)//ωB97XD/6-31G(d,p)/SDD+f (Cu).

Compared to the experimental derived value $\Delta\Delta G^{\ddagger}$ = 1.9 kcal·mol^{-1} (96:4 er), **Lv2** in which Cu atom is treated apart with a larger basis set and including relativistic effects, provides a more accurate result.

Further exploration was focused on the selection of the single-point corrections with larger basis set. For this, **Lv2**, **Lv3**, **Lv4**, **Lv5** and **Lv6** were evaluated:

Table S2. Calculated single-point energies for benchmarking the single-point correction to the experimental value

Calculation Level	TS_{OA}-*Z*,*S*	TS_{OA}-*Z*,*R*	$\Delta\Delta G^{\ddagger}_{sol}$ / kcal·mol^{-1}
	G_{SOL} / Hartree	G_{SOL} / Hartree	
**Lv2**	-6163.604391	-6163.600132	2.67
**Lv3**	-6163.956308	-6163.950756	3.48
**Lv4**	-6164.827151	-6164.821962	3.26
**Lv5**	-6163.586734	-6163.582406	2.72
**Lv6**	-6163.277975	-6163.27329	2.94

- **Lv2** consists in the combination of ωB97XD/*def2*-TZVP/*def2*-QZVP (Cu) (scrf=smd, toluene)//ωB97XD/6-31G(d,p)/SDD+f (Cu).
- **Lv3** consists in the combination of M06L/*def2*-TZVP/*def2*-QZVP (Cu) (scrf=smd, toluene)//ωB97XD/6-31G(d,p)/SDD+f (Cu).
- **Lv4** consists in the combination of B3LYP-GD3(BJ)/*def2*-TZVP/*def2*-QZVP (Cu) (scrf=smd, toluene)//ωB97XD/6-31G(d,p)/SDD+f (Cu).
- **Lv5** consists in the combination of ωB97XD/*def2*-TZVPP (scrf=smd, toluene)//ωB97XD/6-31G(d,p)/SDD+f (Cu).

- **Lv6** consists in the combination of ωB97XD/6-311++G(d,p)/aug-cc-pVTZ (Cu) (scrf=smd, toluene)//ωB97XD/6-31G(d,p)/SDD+f (Cu).

Other functionals, like M06L (**Lv3**) or B3LYP with D3(BJ) empirical dispersion correction (**Lv4**), led to a moderate overestimation of $\Delta\Delta G^{\ddagger}_{sol}$. In the last case, it can be attributed to overstabilization of the complexes due to empirical dispersion correction.[16] The use of other basis sets in combination with the ωB97XD functional for the single-point calculation – **Lv5** and **Lv6** – led to similar energy differences than the one obtained with **Lv2**. Since our system has a transition metal (Cu), we decided to use the larger basis set combination **Lv2** for better description of its electrons.

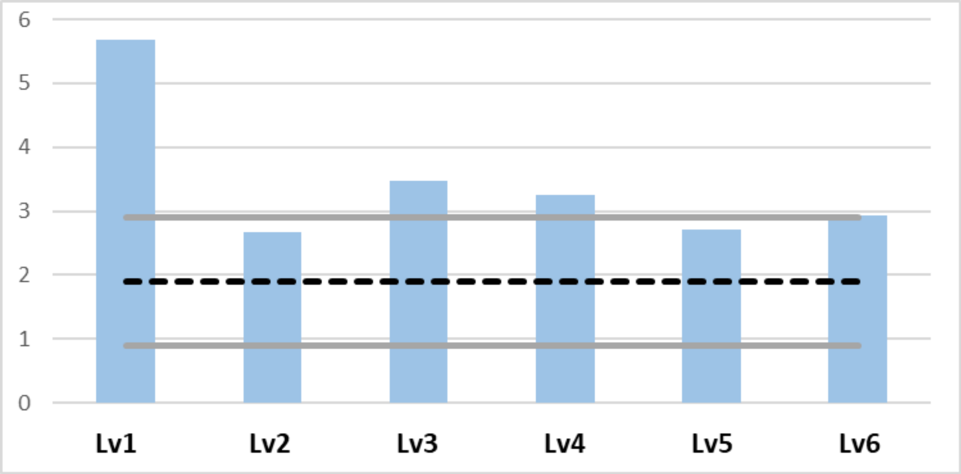

Figure S2. Comparison between calculated single-point energies (in blue) with the experimental derived value (1.9 kcal·mol^{-1}, black dotted line) considering a range of error of ± 1.0 kcal·mol^{-1} (grey lines).

We additionally tested different calculation levels for the transition states **TS$_{OA}$-*Z*,*S*-Li** and **TS$_{OA}$-*Z*,*R*-Li** related to the oxidative addition step in the formation of products **(*S*)-3-*Z*,*Z*** and **(*R*)-3-*Z*,*Z*** when using Li as metal cation. The obtained results are summarized below:

Table S3. Calculated single-point energies for benchmarking the single-point correction to the experimental value for the catalytic system using Li as metal cation

Calculation Level	TS$_{OA}$-*Z*,*S*-Li	TS$_{OA}$-*Z*,*R*-Li	$\Delta\Delta G^{\ddagger}_{sol}$ / kcal·mol^{-1}
	G_{SOL} / Hartree	G_{SOL} / Hartree	
**Lv2**	-5571.199026	-5571.195574	2.1
**Lv3**	-5571.561073	-5571.556745	2.7
**Lv4**	-5572.413165	-5572.409171	2.5
**Lv5**	-5571.182191	-5571.178638	2.2
**Lv6**	-5570.872821	-5570.86936	2.2

Compared to the experimental derived value $\Delta\Delta G^{\ddagger}$ = 0.7 kcal·mol^{-1} (77:23 er), the level of calculation **Lv2** also provides the most accurate result for the system involving the use of Li as metal cation. This result represents a small 1.4 kcal·mol^{-1} overestimation, but this value is within the 1-2 kcal·mol^{-1} error which is typically considered sufficient for reaction energies.

According to our benchmarking studies, the structures of all the intermediates and transition states were optimized using the ωB97XD functional[17] in combination with basis set BS1. BS1 includes the 6-31G(d,p) basis set for the main group elements[18] and the scalar relativistic Stuttgart-Dresden SDD pseudopotential and its associated double-ζ basis set,[19] complemented with a set of *f* polarization functions,[20] for the copper atom. Frequency calculations were performed at the same level to evaluate the zero-point vibrational energy and thermal corrections at 298 K and to confirm the nature of the stationary points, yielding one imaginary frequency for the transition states and none for the minima. It was confirmed that transition states connect with the corresponding intermediates by usual intrinsic reaction coordinate (IRC) calculations and subsequent optimization to minima.

Single-point energies were calculated using the ωB97XD functional within the self-consistent reaction field (SCRF) using the SMD model (toluene)[21] and a larger basis set BS2. BS2 consists in the *def2*-TZVP basis set for the main group elements and the quadruple-ζ *def2*-QZVP basis set for Cu.[22] The final Gibbs energies were obtained adding the thermal and entropic corrections computed at the BS1 level to the electronic energy computed at BS2 level. The reaction profiles were built up in terms of ΔG_{sol}.

All 3D chemical structures were prepared using CYLview20.[23]

12.2. Energy profiles and optimized structures

12.2.1. Energy profile for the pathways associated to the formation of products (*S*)-3-*Z,Z* and (*R*)-3-*Z,Z* using K as metal cation

Two lowest-energy structures (**Ia** and **Ib**), which rapidly interconvert (ΔG_{sol} = 0.5 kcal·mol^{-1}), were found for the σ-allyl-Cu(I) intermediate (Figure S3).

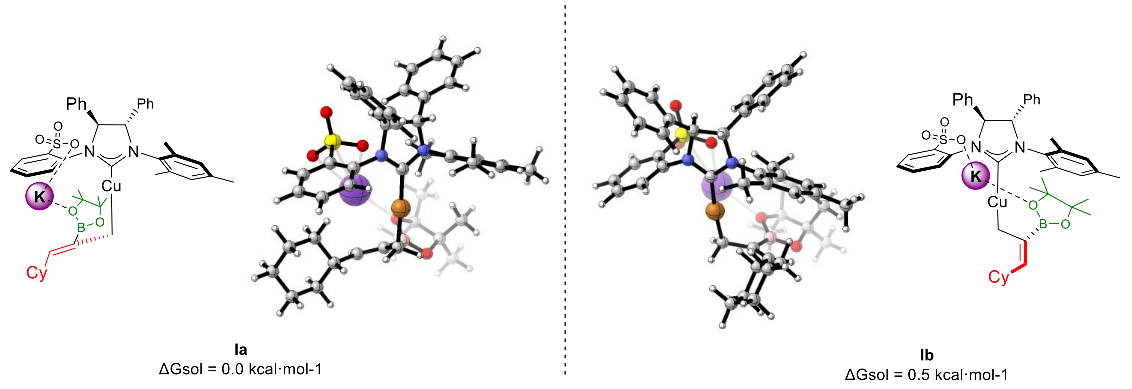

Figure S3. Lowest-energy structures related to σ-allyl-Cu(I) intermediate.

From these intermediates, different modes of coordination between these Cu(I) intermediates and the allylic *gem*-dichloride (coordination from both pro-chiral faces) were investigated in order to find the most favorable pathway. The energies for the different pathways associated to the formation of both enantiomers of product **3-*Z,Z*** are shown in Figure S4 and the related structures in Figures S5-S8.

Comparison of the activation energy barriers for the oxidative addition of the allylic gem-dichloride into the allyl-Cu(I) intermediate ($\Delta G^{‡}$=23.0 kcal·mol^{-1}) and reductive elimination from π-allyl-Cu(III) intermediate ($\Delta G^{‡}$=6.8 kcal·mol^{-1}) in the pathway associated with the formation of product **(*S*)-3-*Z,Z*** (black pathway) revealed that the former is the stereodetermining step. This was assumed for the other pathways, and we thus focused on the study of the different transition states related to this step.

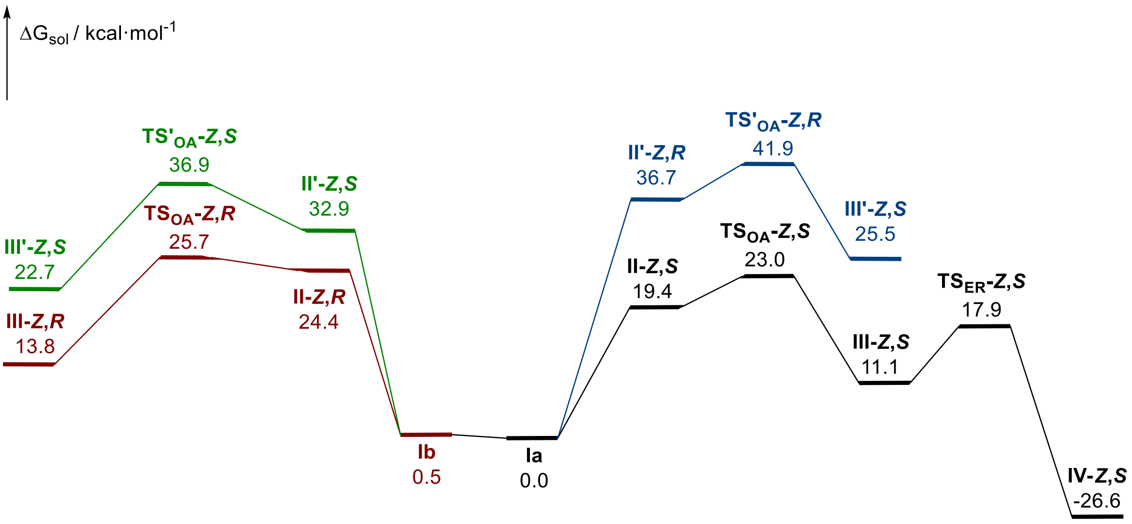

Figure S4. Free energy profile computed at the ωB97XD/*def2*-TZVP/*def2*-QZVP (Cu) (scrf=smd, toluene) //ωB97XD/6-31G(d,p)/SDD+f (Cu) level with K as metal cation for the pathways associated to the formation of both enantiomers of product **3-*Z,Z***. Energies are relative to complex **Ia** combined with those of the relevant substrates.

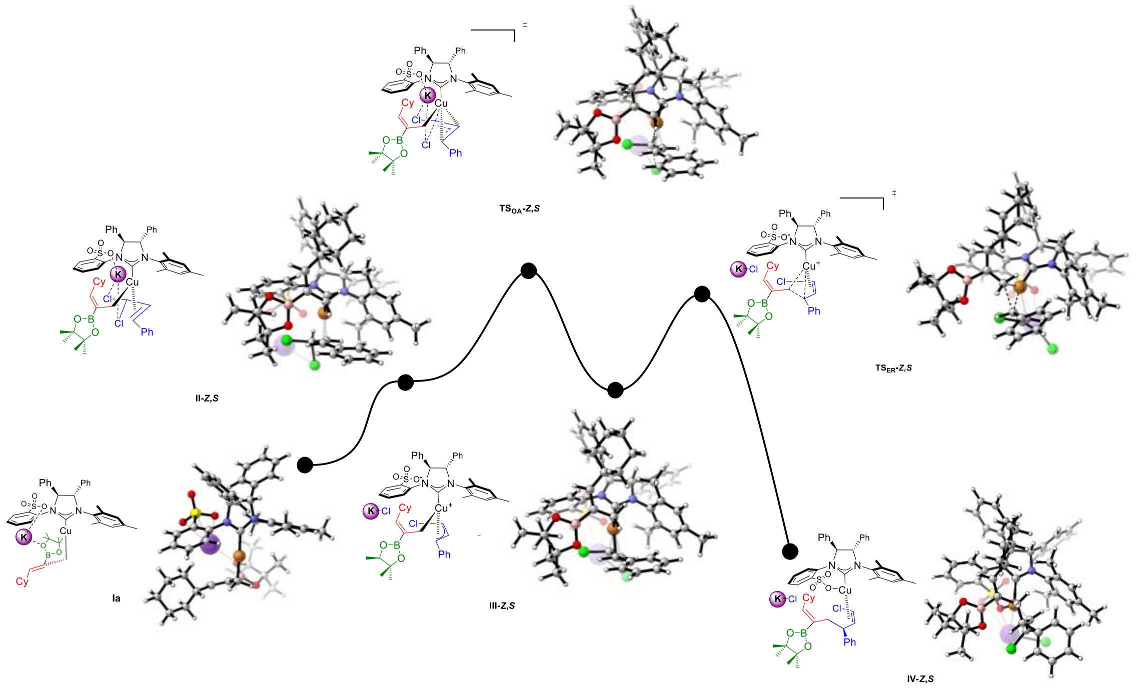

Figure S5. Computed structures related to the oxidative addition and reductive elimination steps associated with the formation of **(*S*)-3-*Z,Z*** from **Ia** with K as metal cation (black pathway).

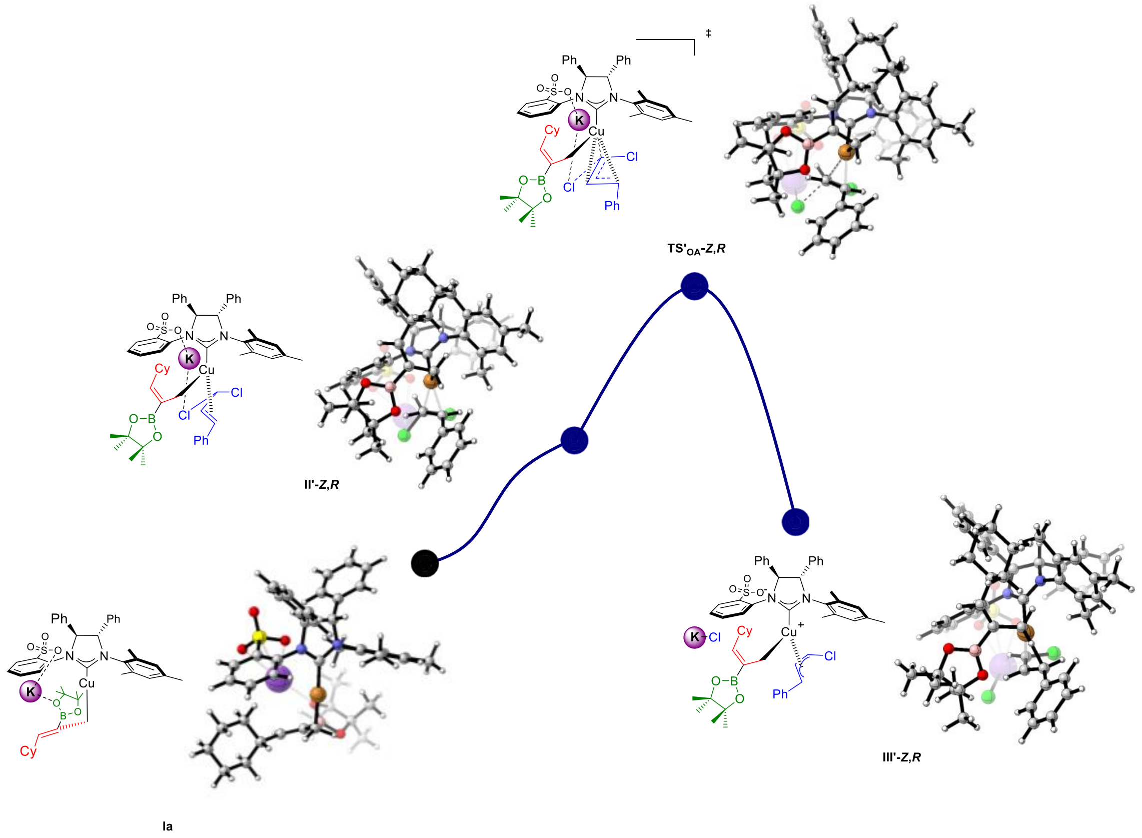

Figure S6. Computed structures related to the oxidative addition step associated with the formation of **(*R*)-3-*Z,Z*** from **Ia** with K as metal cation (blue pathway).

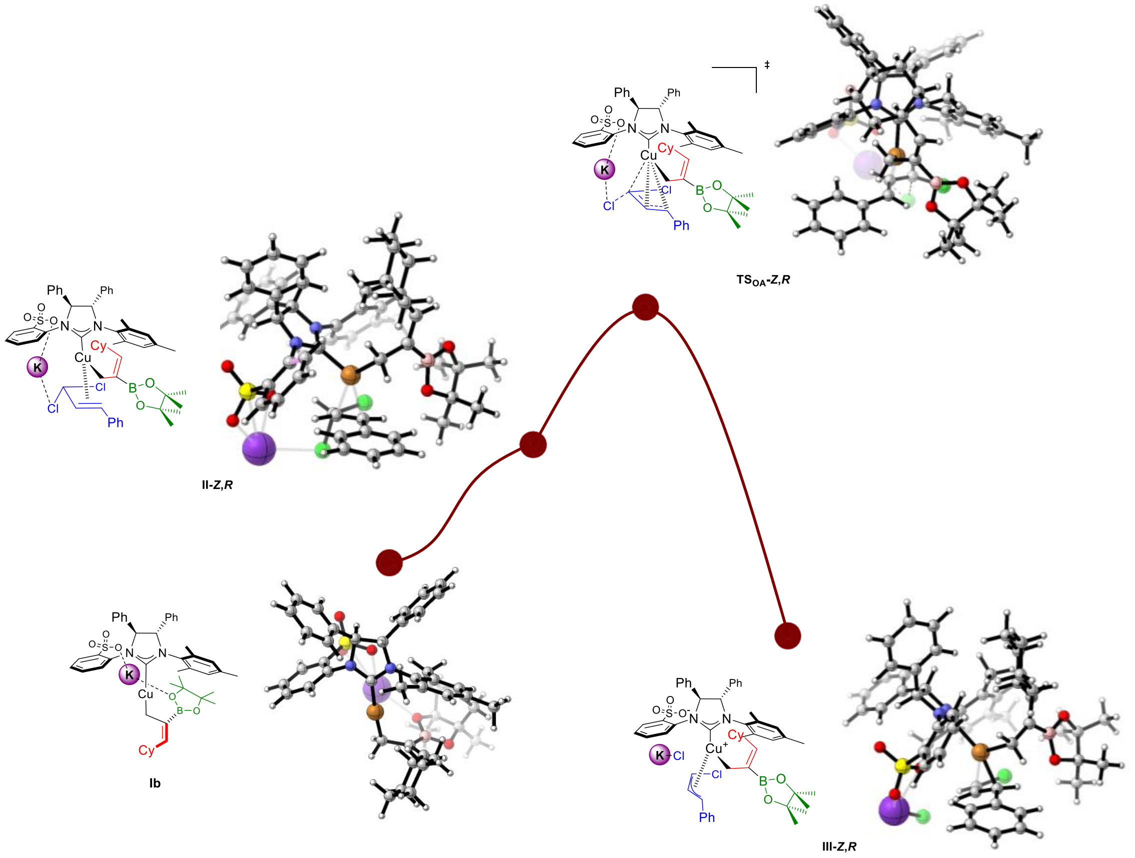

Figure S7. Computed structures related to the oxidative addition step associated with the formation of **(*R*)-3-*Z,Z*** from **Ib** with K as metal cation (dark red pathway).

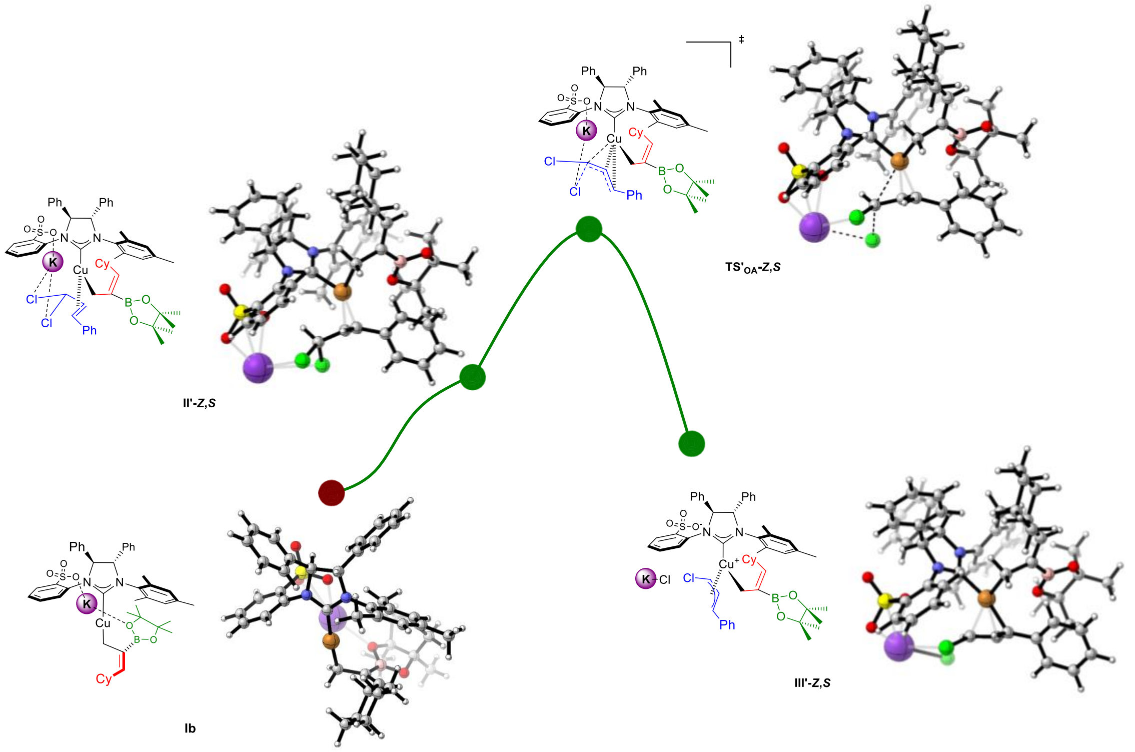

Figure S8. Computed structures related to the oxidative addition step associated with the formation of **(*S*)-3-*Z,Z*** from **Ib** with K as metal cation (green pathway).

12.2.2. Energy profile for the pathways associated to the formation of products (*S*)-3-*Z,E* and (*R*)-3-*Z,E* using K as metal cation

The energies for the different pathways associated to the formation of both enantiomers of product **3-*Z,E*** are shown in Figure S9 and the related structures in Figures S10 and S11.

Only the two most favorable pathways for the formation of both enantiomers are shown (i.e. formation of the *S* enantiomer from **Ia** and formation of the *R* enantiomer from **Ib**).

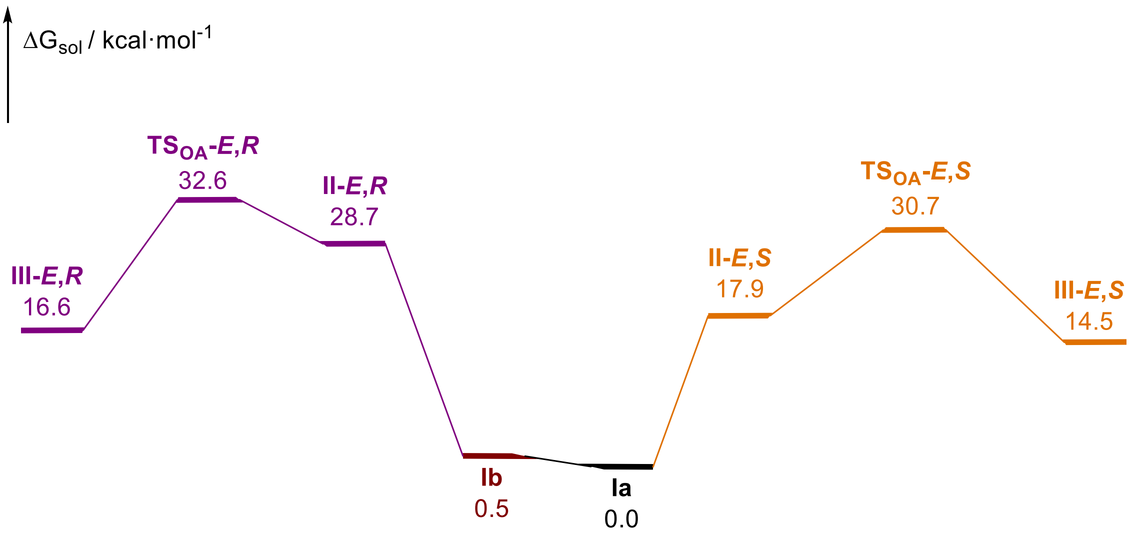

Figure S9. Free energy profile computed at the ωB97XD/*def2*-TZVP/*def2*-QZVP (Cu) (scrf=smd, toluene) //ωB97XD/6-31G(d,p)/SDD+f (Cu) level with K as metal cation for the pathways associated to the formation of both enantiomers of product **3-*Z,E***. Energies are relative to complex **Ia** combined with those of the relevant substrates.

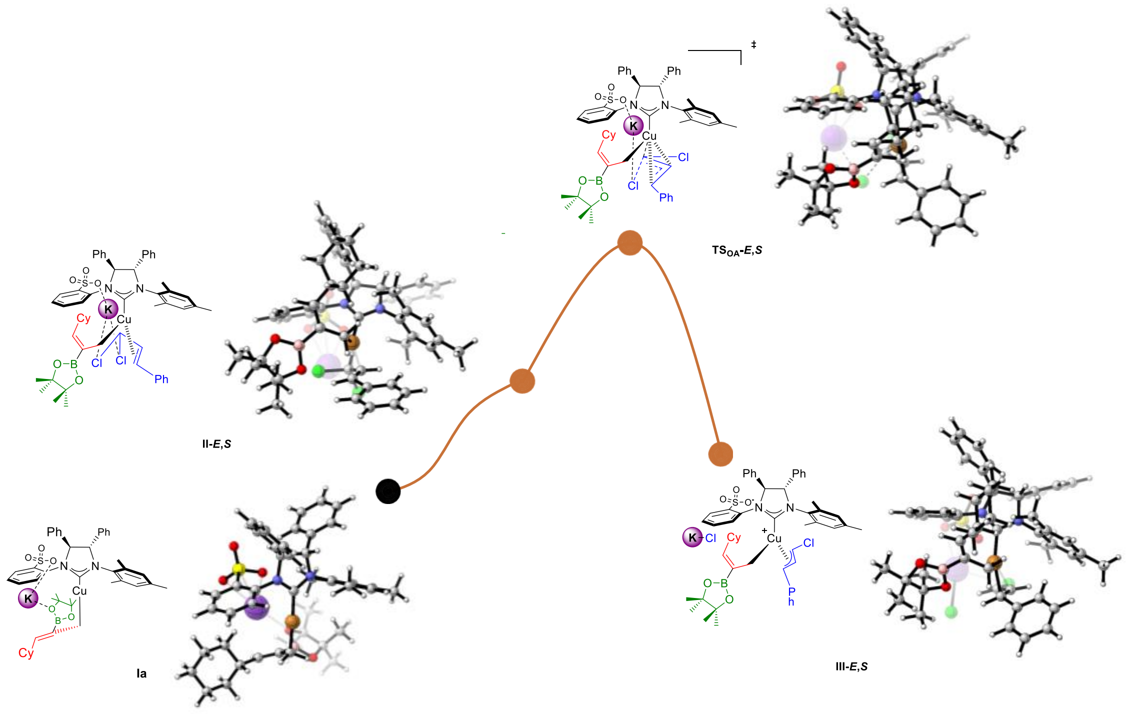

Figure S10. Computed structures related to the oxidative addition step associated with the formation of **(*S*)-3-*Z,E*** from **Ia** with K as metal cation (orange pathway).

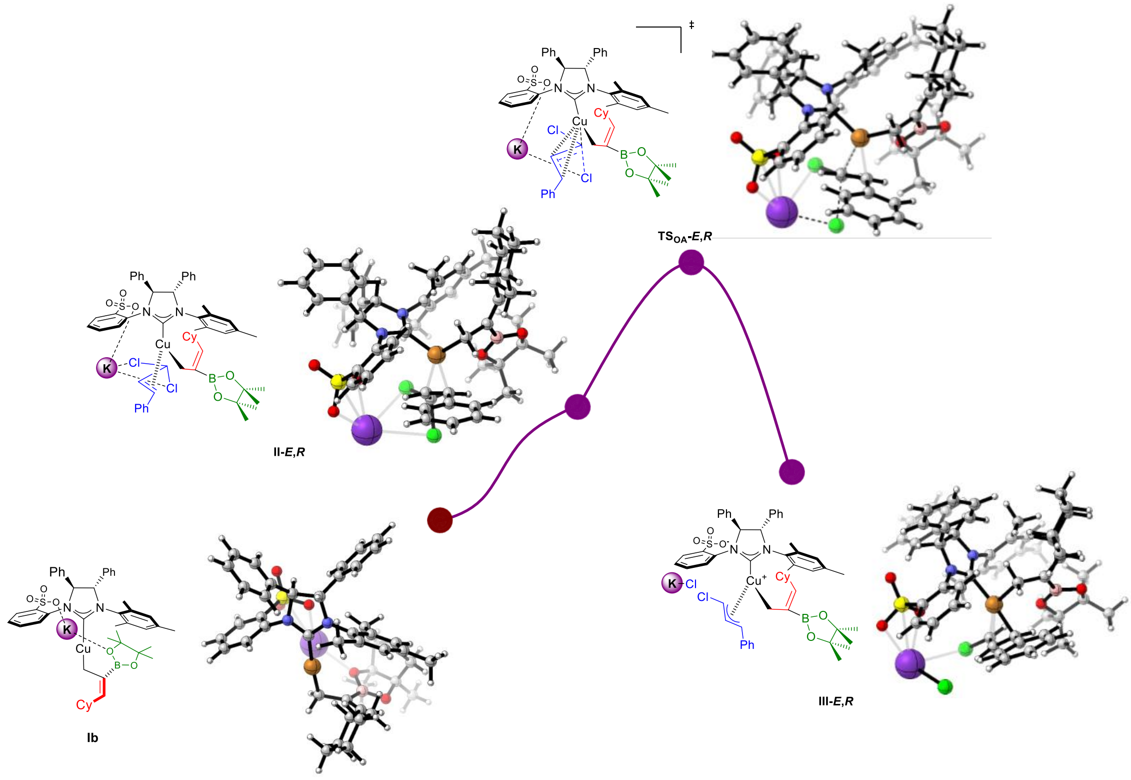

Figure S11. Computed structures related to the oxidative addition step associated with the formation of **(*R*)-3-*Z,E*** from **Ib** with K as metal cation (purple pathway).

12.2.3. Energy profile for the pathways associated to the formation of products (*S*)-3-*Z,Z* and (*R*)-3-*Z,Z* using Li as metal cation

The energies for the different pathways associated to the formation of both enantiomers of product **3-*Z,Z*** employing Li as metal cation are shown in Figure S12 and the related structures in Figures S13 and S14.

Only the two most favorable pathways for the formation of both enantiomers are shown (i.e. formation of the *S* enantiomer from **Ia-Li** and formation of the *R* enantiomer from **Ib-Li**).

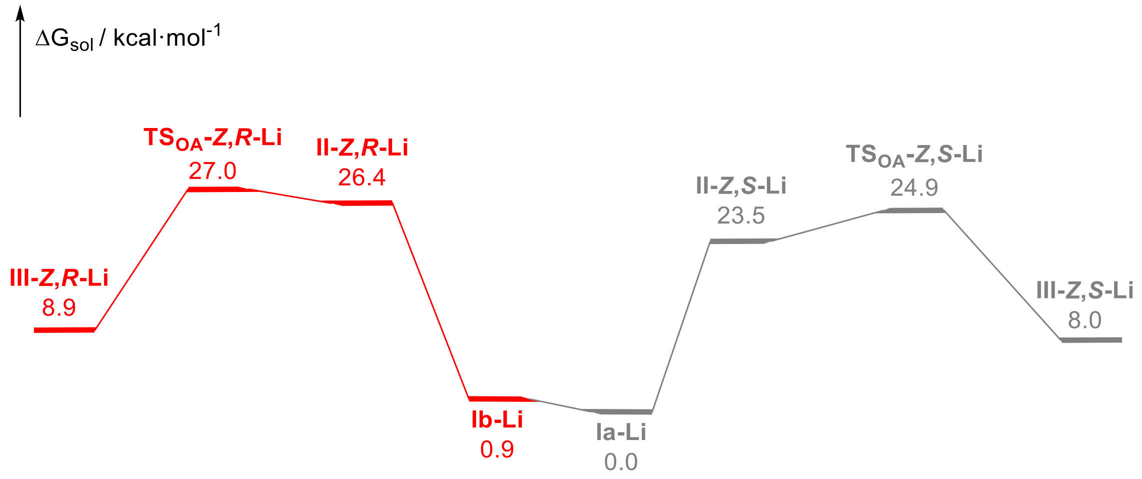

Figure S12. Free energy profile computed at the ωB97XD/*def2*-TZVP/*def2*-QZVP (Cu) (scrf=smd, toluene) //ωB97XD/6-31G(d,p)/SDD+f (Cu) level with Li as metal cation for the pathways associated to the formation of both enantiomers of product **3-*Z,Z***. Energies are relative to complex **I** combined with those of the relevant substrates.

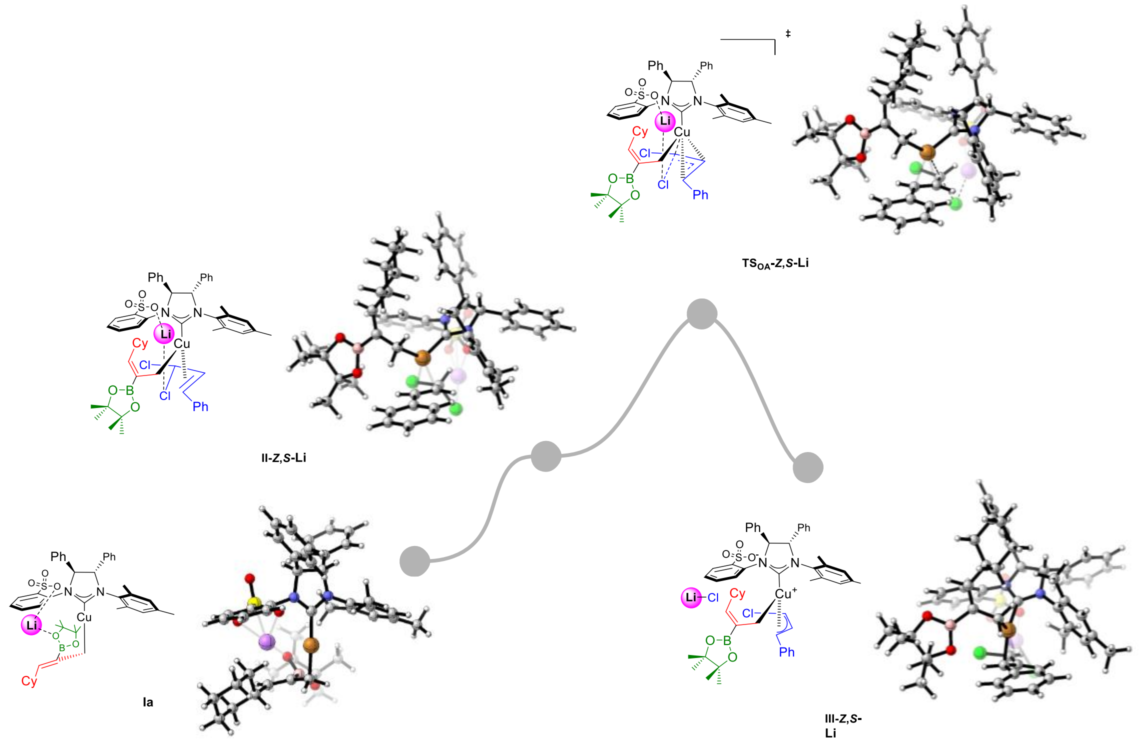

Figure S13. Computed structures related to the oxidative addition step associated with the formation of **(*S*)-3-*Z,Z*** from **Ia-Li** (grey pathway).

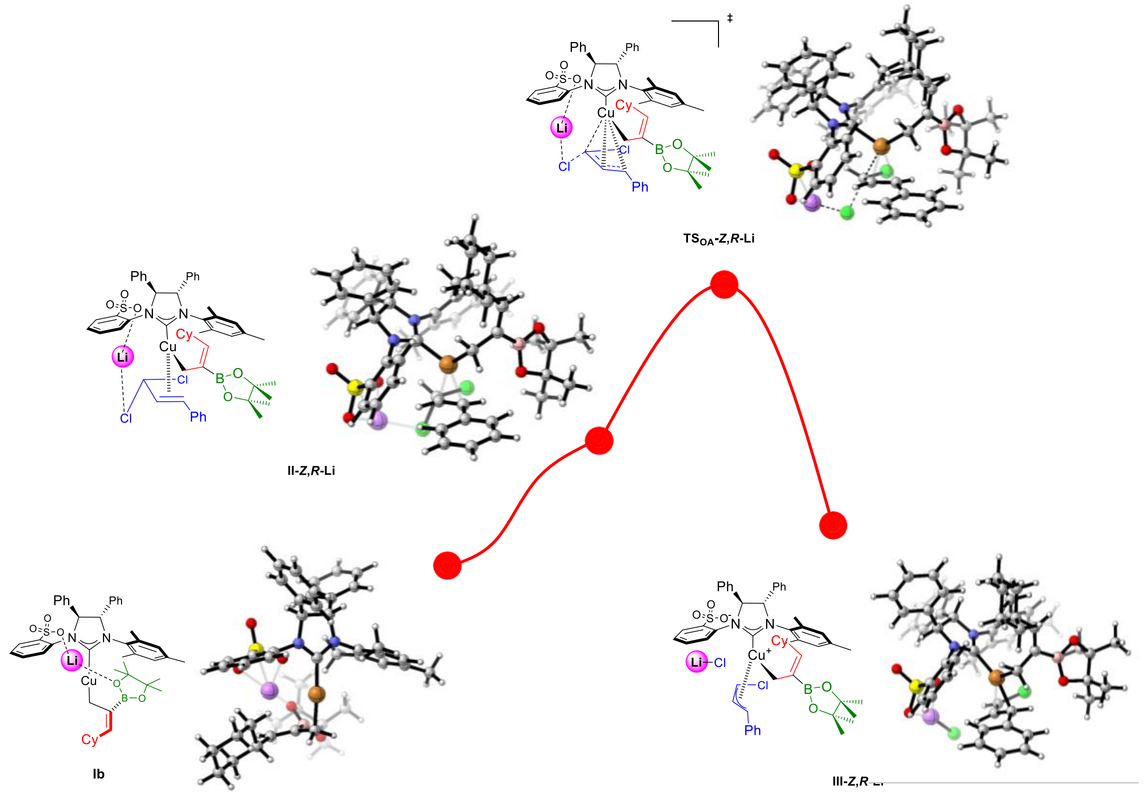

Figure S14. Computed structures related to the oxidative addition step associated with the formation of **(*R*)-3-*Z,Z*** from **Ib-Li** (red pathway).

12.3. Non-Covalent Interactions (NCI) plots

Non-Covalent Interaction (NCI) analysis was performed using NCI theory[24] embedded in Multiwfn 3.8 software.[25] Attractive forces (blue) and weak attractive Van der Waals interactions (green) were plotted with at iso-surfaces of s(r) = 0.40. Repulsive forces (red) and weak repulsive Van der Waals interactions (brownish green) were plotted with at iso-surfaces of s(r) = 0.30. Color code corresponds with sign(λ2)ρ in a.u.. NCI surfaces were plotted using VMD software.[26]

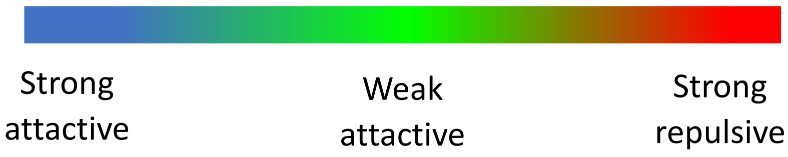

12.3.1. NCI plots for TS_{OA}-*Z,S* and TS_{OA}-*Z,R*

Attractive interactions.

Comparison between the NCI plots for both transition state structures (Figure S15) revealed some differences which mainly arise from the different modes of cation-bridge ligand-substrate interaction (blue circles). While **TS_{OA}-*Z,S*** displays two Cl···K attractive isosurfaces, **TS_{OA}-*Z,R*** only shows one Cl···K interaction due to its geometrical conformation.

Furthermore, we could notice a strong attractive isosurface that reveals an O···H interaction, shifted to hydrogen bond type (red circle), between the sulfonate unit and the H atom from the Cα of the allylic *gem*-dichloride, that is slightly stronger in **TS_{OA}-*Z,S*** than in **TS_{OA}-*Z,R***.

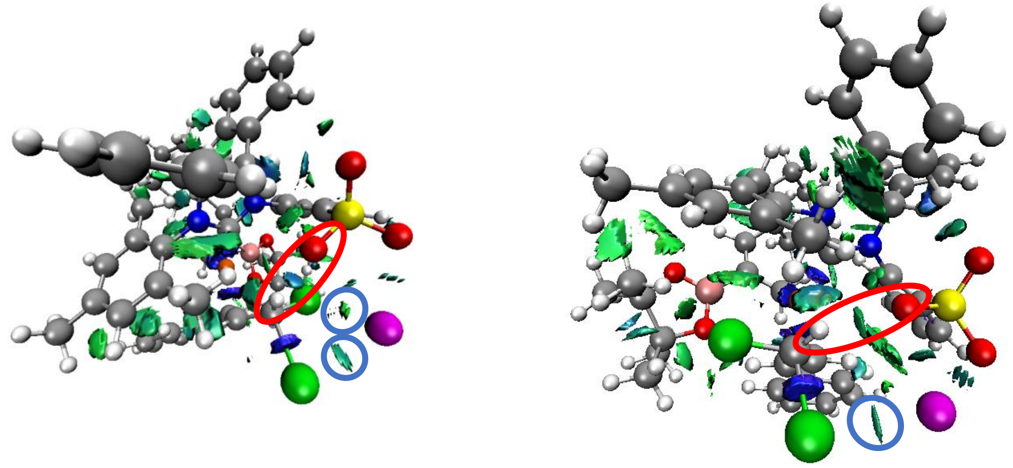

TS_{OA}-*Z,S* **TS_{OA}-*Z,R***

Figure S15. NCI plots showing attractive interactions for **TS_{OA}-*Z,S*** and **TS_{OA}-*Z,R***.

Repulsive interactions.

No significant differences were found in terms of repulsive interactions after comparing the NCI plots of both transition states.

Three different views of these structures are given in Figures S16-S18 for a better observation.

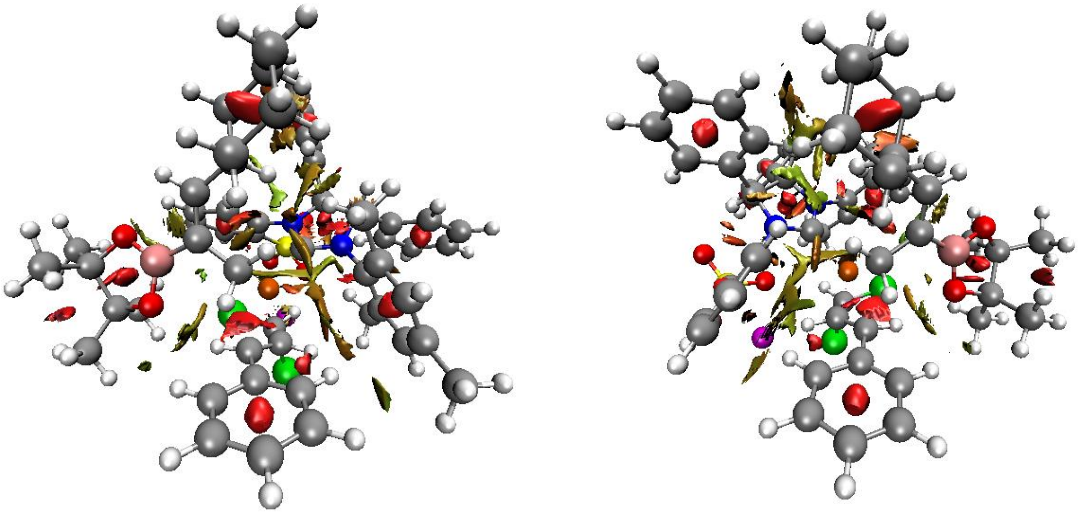

TS$_{OA}$-*Z,S* **TS$_{OA}$-*Z,R***

Figure S16. NCI plots showing repulsive interactions for **TS$_{OA}$-*Z,S*** and **TS$_{OA}$-*Z,R***: *Front view*

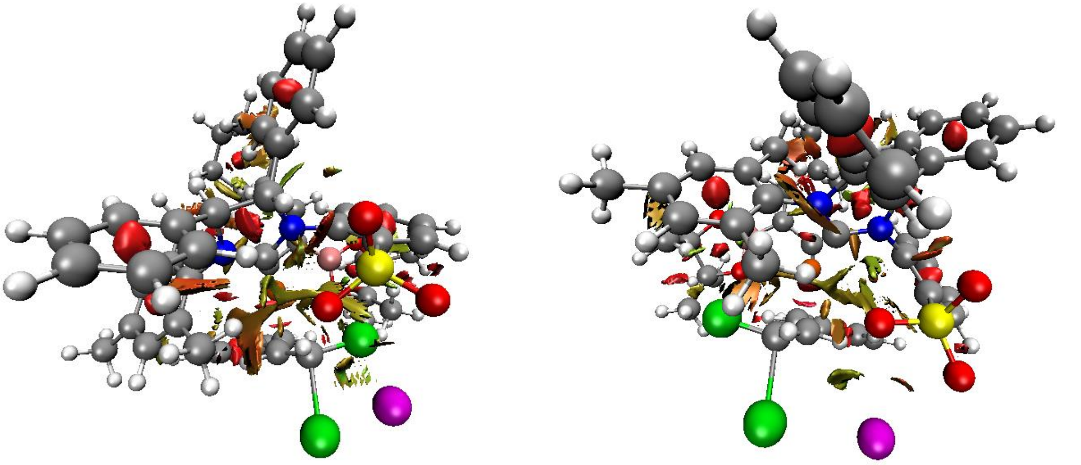

TS$_{OA}$-*Z,S* **TS$_{OA}$-*Z,R***

Figure S17. NCI plots showing repulsive interactions for **TS$_{OA}$-*Z,S*** and **TS$_{OA}$-*Z,R***: *Sulfonate-side view*

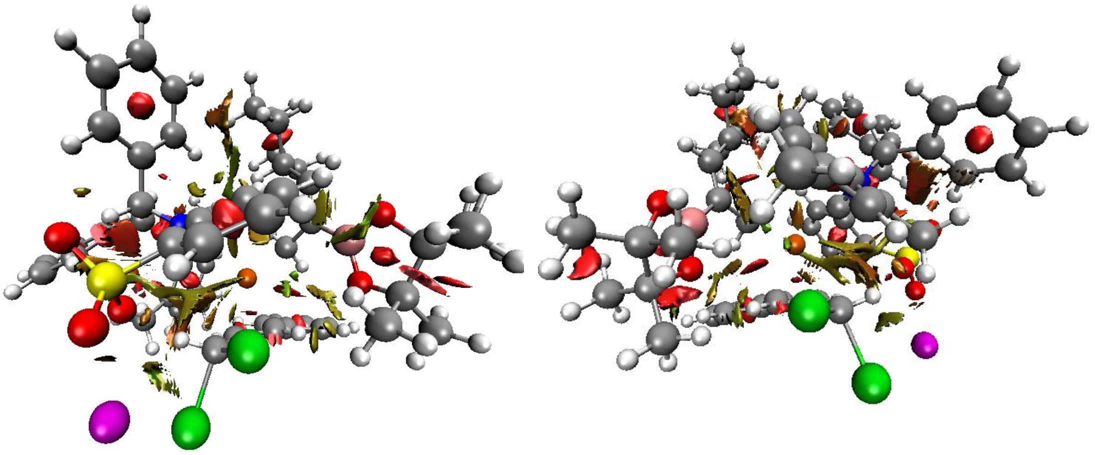

TS$_{OA}$-*Z,S* **TS$_{OA}$-*Z,R***

Figure S18. NCI plots showing repulsive interactions for **TS$_{OA}$-*Z,S*** and **TS$_{OA}$-*Z,R***: *Bpin-side view*

12.3.2. NCI plots for TS$_{OA}$-*Z,S*-Li and TS$_{OA}$-*Z,R*-Li

Attractive interactions.

Comparison between both NCI plots revealed no major differences in terms of attractive interactions (Figure S19). In contrast to the K system, **TS$_{OA}$-*Z,S*-Li** only features one Cl···K interaction, thus showing a similar scenario than **TS$_{OA}$-*Z,R*-Li**.

Nevertheless, we could notice that the O···H interaction established between the sulfonate unit and the H from the Cα of the allylic *gem*-dichloride (red circle) is still stronger in **TS$_{OA}$-*Z,S*-Li** than in **TS$_{OA}$-*Z,R*-Li** (darker blue interaction in **TS$_{OA}$-*Z,S*-Li**). This could explain the preferential formation of product **(*S*)-3-*Z,Z*** enantiomer albeit with a lower enantiomeric ratio.

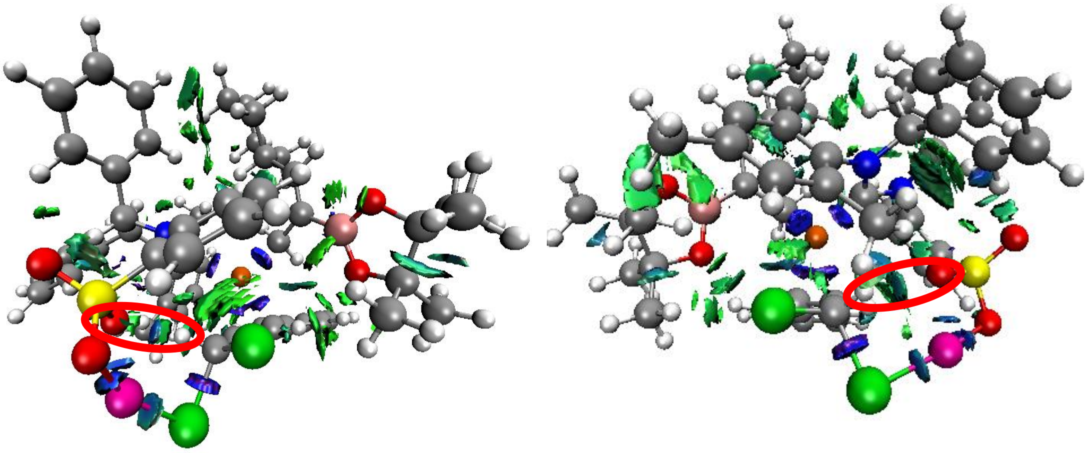

TS$_{OA}$-*Z,S*-Li **TS$_{OA}$-*Z,R*-Li**

Figure S19. NCI plots showing attractive interactions for **TS$_{OA}$-*Z,S*-Li** and **TS$_{OA}$-*Z,R*-Li**.

Repulsive interactions.

No significant differences were found in terms of repulsive interactions after comparing the NCI plots of both transition states (see Figures S20 and S21).

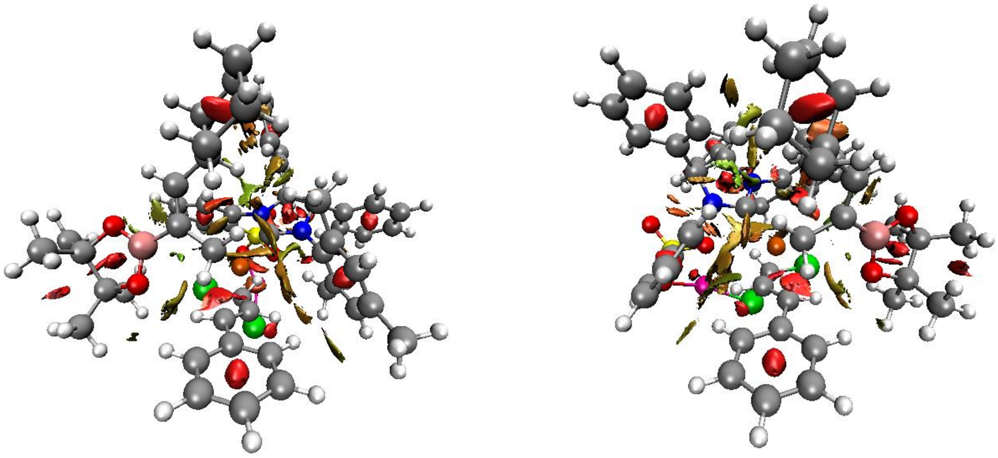

TS$_{OA}$-*Z*,*S*-Li **TS$_{OA}$-*Z*,*R*-Li**

Figure S20. NCI plots showing repulsive interactions for **TS$_{OA}$-*Z*,*S*-Li** and **TS$_{OA}$-*Z*,*R*-Li**: *Front view*

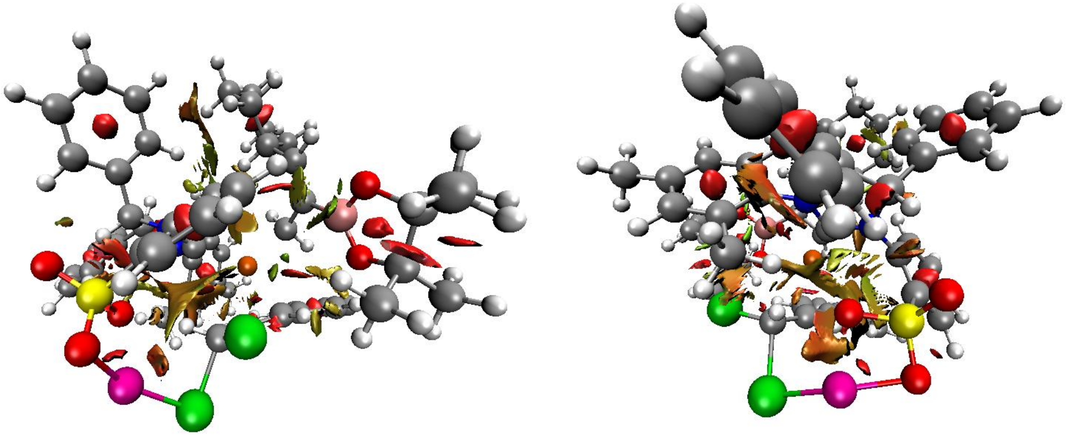

TS$_{OA}$-*Z*,*S*-Li **TS$_{OA}$-*Z*,*R*-Li**

Figure S21. NCI plots showing repulsive interactions for **TS$_{OA}$-*Z*,*S*-Li** and **TS$_{OA}$-*Z*,*R*-Li**: *Sulfonate side view*

12.3.3. NCI plots for TS$_{OA}$-*Z*,*S* and TS$_{OA}$-*Z*,S-Li

Attractive interactions.

Figure S22 shows the comparison between the NCI plots showing the attractive interactions in stereodetermining oxidative addition transition states that lead to the major product **(*S*)-3-*Z*,*Z*** using either K or Li cation. The main difference in terms of attractive interactions between these transitions states lies on the absence of the second chlorine-metal cation interaction in **TS$_{OA}$-*Z*,*S*-**

Li. This is presumably due to the smaller size of the Li cation that hampers its interaction with the second chlorine atom.

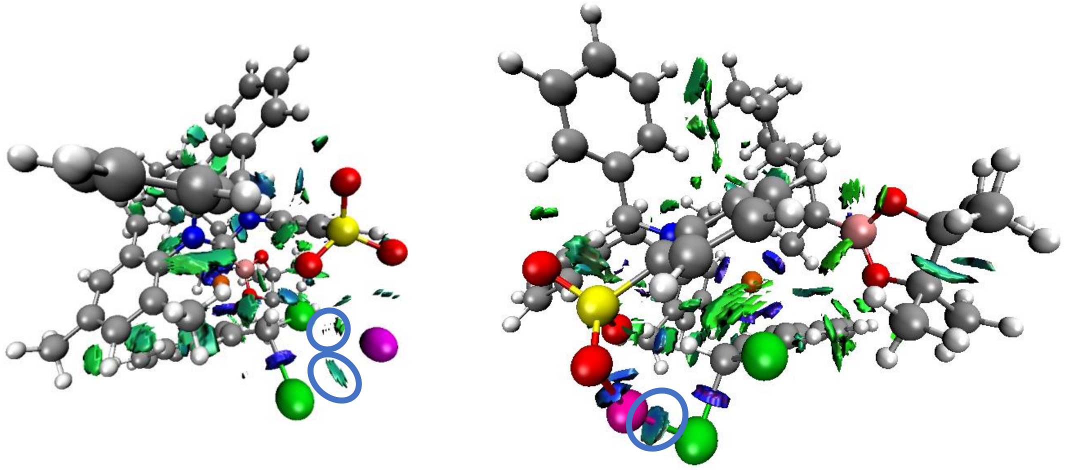

TS$_{OA}$-*Z*,*S* **TS$_{OA}$-*Z*,*S*-Li**

Figure S22. NCI plots showing attractive interactions for **TS$_{OA}$-*Z*,*S*** and **TS$_{OA}$-*Z*,*S*-Li**.

Repulsive interactions.

A repulsive interaction between the Cα of the allylic *gem*-dichloride and the metal cation was observed in both cases (red circle). This interaction is stronger in the **TS$_{OA}$-*Z*,*S*-Li** (darker brown isosurface). This is likely due to the smaller size of the Li cation, which has to get closer to the Cα of the allylic *gem*-dichloride in order to interact with the chlorine atom.

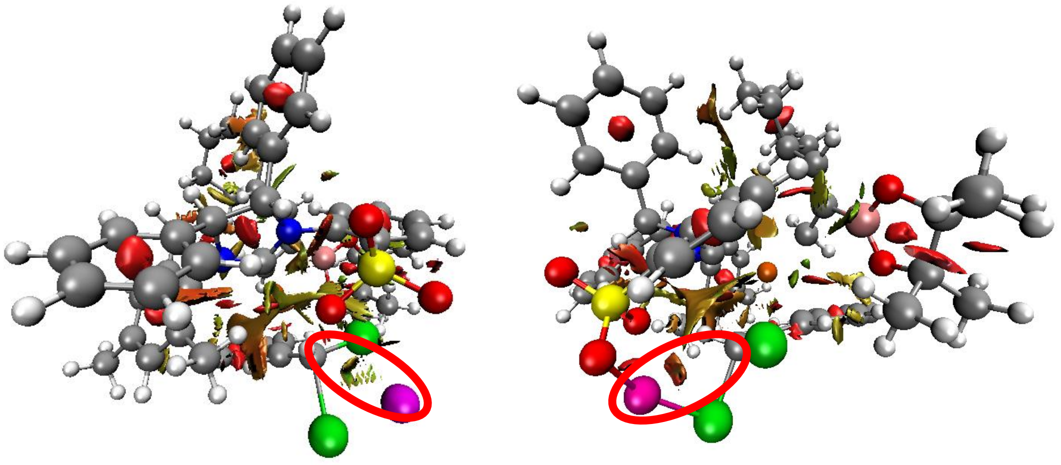

TS$_{OA}$-*Z*,*S* **TS$_{OA}$-*Z*,*S*-Li**

Figure S23. NCI plots showing repulsive interactions for **TS$_{OA}$-*Z*,*S*** and **TS$_{OA}$-*Z*,*S*-Li**.

The absence of the second stabilizing Cl···M interaction (see Figure S22) and the presence of this stronger repulsive interaction between the metal cation and the Cα of the allylic *gem*-dichloride (Figure S23) might explain the increase in the activation energy barrier observed for **TS$_{OA}$-*Z*,*S*-Li** and thus the decrease both in efficiency and enantioselelectivity observed when LiO^{t}Bu is used instead of KO^{t}Bu.

12.3.4. NCI plots for TS$_{OA}$-*Z,S* and TS$_{OA}$-*E,S*

Attractive interactions.

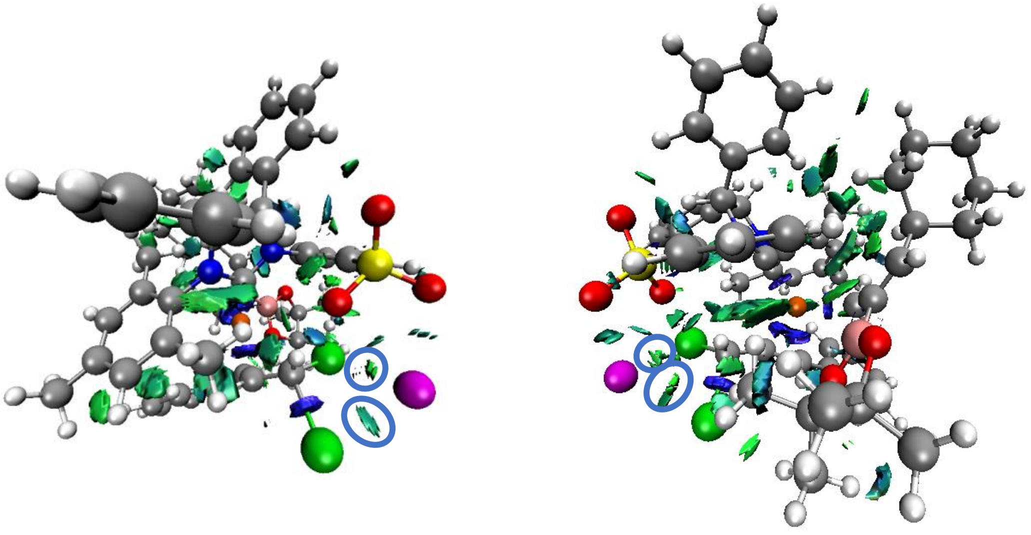

TS$_{OA}$-*Z,S* **TS$_{OA}$-*E,S***

Figure S24. NCI plots showing attractive interactions for **TS$_{OA}$-*Z,S*** and **TS$_{OA}$-*E,S***.

Comparison between NCI plots of both transition states revealed no major differences in terms of attractive interactions (Figure S24). Notably, both transition states feature the two stabilizing Cl···K interactions (blue circles).

Repulsive interactions.

Comparison of the NCI plots showing repulsive interactions for both transition states (Figure S25) shows that **TS$_{OA}$-*E,S*** features a strong repulsive interaction engendered between the Bpin unit and the arylsulfonate ring (red circle). This repulsive interaction is not present in **TS$_{OA}$-*Z,S***.

Additionally, the repulsive interactions between the cyclohexyl moiety and the NHC's mesityl and phenyl rings are stronger (bigger and darker isosurfaces) in **TS$_{OA}$-*E,S*** (green circle).

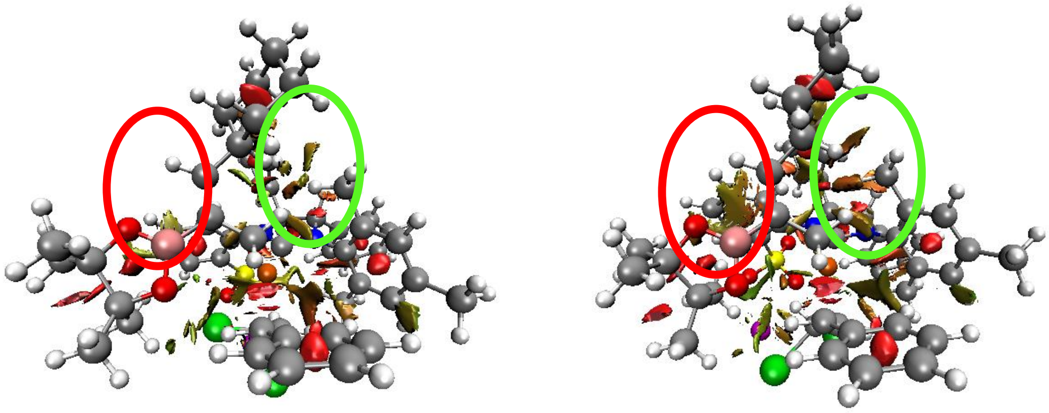

TS$_{OA}$-*Z,S* **TS$_{OA}$-*E,S***

Figure S25. NCI plots showing repulsive interactions for **TS$_{OA}$-*Z,S*** and **TS$_{OA}$-*E,S***.

The increase on the repulsive forces in **TS$_{OA}$-*E*,*S*** is likely promoted by the structural reorganization that the system has to adopt in order to keep the stabilizing double potassium-bridge ligand-substrate interaction. Since *E* diastereomer formation implies a conformational change in Cα of the allylic *gem*-dichloride, the arylsulfonate moiety is forced to rotate to keep both Cl···K interactions. This structural reorganization pushes the *N*-arylsulfonate ring closer to the Bpin unit thus generating a new repulsive interaction (red circle). Consequently, the allyl-Bpin structure adopts a new spatial disposition which enhances the repulsive interactions between the cyclohexyl group and the NHC's phenyl and mesityl rings (green circle).

12.4. Cartesian coordinates

2

Electronic Energy BS1 = -1268.00997593 Hartree

Electronic Energy BS2 = -1268.19650959 Hartree

Zero-point Energy Correction = 0.145610 Hartree

Thermal Correction to Enthalpy = 0.156715 Hartree

Thermal Correction to Free Energy = 0.106926 Hartree

Chemical symbol X, Y, Z

C	-3.400034	0.914075	1.050782
C	-2.052944	0.764025	0.751543
C	-1.640004	-0.117699	-0.255836
C	-2.616791	-0.827868	-0.962803
C	-3.965984	-0.679175	-0.662858
C	-4.361496	0.191424	0.347207
H	-3.702823	1.603437	1.832261
H	-1.319225	1.350143	1.295538
H	-2.312473	-1.509529	-1.752441
H	-4.707819	-1.242237	-1.219712
H	-5.413894	0.312857	0.582072
C	-0.225644	-0.323529	-0.601323
C	0.832782	0.059490	0.116738
H	-0.049828	-0.857358	-1.535149
H	0.738321	0.570473	1.070423
C	2.222617	-0.199599	-0.340582
H	2.253290	-0.721860	-1.295089
Cl	3.117197	1.343819	-0.578312
Cl	3.103946	-1.237512	0.835070

Ib

Electronic Energy BS1 = -3452.19485477 Hartree

Electronic Energy BS2 = -4896.36809522 Hartree

Zero-point Energy Correction = 0.910576 Hartree

Thermal Correction to Enthalpy = 0.966311 Hartree

Thermal Correction to Free Energy = 0.817319 Hartree

Chemical symbol X, Y, Z

H	3.378776	-0.744429	1.017505
C	2.500129	1.241858	1.209235

H	2.935032	2.229924	1.021075	C	4.869674	1.753532	-0.764142
C	1.149248	0.408706	-0.537556	C	7.013899	0.055491	-0.259886
C	2.683648	-1.080238	-1.688501	H	5.568653	-1.212259	0.723647
C	3.009609	-0.638286	-2.968956	C	6.139480	2.086204	-1.220221
C	2.660696	-2.451306	-1.408170	H	4.029089	2.409756	-0.978960
C	3.309687	-1.547826	-3.975137	C	7.215831	1.237921	-0.964135
H	3.023136	0.430208	-3.154545	H	7.846624	-0.613510	-0.067629
C	2.970230	-3.357348	-2.422289	H	6.291393	3.005330	-1.777745
C	3.293122	-2.911426	-3.698612	H	8.208103	1.497749	-1.319637
H	3.558870	-1.191351	-4.969127	N	2.377896	-0.097995	-0.705081
H	2.934507	-4.417108	-2.195123	N	1.171684	1.182909	0.553315
H	3.529457	-3.630306	-4.476377	C	0.075618	1.964482	1.028514
S	2.269827	-3.145791	0.214385	C	0.105854	3.354692	0.836688
O	1.622281	-4.436633	-0.113792	C	-0.978007	1.350914	1.717760
O	1.275076	-2.210955	0.816152	C	-0.913968	4.125051	1.386834
O	3.533007	-3.210794	0.949425	C	-1.988702	2.163394	2.233317
Cu	-0.413682	-0.119018	-1.522643	C	-1.969616	3.547246	2.091945
C	4.664242	0.574161	-0.044567	H	2.058637	-1.081952	2.635796
C	5.740387	-0.278098	0.195321	H	1.858386	-1.313226	5.080731

C	2.166273	-0.206428	3.270829	H	-0.899279	5.202143	1.236359
C	2.042607	-0.333837	4.650267	H	-2.812736	1.693746	2.766033
C	2.411588	1.047634	2.705523	B	-3.961914	-1.233001	-0.517641
C	2.157619	0.782043	5.476133	O	-5.185484	-1.001769	0.062649
H	2.061742	0.675913	6.552368	O	-3.397820	-2.416738	-0.036209
C	2.525155	2.161160	3.537482	C	-5.327595	-1.888226	1.182270
C	2.398934	2.033036	4.917232	C	-4.381616	-3.075472	0.791096
H	2.710056	3.139392	3.100413	C	-5.066963	-4.128251	-0.079744
H	2.490420	2.908185	5.552829	H	-4.314207	-4.818467	-0.472179
C	-2.071017	-0.763177	-2.315276	H	-5.795479	-4.709236	0.492178
C	-3.237162	-0.284969	-1.499473	H	-5.576118	-3.663481	-0.928019
C	1.200420	4.015785	0.038680	C	-3.670308	-3.735172	1.965351
H	0.847552	4.959886	-0.383270	H	-3.062443	-4.577758	1.617971
H	2.076439	4.241791	0.658065	H	-3.019759	-3.029142	2.486494
H	1.530381	3.377063	-0.785726	H	-4.394984	-4.132947	2.682009
C	-1.068310	-0.141315	1.894165	C	-6.794059	-2.268766	1.331465
H	-1.453099	-0.384001	2.889487	H	-7.200566	-2.668406	0.401187
H	-1.762643	-0.555884	1.151944	H	-6.921244	-3.014428	2.122880
H	-0.107359	-0.638165	1.763668	H	-7.375188	-1.383467	1.601001

C	-4.853727	-1.125740	2.422276
H	-3.799509	-0.849125	2.337601
H	-5.436315	-0.205285	2.508200
H	-4.991501	-1.711016	3.335873
C	3.289002	0.170097	0.425547
H	-2.078039	-0.308125	-3.315038
H	-2.145635	-1.845243	-2.496747
C	-3.638706	1.005501	-1.444245
H	-4.488389	1.264730	-0.812606
C	-2.957688	2.143252	-2.146537
C	-3.433436	2.346366	-3.596476
C	-3.100402	3.451881	-1.361856
H	-1.880518	1.902176	-2.195403
C	-2.658832	3.481706	-4.273513
H	-4.506471	2.584226	-3.587698
H	-3.321809	1.415572	-4.162241
C	-2.320029	4.588801	-2.021313
H	-4.166457	3.720508	-1.308635
H	-2.755697	3.298146	-0.336521
C	-2.756402	4.787303	-3.476471
H	-3.023845	3.634657	-5.295418
H	-1.602469	3.189125	-4.356244
H	-2.446288	5.518491	-1.453800
H	-1.248257	4.344929	-1.991022
H	-2.155445	5.569422	-3.954189
H	-3.798162	5.136886	-3.490520
K	-0.852057	-3.173365	-0.383108
C	-3.098356	4.396892	2.614459
H	-3.614237	3.909785	3.446019
H	-2.740105	5.371446	2.957388
H	-3.836664	4.575617	1.824147

Ia

Electronic Energy BS1 = -3452.19704694 Hartree

Electronic Energy BS2 = -4896.36900178 Hartree

Zero-point Energy Correction = 0.910814 Hartree

Thermal Correction to Enthalpy = 0.966595 Hartree

Thermal Correction to Free Energy = 0.817489 Hartree

Chemical symbol X, Y, Z				Cu	-0.638726	-0.130769	-1.523694
H	3.128044	-0.823245	1.181642	C	4.394605	-1.394080	-0.427166
C	3.236020	0.898119	-0.153710	C	5.085267	-2.260322	0.417637
H	3.936609	1.119556	-0.967777	C	4.773108	-1.296179	-1.768128
C	1.171185	0.013737	-0.881676	C	6.157360	-3.006138	-0.066976
C	1.497016	-2.365417	-0.469132	H	4.766213	-2.358562	1.451824
C	1.551065	-3.140171	-1.625529	C	5.840891	-2.041372	-2.253243
C	1.030299	-2.924624	0.726004	H	4.220133	-0.642008	-2.438788
C	1.155270	-4.471535	-1.601067	C	6.538242	-2.895752	-1.400231
H	1.918622	-2.678910	-2.535727	H	6.690549	-3.677745	0.598343
C	0.646748	-4.264476	0.744480	H	6.129171	-1.959549	-3.296729
C	0.714189	-5.036868	-0.409047	H	7.372794	-3.477944	-1.778383
H	1.200861	-5.065956	-2.507513	N	1.953721	-1.019821	-0.543218
H	0.268965	-4.681440	1.671600	N	1.860859	1.136440	-0.653674
H	0.407802	-6.077204	-0.378254	C	1.420487	2.451302	-0.989099
S	0.910835	-2.016807	2.283277	C	1.863438	3.030349	-2.186174
O	-0.272840	-2.609175	2.945947	C	0.597778	3.155304	-0.100763
O	0.633141	-0.602081	1.900660	C	1.486324	4.341460	-2.470421
O	2.197786	-2.193819	2.958709	C	0.262758	4.470511	-0.419123

C	0.696724	5.081939	-1.592964	H	-0.051848	3.242712	1.947323
H	2.538950	0.641550	2.518793	H	-0.984207	2.142226	0.919370
H	3.120625	2.168176	4.357562	H	0.617092	1.665886	1.478812
C	3.148947	1.517151	2.315501	H	1.816104	4.793244	-3.403134
C	3.466672	2.388299	3.352502	H	-0.363540	5.031212	0.271509
C	3.594252	1.784060	1.018295	B	-3.820324	1.055153	-0.390367
C	4.223423	3.531723	3.108336	O	-3.629226	1.368875	0.952448
H	4.469464	4.206726	3.922332	O	-4.386094	2.087985	-1.080407
C	4.352349	2.931056	0.780862	C	-4.342062	2.603575	1.209257
C	4.666051	3.804022	1.817578	C	-4.411649	3.243608	-0.221726
H	4.695295	3.145604	-0.228380	C	-3.179771	4.076670	-0.571827
H	5.256906	4.692276	1.617201	H	-3.216314	4.332753	-1.633357
C	-2.479356	-0.333500	-2.122506	H	-3.138125	5.003455	0.008298
C	-3.436565	-0.331288	-0.987980	H	-2.259355	3.513530	-0.403206
C	2.715596	2.257072	-3.159709	C	-5.681795	4.035858	-0.497557
H	2.646680	2.689802	-4.160344	H	-5.647766	4.437753	-1.513064
H	3.772944	2.271417	-2.869846	H	-6.571997	3.410909	-0.411323
H	2.401172	1.211074	-3.217770	H	-5.769923	4.875354	0.199638
C	0.026851	2.514218	1.135066	C	-3.575250	3.420846	2.237511

H	-2.544439	3.595808	1.926942
H	-4.062186	4.388763	2.391916
H	-3.561235	2.895704	3.197276
C	-5.713030	2.208010	1.758653
H	-6.273032	1.614963	1.030155
H	-5.576321	1.601801	2.658499
H	-6.306950	3.086512	2.024475
C	3.216040	-0.621309	0.111351
C	-4.011937	-1.405501	-0.394960
H	-4.682746	-1.236554	0.455296
C	-3.863727	-2.848737	-0.794563
C	-2.833384	-3.576047	0.090547
C	-5.216885	-3.575572	-0.744717
H	-3.494853	-2.894835	-1.828216
C	-2.717915	-5.064884	-0.245699
H	-3.135875	-3.477903	1.145928
H	-1.851076	-3.098405	-0.016964
C	-5.092264	-5.057888	-1.103263
H	-5.629078	-3.485392	0.272171
H	-5.924203	-3.074731	-1.414864
C	-4.079359	-5.761657	-0.197246
H	-2.018551	-5.547694	0.444664
H	-2.289034	-5.168907	-1.251699
H	-6.070174	-5.547261	-1.034854
H	-4.765930	-5.149444	-2.148376
H	-3.976933	-6.814454	-0.482796
H	-4.452716	-5.750001	0.836480
K	-1.976985	-0.686715	1.838735
C	0.326470	6.511492	-1.894611
H	0.979562	7.205657	-1.354333
H	0.418824	6.731483	-2.961294
H	-0.702469	6.725738	-1.591170
H	-2.530112	-1.252638	-2.720910
H	-2.675429	0.519181	-2.785635

II’-*Z,S*

Electronic Energy BS1 = -4720.19036475 Hartree

Electronic Energy BS2 = -6164.54336178 Hartree

Zero-point Energy Correction = 1.058639 Hartree

Thermal Correction to Enthalpy = 1.125071 Hartree

Thermal Correction to Free Energy = 0.954711 Hartree

Chemical symbol X, Y, Z

H	3.475702	1.913906	0.546750
C	1.575138	2.070063	1.620349
H	1.027399	3.014305	1.693846
C	0.799935	0.325336	0.211131
C	2.385037	0.514804	-1.657493
C	1.603041	0.791026	-2.778412
C	3.659587	-0.040813	-1.834721
C	2.069444	0.508475	-4.056657
H	0.629682	1.240640	-2.632778
C	4.133677	-0.291677	-3.119773
C	3.342081	-0.022768	-4.229795
H	1.441786	0.726295	-4.914548
H	5.122013	-0.722617	-3.228048
H	3.720397	-0.230231	-5.225418
S	4.730313	-0.492205	-0.463966
O	5.761752	-1.379609	-1.061543
O	3.869657	-1.286122	0.470168
O	5.250007	0.745916	0.119686
Cu	-0.467248	-0.800681	-0.806249
C	2.256918	3.280041	-0.549788
C	3.383604	3.862280	-1.126582
C	0.991361	3.785291	-0.859036
C	3.249885	4.946542	-1.991303
H	4.364775	3.451176	-0.904548
C	0.856362	4.865069	-1.722564
H	0.104833	3.321601	-0.433859
C	1.987807	5.449987	-2.289368
H	4.133672	5.394275	-2.434815
H	-0.132368	5.248321	-1.955832
H	1.882569	6.293757	-2.964209
N	1.892818	0.858401	-0.367043
N	0.594075	0.983533	1.358861
C	-0.706407	-2.435817	-1.939799
C	0.006577	-2.771063	-0.777624

C	-0.292144	0.604377	2.408719	C	-1.648508	2.689229	1.922871
C	-1.326009	1.480734	2.759799	H	-2.700816	2.960968	2.036227
C	-0.020574	-0.544323	3.166729	H	-1.048286	3.560214	2.213003
C	-2.056568	1.217348	3.917364	H	-1.475587	2.485163	0.864599
C	-0.773140	-0.760440	4.318886	C	1.022146	-1.549452	2.757156
C	-1.786154	0.109951	4.717051	H	1.379303	-2.107080	3.626574
H	3.768200	0.456796	2.151968	H	0.586734	-2.266409	2.052580
H	4.846887	0.086335	4.327490	H	1.883232	-1.093337	2.265074
C	3.396731	0.981098	3.026669	H	-2.859259	1.896149	4.197275
C	4.003250	0.766305	4.260188	H	-0.564650	-1.642161	4.920654
C	2.316877	1.859610	2.922747	B	-3.604237	-0.334148	-0.074874
C	3.538688	1.418836	5.398967	O	-2.897912	-1.320716	0.581920
H	4.016046	1.247696	6.359041	O	-4.945560	-0.369154	0.234789
C	1.858864	2.513975	4.067121	C	-3.839198	-2.142825	1.305456
C	2.462160	2.295382	5.300910	C	-5.104286	-1.215023	1.383813
H	1.011883	3.191533	3.992778	C	-5.103085	-0.298675	2.609545
H	2.092523	2.809405	6.182624	H	-5.898429	0.442655	2.494994
C	-1.616131	0.618246	-1.611426	H	-5.279748	-0.858348	3.533182
C	-2.951092	0.793223	-0.927423	H	-4.150535	0.229963	2.700625

C	-6.437132	-1.943337	1.276144	C	-2.034108	-3.023960	-2.276287
H	-7.254374	-1.218974	1.328640	C	-3.076198	-2.271204	-2.826845
H	-6.520275	-2.481951	0.330849	C	-2.210212	-4.405093	-2.140220
H	-6.556892	-2.654575	2.099891	C	-4.264150	-2.881067	-3.215491
C	-3.234985	-2.503559	2.652768	H	-2.959538	-1.200015	-2.934041
H	-2.935550	-1.615968	3.206479	C	-3.397235	-5.017774	-2.531536
H	-3.949336	-3.079335	3.250998	H	-1.399850	-5.001537	-1.730157
H	-2.342680	-3.118574	2.501664	C	-4.430184	-4.257111	-3.070556
C	-4.078431	-3.410520	0.494406	H	-5.065661	-2.275552	-3.627156
H	-4.482111	-3.184692	-0.494255	H	-3.512534	-6.091316	-2.416446
H	-3.128831	-3.929857	0.354583	H	-5.357563	-4.731445	-3.376279
H	-4.765223	-4.085838	1.013051	H	-1.083414	1.580619	-1.606123
H	-0.122943	-2.143824	-2.814979	H	-1.767364	0.390257	-2.676433
C	1.481641	-3.008448	-0.679034	C	-3.591637	1.979294	-0.853238
H	2.062816	-2.254552	-0.148377	H	-4.539883	2.021344	-0.314778
H	-0.554662	-3.224689	0.037217	C	-3.126438	3.280762	-1.452548
C	2.417069	2.061116	0.330000	C	-3.388493	3.326294	-2.969380
Cl	1.795080	-4.550143	0.302990	C	-3.765697	4.494269	-0.766002
Cl	2.299683	-3.203468	-2.274779	H	-2.036131	3.363307	-1.320984

C	-2.899462	4.638979	-3.586000
H	-4.467568	3.214683	-3.144391
H	-2.898618	2.470347	-3.444358
C	-3.281606	5.812324	-1.374664
H	-4.858475	4.424629	-0.867834
H	-3.549402	4.470730	0.308709
C	-3.521057	5.852123	-2.887193
H	-3.126104	4.662509	-4.657828
H	-1.805005	4.687845	-3.497289
H	-3.778314	6.660143	-0.889818
H	-2.206929	5.925310	-1.172981
H	-3.124557	6.781920	-3.310491
H	-4.603486	5.856053	-3.075895
K	4.899624	-3.641520	-0.112031
C	-2.619013	-0.183902	5.937462
H	-3.462161	-0.835374	5.677669
H	-2.034081	-0.694747	6.707210
H	-3.031751	0.730908	6.370923

TS'$_{OA}$-*Z,S*

Imaginary Freq = -177.2327 cm^{-1}

Electronic Energy BS1 = -4720.18254731 Hartree

Electronic Energy BS2 = -6164.53635847 Hartree

Zero-point Energy Correction = 1.057643 Hartree

Thermal Correction to Enthalpy = 1.123911 Hartree

Thermal Correction to Free Energy = 0.954137 Hartree

Chemical symbol X, Y, Z

H	3.681995	1.294999	0.699618
C	1.785570	1.749811	1.688258
H	1.352364	2.751439	1.773183
C	0.836970	0.236162	0.130486
C	2.527676	0.330419	-1.662392
C	1.923924	0.839349	-2.810096
C	3.683349	-0.452884	-1.780218
C	2.448764	0.565295	-4.067552
H	1.047807	1.466435	-2.701272
C	4.220749	-0.698356	-3.040461
C	3.606393	-0.195689	-4.182164

H	1.962303	0.964932	-4.951200	N	1.990746	0.677032	-0.391073
H	5.109882	-1.314363	-3.108727	N	0.688303	0.807694	1.331304
H	4.032399	-0.402180	-5.158514	C	-1.235076	-2.289153	-1.990492
S	4.509491	-1.216917	-0.372538	C	-0.529135	-2.789386	-0.867235
O	5.358500	-2.280872	-0.960914	C	-0.257299	0.487247	2.350825
O	3.426307	-1.829361	0.455069	C	-1.156686	1.483294	2.752355
O	5.242929	-0.150311	0.316128	C	-0.128670	-0.710821	3.070615
Cu	-0.530576	-0.741717	-0.911323	C	-1.892789	1.285893	3.919021
C	2.850104	2.974104	-0.317656	C	-0.885283	-0.859788	4.231309
C	4.118887	3.371489	-0.733487	C	-1.758113	0.128218	4.681334
C	1.738045	3.735599	-0.686755	H	3.659204	-0.206979	2.272448
C	4.278925	4.525454	-1.497452	H	4.553894	-0.792297	4.484068
H	4.978143	2.761528	-0.468112	C	3.337041	0.355117	3.143345
C	1.896007	4.883740	-1.453173	C	3.838505	0.019463	4.396738
H	0.741247	3.420772	-0.384424	C	2.421896	1.401647	3.015669
C	3.169275	5.282384	-1.857907	C	3.431921	0.718042	5.530499
H	5.271632	4.827850	-1.815478	H	3.826297	0.450991	6.506163
H	1.026644	5.467313	-1.739876	C	2.023042	2.101992	4.154677
H	3.292024	6.179558	-2.456466	C	2.521387	1.763299	5.407951

H	1.303872	2.911812	4.060517	C	-5.133591	0.659639	2.417518
H	2.199400	2.314818	6.285618	H	-5.762007	1.539029	2.253041
C	-1.486717	0.760300	-1.764909	H	-5.429439	0.191511	3.361098
C	-2.742734	1.173285	-1.049531	H	-4.095014	0.989023	2.506787
C	-1.315046	2.763421	1.975860	C	-6.753476	-0.739441	1.120131
H	-2.350937	3.109677	2.022534	H	-7.405498	0.138096	1.122124
H	-0.681859	3.562676	2.379967	H	-6.929375	-1.291658	0.195710
H	-1.065121	2.621273	0.923125	H	-7.030480	-1.373279	1.968594
C	0.786881	-1.827167	2.643181	C	-3.755677	-1.871763	2.603634
H	1.214586	-2.322338	3.518624	H	-3.288406	-1.036862	3.120777
H	0.223785	-2.582778	2.082628	H	-4.583583	-2.255395	3.209390
H	1.610693	-1.488563	2.013330	H	-3.009059	-2.665183	2.506185
H	-2.584457	2.061452	4.240933	C	-4.732835	-2.693620	0.469908
H	-0.782009	-1.777949	4.804986	H	-5.072012	-2.440142	-0.535649
B	-3.626712	0.198967	-0.211238	H	-3.908259	-3.402241	0.377242
O	-3.147585	-0.883291	0.492115	H	-5.548892	-3.187557	1.005227
O	-4.952309	0.449036	0.054509	H	-0.645405	-2.123279	-2.893259
C	-4.251977	-1.463229	1.227328	C	0.858250	-3.054189	-0.833184
C	-5.302011	-0.294819	1.233197	H	1.475508	-2.807865	0.018621

H	-1.076404	-3.045206	0.037455	C	-2.365868	3.664621	-1.462294
C	2.692812	1.680324	0.446517	C	-2.514092	3.818952	-2.986869
Cl	1.114198	-5.285412	-0.178578	C	-2.794205	4.957646	-0.756524
Cl	1.752792	-3.003909	-2.324466	H	-1.290300	3.524242	-1.267959
C	-2.645326	-2.661376	-2.281924	C	-1.710548	5.015888	-3.501246
C	-3.560326	-1.779332	-2.865229	H	-3.577589	3.952761	-3.227752
C	-3.024686	-3.995934	-2.097825	H	-2.190589	2.897058	-3.480612
C	-4.827358	-2.216584	-3.236501	C	-2.009174	6.168821	-1.263545
H	-3.286027	-0.743304	-3.021404	H	-3.867911	5.116043	-0.932621
C	-4.288540	-4.435817	-2.479502	H	-2.666993	4.851014	0.327138
H	-2.311742	-4.694871	-1.668850	C	-2.112660	6.309388	-2.785603
C	-5.195863	-3.547094	-3.048254	H	-1.843171	5.125766	-4.583156
H	-5.528331	-1.512968	-3.674431	H	-0.641138	4.823016	-3.334423
H	-4.561561	-5.476184	-2.332837	H	-2.363298	7.082676	-0.774113
H	-6.182548	-3.887997	-3.345756	H	-0.954871	6.052440	-0.976618
H	-0.779847	1.596756	-1.805635	H	-1.494783	7.145325	-3.132263
H	-1.693390	0.483479	-2.804422	H	-3.150460	6.554193	-3.050122
C	-3.110613	2.466951	-0.932880	None	4.021643	-4.405170	-0.086137
H	-4.032483	2.687154	-0.393200	C	-2.589461	-0.082597	5.919581

H	-3.522978	-0.601252	5.670380
H	-2.060784	-0.693796	6.655914
H	-2.855642	0.867683	6.389908

III'-*Z,S*

Electronic Energy BS1 = -4720.19894318 Hartree

Electronic Energy BS2 = -6164.56066763 Hartree

Zero-point Energy Correction = 1.059716 Hartree

Thermal Correction to Enthalpy = 1.126494 Hartree

Thermal Correction to Free Energy = 0.955707 Hartree

Chemical symbol X, Y, Z

H	3.794183	0.814149	0.544293
C	2.060848	1.071131	1.845748
H	1.641229	2.010554	2.219032
C	0.905038	0.064069	0.060012
C	2.323409	0.695119	-1.850826
C	1.606874	1.501946	-2.732244
C	3.404396	-0.058591	-2.322907
C	1.930488	1.532962	-4.083995
H	0.806917	2.120043	-2.343209
C	3.734015	-0.007632	-3.674030
C	2.996928	0.775828	-4.555441
H	1.360288	2.162023	-4.759733
H	4.561509	-0.614731	-4.023643
H	3.260857	0.797180	-5.607667
S	4.422620	-1.104187	-1.263683
O	4.989410	-2.128169	-2.167049
O	3.478436	-1.721449	-0.291454
O	5.396435	-0.198907	-0.642283
Cu	-0.586554	-0.582721	-1.001185
C	3.060452	2.780045	0.177053
C	4.307825	3.164954	-0.311803
C	2.026762	3.718734	0.235193
C	4.524857	4.478635	-0.721251
H	5.097436	2.421805	-0.386663
C	2.242597	5.028736	-0.176216
H	1.043713	3.425733	0.598371
C	3.495257	5.411388	-0.653365

H	5.499760	4.770011	-1.098783	H	4.661748	-1.233348	5.963179
H	1.435091	5.753158	-0.126576	C	2.642162	0.904293	4.273035
H	3.664172	6.434630	-0.973859	C	3.294800	0.309919	5.348211
N	1.955780	0.691606	-0.473434	H	1.971664	1.742732	4.445380
N	0.927838	0.226409	1.381922	H	3.136407	0.686779	6.353667
C	-1.754793	-1.269095	-2.577817	C	-1.396336	1.214275	-1.094071
C	-1.283090	-2.355315	-1.798161	C	-2.631669	1.420628	-0.260626
C	0.049423	-0.358985	2.344954	C	-0.904356	1.962654	2.780673
C	-0.794804	0.489907	3.077351	H	-1.922153	2.313647	2.968754
C	0.159996	-1.722899	2.648483	H	-0.228876	2.549225	3.414753
C	-1.521498	-0.049217	4.133835	H	-0.671649	2.181125	1.737066
C	-0.615224	-2.219117	3.699373	C	1.098869	-2.660082	1.939955
C	-1.447733	-1.403958	4.458609	H	1.823394	-3.058934	2.657034
H	3.838560	-1.036111	1.753236	H	0.570298	-3.519415	1.514515
H	5.013003	-2.078020	3.656738	H	1.663254	-2.182205	1.139951
C	3.692298	-0.648416	2.757711	H	-2.163677	0.606609	4.717915
C	4.345621	-1.240312	3.833362	H	-0.540869	-3.277660	3.936916
C	2.834658	0.431808	2.975018	B	-3.546289	0.272760	0.264646
C	4.148940	-0.766047	5.128179	O	-3.155960	-1.036639	0.400465

O	-4.800521	0.506797	0.765363	H	-1.073264	-0.860057	-3.326499
C	-4.277819	-1.783261	0.937714	C	0.058426	-2.640244	-1.620528
C	-5.187296	-0.646980	1.535884	H	0.400898	-3.400857	-0.911539
C	-4.873019	-0.329566	2.996889	H	-1.967350	-2.812263	-1.086792
H	-5.404004	0.582046	3.283448	C	2.832882	1.331118	0.537655
H	-5.191670	-1.139104	3.660003	Cl	1.084809	-5.510084	-0.305405
H	-3.802693	-0.165045	3.138255	Cl	1.200276	-2.358245	-2.910087
C	-6.684115	-0.865714	1.360274	C	-3.198417	-1.174496	-2.925097
H	-7.227481	-0.024255	1.797952	C	-3.923106	0.020781	-2.960921
H	-6.958471	-0.935611	0.306629	C	-3.837504	-2.354146	-3.328648
H	-7.003055	-1.779783	1.870797	C	-5.262357	0.024079	-3.337793
C	-3.744148	-2.780581	1.953339	H	-3.459332	0.954466	-2.672011
H	-3.129288	-2.289183	2.706298	C	-5.168365	-2.346412	-3.730537
H	-4.569435	-3.305607	2.445027	H	-3.280314	-3.286568	-3.334032
H	-3.120852	-3.525429	1.450361	C	-5.890424	-1.156906	-3.724123
C	-4.946945	-2.517689	-0.218166	H	-5.814039	0.958531	-3.332104
H	-5.307416	-1.822691	-0.979240	H	-5.640996	-3.272714	-4.040689
H	-4.229606	-3.193470	-0.688591	H	-6.932869	-1.147897	-4.025755
H	-5.787652	-3.119389	0.137826	H	-0.592271	1.878246	-0.767004

H	-1.563275	1.425528	-2.153203	H	-2.777996	7.179003	-0.728025
C	-2.950825	2.656362	0.175548	None	3.587162	-4.258981	-1.095354
H	-3.858802	2.763468	0.769702	C	-2.291346	-1.972349	5.569644
C	-2.161700	3.915083	-0.057555	H	-3.294157	-2.220585	5.201303
C	-2.379138	4.495678	-1.467333	H	-1.853753	-2.887810	5.975308
C	-2.451771	4.981538	1.005665	H	-2.410793	-1.256074	6.387171
H	-1.090132	3.670024	0.010297				

C	-1.502482	5.730934	-1.688940
H	-3.438381	4.762199	-1.584061
H	-2.159467	3.731863	-2.221128
C	-1.588161	6.227048	0.796230
H	-3.515128	5.255623	0.953806
H	-2.281858	4.566249	2.005805
C	-1.754594	6.795675	-0.616607
H	-1.681193	6.148156	-2.685702
H	-0.446961	5.425526	-1.659777
H	-1.834479	6.989200	1.542992
H	-0.535470	5.958220	0.961692
H	-1.082621	7.648336	-0.763374

II-*Z,R*

Electronic Energy BS1 = -4720.20268082 Hartree

Electronic Energy BS2 = -6164.55879377 Hartree

Zero-point Energy Correction = 1.059788 Hartree

Thermal Correction to Enthalpy = 1.125970 Hartree

Thermal Correction to Free Energy = 0.956516 Hartree

Chemical symbol X, Y, Z

H	3.960681	0.927340	0.340154
C	2.435551	1.113941	1.909254
H	2.087252	2.048893	2.364355
C	0.981000	0.171479	0.300772
C	2.125422	0.704158	-1.780447

C	1.436353	1.632684	-2.556139	C	2.135769	5.061307	-0.167572
C	2.939312	-0.249493	-2.407397	H	1.037367	3.329417	0.476672
C	1.554524	1.623795	-3.941202	C	3.375927	5.568472	-0.553921
H	0.807325	2.358074	-2.054622	H	5.452352	5.118514	-0.892302
C	3.069399	-0.243474	-3.794288	H	1.265332	5.709561	-0.152592
C	2.380112	0.692251	-4.559377	H	3.471686	6.612928	-0.833464
H	1.000915	2.345787	-4.531407	N	1.992401	0.750565	-0.365185
H	3.714927	-0.981450	-4.256080	N	1.238611	0.288186	1.608222
H	2.486553	0.687275	-5.639399	C	-1.423647	-1.953790	-1.787795
S	3.889787	-1.464191	-1.483459	C	-0.210763	-2.354619	-1.230832
O	4.378370	-2.424252	-2.509185	C	0.347634	-0.160721	2.632918
O	2.933242	-2.157102	-0.569204	C	-0.695157	0.687120	3.032695
O	4.947415	-0.725507	-0.786616	C	0.527637	-1.417639	3.221095
Cu	-0.625059	-0.418364	-0.670135	C	-1.596766	0.230557	3.989410
C	3.122530	2.877766	0.158711	C	-0.389700	-1.825486	4.190511
C	4.356125	3.388954	-0.236452	C	-1.464648	-1.029361	4.573122
C	2.010025	3.724411	0.189951	H	4.564075	-0.636317	1.440601
C	4.485483	4.731459	-0.586431	H	5.953658	-1.748727	3.125454
H	5.216696	2.727072	-0.280432	C	4.384663	-0.432221	2.492115

C	5.164252	-1.076806	3.447995	H	-0.266400	-2.807477	4.642093
C	3.370751	0.441239	2.891067	B	-4.001139	0.370338	0.141403
C	4.932755	-0.871903	4.805702	O	-4.149920	-0.847423	-0.496887
H	5.541278	-1.381234	5.546749	O	-4.885653	0.500565	1.192267
C	3.150824	0.650707	4.253978	C	-5.338312	-1.469148	0.022024
C	3.919680	-0.005975	5.208899	C	-5.475515	-0.787553	1.424434
H	2.356418	1.322559	4.569167	C	-4.636619	-1.471771	2.507370
H	3.730938	0.162355	6.264447	H	-4.623655	-0.832929	3.394495
C	-1.855998	1.087708	-1.187900	H	-5.049640	-2.446495	2.784540
C	-2.973131	1.458135	-0.270237	H	-3.601456	-1.602780	2.179327
C	-0.828009	2.064228	2.449148	C	-6.907256	-0.601766	1.907073
H	-1.793532	2.500667	2.706618	H	-6.905005	-0.117477	2.887164
H	-0.040768	2.732229	2.819506	H	-7.481336	0.024299	1.222014
H	-0.767131	2.030704	1.360078	H	-7.409290	-1.569882	2.005401
C	1.668361	-2.332051	2.859245	C	-5.137462	-2.976859	0.054341
H	2.449884	-2.281865	3.625133	H	-4.232553	-3.247621	0.599422
H	1.318805	-3.368884	2.808514	H	-5.995021	-3.469674	0.523926
H	2.134860	-2.060594	1.909633	H	-5.043430	-3.359006	-0.966469
H	-2.423984	0.873558	4.279700	C	-6.482438	-1.110296	-0.929185

H	-6.644128	-0.029232	-0.958911	H	0.092321	-0.721727	-6.027910
H	-6.221600	-1.442438	-1.937809	H	-2.225904	-0.353606	-6.849756
H	-7.417076	-1.597758	-0.637114	H	-1.228499	1.957185	-1.413633
H	-2.344048	-2.199215	-1.263737	H	-2.270782	0.742413	-2.141798
C	-0.055402	-3.222936	-0.052601	C	-3.174665	2.691596	0.242425
H	0.854211	-3.012918	0.500115	H	-4.016700	2.841741	0.919551
H	0.715363	-2.281025	-1.795518	C	-2.330069	3.910413	-0.030469
C	2.994834	1.409518	0.496257	C	-2.572227	4.501387	-1.431803
Cl	-1.415118	-3.240779	1.083081	C	-2.534493	5.001635	1.028776
Cl	0.224123	-5.027103	-0.594747	H	-1.267728	3.617611	0.000880
C	-1.600118	-1.523541	-3.188198	C	-1.636094	5.683430	-1.696171
C	-2.900314	-1.292442	-3.654947	H	-3.617874	4.831857	-1.501673
C	-0.529139	-1.308214	-4.063736	H	-2.435741	3.721867	-2.187647
C	-3.124584	-0.878029	-4.962773	C	-1.622900	6.206018	0.787056
H	-3.728880	-1.410459	-2.962849	H	-3.585675	5.323725	1.002721
C	-0.754590	-0.890607	-5.369347	H	-2.358825	4.593853	2.030719
H	0.493800	-1.439810	-3.723411	C	-1.792911	6.770363	-0.627448
C	-2.052681	-0.679088	-5.828702	H	-1.820548	6.104251	-2.691057
H	-4.140226	-0.701909	-5.304099	H	-0.597794	5.320137	-1.697010

H	-1.816316	6.987306	1.530724
H	-0.580820	5.890010	0.934476
H	-1.078220	7.583634	-0.800267
H	-2.795934	7.210157	-0.714700
K	3.122189	-4.538619	-1.670512
C	-2.477526	-1.531778	5.569541
H	-2.002238	-2.118468	6.360880
H	-3.022892	-0.706886	6.035834
H	-3.213353	-2.177933	5.077528

TS_{OA}-*Z,R*

Imaginary Freq = -194.5319 cm^{-1}

Electronic Energy BS1 = -4720.19904620 Hartree

Electronic Energy BS2 = -6164.55378663 Hartree

Zero-point Energy Correction = 1.058043 Hartree

Thermal Correction to Enthalpy = 1.124400 Hartree

Thermal Correction to Free Energy = 0.953655 Hartree

Chemical symbol X, Y, Z

H	3.922559	0.259389	0.343657
C	2.496391	0.699147	1.943377
H	2.274771	1.659626	2.426155
C	0.909997	0.083165	0.309372
C	2.105356	0.587785	-1.754924
C	1.599638	1.669595	-2.470669
C	2.748239	-0.454389	-2.439119
C	1.713519	1.719557	-3.855068
H	1.120103	2.471320	-1.921013
C	2.875631	-0.390601	-3.824977
C	2.358150	0.690769	-4.531525
H	1.302793	2.563834	-4.397991
H	3.383183	-1.202580	-4.332835
H	2.461321	0.726489	-5.611244
S	3.508741	-1.856721	-1.601222
O	3.796397	-2.834170	-2.680475
O	2.481952	-2.410602	-0.672488
O	4.697297	-1.329255	-0.919932
Cu	-0.733705	-0.320557	-0.699350
C	3.583261	2.352431	0.271849

C	4.893871	2.546997	-0.158630	C	-1.597542	-1.153256	4.542579
C	2.727155	3.452375	0.376243	H	4.370026	-1.311122	1.416457
C	5.353167	3.824929	-0.468861	H	5.592757	-2.649761	3.062166
H	5.549852	1.687513	-0.265580	C	4.216220	-1.115301	2.473670
C	3.181225	4.726578	0.055962	C	4.899645	-1.888861	3.407164
H	1.695023	3.307868	0.691038	C	3.326458	-0.127302	2.901959
C	4.497279	4.915414	-0.363699	C	4.692411	-1.699582	4.770890
H	6.376626	3.964260	-0.802235	H	5.224673	-2.309896	5.493978
H	2.508840	5.575912	0.125653	C	3.130992	0.063461	4.271223
H	4.848999	5.911500	-0.613333	C	3.801747	-0.720834	5.203294
N	1.972302	0.585790	-0.337126	H	2.432382	0.824702	4.609382
N	1.197576	0.056324	1.613649	H	3.632442	-0.564361	6.263873
C	-1.776276	-1.583208	-1.910220	C	-1.742621	1.328653	-1.211029
C	-0.641765	-2.261842	-1.420770	C	-2.693350	1.862194	-0.198590
C	0.274119	-0.358482	2.625229	C	-0.561791	2.025930	2.732612
C	-0.623385	0.583824	3.147385	H	-1.509076	2.525256	2.940900
C	0.290580	-1.678982	3.090273	H	0.229943	2.557559	3.275302
C	-1.557289	0.165214	4.090470	H	-0.367673	2.124940	1.664148
C	-0.655681	-2.049212	4.046939	C	1.293046	-2.699911	2.620899

H	2.077745	-2.823770	3.374885	H	-4.756023	-2.556478	0.561691
H	0.807768	-3.672363	2.484718	H	-6.530558	-2.459817	0.543293
H	1.784174	-2.409108	1.690059	H	-5.619732	-2.460940	-0.975882
H	-2.272173	0.887765	4.476703	C	-6.635470	0.004421	-0.809514
H	-0.662109	-3.078522	4.398346	H	-6.603572	1.097442	-0.797364
B	-3.898749	0.975117	0.217799	H	-6.470028	-0.328772	-1.837605
O	-4.280530	-0.165768	-0.459018	H	-7.631988	-0.321321	-0.498050
O	-4.705538	1.221633	1.305537	H	-2.717594	-1.712020	-1.381338
C	-5.544935	-0.588783	0.085126	C	-0.586731	-3.035680	-0.239894
C	-5.513698	0.050030	1.514823	H	0.365567	-3.146231	0.255593
C	-4.781249	-0.817435	2.541633	H	0.280478	-2.294184	-1.993336
H	-4.626029	-0.229924	3.450427	C	3.101223	0.947469	0.544440
H	-5.357378	-1.711559	2.798095	Cl	-1.924356	-3.038793	0.864334
H	-3.797245	-1.121523	2.173121	Cl	-0.477489	-5.222760	-0.871647
C	-6.872427	0.472250	2.055027	C	-1.902869	-1.099002	-3.295962
H	-6.753443	0.908223	3.050394	C	-3.171054	-0.727911	-3.759263
H	-7.343475	1.218155	1.412758	C	-0.806520	-0.951727	-4.154697
H	-7.538431	-0.392701	2.138358	C	-3.343107	-0.244711	-5.051788
C	-5.613714	-2.108061	0.059413	H	-4.015793	-0.799361	-3.080249

C	-0.979284	-0.465975	-5.443782
H	0.195610	-1.190956	-3.812168
C	-2.248251	-0.115515	-5.901395
H	-4.334208	0.037486	-5.393457
H	-0.114767	-0.353418	-6.091037
H	-2.380130	0.263229	-6.910090
H	-0.960970	2.046692	-1.469963
H	-2.275285	1.066459	-2.127348
C	-2.592667	3.079578	0.375481
H	-3.351798	3.370096	1.103041
C	-1.504011	4.092014	0.123671
C	-1.616586	4.793714	-1.242929
C	-1.448408	5.150180	1.234391
H	-0.536422	3.562363	0.115973
C	-0.427364	5.732751	-1.465747
H	-2.554012	5.365761	-1.273817
H	-1.676318	4.048616	-2.042016
C	-0.288753	6.126741	1.036598
H	-2.399796	5.701434	1.236449
H	-1.366556	4.667724	2.214201
C	-0.324060	6.777570	-0.349938
H	-0.508073	6.228126	-2.439642
H	0.498072	5.138058	-1.490568
H	-0.302524	6.896890	1.815742
H	0.653436	5.576804	1.162400
H	0.561273	7.407513	-0.496865
H	-1.195379	7.444140	-0.406629
K	2.334070	-4.846858	-1.793699
C	-2.653296	-1.604754	5.518192
H	-2.267263	-2.367292	6.200301
H	-3.029691	-0.769688	6.115214
H	-3.506269	-2.040698	4.985683

III-*Z,R*

Electronic Energy BS1 = -4720.21211126 Hartree

Electronic Energy BS2 = -6164.57066769 Hartree

Zero-point Energy Correction = 1.059216 Hartree

Thermal Correction to Enthalpy = 1.126639 Hartree

Thermal Correction to Free Energy = 0.951546 Hartree

Chemical symbol X, Y, Z

H	-3.851430	0.175038	-0.392294
C	-2.475848	0.012726	-2.081418
H	-2.268247	0.705204	-2.906659
C	-0.900242	0.105377	-0.339424
C	-2.097903	1.393043	1.356889
C	-1.871364	2.749235	1.571016
C	-2.488813	0.584648	2.433013
C	-1.965837	3.291099	2.848411
H	-1.626726	3.373591	0.719803
C	-2.584103	1.129844	3.708847
C	-2.306388	2.476148	3.921602
H	-1.771830	4.348099	2.999958
H	-2.881946	0.479960	4.523810
H	-2.372433	2.888275	4.922927
S	-2.998456	-1.121745	2.205250
O	-3.129130	-1.701399	3.559268
O	-1.898236	-1.791913	1.457357
O	-4.256201	-1.052871	1.442934
Cu	0.687251	-0.259691	0.778073
C	-3.691418	2.119284	-1.234733
C	-4.984416	2.411197	-0.803961
C	-2.987446	3.074026	-1.972731
C	-5.577141	3.631176	-1.119474
H	-5.526514	1.674887	-0.217037
C	-3.582681	4.287570	-2.299723
H	-1.967806	2.870272	-2.293708
C	-4.879621	4.568374	-1.873793
H	-6.585228	3.845202	-0.779260
H	-3.033728	5.016762	-2.886937
H	-5.342061	5.516195	-2.130323
N	-1.937992	0.874521	0.035887
N	-1.171514	-0.422311	-1.528995
C	1.910284	-1.061249	2.097443
C	1.201904	-2.257894	1.656852
C	-0.189265	-1.098186	-2.323739
C	0.694087	-0.321212	-3.087237

C	-0.127717	-2.498678	-2.321449	H	-0.180864	1.453200	-3.942012
C	1.692092	-0.971548	-3.808747	H	0.232350	1.611232	-2.227260
C	0.879816	-3.101974	-3.074853	C	-1.102612	-3.350514	-1.556134
C	1.807060	-2.360640	-3.803290	H	-1.940014	-3.627639	-2.205146
H	-4.190950	-1.735711	-0.752987	H	-0.639429	-4.274901	-1.200784
H	-5.412232	-3.628164	-1.726868	H	-1.508862	-2.821840	-0.691297
C	-4.104274	-1.931345	-1.818294	H	2.396853	-0.376122	-4.384329
C	-4.782636	-3.015887	-2.364807	H	0.954542	-4.186523	-3.061392
C	-3.292764	-1.135441	-2.630132	B	3.781337	1.099502	-0.417905
C	-4.645634	-3.328117	-3.715049	O	4.448296	0.239103	0.419568
H	-5.170199	-4.181390	-4.133366	O	4.465886	1.348540	-1.580585
C	-3.166166	-1.447867	-3.984039	C	5.786872	0.091034	-0.106405
C	-3.831817	-2.542467	-4.525964	C	5.591724	0.449332	-1.622212
H	-2.526829	-0.836866	-4.616331	C	5.177176	-0.744064	-2.484035
H	-3.716779	-2.778975	-5.578891	H	4.906742	-0.382991	-3.479780
C	1.701054	1.417058	1.166736	H	5.990255	-1.467950	-2.587957
C	2.443030	1.808290	-0.055612	H	4.301014	-1.246441	-2.067213
C	0.553987	1.176085	-3.175550	C	6.772955	1.168635	-2.258975
H	1.506371	1.638594	-3.443641	H	6.548431	1.387737	-3.305990

H	6.989511	2.111475	-1.754312	C	0.458148	-0.700257	4.134222
H	7.667430	0.538464	-2.227163	C	2.588738	0.507473	5.458274
C	6.284251	-1.320551	0.160285	H	3.719446	0.112755	3.668717
H	5.608721	-2.072790	-0.245833	C	0.293488	-0.217399	5.425576
H	7.275807	-1.461911	-0.281395	H	-0.390880	-1.151187	3.629723
H	6.364787	-1.486626	1.237791	C	1.356165	0.388241	6.093550
C	6.658401	1.103434	0.640298	H	3.423930	0.974225	5.971144
H	6.321783	2.127741	0.456440	H	-0.673729	-0.314730	5.908610
H	6.583309	0.908680	1.713161	H	1.224049	0.762558	7.103762
H	7.708675	1.022535	0.347276	H	0.833189	2.033497	1.415225
H	2.932854	-0.966240	1.735423	H	2.348301	1.319538	2.032652
C	1.526114	-3.059412	0.619356	C	2.119327	2.876684	-0.815396
H	0.961460	-3.971208	0.411233	H	2.774219	3.104215	-1.657242
H	0.307292	-2.567910	2.185059	C	1.012800	3.864460	-0.573226
C	-3.090301	0.778865	-0.890187	C	1.402368	4.811817	0.584055
Cl	2.935391	-2.836621	-0.348346	C	0.685470	4.673903	-1.836181
Cl	-0.340249	-5.809094	1.144679	H	0.108874	3.316331	-0.275816
C	1.695384	-0.586712	3.483836	C	0.356314	5.900446	0.830823
C	2.756049	0.024478	4.164591	H	2.362820	5.283229	0.334021

H	1.569793	4.226663	1.494050
C	-0.365189	5.753888	-1.570858
H	1.608788	5.147797	-2.199270
H	0.347881	4.001096	-2.633711
C	0.066140	6.693775	-0.444210
H	0.695662	6.566605	1.631333
H	-0.576024	5.437294	1.181837
H	-0.562735	6.318681	-2.488950
H	-1.309669	5.274345	-1.284805
H	-0.708616	7.445197	-0.258609
H	0.971445	7.237400	-0.747063
K	-2.352757	-4.260014	2.461274
C	2.939311	-3.043825	-4.524527
H	2.618865	-3.991507	-4.965632
H	3.347724	-2.414150	-5.319670
H	3.754028	-3.267532	-3.825920

II-*Z,S*

Electronic Energy BS1 = -4720.21139358 Hartree

Electronic Energy BS2 = -6164.56584838 Hartree

Zero-point Energy Correction = 1.059473 Hartree

Thermal Correction to Enthalpy = 1.125879 Hartree

Thermal Correction to Free Energy = 0.955604 Hartree

Chemical symbol X, Y, Z

C	-2.648332	0.039980	1.732271
H	-3.336070	-0.802036	1.686486
C	-3.083975	1.159543	0.757351
H	-3.066237	2.128659	1.271685
C	-0.981372	0.314408	0.075502
C	-0.527704	-1.372599	1.755073
C	0.730586	-0.990905	2.209485
C	-0.918657	-2.715793	1.849683
C	1.615473	-1.925782	2.732808
H	1.026760	0.047554	2.123825
C	-0.042509	-3.642698	2.411143
C	1.221610	-3.254993	2.843826
H	2.606266	-1.603842	3.037327
H	-0.360188	-4.676904	2.473455

H	1.895911	-3.994701	3.264065	C	1.548479	-0.492184	-2.647028
S	-2.485466	-3.344674	1.229224	C	0.200829	-0.831309	-2.583244
O	-2.290955	-4.814177	1.125576	C	-0.376044	-2.128417	-2.177539
O	-2.640813	-2.759079	-0.141909	H	-1.275987	-2.045738	-1.569533
O	-3.542990	-2.933593	2.154800	H	-0.524926	-0.233425	-3.127192
Cu	0.723545	0.283765	-0.918518	H	2.288354	-1.195244	-2.269396
C	-2.522477	0.453314	3.180082	C	-1.920253	2.037753	-1.350946
C	-3.222801	-0.263014	4.149415	C	-1.319995	3.294592	-1.179193
C	-1.696662	1.511022	3.569596	C	-2.420623	1.635835	-2.595997
C	-3.120271	0.087908	5.493223	C	-1.194083	4.129406	-2.285423
H	-3.827567	-1.111731	3.844577	C	-2.278862	2.510654	-3.675593
C	-1.594542	1.859628	4.911418	C	-1.663973	3.752694	-3.543571
H	-1.119534	2.055771	2.824000	H	-4.485476	-1.168913	0.131233
C	-2.310158	1.151214	5.875045	H	-6.658469	-1.328787	-0.996317
H	-3.669394	-0.476419	6.240364	C	-5.005959	-0.257747	-0.147733
H	-0.954231	2.682774	5.208312	C	-6.228092	-0.350407	-0.806286
H	-2.226642	1.425912	6.922017	C	-4.443968	0.994423	0.111795
N	-1.344669	-0.370587	1.167010	C	-6.893544	0.798195	-1.227274
N	-1.984638	1.147548	-0.235303	H	-7.845110	0.719709	-1.744012

C	-5.120716	2.141812	-0.305257	C	5.048034	-1.982542	-0.284065
C	-6.334761	2.048626	-0.977867	C	5.632589	-1.686003	1.141141
H	-4.684212	3.118457	-0.111498	C	6.951980	-0.911674	1.099842
H	-6.845471	2.950503	-1.300192	H	7.189747	-0.567815	2.109538
C	2.236820	1.479218	-0.335986	H	7.775999	-1.535093	0.740793
C	3.034633	1.022202	0.830927	H	6.870048	-0.032303	0.454864
C	-0.818467	3.727125	0.171305	C	5.767695	-2.905227	2.043527
H	-0.152010	4.588035	0.084112	H	6.169997	-2.600375	3.013241
H	-1.645775	4.007850	0.833951	H	4.800388	-3.382636	2.211998
H	-0.263061	2.920703	0.653705	H	6.451457	-3.639404	1.605299
C	-3.105123	0.310397	-2.807636	C	6.089042	-2.146226	-1.383407
H	-4.192037	0.442172	-2.801479	H	6.694186	-1.245382	-1.498225
H	-2.825951	-0.119567	-3.774654	H	6.751675	-2.990359	-1.166574
H	-2.866021	-0.406938	-2.020969	H	5.588756	-2.343535	-2.335874
H	-0.704349	5.092539	-2.164271	C	4.084263	-3.168524	-0.295486
H	-2.650544	2.200547	-4.649833	H	3.307513	-3.051341	0.466406
B	3.978541	-0.198869	0.653765	H	3.596362	-3.217479	-1.271929
O	4.274549	-0.799611	-0.553469	H	4.609295	-4.113113	-0.125346
O	4.645874	-0.806929	1.699474	Cl	0.724149	-3.204997	-1.271219

Cl	-0.978106	-3.073229	-3.686671	H	1.266113	2.781204	1.918411
C	2.092066	0.601446	-3.478243	C	1.138416	4.100602	4.341221
C	3.481170	0.715011	-3.606344	H	2.843177	2.787889	4.523719
C	1.281518	1.552456	-4.111304	H	1.326515	1.940076	4.231104
C	4.044516	1.738418	-4.361162	C	2.198660	5.348018	2.426875
H	4.116048	0.011633	-3.075144	H	3.951370	4.089116	2.515037
C	1.846121	2.576333	-4.860230	H	3.140935	4.055605	0.953121
H	0.202088	1.517346	-4.002643	C	1.893251	5.365728	3.926184
C	3.230061	2.672301	-4.994507	H	0.941663	4.113156	5.419979
H	5.124523	1.810648	-4.446692	H	0.159302	4.097798	3.839230
H	1.198926	3.306047	-5.338248	H	2.767297	6.240234	2.141289
H	3.668960	3.473532	-5.580911	H	1.251527	5.386078	1.870052
H	2.880895	1.533454	-1.218043	H	1.314637	6.257976	4.190644
C	3.015086	1.577825	2.064043	H	2.837095	5.421743	4.485854
H	1.809755	2.477688	-0.185780	K	-2.183129	-5.034167	-1.445661
H	3.647431	1.149155	2.842476	C	-1.516881	4.676004	-4.725753
C	2.224929	2.796893	2.455653	H	-2.327002	5.413228	-4.750444
C	1.903053	2.831506	3.954875	H	-0.573156	5.226955	-4.680083
C	2.963551	4.082826	2.033152	H	-1.544837	4.123077	-5.668647

TS_{OA}-*Z,S*

Imaginary Freq = -199.7965 cm^{-1}

Electronic Energy BS1 = -4720.20596588 Hartree

Electronic Energy BS2 = -6164.55870573 Hartree

Zero-point Energy Correction = 1.058480 Hartree

Thermal Correction to Enthalpy = 1.124841 Hartree

Thermal Correction to Free Energy = 0.954315 Hartree

Chemical symbol X, Y, Z

C	2.628308	1.437471	-0.964567
H	3.311174	0.875196	-1.598232
C	3.014663	1.289187	0.525098
H	2.942388	2.261975	1.029320
C	0.933808	0.236654	0.155318
C	0.539343	0.650693	-2.199009
C	-0.696416	1.285905	-2.247560
C	0.961613	-0.132007	-3.282920
C	-1.532844	1.136098	-3.347685
H	-1.011213	1.876754	-1.396147
C	0.136672	-0.246285	-4.399355
C	-1.107787	0.376195	-4.431946
H	-2.510470	1.607439	-3.337416
H	0.474006	-0.860888	-5.225753
H	-1.743408	0.259672	-5.304141
S	2.501337	-1.067115	-3.299088
O	2.336155	-2.057470	-4.390516
O	2.565017	-1.764131	-1.976918
O	3.596487	-0.112479	-3.499478
Cu	-0.751956	-0.634233	0.692178
C	2.572340	2.851168	-1.491243
C	3.285993	3.166564	-2.646567
C	1.794272	3.833423	-0.874404
C	3.243452	4.457424	-3.167451
H	3.855113	2.386955	-3.144610
C	1.752227	5.121644	-1.395007
H	1.206785	3.588384	0.009106
C	2.480972	5.436638	-2.540566
H	3.802786	4.693570	-4.067170

H	1.149439	5.881468	-0.909418	C	6.252556	-0.731279	0.301004
H	2.445607	6.443400	-2.945131	C	4.386056	0.714481	0.810865
N	1.300649	0.785753	-1.005371	C	6.863070	-0.387574	1.504237
N	1.919328	0.433959	1.038979	H	7.825334	-0.814469	1.769524
C	-1.762279	-2.353507	1.140809	C	5.008070	1.060116	2.011980
C	-0.426390	-2.686462	0.843044	C	6.235902	0.509892	2.363728
C	0.120456	-2.910093	-0.439003	H	4.518590	1.761398	2.683385
H	1.149482	-2.651369	-0.647440	H	6.703614	0.786071	3.303409
H	0.285327	-2.844316	1.646934	C	-2.158446	0.609727	1.380778
H	-2.499074	-2.388439	0.340560	C	-2.903112	1.363841	0.340164
C	1.866883	-0.074503	2.373002	C	0.874071	2.133211	3.117292
C	1.320625	0.721967	3.388076	H	0.046996	2.414176	3.774053
C	2.337007	-1.370638	2.636214	H	1.691950	2.843406	3.288064
C	1.209659	0.185203	4.669737	H	0.539461	2.253656	2.085983
C	2.209158	-1.862539	3.935102	C	2.977951	-2.234117	1.580158
C	1.644052	-1.105702	4.961011	H	4.061089	-2.265250	1.736194
H	4.545554	-0.504280	-0.970680	H	2.604326	-3.262075	1.632278
H	6.736529	-1.429243	-0.374964	H	2.812610	-1.853312	0.571646
C	5.017518	-0.191356	-0.044484	H	0.761045	0.789129	5.454731

H	2.554497	-2.872424	4.145725	H	-3.367227	-1.205579	-2.845655
B	-3.896867	0.596798	-0.577871	H	-3.758438	-2.712528	-2.002240
O	-4.279954	-0.714329	-0.396602	H	-4.749389	-2.193886	-3.376268
O	-4.523118	1.170331	-1.663843	Cl	-0.873185	-2.713449	-1.854703
C	-5.092511	-1.098687	-1.524139	Cl	0.719222	-5.129940	-0.535237
C	-5.580142	0.287532	-2.074416	C	-2.320091	-2.386957	2.505159
C	-6.862627	0.783363	-1.402982	C	-3.710983	-2.376040	2.660550
H	-7.027894	1.825993	-1.685497	C	-1.515273	-2.372206	3.652088
H	-7.732500	0.196729	-1.711870	C	-4.283744	-2.374747	3.928242
H	-6.778533	0.738215	-0.313483	H	-4.338248	-2.339295	1.774586
C	-5.718373	0.361741	-3.588852	C	-2.089036	-2.365766	4.915912
H	-6.051064	1.362217	-3.877715	H	-0.433867	-2.337066	3.565236
H	-4.766635	0.163306	-4.085409	C	-3.475596	-2.372976	5.060961
H	-6.458741	-0.360876	-3.946512	H	-5.364557	-2.367908	4.030023
C	-6.200597	-2.020034	-1.032762	H	-1.448613	-2.347824	5.792789
H	-6.767472	-1.565981	-0.218216	H	-3.921421	-2.368264	6.050660
H	-6.889561	-2.264234	-1.847638	H	-2.841576	-0.016765	1.956441
H	-5.765412	-2.954101	-0.666336	C	-2.805264	2.695119	0.129187
C	-4.188678	-1.843250	-2.505068	H	-1.616565	1.256305	2.078548

H	-3.415724	3.137563	-0.659016
C	-1.960256	3.657662	0.919075
C	-1.640604	4.928923	0.121509
C	-2.644134	4.018249	2.253728
H	-1.004571	3.170772	1.161320
C	-0.826453	5.930607	0.944469
H	-2.584908	5.399585	-0.187986
H	-1.104940	4.665140	-0.798331
C	-1.827919	5.022812	3.069837
H	-3.631926	4.445733	2.032203
H	-2.821116	3.103119	2.828385
C	-1.528095	6.284408	2.257691
H	-0.639302	6.838169	0.358597
H	0.156664	5.495266	1.176891
H	-2.358988	5.278925	3.993335
H	-0.877677	4.559530	3.370494
H	-0.914945	6.979880	2.841338
H	-2.470770	6.802495	2.034292
K	2.256987	-4.264652	-2.965181
C	1.513977	-1.675692	6.350294
H	2.472802	-1.641815	6.878926
H	0.787329	-1.115026	6.944124
H	1.195889	-2.722120	6.318816

III-*Z,S*

Electronic Energy BS1 = -4720.21773514 Hartree

Electronic Energy BS2 = -6164.57758091 Hartree

Zero-point Energy Correction = 1.059560 Hartree

Thermal Correction to Enthalpy = 1.126713 Hartree

Thermal Correction to Free Energy = 0.954188 Hartree

Chemical symbol X, Y, Z

C	2.487247	1.811380	-0.219334
H	3.249075	1.517727	-0.940869
C	2.758815	1.152462	1.153137
H	2.536090	1.861515	1.960926
C	0.844522	0.187905	0.191234
C	0.561361	1.456817	-1.860004
C	-0.681818	2.076408	-1.814499

C	1.111806	1.089013	-3.094938	H	0.962452	3.459737	1.380864
C	-1.408433	2.300476	-2.978775	C	2.230479	6.102913	-0.332592
H	-1.083851	2.356738	-0.849778	H	3.623813	5.963920	-1.966147
C	0.394376	1.344534	-4.259923	H	0.838520	5.925330	1.299908
C	-0.864466	1.936595	-4.205635	H	2.167915	7.186033	-0.369800
H	-2.393448	2.750652	-2.910762	N	1.206161	1.180704	-0.618151
H	0.828380	1.038869	-5.205101	N	1.725125	0.090813	1.189714
H	-1.415810	2.111322	-5.124178	C	-1.934465	-2.653466	0.100608
S	2.708617	0.273088	-3.263457	C	-0.615579	-3.037353	-0.263137
O	2.692887	-0.344003	-4.608211	C	0.075142	-2.476598	-1.314173
O	2.732264	-0.772286	-2.200888	H	1.120648	-2.703349	-1.481406
O	3.726210	1.314972	-3.074539	H	0.007009	-3.605416	0.424082
Cu	-0.703629	-0.994219	0.181450	H	-2.607489	-2.310213	-0.683081
C	2.394673	3.317383	-0.226758	C	1.635005	-0.921078	2.199896
C	3.128215	4.029019	-1.175376	C	1.039017	-0.612195	3.429914
C	1.562110	4.007783	0.656376	C	2.120519	-2.211943	1.928077
C	3.048446	5.418537	-1.224919	C	0.867302	-1.632412	4.364467
H	3.741828	3.482367	-1.885745	C	1.919636	-3.199748	2.893249
C	1.483141	5.394773	0.606766	C	1.289672	-2.934490	4.108672

H	4.476076	-0.072655	-0.649791	H	2.571269	-3.530364	0.265578
H	6.729299	-0.945246	-0.191991	H	2.754618	-1.811591	-0.111479
C	4.894900	0.024278	0.347807	H	0.382345	-1.401521	5.310002
C	6.163007	-0.483120	0.610383	H	2.272499	-4.206497	2.680834
C	4.157699	0.623367	1.371982	B	-3.785782	0.643258	-0.588727
C	6.703230	-0.406996	1.891837	O	-4.147586	-0.631926	-0.955681
H	7.692796	-0.806640	2.090261	O	-4.401893	1.599648	-1.361026
C	4.705669	0.698905	2.652765	C	-4.941682	-0.537503	-2.160940
C	5.970839	0.184321	2.916576	C	-5.442948	0.948517	-2.112236
H	4.133910	1.163900	3.452384	C	-6.739127	1.121123	-1.318239
H	6.384141	0.249122	3.918051	H	-6.916692	2.187637	-1.159898
C	-2.052859	-0.047247	1.301222	H	-7.596612	0.701621	-1.851642
C	-2.829688	1.004590	0.587111	H	-6.668093	0.641042	-0.337996
C	0.609806	0.788819	3.773457	C	-5.562988	1.626403	-3.469610
H	-0.310267	0.782014	4.363988	H	-5.900897	2.657279	-3.335724
H	1.379829	1.296524	4.365953	H	-4.604352	1.646423	-3.991111
H	0.433431	1.390181	2.880481	H	-6.293257	1.106753	-4.097771
C	2.871725	-2.558945	0.672842	C	-6.040166	-1.589117	-2.096946
H	3.940717	-2.620344	0.901974	H	-6.625307	-1.504042	-1.179511

H	-6.714882	-1.493604	-2.953338	H	-2.711812	-0.815848	1.702442
H	-5.594957	-2.587553	-2.128034	C	-2.822976	2.307209	0.942656
C	-4.015056	-0.815245	-3.342350	H	-1.435296	0.343270	2.111979
H	-3.201269	-0.085286	-3.387739	H	-3.468263	2.981880	0.378656
H	-3.574647	-1.808154	-3.226318	C	-2.062777	2.946338	2.071579
H	-4.563169	-0.792480	-4.288280	C	-1.878465	4.452110	1.836036
Cl	-0.719919	-1.756550	-2.680317	C	-2.769775	2.690800	3.418965
Cl	2.148880	-4.930462	-2.008862	H	-1.063918	2.492352	2.130737
C	-2.590573	-3.149844	1.320702	C	-1.170914	5.134922	3.008413
C	-3.988852	-3.154577	1.380471	H	-2.868189	4.910435	1.698135
C	-1.862864	-3.556000	2.448003	H	-1.323354	4.615692	0.904963
C	-4.645946	-3.586653	2.528155	C	-2.065909	3.389140	4.585058
H	-4.553344	-2.798037	0.524149	H	-3.802477	3.058336	3.344444
C	-2.521253	-3.981325	3.593114	H	-2.835173	1.611553	3.595872
H	-0.777835	-3.510246	2.446422	C	-1.908163	4.888725	4.326337
C	-3.914899	-4.004961	3.635739	H	-1.080312	6.210234	2.817601
H	-5.730947	-3.591084	2.558271	H	-0.147486	4.742366	3.096563
H	-1.943284	-4.286846	4.459903	H	-2.620624	3.215819	5.513558
H	-4.426853	-4.338634	4.532747	H	-1.069638	2.948367	4.725879

H	-1.374953	5.364899	5.156028
H	-2.901477	5.355204	4.277223
K	3.084028	-2.906867	-3.835150
C	1.080095	-4.036635	5.115269
H	2.031700	-4.349059	5.557710
H	0.422239	-3.716884	5.927662
H	0.638104	-4.919741	4.642934

TS_{ER}-*Z,S*

Imaginary Freq = -174.2675 cm^{-1}

Electronic Energy BS1 = -4720.20622861 Hartree

Electronic Energy BS2 = -6164.56722335 Hartree

Zero-point Energy Correction = 1.059420 Hartree

Thermal Correction to Enthalpy = 1.125945 Hartree

Thermal Correction to Free Energy = 0.954618 Hartree

Chemical symbol X, Y, Z

C	-2.299130	-1.787760	1.327289
H	-2.858723	-2.405225	0.624466
C	-2.958331	-0.399704	1.498129
H	-3.007610	-0.137384	2.561760
C	-0.856107	-0.141120	0.458190
C	-0.070321	-2.436400	0.267858
C	1.095877	-2.620265	1.001133
C	-0.295185	-3.211408	-0.877924
C	2.061496	-3.532098	0.588893
H	1.237427	-2.025255	1.893255
C	0.667035	-4.131004	-1.282421
C	1.845653	-4.286677	-0.558395
H	2.979938	-3.634565	1.155481
H	0.474987	-4.710710	-2.178290
H	2.588695	-5.006064	-0.889072
S	-1.825886	-3.136678	-1.816030
O	-1.530529	-3.776954	-3.116699
O	-2.128063	-1.690828	-1.985234
O	-2.818062	-3.846014	-0.993044
Cu	0.606868	0.845979	-0.348094
C	-2.088223	-2.584798	2.593307
C	-2.320313	-3.959579	2.566965

C	-1.597328	-1.996875	3.760495	C	-2.606883	2.417838	-0.513019
C	-2.078185	-4.733752	3.699135	C	-2.028470	4.118893	1.640261
H	-2.678113	-4.416431	1.647860	C	-2.724317	3.803308	-0.629904
C	-1.356097	-2.768356	4.891471	C	-2.439909	4.667492	0.426962
H	-1.392388	-0.929150	3.789401	H	-4.160528	-1.637670	-0.697937
C	-1.598453	-4.140348	4.862652	H	-6.406259	-1.206435	-1.586880
H	-2.263539	-5.802736	3.669533	C	-4.798069	-0.915862	-0.199590
H	-0.978517	-2.298200	5.794383	C	-6.064700	-0.657980	-0.714776
H	-1.411344	-4.743706	5.745423	C	-4.347160	-0.219133	0.922712
N	-0.994615	-1.443421	0.709989	C	-6.885001	0.302739	-0.129862
N	-1.960712	0.492657	0.862473	H	-7.868910	0.503754	-0.541947
C	1.715649	2.129397	-1.512067	C	-5.178765	0.735921	1.510932
C	0.444249	1.874504	-2.251052	C	-6.438410	1.003874	0.986594
C	0.115551	0.810529	-3.003140	H	-4.830419	1.284248	2.383245
H	-0.788423	0.797534	-3.621068	H	-7.070268	1.754174	1.451166
H	-0.307271	2.659589	-2.231862	C	2.356727	1.617361	0.454176
H	2.569989	1.624273	-1.961575	C	2.957792	0.338018	0.938309
C	-2.151056	1.902875	0.712934	C	-1.482467	2.174558	3.139458
C	-1.884186	2.740980	1.803722	H	-0.897052	2.895737	3.715029

H	-2.364558	1.916644	3.738070	H	4.863668	-3.819151	-0.906108
H	-0.889715	1.264384	3.025386	H	6.541896	-3.579719	-1.439218
C	-3.009331	1.541366	-1.666132	C	6.186041	-0.451198	-2.666086
H	-4.095237	1.400634	-1.644902	H	6.743853	0.302146	-2.107486
H	-2.763467	1.991754	-2.632402	H	6.888504	-1.201778	-3.041701
H	-2.557515	0.549979	-1.624894	H	5.719555	0.036896	-3.525616
H	-1.809214	4.774839	2.479572	C	4.227774	-2.004540	-2.671996
H	-3.056139	4.213428	-1.580867	H	3.421025	-2.451809	-2.083580
B	3.935413	-0.452330	0.019984	H	3.781279	-1.403973	-3.466908
O	4.270685	-0.069531	-1.251970	H	4.811591	-2.804781	-3.135129
O	4.607115	-1.570836	0.439766	Cl	1.131081	-0.582906	-3.184477
C	5.113076	-1.107842	-1.809419	Cl	-2.337940	1.123650	-5.290369
C	5.648389	-1.832755	-0.521722	C	1.918651	3.594313	-1.277170
C	6.929646	-1.209711	0.034686	C	3.058318	4.232805	-1.766198
H	7.129083	-1.633902	1.021835	C	0.976841	4.336648	-0.557466
H	7.788463	-1.413482	-0.610656	C	3.248848	5.595601	-1.549981
H	6.826178	-0.126640	0.146450	H	3.795866	3.660671	-2.322573
C	5.812355	-3.339541	-0.659637	C	1.171676	5.693534	-0.335124
H	6.172742	-3.756750	0.284394	H	0.089764	3.849139	-0.161125

C	2.308116	6.328541	-0.833504
H	4.134936	6.083560	-1.943418
H	0.434216	6.252798	0.232487
H	2.459190	7.389641	-0.663063
H	3.147908	2.268539	0.099503
C	2.842822	-0.074600	2.216381
H	1.772847	2.178160	1.190919
H	3.418222	-0.955776	2.503929
C	2.058406	0.575147	3.318155
C	1.647573	-0.443829	4.391665
C	2.869450	1.722007	3.957888
H	1.145450	1.013452	2.892403
C	0.888056	0.212195	5.546530
H	2.555674	-0.923885	4.783637
H	1.039130	-1.239829	3.948977
C	2.104474	2.383064	5.106390
H	3.817346	1.313572	4.334555
H	3.128613	2.461494	3.192439
C	1.690063	1.359535	6.166426
H	0.650443	-0.537589	6.308922
H	-0.071618	0.600977	5.178614
H	2.715851	3.171976	5.557104
H	1.207090	2.872269	4.704101
H	1.109572	1.845053	6.957979
H	2.592130	0.950212	6.641153
K	-2.339128	-1.684615	-4.660236
C	-2.573547	6.157688	0.245188
H	-3.611584	6.435234	0.036505
H	-2.253002	6.701830	1.137673
H	-1.969548	6.501213	-0.600785

IV-*Z,S*

Electronic Energy BS1 = -4720.28814258 Hartree

Electronic Energy BS2 = -6164.64157921 Hartree

Zero-point Energy Correction = 1.062292 Hartree

Thermal Correction to Enthalpy = 1.128762 Hartree

Thermal Correction to Free Energy = 0.958091 Hartree

Chemical symbol X, Y, Z

C	3.271406	-0.302542	-0.558359	Cu	-0.943285	-0.306283	-0.791371
H	3.417806	-0.578868	-1.608091	C	4.460423	0.515453	-0.112754
C	2.930546	-1.562598	0.253660	C	5.125911	1.276375	-1.077705
H	3.210184	-1.430101	1.305270	C	4.898788	0.583886	1.210148
C	0.947866	-0.396703	-0.234836	C	6.186403	2.104711	-0.728334
C	1.829328	1.663315	-1.212260	H	4.797057	1.224918	-2.112245
C	2.259183	2.821966	-0.570530	C	5.962164	1.408874	1.563762
C	1.357083	1.747961	-2.531839	H	4.417299	-0.004526	1.983683
C	2.245082	4.045346	-1.228509	C	6.605342	2.176151	0.597170
H	2.619441	2.739815	0.448328	H	6.686965	2.690972	-1.492146
C	1.353450	2.973475	-3.191380	H	6.290648	1.447195	2.597933
C	1.808082	4.118754	-2.546068	H	7.434772	2.819027	0.874238
H	2.581239	4.937398	-0.711331	N	1.971986	0.430382	-0.514191
H	0.995124	3.007161	-4.214078	N	1.456439	-1.559467	0.179241
H	1.810033	5.067921	-3.071928	C	-2.961461	0.091989	1.542675
S	0.721911	0.326955	-3.425316	C	-2.455035	-0.897791	0.516256
O	0.495158	0.740975	-4.813252	C	-2.883963	-1.061398	-0.774014
O	-0.623157	0.038471	-2.785034	H	-2.757032	-1.992501	-1.338517
O	1.687404	-0.757505	-3.221696	H	-1.924374	-1.755933	0.928245

H	-3.527798	0.872658	1.034294	C	-1.104035	1.940827	1.776122
C	0.616422	-2.612987	0.671467	C	1.169051	-1.854083	3.027789
C	0.409449	-2.717578	2.055810	H	0.645839	-1.787112	3.985504
C	-0.068930	-3.433695	-0.233633	H	2.167703	-2.260559	3.228376
C	-0.539137	-3.624312	2.517701	H	1.296972	-0.840626	2.640320
C	-1.040295	-4.302393	0.276739	C	0.179889	-3.421678	-1.716910
C	-1.300541	-4.400628	1.639846	H	0.870666	-4.233237	-1.971234
H	2.990411	-2.563430	-2.306405	H	-0.757022	-3.595395	-2.257178
H	4.017510	-4.672799	-3.064854	H	0.628477	-2.492277	-2.067278
C	3.501274	-3.202498	-1.591923	H	-0.716008	-3.702040	3.588694
C	4.073467	-4.395972	-2.016939	H	-1.625473	-4.884802	-0.430478
C	3.561574	-2.839582	-0.244030	B	-1.661984	2.843459	0.631987
C	4.710343	-5.235417	-1.105186	O	-2.770239	2.572069	-0.129383
H	5.154556	-6.166828	-1.441827	O	-1.100511	4.059856	0.311882
C	4.199637	-3.683405	0.663236	C	-2.862809	3.587703	-1.151967
C	4.773771	-4.877713	0.237458	C	-2.055586	4.762988	-0.506593
H	4.247456	-3.404251	1.713079	C	-2.901227	5.623715	0.434919
H	5.267463	-5.526673	0.953703	H	-2.238324	6.279347	1.005040
C	-1.826506	0.735958	2.367230	H	-3.611966	6.243890	-0.118429

H	-3.458051	5.004327	1.143948	H	-5.677479	0.182241	1.557611
C	-1.313465	5.645623	-1.497534	C	-4.333330	-2.373933	4.126144
H	-0.765283	6.423539	-0.958893	H	-2.379473	-1.785323	3.464666
H	-0.597981	5.068510	-2.083142	C	-5.707596	-2.199164	3.979773
H	-2.019603	6.133230	-2.177318	H	-7.256634	-1.134440	2.932968
C	-4.331011	3.882495	-1.417679	H	-3.948625	-3.096095	4.840394
H	-4.865254	4.126824	-0.497999	H	-6.399306	-2.782956	4.578847
H	-4.432063	4.719111	-2.116539	H	-2.262689	1.047650	3.325032
H	-4.805440	3.004596	-1.863110	C	0.037536	2.395008	2.325164
C	-2.191712	3.028084	-2.403379	H	-1.096957	-0.045389	2.612870
H	-1.134852	2.824896	-2.220119	H	0.471538	3.303422	1.904797
H	-2.669871	2.084493	-2.672350	C	0.791796	1.836026	3.493056
H	-2.274407	3.721610	-3.245505	C	2.283510	1.699759	3.156351
Cl	-4.127141	-0.065528	-1.520512	C	0.608796	2.721533	4.740265
Cl	-3.120058	-3.557786	-3.172399	H	0.407878	0.840835	3.747211
C	-3.920958	-0.694024	2.427272	C	3.087452	1.156429	4.337938
C	-5.299114	-0.529652	2.285986	H	2.677325	2.688278	2.876723
C	-3.448626	-1.627781	3.354857	H	2.400540	1.063425	2.272037
C	-6.187355	-1.274565	3.057367	C	1.407228	2.178156	5.928535

H	0.944150	3.741717	4.506109
H	-0.455495	2.788642	4.990184
C	2.891655	2.024065	5.584760
H	4.151029	1.104498	4.078760
H	2.762322	0.129351	4.555930
H	1.285298	2.835450	6.796035
H	1.000164	1.198810	6.215701
H	3.436332	1.597647	6.433893
H	3.322372	3.017536	5.399055
K	-2.170228	-1.198750	-4.501733
C	-2.410302	-5.273307	2.163805
H	-2.744137	-5.989605	1.410114
H	-2.095676	-5.827428	3.053515
H	-3.271667	-4.656195	2.444283

II'-*Z,R*

Electronic Energy BS1 = -4720.18446356 Hartree

Electronic Energy BS2 = -6164.53942308 Hartree

Zero-point Energy Correction = 1.059631 Hartree

Thermal Correction to Enthalpy = 1.125772 Hartree

Thermal Correction to Free Energy = 0.956888 Hartree

Chemical symbol X, Y, Z

C	2.966027	0.138206	-1.087232
H	3.643684	-0.714105	-1.106521
C	3.231112	1.021618	0.153405
H	3.643547	1.981909	-0.161634
C	0.967212	0.359130	0.159583
C	0.833389	-1.057191	-1.798282
C	-0.363659	-0.494930	-2.248496
C	1.231029	-2.302151	-2.316862
C	-1.164379	-1.146914	-3.176213
H	-0.670076	0.474959	-1.871115
C	0.426162	-2.942880	-3.259032
C	-0.768255	-2.377148	-3.687334
H	-2.088081	-0.669514	-3.486189
H	0.752461	-3.905479	-3.634844
H	-1.379782	-2.895178	-4.419017
S	2.734784	-3.168099	-1.844164

O	2.521271	-4.580110	-2.251934	C	0.043576	-2.913498	1.115299
O	2.763929	-3.087412	-0.347323	H	0.787112	-2.696510	0.351941
O	3.864906	-2.502967	-2.489791	H	-1.824483	-2.207568	0.356272
Cu	-0.841775	0.034351	0.970163	H	-0.957250	-1.197256	3.126378
C	3.094790	0.894317	-2.392371	C	1.583216	2.140861	1.738475
C	4.200168	0.657762	-3.207492	C	1.641253	3.518429	1.469068
C	2.157053	1.857241	-2.774625	C	1.280903	1.682555	3.027366
C	4.384702	1.396163	-4.373398	C	1.258659	4.410525	2.467923
H	4.899350	-0.127008	-2.937475	C	0.888749	2.612918	3.990259
C	2.343483	2.597661	-3.936416	C	0.842258	3.977098	3.724884
H	1.268326	2.020962	-2.169490	H	3.587922	-1.618838	0.910314
C	3.462061	2.372182	-4.735689	H	4.862593	-2.444646	2.848843
H	5.247006	1.200397	-5.002947	C	4.131277	-0.915109	1.534952
H	1.613934	3.349675	-4.220343	C	4.848176	-1.380434	2.632842
H	3.605419	2.948280	-5.644536	C	4.104301	0.450690	1.249830
N	1.580453	-0.322672	-0.833613	C	5.533794	-0.493054	3.458733
N	1.859347	1.217971	0.676936	H	6.087224	-0.861312	4.317185
C	-1.641799	-1.333820	2.287241	C	4.799333	1.336401	2.074818
C	-1.135085	-2.011047	1.177683	C	5.509210	0.870315	3.176826

H	4.769905	2.402228	1.862200	C	-6.282068	0.727191	-2.376748
H	6.041568	1.570127	3.813263	H	-6.157138	1.419490	-3.213207
C	-2.029827	1.648515	0.958464	H	-7.238020	0.208596	-2.492662
C	-2.535632	1.703487	-0.439707	H	-6.311675	1.313632	-1.454167
C	2.168642	4.066318	0.170400	C	-5.093485	-1.061181	-3.656397
H	1.821440	5.089041	0.015700	H	-5.121300	-0.385407	-4.515451
H	3.264924	4.098202	0.180811	H	-4.197878	-1.679992	-3.737842
H	1.867689	3.471605	-0.692200	H	-5.970969	-1.713913	-3.706546
C	1.464197	0.248759	3.437926	C	-6.365754	-1.406780	-0.414707
H	2.464712	0.116546	3.866179	H	-6.888949	-0.492319	-0.130984
H	0.732492	-0.040178	4.197287	H	-6.995981	-1.975932	-1.106413
H	1.387717	-0.442796	2.602517	H	-6.215833	-1.998803	0.489711
H	1.283250	5.476245	2.252031	C	-4.213838	-2.387609	-1.231578
H	0.627279	2.252740	4.982760	H	-3.237239	-2.185666	-1.682445
B	-3.567996	0.654502	-0.955276	H	-4.053658	-2.838637	-0.250604
O	-4.266454	-0.238303	-0.178764	H	-4.744814	-3.110164	-1.858136
O	-3.907229	0.537376	-2.294832	Cl	-0.567248	-4.614920	0.621734
C	-5.019140	-1.099428	-1.053377	Cl	0.963488	-3.099487	2.638411
C	-5.103746	-0.250410	-2.367290	C	-3.068627	-1.273702	2.676830

C	-3.909699	-2.359739	2.422047	H	-1.696841	4.523669	-3.190289
C	-3.562673	-0.204901	3.431769	H	-0.296150	3.470756	-3.138386
C	-5.225262	-2.361160	2.875197	C	-1.048082	6.165910	-0.353179
H	-3.512488	-3.224500	1.898177	H	-2.871545	5.199807	-0.996945
C	-4.879768	-0.198603	3.872307	H	-2.248587	4.584543	0.530783
H	-2.903974	0.627450	3.660150	C	-0.580073	6.646031	-1.731215
C	-5.719630	-1.273889	3.588867	H	0.328656	5.875422	-3.541967
H	-5.861308	-3.219822	2.679678	H	1.048923	5.260229	-2.065362
H	-5.252095	0.646312	4.443413	H	-1.574407	6.974988	0.166200
H	-6.747173	-1.271644	3.939036	H	-0.178028	5.913466	0.267037
H	-2.854508	1.473652	1.652720	H	0.100206	7.499370	-1.628958
C	-2.173943	2.602405	-1.386537	H	-1.453285	7.005737	-2.293046
H	-1.482858	2.548833	1.257025	K	2.406842	-5.605010	0.161554
H	-2.620264	2.501302	-2.375981	C	0.357868	4.958420	4.760062
C	-1.259515	3.784930	-1.223751	H	0.495066	4.570982	5.773181
C	-0.799025	4.283826	-2.601693	H	0.886582	5.912697	4.684026
C	-1.948966	4.933794	-0.462016	H	-0.710028	5.162615	4.625405
H	-0.380015	3.483610	-0.635088				
C	0.092088	5.523983	-2.530628				

TS'$_{OA}$-*Z*,*R*

Imaginary Freq = -197.1635 cm^{-1}

Electronic Energy BS1 = -4720.17996432 Hartree

Electronic Energy BS2 = -6164.53274252 Hartree

Zero-point Energy Correction = 1.059349 Hartree

Thermal Correction to Enthalpy = 1.124985 Hartree

Thermal Correction to Free Energy = 0.958390 Hartree

Chemical symbol X, Y, Z

C	2.957064	-0.514513	-1.078913
H	3.413112	-1.503254	-1.103320
C	3.417115	0.274005	0.165894
H	4.073211	1.092494	-0.134491
C	1.066989	0.180278	0.152681
C	0.610510	-1.181806	-1.799175
C	-0.404472	-0.345856	-2.269770
C	0.709285	-2.484066	-2.319422
C	-1.320722	-0.779830	-3.217902
H	-0.471382	0.668106	-1.892575
C	-0.206576	-2.904297	-3.284220
C	-1.220306	-2.066204	-3.732390
H	-2.096152	-0.093480	-3.541047
H	-0.112448	-3.914648	-3.664078
H	-1.921938	-2.419628	-4.480975
S	1.935485	-3.704220	-1.810592
O	1.422744	-4.998674	-2.321079
O	1.873917	-3.690527	-0.315681
O	3.230561	-3.295517	-2.354673
Cu	-0.765254	0.187817	0.967571
C	3.257311	0.191334	-2.384003
C	4.269837	-0.302947	-3.204230
C	2.572389	1.347741	-2.766047
C	4.617483	0.368714	-4.373437
H	4.763357	-1.231434	-2.935594
C	2.921709	2.020964	-3.930909
H	1.750930	1.717635	-2.157327
C	3.950808	1.535066	-4.734627
H	5.404843	-0.027350	-5.006917
H	2.388609	2.923459	-4.213989
H	4.221652	2.058512	-5.646196

N	1.501615	-0.639203	-0.827118	C	5.136710	-1.801095	3.519443
N	2.130229	0.821844	0.661810	H	5.546473	-2.304744	4.389605
C	-1.954900	-0.850498	2.233665	C	4.945281	0.164796	2.141264
C	-1.585481	-1.724178	1.182085	C	5.472266	-0.474885	3.258560
C	-0.613973	-2.753135	1.205204	H	5.197399	1.203921	1.944025
H	-0.045217	-3.009630	0.321043	H	6.143395	0.060578	3.922694
H	-2.233223	-1.779541	0.307945	C	-1.576366	2.005723	0.987505
H	-1.274166	-0.825451	3.088169	C	-2.043760	2.193038	-0.411585
C	2.084209	1.802971	1.707123	C	3.170067	3.488623	0.125144
C	2.505177	3.113828	1.422234	H	3.104977	4.565087	-0.041875
C	1.661990	1.462642	2.999427	H	4.236171	3.233000	0.149328
C	2.364892	4.089437	2.406602	H	2.734113	2.982547	-0.736197
C	1.518126	2.478581	3.945038	C	1.462592	0.039962	3.434613
C	1.837427	3.802044	3.663660	H	2.382156	-0.330989	3.902646
H	3.063143	-2.382329	0.892962	H	0.654454	-0.041884	4.166526
H	4.012734	-3.512800	2.853942	H	1.249155	-0.628664	2.605568
C	3.744569	-1.838644	1.542834	H	2.677179	5.106224	2.179811
C	4.278262	-2.478614	2.657252	H	1.163797	2.213881	4.938690
C	4.077108	-0.508746	1.280257	B	-3.286556	1.420909	-0.957633

O	-4.169377	0.687573	-0.209287	H	-5.250501	-1.961253	-1.974650
O	-3.626848	1.429855	-2.298437	Cl	-1.819622	-4.667841	0.995532
C	-5.085904	0.036491	-1.114304	Cl	0.327126	-3.049839	2.637800
C	-4.966369	0.920561	-2.402852	C	-3.352955	-0.544509	2.601022
C	-5.903225	2.131005	-2.392771	C	-4.359685	-1.476839	2.331434
H	-5.620980	2.800994	-3.208884	C	-3.670301	0.590412	3.354780
H	-6.947077	1.835401	-2.529853	C	-5.661586	-1.256572	2.768354
H	-5.817791	2.685672	-1.454130	H	-4.104125	-2.400562	1.819742
C	-5.112348	0.161839	-3.714785	C	-4.974705	0.819749	3.773926
H	-4.978719	0.849679	-4.554073	H	-2.883759	1.293137	3.613024
H	-4.373007	-0.636107	-3.804475	C	-5.977829	-0.099398	3.474576
H	-6.110258	-0.281400	-3.791902	H	-6.428565	-1.998565	2.566160
C	-6.474425	0.018605	-0.492913	H	-5.207863	1.712921	4.345314
H	-6.783886	1.017139	-0.180213	H	-6.995329	0.074495	3.810566
H	-7.206105	-0.371739	-1.208128	H	-2.413344	2.009257	1.684353
H	-6.473670	-0.622455	0.389889	C	-1.476880	3.011774	-1.328279
C	-4.581348	-1.391407	-1.323813	H	-0.809258	2.719057	1.302116
H	-3.578999	-1.398884	-1.762971	H	-1.938738	3.041743	-2.315307
H	-4.537095	-1.896548	-0.357308	C	-0.311740	3.946344	-1.156856

C	0.222765	4.355859	-2.538991
C	-0.711571	5.204620	-0.360606
H	0.484850	3.438545	-0.592181
C	1.361945	5.372938	-2.476657
H	-0.612819	4.794242	-3.104056
H	0.527137	3.462158	-3.096489
C	0.441847	6.205198	-0.262966
H	-1.566404	5.677324	-0.864133
H	-1.054547	4.913069	0.636972
C	0.966688	6.599420	-1.647220
H	1.642946	5.680692	-3.490531
H	2.250071	4.901963	-2.040997
H	0.119658	7.097952	0.284821
H	1.250668	5.753770	0.325352
H	1.817298	7.283867	-1.552832
H	0.178474	7.148984	-2.180143
None	0.651882	-6.036573	-0.018619
C	1.625059	4.891813	4.681534
H	1.647316	4.496592	5.700534
H	2.389667	5.669224	4.599106
H	0.651286	5.371570	4.533726

III'-*Z,R*

Electronic Energy BS1 = -4720.19340421 Hartree

Electronic Energy BS2 = -6164.55429036 Hartree

Zero-point Energy Correction = 1.059626 Hartree

Thermal Correction to Enthalpy = 1.126539 Hartree

Thermal Correction to Free Energy = 0.953789 Hartree

Chemical symbol X, Y, Z

C	-3.092995	-0.573459	0.851553
H	-3.596260	-1.521577	0.662721
C	-3.448333	0.465496	-0.235242
H	-4.054628	1.266545	0.191022
C	-1.123363	0.179966	-0.186259
C	-0.826299	-1.532236	1.507032
C	0.168241	-0.857451	2.214526
C	-1.001167	-2.908434	1.712247
C	1.005980	-1.526037	3.097606

H	0.280883	0.210049	2.072976	H	-5.658117	-0.809321	4.722524
C	-0.165013	-3.566600	2.612057	H	-2.498682	2.097534	4.660517
C	0.834909	-2.888896	3.300803	H	-4.416696	1.035087	5.824467
H	1.782927	-0.968287	3.610643	N	-1.637685	-0.748944	0.629891
H	-0.305007	-4.632738	2.743626	N	-2.108465	0.994119	-0.598960
H	1.480213	-3.431255	3.983606	C	2.022391	-0.180860	-2.530689
S	-2.200729	-3.923664	0.821917	C	1.900978	-1.342793	-1.729948
O	-1.823151	-5.316612	1.146075	C	0.718249	-2.048341	-1.615269
O	-1.924239	-3.615690	-0.608777	H	0.597660	-2.787051	-0.833771
O	-3.536637	-3.524450	1.276956	H	2.659362	-1.557166	-0.981740
Cu	0.690044	0.152529	-0.939983	H	1.215613	0.002130	-3.243926
C	-3.414137	-0.135492	2.263031	C	-1.932057	2.168532	-1.403817
C	-4.472702	-0.746945	2.932378	C	-2.206648	3.425363	-0.833013
C	-2.697601	0.878462	2.902918	C	-1.505646	2.079208	-2.735944
C	-4.834506	-0.323801	4.208885	C	-1.887892	4.571479	-1.557195
H	-4.991968	-1.574125	2.458683	C	-1.179055	3.255842	-3.413414
C	-3.059160	1.303951	4.175601	C	-1.330007	4.510049	-2.833330
H	-1.837242	1.324934	2.411223	H	-3.197109	-2.029703	-1.434810
C	-4.135233	0.706649	4.828920	H	-4.155661	-2.733393	-3.573640

C	-3.840218	-1.339981	-1.978391	H	-2.084231	5.542367	-1.108425
C	-4.375562	-1.738701	-3.199136	H	-0.821941	3.180090	-4.438133
C	-4.117149	-0.062977	-1.484998	B	3.239636	0.755556	1.221418
C	-5.179908	-0.873048	-3.936465	O	3.933662	-0.030720	0.341197
H	-5.592562	-1.190409	-4.889179	O	3.684852	0.612352	2.515207
C	-4.930330	0.799449	-2.221640	C	4.818250	-0.890619	1.111219
C	-5.459789	0.399576	-3.444735	C	4.929946	-0.109165	2.467306
H	-5.140366	1.796128	-1.840631	C	6.044482	0.939889	2.471696
H	-6.090810	1.078230	-4.010000	H	5.931246	1.571480	3.356421
C	1.523075	1.912346	-0.496923	H	7.034257	0.476347	2.500804
C	2.061001	1.730434	0.877842	H	5.985657	1.580877	1.587294
C	-2.909411	3.576659	0.489162	C	5.033191	-0.990590	3.703197
H	-2.748511	4.573920	0.901608	H	5.080698	-0.364347	4.598102
H	-3.991380	3.454359	0.361566	H	4.172419	-1.655382	3.791940
H	-2.587596	2.845010	1.229254	H	5.939994	-1.601977	3.664544
C	-1.500467	0.784762	-3.494643	C	6.141462	-1.038636	0.371664
H	-2.445702	0.683438	-4.040819	H	6.539971	-0.071718	0.058820
H	-0.689018	0.750103	-4.227234	H	6.875687	-1.534405	1.014460
H	-1.428661	-0.085243	-2.849530	H	6.008929	-1.664037	-0.514327

C	4.139567	-2.248881	1.249207	H	0.729455	2.657273	-0.580687
H	3.164330	-2.165267	1.734797	H	2.090218	2.222260	2.902650
H	3.976810	-2.718615	0.275305	C	0.490570	3.424643	2.010681
H	4.756832	-2.939514	1.830143	C	-0.052434	3.519343	3.445606
Cl	2.412101	-4.721320	-0.736906	C	0.998418	4.805333	1.546328
Cl	-0.334256	-2.324163	-2.974763	H	-0.324389	3.126436	1.334867
C	3.271233	0.510618	-2.852121	C	-1.128116	4.593391	3.604729
C	4.521432	-0.061225	-2.596440	H	0.789408	3.757109	4.111790
C	3.213001	1.766429	-3.473429	H	-0.426164	2.540006	3.766969
C	5.685203	0.617038	-2.932183	C	-0.075318	5.885554	1.690747
H	4.576442	-1.047220	-2.150656	H	1.875399	5.074944	2.150833
C	4.377862	2.447164	-3.803237	H	1.338917	4.737829	0.507765
H	2.241868	2.209492	-3.682464	C	-0.623709	5.956470	3.119421
C	5.617828	1.874435	-3.528497	H	-1.436324	4.659686	4.654249
H	6.648584	0.158842	-2.732238	H	-2.021171	4.303842	3.038529
H	4.319422	3.421722	-4.277114	H	0.332634	6.858513	1.395583
H	6.529768	2.401924	-3.789615	H	-0.891772	5.665719	0.992006
H	2.307420	2.102352	-1.225829	H	-1.423882	6.701992	3.182526
C	1.589647	2.404634	1.951588	H	0.177126	6.297139	3.789858

K	-0.264445	-5.766583	-0.986732
C	-0.921053	5.764647	-3.559286
H	-0.890040	5.608664	-4.640647
H	-1.610670	6.587604	-3.352162
H	0.077027	6.085414	-3.241089

II-*E,R*

Electronic Energy BS1 = -4720.19608527 Hartree

Electronic Energy BS2 = -6164.55269254 Hartree

Zero-point Energy Correction = 1.059939 Hartree

Thermal Correction to Enthalpy = 1.125993 Hartree

Thermal Correction to Free Energy = 0.957301 Hartree

Chemical symbol X, Y, Z

H	3.529743	-1.648442	-0.135329
C	1.665911	-2.704717	-0.531718
H	1.266924	-3.593330	-0.027755
C	0.687517	-0.639155	0.062047
C	2.288050	0.177753	1.719157
C	1.510785	0.305111	2.872681
C	3.488740	0.892077	1.619457
C	1.932897	1.095751	3.931374
H	0.572246	-0.235556	2.924190
C	3.915483	1.669679	2.697281
C	3.152438	1.760782	3.853752
H	1.311844	1.184922	4.815957
H	4.848209	2.213890	2.601218
H	3.501139	2.365117	4.684822
S	4.561281	0.866223	0.173268
O	5.201819	2.208864	0.175683
O	3.646653	0.750930	-0.998354
O	5.474177	-0.264873	0.333770
Cu	-0.438854	0.959923	0.290711
C	3.063280	-2.713546	1.637588
C	4.405183	-3.054763	1.794422
C	2.144607	-3.079273	2.623884
C	4.818142	-3.779577	2.909843
H	5.126784	-2.721721	1.055114
C	2.556369	-3.797463	3.739724

H	1.105438	-2.780664	2.525870	C	2.271081	-3.130458	-1.848908
C	3.895862	-4.155713	3.880877	C	3.256590	-3.902206	-4.346739
H	5.865760	-4.039679	3.023626	H	3.641332	-4.200862	-5.317026
H	1.834231	-4.073610	4.501907	C	1.934195	-4.370261	-2.388114
H	4.219531	-4.716639	4.752222	C	2.421758	-4.757170	-3.633998
N	1.848880	-0.738678	0.725423	H	1.275500	-5.033537	-1.833598
N	0.553497	-1.734466	-0.706388	H	2.151008	-5.724917	-4.044425
C	-0.470454	2.958190	0.131864	C	-2.005506	0.795604	1.521060
C	0.576896	2.460216	-0.670137	C	-3.244379	0.301079	0.831703
C	-0.703730	-2.188921	-1.232465	C	-1.353790	-2.797903	1.143616
C	-1.642903	-2.720683	-0.329650	H	-2.209316	-3.208456	1.682002
C	-0.966998	-2.169809	-2.606705	H	-0.496687	-3.449347	1.341906
C	-2.851202	-3.200410	-0.820372	H	-1.135905	-1.808397	1.554694
C	-2.191684	-2.675165	-3.053710	C	0.014034	-1.659472	-3.623398
C	-3.143730	-3.192920	-2.183518	H	0.499092	-2.493614	-4.141845
H	3.362573	-1.293329	-2.169451	H	-0.493103	-1.044069	-4.372628
H	4.253100	-1.993828	-4.361139	H	0.791775	-1.062573	-3.157116
C	3.110693	-2.274326	-2.567730	H	-3.584951	-3.589113	-0.118754
C	3.600781	-2.663135	-3.808641	H	-2.398883	-2.657595	-4.121941

B	-3.610454	0.801633	-0.594699	H	-2.425544	3.633948	-2.592842
O	-2.705233	1.222732	-1.557214	H	-3.988569	3.714837	-3.410040
O	-4.903824	0.865785	-1.059478	H	-1.407008	3.179927	-0.376866
C	-3.446895	1.727976	-2.685582	C	0.377225	2.544402	-2.126850
C	-4.870954	1.094560	-2.474056	H	-0.655612	2.367524	-2.411172
C	-5.030559	-0.267614	-3.148920	H	1.603059	2.445713	-0.312137
H	-5.958274	-0.725366	-2.795828	C	2.633228	-1.947474	0.408785
H	-5.079809	-0.180027	-4.238259	Cl	0.737332	4.275954	-2.743475
H	-4.202885	-0.928527	-2.884783	Cl	1.392204	1.455982	-3.116682
C	-6.035481	2.001148	-2.852254	C	-0.304152	3.659518	1.418749
H	-6.977258	1.480147	-2.661858	C	-1.417379	4.292924	1.987299
H	-6.035619	2.922022	-2.266878	C	0.920168	3.739806	2.090627
H	-5.996886	2.257987	-3.915864	C	-1.312987	4.984481	3.188051
C	-2.755676	1.285847	-3.969974	H	-2.374422	4.239487	1.474937
H	-2.569803	0.210260	-3.960768	C	1.025740	4.435265	3.289021
H	-3.369533	1.535179	-4.841315	H	1.798797	3.242307	1.690808
H	-1.794461	1.794407	-4.090075	C	-0.087433	5.061038	3.844289
C	-3.451444	3.254363	-2.576475	H	-2.189597	5.466169	3.610028
H	-3.916118	3.576962	-1.640248	H	1.985878	4.479922	3.793669

H	-0.000210	5.604041	4.780093	H	-3.745651	-3.399066	4.455006
H	-1.763790	0.178720	2.397063	H	-5.194367	-2.555790	6.281221
H	-2.191756	1.802640	1.913702	H	-6.344687	-1.921894	5.108044
C	-4.109506	-0.592475	1.356632	None	3.904215	3.193238	-1.905715
H	-4.981624	-0.873587	0.763855	C	-4.456468	-3.736507	-2.685471
C	-4.046556	-1.199865	2.733238	H	-4.486022	-4.828194	-2.602130
C	-4.557379	-0.203974	3.792891	H	-5.292214	-3.339336	-2.101361
C	-4.836796	-2.511898	2.821081	H	-4.622191	-3.475395	-3.733980
H	-3.000829	-1.420644	2.988717				
C	-4.503858	-0.802333	5.200972				
H	-5.592183	0.073505	3.548151				
H	-3.961010	0.712601	3.737799				
C	-4.783236	-3.117790	4.225315				
H	-5.883649	-2.312824	2.549003				
H	-4.458518	-3.229618	2.082270				
C	-5.277692	-2.121701	5.278552				
H	-4.897718	-0.087529	5.932199				
H	-3.454456	-0.984603	5.472633				
H	-5.374835	-4.039289	4.265350				

TS_{OA}-*E*,*R*

Imaginary Freq = -124.4661 cm^{-1}

Electronic Energy BS1 = -4720.18718808 Hartree

Electronic Energy BS2 = -6164.54486090 Hartree

Zero-point Energy Correction = 1.058854 Hartree

Thermal Correction to Enthalpy = 1.124954 Hartree

Thermal Correction to Free Energy = 0.955680 Hartree

Chemical symbol X, Y, Z

H	3.449843	-1.559454	-0.789153
C	1.587880	-2.476092	-1.461480

H	1.238119	-3.502348	-1.296196	C	2.216495	-3.920861	1.329503
C	0.604597	-0.783090	-0.149306	C	4.929590	-4.517884	1.333151
C	2.259706	-0.537076	1.635818	H	5.130901	-2.865257	-0.039712
C	1.519265	-0.845784	2.778867	C	2.695760	-4.955954	2.122582
C	3.446818	0.196234	1.762399	H	1.160688	-3.668659	1.350659
C	1.962070	-0.467925	4.038181	C	4.055716	-5.261137	2.119744
H	0.597131	-1.404301	2.659924	H	5.992071	-4.739195	1.340986
C	3.899388	0.545190	3.035637	H	2.010758	-5.521869	2.746399
C	3.171993	0.205736	4.169058	H	4.432436	-6.069500	2.738674
H	1.370599	-0.714332	4.913258	N	1.796756	-1.056116	0.394648
H	4.819629	1.113130	3.113186	N	0.444077	-1.553920	-1.233583
H	3.540335	0.484504	5.150872	C	-0.385001	2.664216	0.983679
S	4.445268	0.759653	0.367907	C	0.643900	2.420781	0.011981
O	5.098369	1.992450	0.872357	C	-0.841603	-1.806704	-1.822221
O	3.460935	1.100707	-0.697001	C	-1.748878	-2.603455	-1.099453
O	5.346981	-0.338575	0.018144	C	-1.178078	-1.303310	-3.083327
Cu	-0.471975	0.732006	0.529085	C	-2.995837	-2.870958	-1.650231
C	3.086814	-3.182307	0.524902	C	-2.440875	-1.609472	-3.600280
C	4.448778	-3.476770	0.542528	C	-3.361215	-2.385204	-2.905724

H	3.141618	-0.478493	-2.531711	H	-0.794034	0.321771	-4.431132
H	3.940536	-0.288562	-4.854370	H	0.517573	0.004506	-3.288384
C	2.906627	-1.263237	-3.248645	H	-3.702870	-3.473163	-1.085090
C	3.346073	-1.148382	-4.561857	H	-2.707012	-1.215948	-4.579172
C	2.139432	-2.366921	-2.863491	B	-3.616776	1.017449	-0.207486
C	3.023699	-2.126741	-5.501259	O	-2.717279	1.671266	-1.027362
H	3.368459	-2.031824	-6.526227	O	-4.916338	1.248225	-0.577655
C	1.825011	-3.345691	-3.803650	C	-3.465943	2.529548	-1.917560
C	2.262833	-3.227804	-5.120592	C	-4.909861	1.905556	-1.856554
H	1.224152	-4.201208	-3.506264	C	-5.145493	0.822133	-2.908592
H	2.011301	-3.995531	-5.845482	H	-6.084684	0.311586	-2.680180
C	-1.974299	0.405209	1.775049	H	-5.215995	1.242334	-3.915896
C	-3.213084	0.108481	0.990419	H	-4.342243	0.082676	-2.889988
C	-1.387062	-3.171876	0.245465	C	-6.044296	2.920888	-1.890537
H	-2.219542	-3.741536	0.661674	H	-7.002373	2.397801	-1.835043
H	-0.529589	-3.847704	0.169360	H	-5.988923	3.614862	-1.050479
H	-1.131851	-2.378813	0.954938	H	-6.021624	3.493426	-2.823226
C	-0.243796	-0.463837	-3.905916	C	-2.830549	2.484330	-3.300987
H	0.268931	-1.075283	-4.655902	H	-2.702174	1.453924	-3.636855

H	-3.453637	3.019643	-4.024203	H	1.932014	2.307018	2.450435
H	-1.849028	2.966051	-3.295440	C	0.301523	3.697875	5.077058
C	-3.398492	3.945190	-1.341505	H	-1.742838	4.374295	5.051245
H	-3.821313	3.977677	-0.332919	H	2.296863	2.939372	4.784675
H	-2.359781	4.284351	-1.285179	H	0.467399	3.972932	6.113813
H	-3.941392	4.657042	-1.968696	H	-1.665430	-0.422709	2.423204
H	-1.296025	3.108586	0.588934	H	-2.110028	1.285436	2.407249
C	0.410522	2.825987	-1.293539	C	-4.040117	-0.924999	1.251145
H	-0.576469	3.138688	-1.599136	H	-4.930050	-1.032550	0.629095
H	1.642307	2.091311	0.283931	C	-3.912573	-1.917969	2.375366
C	2.584838	-2.059028	-0.351031	C	-4.346932	-1.293113	3.716400
Cl	1.070246	5.221493	-1.238445	C	-4.720990	-3.194177	2.107912
Cl	1.401343	2.349356	-2.621986	H	-2.857513	-2.206606	2.488185
C	-0.116922	2.994390	2.392534	C	-4.215984	-2.292414	4.868646
C	-1.141622	3.575822	3.151839	H	-5.390820	-0.961527	3.630135
C	1.121826	2.770467	3.003855	H	-3.745938	-0.398644	3.910266
C	-0.936978	3.922127	4.481845	C	-4.588298	-4.200926	3.252328
H	-2.103906	3.766990	2.684245	H	-5.778704	-2.921197	1.982055
C	1.327794	3.122342	4.331565	H	-4.406185	-3.648293	1.160607

C	-5.000859	-3.577384	4.589027
H	-4.555573	-1.835898	5.804795
H	-3.154398	-2.541814	5.007013
H	-5.190691	-5.092336	3.045876
H	-3.542913	-4.534920	3.317030
H	-4.859318	-4.296076	5.403618
H	-6.073639	-3.342390	4.557484
K	3.708201	3.836441	-0.557905
C	-4.720977	-2.693872	-3.476430
H	-4.821268	-3.761893	-3.696172
H	-5.510304	-2.428960	-2.765955
H	-4.900717	-2.141031	-4.401930

III-*E,R*

Electronic Energy BS1 = -4720.21018509 Hartree

Electronic Energy BS2 = -6164.56963733 Hartree

Zero-point Energy Correction = 1.059356 Hartree

Thermal Correction to Enthalpy = 1.126104 Hartree

Thermal Correction to Free Energy = 0.955064 Hartree

Chemical symbol X, Y, Z

H	-3.005162	0.587775	2.625222
C	-1.109873	1.692122	2.536082
H	-0.370004	1.679527	3.342535
C	-0.751012	0.516136	0.529921
C	-2.192050	-1.427579	0.769810
C	-1.437796	-2.585125	0.945392
C	-3.449183	-1.499896	0.161425
C	-1.904768	-3.804747	0.466289
H	-0.487819	-2.522951	1.462233
C	-3.911025	-2.722507	-0.311660
C	-3.135889	-3.869824	-0.172817
H	-1.295066	-4.694365	0.581089
H	-4.876259	-2.757738	-0.801482
H	-3.497481	-4.810398	-0.573661
S	-4.480093	-0.038912	-0.035561
O	-5.674493	-0.515496	-0.766833
O	-3.668995	0.887627	-0.869702
O	-4.749039	0.446743	1.326452

Cu	0.101113	-0.337539	-0.986769	C	2.603072	3.758726	1.019204
C	-1.550849	-0.631642	3.612613	C	0.948642	4.567223	-0.506025
C	-2.554969	-1.386458	4.217002	C	2.209465	4.700239	0.069923
C	-0.215087	-0.921129	3.903110	H	-3.671307	2.309845	1.627355
C	-2.231845	-2.412046	5.101802	H	-4.778161	4.495352	1.718532
H	-3.593150	-1.178667	3.972896	C	-3.154979	3.147072	2.088604
C	0.109928	-1.947303	4.782770	C	-3.780450	4.389904	2.132488
H	0.580615	-0.351716	3.431607	C	-1.875251	2.994885	2.625227
C	-0.899585	-2.696025	5.384455	C	-3.134596	5.488458	2.693519
H	-3.022933	-2.993550	5.564130	H	-3.627294	6.455562	2.720922
H	1.151726	-2.164913	4.997386	C	-1.234689	4.097954	3.193413
H	-0.646279	-3.498285	6.070209	C	-1.855877	5.341629	3.224433
N	-1.660589	-0.175849	1.213590	H	-0.233972	3.984344	3.603896
N	-0.382001	1.580446	1.242519	H	-1.344882	6.191444	3.665886
C	0.789245	-1.452681	-2.526805	C	1.480596	-1.196034	0.136227
C	-0.380740	-0.781654	-3.035339	C	2.887707	-0.667670	-0.009791
C	0.506701	2.606059	0.798673	C	2.238873	1.727453	2.440033
C	1.773804	2.703267	1.392044	H	3.329237	1.721743	2.504695
C	0.070781	3.542682	-0.149642	H	1.848476	1.987110	3.431270

H	1.918973	0.711833	2.203996	H	5.969909	2.135978	-4.068950
C	-1.317996	3.536123	-0.731669	C	2.842471	3.042093	-3.281673
H	-1.945766	4.240082	-0.175668	H	2.737837	3.635329	-2.373042
H	-1.291948	3.864543	-1.773838	H	3.513566	3.556096	-3.976909
H	-1.814577	2.565101	-0.692263	H	1.857951	2.975743	-3.754748
H	3.582359	3.845892	1.484467	C	3.234063	0.745157	-4.195918
H	0.619658	5.299060	-1.240591	H	3.609913	-0.262312	-3.993605
B	3.373461	0.302659	-1.123961	H	2.180849	0.665722	-4.479835
O	2.550065	1.071514	-1.920970	H	3.783490	1.152758	-5.048440
O	4.698472	0.488368	-1.426741	H	1.733816	-0.946058	-2.689621
C	3.365756	1.649035	-2.968675	C	-0.426625	0.587018	-2.984316
C	4.797294	1.607979	-2.328497	H	0.443553	1.207388	-2.781536
C	5.110424	2.840849	-1.482186	H	-1.289253	-1.337529	-3.309564
H	6.023434	2.653935	-0.911238	C	-1.935635	0.383917	2.560597
H	5.268656	3.724740	-2.106765	Cl	-3.211328	-2.450237	-4.011341
H	4.300394	3.046649	-0.778749	Cl	-1.762186	1.498660	-3.605627
C	5.927886	1.345299	-3.313186	C	0.885140	-2.916104	-2.406179
H	6.881721	1.333671	-2.779876	C	2.143221	-3.474347	-2.129616
H	5.809822	0.384234	-3.815893	C	-0.227476	-3.762049	-2.504842

C	2.289577	-4.843927	-1.947927	C	4.351579	-2.388592	4.484475
H	3.008879	-2.821986	-2.038778	H	5.605279	-1.673038	2.876815
C	-0.071049	-5.133063	-2.333694	H	4.495258	-0.457987	3.504864
H	-1.216150	-3.369129	-2.734510	C	4.391563	-3.881020	4.142678
C	1.178573	-5.678622	-2.049195	H	3.490537	-5.285228	2.754978
H	3.269219	-5.257810	-1.730040	H	2.386517	-4.051270	3.353352
H	-0.940523	-5.776178	-2.426275	H	5.096235	-2.153687	5.252441
H	1.288645	-6.750206	-1.913935	H	3.371974	-2.142282	4.916674
H	1.110475	-1.034462	1.153305	H	4.168027	-4.478789	5.032854
H	1.421430	-2.265857	-0.070270	H	5.410057	-4.149204	3.830184
C	3.818449	-1.003355	0.906967	K	-4.724098	-0.131001	-3.212091
H	4.833271	-0.628508	0.767204	C	3.133016	5.815915	-0.346668
C	3.595817	-1.862122	2.119430	H	2.587209	6.753950	-0.480235
C	3.679415	-3.362786	1.773355	H	3.920427	5.980067	0.393265
C	4.580823	-1.520095	3.245540	H	3.621006	5.582899	-1.300555
H	2.576757	-1.683190	2.497640				
C	3.417167	-4.223103	3.011627				
H	4.681692	-3.579030	1.378193				
H	2.968140	-3.604680	0.976776				

II-*E,S*

Electronic Energy BS1 = -4720.21138985 Hartree

Electronic Energy BS2 = -6164.56587898 Hartree

Zero-point Energy Correction = 1.058765 Hartree

Thermal Correction to Enthalpy = 1.125572 Hartree

Thermal Correction to Free Energy = 0.953310 Hartree

Chemical symbol X, Y, Z

C	-2.649825	-0.002097	1.735127
H	-3.338280	-0.842519	1.672767
C	-3.088397	1.140287	0.788362
H	-3.074450	2.096053	1.326993
C	-0.984621	0.315874	0.084332
C	-0.529341	-1.414385	1.719224
C	0.729368	-1.042676	2.180746
C	-0.919237	-2.759614	1.783374
C	1.615548	-1.988929	2.680983
H	1.024926	-0.002376	2.118537
C	-0.041675	-3.698925	2.321531
C	1.222775	-3.320663	2.761506
H	2.606627	-1.673570	2.991370
H	-0.359718	-4.734294	2.359200
H	1.898137	-4.069440	3.163593
S	-2.486613	-3.375364	1.151718
O	-2.291790	-4.842298	1.016727
O	-2.643725	-2.761172	-0.206583
O	-3.542884	-2.983970	2.087241
Cu	0.720119	0.310596	-0.911258
C	-2.517608	0.377428	3.191708
C	-3.212562	-0.362153	4.147326
C	-1.690878	1.426577	3.601915
C	-3.104057	-0.042510	5.498478
H	-3.817879	-1.204233	3.825546
C	-1.582980	1.744076	4.950981
H	-1.117584	1.988975	2.866504
C	-2.293411	1.012636	5.901164
H	-3.649072	-0.624731	6.234842
H	-0.942174	2.560821	5.264012
H	-2.205298	1.263002	6.953858
N	-1.348171	-0.399621	1.155969
N	-1.988227	1.157037	-0.203578
C	1.537515	-0.423746	-2.662864

C	0.189625	-0.761686	-2.603353	C	-6.342849	2.062462	-0.922642
C	-0.388628	-2.067207	-2.226885	H	-4.697940	3.115403	-0.025144
H	-1.284734	-1.998740	-1.611484	H	-6.858052	2.970531	-1.219383
H	-0.536991	-0.149176	-3.129532	C	2.241256	1.483907	-0.304898
H	2.276811	-1.136962	-2.303600	C	3.040203	0.992816	0.847313
C	-1.921663	2.082452	-1.290186	C	-0.817151	3.718661	0.287153
C	-1.319776	3.332315	-1.076818	H	-0.160041	4.589180	0.230020
C	-2.421250	1.722401	-2.548099	H	-1.643969	3.965133	0.963838
C	-1.192034	4.202858	-2.154612	H	-0.251204	2.900137	0.735901
C	-2.277571	2.632373	-3.598366	C	-3.106664	0.405305	-2.803714
C	-1.661311	3.868342	-3.425087	H	-4.193636	0.534980	-2.783453
H	-4.477869	-1.176247	0.095397	H	-2.835003	0.011915	-3.788254
H	-6.649133	-1.314685	-1.038012	H	-2.859600	-0.340498	-2.046571
C	-5.002645	-0.260029	-0.157838	H	-0.701394	5.160994	-2.001483
C	-6.223881	-0.339960	-0.819700	H	-2.648945	2.354930	-4.582506
C	-4.447307	0.987122	0.137669	B	3.979877	-0.226153	0.636940
C	-6.895017	0.816795	-1.208091	O	4.271465	-0.798532	-0.585170
H	-7.845859	0.748227	-1.727573	O	4.648578	-0.860609	1.666005
C	-5.129575	2.142541	-0.246883	C	5.042655	-1.989579	-0.345739

C	5.631780	-1.728137	1.084352	C	2.081301	0.688338	-3.469051
C	6.952784	-0.955902	1.057637	C	3.470336	0.801751	-3.598169
H	7.194029	-0.636279	2.074442	C	1.271221	1.655421	-4.077848
H	7.774394	-1.572585	0.681832	C	4.033987	1.840898	-4.330919
H	6.871147	-0.061529	0.433567	H	4.104904	0.084693	-3.085184
C	5.766737	-2.968715	1.957206	C	1.836119	2.694697	-4.805047
H	6.172179	-2.687753	2.932817	H	0.192056	1.620298	-3.966900
H	4.798817	-3.447890	2.116823	C	3.219916	2.790614	-4.940809
H	6.447807	-3.693711	1.499928	H	5.113913	1.912756	-4.417748
C	6.080203	-2.129989	-1.451512	H	1.189310	3.436154	-5.265158
H	6.687776	-1.228453	-1.546366	H	3.659107	3.603866	-5.510216
H	6.740870	-2.980983	-1.256607	H	2.882622	1.557310	-1.187559
H	5.576859	-2.302975	-2.407076	C	3.025525	1.515988	2.094644
C	4.075559	-3.172372	-0.382414	H	1.820741	2.480840	-0.127608
H	3.301552	-3.071175	0.384521	H	3.657985	1.065008	2.860282
H	3.584503	-3.196493	-1.358192	C	2.241738	2.727948	2.519323
H	4.598497	-4.122141	-0.236466	C	1.919586	2.724386	4.018833
Cl	0.714361	-3.169188	-1.355006	C	2.987809	4.020371	2.130945
Cl	-1.002512	-2.971920	-3.754899	H	1.283034	2.731950	1.981893

C	1.161953	3.987278	4.438223
H	2.859119	2.660362	4.586748
H	1.337655	1.829333	4.271093
C	2.230708	5.279226	2.558503
H	3.975817	4.007874	2.612337
H	3.164521	4.020677	1.050457
C	1.924701	5.258464	4.057698
H	0.963984	3.972133	5.516727
H	0.183523	4.003698	3.935110
H	2.804996	6.175268	2.297233
H	1.284092	5.338281	2.002638
H	1.351395	6.146814	4.345802
H	2.868566	5.293640	4.619003
None	-2.185047	-5.006072	-1.559487
C	-1.509927	4.830615	-4.575172
H	-2.289644	5.599755	-4.547842
H	-0.543824	5.342087	-4.535141
H	-1.585347	4.316941	-5.537358

TS_{OA}-*E,S*

Imaginary Freq = -220.8972 cm^{-1}

Electronic Energy BS1 = -4720.19552887 Hartree

Electronic Energy BS2 = -6164.54884471 Hartree

Zero-point Energy Correction = 1.058864 Hartree

Thermal Correction to Enthalpy = 1.124825 Hartree

Thermal Correction to Free Energy = 0.956721 Hartree

Chemical symbol X, Y, Z

C	-2.607972	0.080899	1.766064
H	-3.349508	-0.704118	1.613322
C	-2.977971	1.341897	0.945999
H	-2.995846	2.232004	1.585646
C	-0.924688	0.478288	0.154015
C	-0.557399	-1.375117	1.722455
C	0.610842	-0.999831	2.377750
C	-0.972497	-2.711957	1.774783
C	1.355775	-1.922895	3.100018
H	0.930039	0.031099	2.313645
C	-0.240096	-3.629125	2.525370

C	0.911820	-3.236673	3.198633	H	-1.849281	0.570068	7.081108
H	2.276500	-1.607534	3.577189	N	-1.337604	-0.346031	1.121005
H	-0.581504	-4.657670	2.551967	N	-1.834351	1.454726	0.010145
H	1.469068	-3.962018	3.782790	C	1.446339	-0.044531	-2.795472
S	-2.421096	-3.322550	0.908687	C	0.069168	-0.306435	-3.006939
O	-2.179049	-4.779490	0.754425	C	-0.577999	-1.527877	-2.680914
O	-2.375431	-2.652426	-0.421584	H	-0.466310	-2.005633	-1.715692
O	-3.593113	-2.966381	1.713416	H	-0.480324	0.350389	-3.674308
Cu	0.636796	0.448001	-1.049327	H	2.085497	-0.872528	-2.494693
C	-2.428749	0.268479	3.255726	C	-1.572017	2.611898	-0.798480
C	-2.982859	-0.669273	4.126548	C	-0.746099	3.620629	-0.266924
C	-1.655203	1.307867	3.774900	C	-2.078021	2.717892	-2.099971
C	-2.777324	-0.556756	5.499682	C	-0.409798	4.706601	-1.066751
H	-3.551593	-1.500034	3.717561	C	-1.722496	3.836432	-2.861345
C	-1.446459	1.419266	5.144079	C	-0.884694	4.831093	-2.372943
H	-1.196006	2.026527	3.104861	H	-4.040886	-0.832574	-0.368977
C	-2.011585	0.486172	6.011012	H	-6.144591	-0.927939	-1.635449
H	-3.212050	-1.292041	6.169393	C	-4.676179	0.049719	-0.418688
H	-0.838033	2.230806	5.531683	C	-5.857342	-0.001313	-1.148796

C	-4.296265	1.238567	0.211763	B	3.474684	-0.916546	0.429933
C	-6.665509	1.128722	-1.262875	O	3.286385	-1.550752	-0.778895
H	-7.586660	1.084082	-1.835608	O	4.061488	-1.745409	1.357451
C	-5.109343	2.364417	0.099418	C	3.515340	-2.958021	-0.574312
C	-6.290219	2.313790	-0.637397	C	4.457424	-2.952259	0.677999
H	-4.812593	3.290778	0.584729	C	5.934681	-2.796852	0.313577
H	-6.915806	3.197093	-0.718966	H	6.504103	-2.595886	1.224503
C	2.351562	1.282655	-0.401753	H	6.331080	-3.703386	-0.152539
C	3.033648	0.552081	0.704429	H	6.083167	-1.957542	-0.371636
C	-0.228777	3.548216	1.143353	C	4.265787	-4.131500	1.620353
H	0.544239	4.301764	1.309104	H	4.949485	-4.040715	2.468770
H	-1.031184	3.737521	1.865328	H	3.245156	-4.166083	2.004247
H	0.204768	2.569649	1.366021	H	4.482605	-5.073316	1.105949
C	-2.974474	1.681096	-2.712019	C	4.116462	-3.548262	-1.841363
H	-4.027489	1.949220	-2.576370	H	5.012627	-3.006497	-2.149617
H	-2.785179	1.596094	-3.785881	H	4.379464	-4.599854	-1.686551
H	-2.825995	0.704348	-2.257879	H	3.382631	-3.495210	-2.650865
H	0.247989	5.472662	-0.663169	C	2.147582	-3.586287	-0.298919
H	-2.104171	3.911698	-3.877010	H	1.681442	-3.142858	0.583585

H	1.511074	-3.404556	-1.169280	C	2.998180	2.396010	2.494095
H	2.225765	-4.667574	-0.147969	C	2.107846	2.302232	3.747240
Cl	0.422357	-3.278947	-3.658412	C	4.300157	3.146030	2.830622
Cl	-2.217682	-1.675737	-3.272952	H	2.466504	2.989977	1.742191
C	2.121148	1.051532	-3.521963	C	1.834098	3.674938	4.366854
C	3.467840	0.910627	-3.869170	H	2.607543	1.661334	4.488188
C	1.465104	2.248152	-3.839605	H	1.160863	1.807120	3.505707
C	4.138813	1.930780	-4.537518	C	4.021781	4.524398	3.435792
H	3.986905	-0.006655	-3.606220	H	4.883130	2.544987	3.543434
C	2.135513	3.265520	-4.504309	H	4.908914	3.238891	1.925019
H	0.433110	2.401267	-3.535990	C	3.134992	4.419113	4.679253
C	3.474883	3.110011	-4.860185	H	1.233382	3.563791	5.277188
H	5.183500	1.803484	-4.803845	H	1.235136	4.274001	3.666784
H	1.611209	4.187406	-4.738484	H	4.963802	5.026548	3.682075
H	3.998287	3.907027	-5.378957	H	3.518104	5.150677	2.685968
H	3.017113	1.346899	-1.263644	H	2.917105	5.415345	5.079638
C	3.299739	1.034970	1.937251	H	3.679371	3.874872	5.463204
H	2.048898	2.302719	-0.138200	K	-1.610752	-4.774752	-1.847827
H	3.815178	0.372316	2.636825	C	-0.486957	6.011564	-3.220477

H	-0.902788	6.941896	-2.819613
H	0.601600	6.124167	-3.243793
H	-0.840471	5.902352	-4.248902

III-*E,S*

Electronic Energy BS1 = -4720.21300375 Hartree

Electronic Energy BS2 = -6164.57201235 Hartree

Zero-point Energy Correction = 1.059504 Hartree

Thermal Correction to Enthalpy = 1.126665 Hartree

Thermal Correction to Free Energy = 0.954083 Hartree

Chemical symbol X, Y, Z

C	-1.877971	-1.767770	1.754629
H	-1.719595	-2.840580	1.642145
C	-2.940827	-1.267065	0.762449
H	-3.627569	-0.564360	1.248699
C	-0.825562	-0.404831	0.135231
C	0.585402	-1.311274	1.954962
C	1.006820	-0.344241	2.860956
C	1.290748	-2.516765	1.847890
C	2.121444	-0.562503	3.662853
H	0.442101	0.575570	2.935093
C	2.387305	-2.742642	2.674015
C	2.798433	-1.773397	3.584480
H	2.450973	0.212682	4.345400
H	2.917071	-3.682999	2.574700
H	3.657609	-1.962269	4.220160
S	0.828515	-3.812317	0.687195
O	1.998505	-4.719525	0.655593
O	0.639141	-3.123320	-0.614509
O	-0.395496	-4.424220	1.230725
Cu	0.350356	0.689423	-1.037416
C	-2.116626	-1.484163	3.219273
C	-1.680793	-2.431131	4.148039
C	-2.684154	-0.295147	3.677964
C	-1.806224	-2.191220	5.512573
H	-1.232427	-3.353967	3.788895
C	-2.813774	-0.054957	5.042106
H	-3.025818	0.458362	2.973645

C	-2.372753	-1.002014	5.962452	H	-3.655341	-5.482005	-1.167259
H	-1.461510	-2.934653	6.224063	C	-3.269392	-3.592678	-0.233163
H	-3.260167	0.873060	5.385772	C	-4.057249	-4.501577	-0.932757
H	-2.473352	-0.813921	7.026736	C	-3.776271	-2.329265	0.080098
N	-0.642274	-1.094164	1.260038	C	-5.344261	-4.157576	-1.338962
N	-2.121236	-0.497134	-0.203190	H	-5.951179	-4.869871	-1.889271
C	1.142333	1.393737	-2.696775	C	-5.070830	-1.993709	-0.319968
C	0.605620	0.129165	-3.202548	C	-5.851817	-2.897896	-1.032290
C	1.316331	-1.017508	-3.123858	H	-5.465474	-1.008322	-0.083882
H	2.340816	-1.102631	-2.747287	H	-6.854766	-2.621286	-1.341621
H	-0.364503	0.130135	-3.693207	C	0.613819	2.569914	-0.397008
H	2.209769	1.381047	-2.473164	C	1.352180	2.502913	0.890145
C	-2.745957	0.268782	-1.236318	C	-3.102905	2.122070	0.459224
C	-3.233078	1.547369	-0.926360	H	-3.114517	3.214515	0.429311
C	-2.922896	-0.285469	-2.513336	H	-3.940013	1.806546	1.094005
C	-3.877316	2.278477	-1.923905	H	-2.180082	1.804554	0.949764
C	-3.571258	0.485087	-3.478641	C	-2.469882	-1.677149	-2.857308
C	-4.057730	1.763047	-3.205476	H	-3.306489	-2.375511	-2.753976
H	-2.258398	-3.877099	0.045509	H	-2.126366	-1.722601	-3.893735

H	-1.665745	-2.028492	-2.208654	H	5.283276	0.252807	-1.869585
H	-4.241747	3.276209	-1.691870	C	4.229336	-0.756111	0.338252
H	-3.701894	0.067870	-4.474550	H	3.463980	-0.859182	1.109975
B	2.791169	1.901172	0.856973	H	3.868916	-1.175761	-0.603927
O	3.336215	1.365316	-0.280251	H	5.111564	-1.330818	0.634263
O	3.634652	1.867916	1.933513	Cl	4.485080	-2.046722	-3.054690
C	4.575474	0.711394	0.110410	Cl	0.764789	-2.474365	-3.857963
C	4.933097	1.471245	1.434707	C	0.650430	2.641200	-3.325342
C	5.724678	2.757314	1.194411	C	1.557960	3.645460	-3.675786
H	5.764312	3.329985	2.124662	C	-0.714594	2.856490	-3.549855
H	6.746936	2.541053	0.873035	C	1.112257	4.832795	-4.250146
H	5.246012	3.377748	0.431383	H	2.618756	3.489121	-3.500715
C	5.620896	0.616347	2.487882	C	-1.158925	4.043248	-4.115658
H	5.831019	1.220291	3.375057	H	-1.436241	2.101245	-3.256350
H	4.991821	-0.224408	2.783017	C	-0.246341	5.035648	-4.471448
H	6.569759	0.228988	2.104058	H	1.829540	5.600217	-4.523141
C	5.591378	0.855914	-1.010466	H	-2.222833	4.196542	-4.270087
H	5.703304	1.898582	-1.316111	H	-0.593876	5.963356	-4.914841
H	6.566421	0.482253	-0.680872	H	1.149004	3.181423	-1.116343

C	0.910145	3.010070	2.058166
H	-0.429933	2.893770	-0.351870
H	1.588132	2.940796	2.912116
C	-0.383728	3.703298	2.354012
C	-1.149197	2.965254	3.464636
C	-0.140873	5.168278	2.762107
H	-1.012374	3.716395	1.456492
C	-2.459390	3.669494	3.820746
H	-0.511649	2.905525	4.358500
H	-1.343500	1.932601	3.158829
C	-1.453142	5.875649	3.111338
H	0.530244	5.189275	3.632384
H	0.375721	5.693450	1.951741
C	-2.217533	5.130626	4.209352
H	-2.962260	3.137338	4.635907
H	-3.136180	3.632987	2.955677
H	-1.253017	6.907020	3.420545
H	-2.080406	5.933446	2.210731
H	-3.168920	5.631480	4.418108
H	-1.632738	5.161627	5.138714
K	2.949516	-4.134963	-1.756944
C	-4.746866	2.561847	-4.281703
H	-5.683238	2.083344	-4.586173
H	-4.982411	3.573350	-3.941125
H	-4.116932	2.640232	-5.173566

Ia-Li

Electronic Energy BS1 = -2859.84054416 Hartree

Electronic Energy BS2 = -4303.96946100 Hartree

Zero-point Energy Correction = 0.912914 Hartree

Thermal Correction to Enthalpy = 0.967615 Hartree

Thermal Correction to Free Energy = 0.821760 Hartree

Chemical symbol X, Y, Z

H	-2.790737	0.884785	1.570938
C	-3.151005	-0.695585	0.097774
H	-4.001342	-0.802729	-0.585822
C	-1.162041	0.166485	-0.851044
C	-1.230892	2.472352	0.005670

C	-1.597684	3.529570	-0.824696	C	-5.725872	2.558226	-1.477046
C	-0.403380	2.717034	1.107485	H	-4.101186	1.230983	-1.950237
C	-1.171996	4.823240	-0.550833	C	-6.396018	3.250413	-0.468831
H	-2.240266	3.319138	-1.672057	H	-6.478973	3.683130	1.636689
C	0.016566	4.016763	1.381534	H	-6.056092	2.642812	-2.507718
C	-0.374252	5.068485	0.562403	H	-7.250407	3.873720	-0.713033
H	-1.467719	5.637829	-1.203523	N	-1.792024	1.188369	-0.245189
H	0.669222	4.186739	2.230433	N	-1.908555	-0.928587	-0.675212
H	-0.039789	6.076327	0.785014	C	-1.653677	-2.176082	-1.326203
S	0.134717	1.435289	2.250237	C	-2.242457	-2.417818	-2.574726
O	1.495051	1.869729	2.679635	C	-0.827723	-3.124228	-0.711177
O	0.308095	0.182447	1.455030	C	-2.006932	-3.643670	-3.192912
O	-0.889140	1.348706	3.290127	C	-0.620268	-4.336775	-1.368028
Cu	0.593494	0.156511	-1.642162	C	-1.204123	-4.618197	-2.601156
C	-4.195907	1.646388	0.159644	H	-2.154132	-0.638989	2.711300
C	-4.864872	2.345409	1.160361	H	-2.489338	-2.324447	4.450751
C	-4.632883	1.760515	-1.162871	C	-2.767538	-1.508060	2.489381
C	-5.964238	3.142888	0.848588	C	-2.942903	-2.473438	3.476097
H	-4.519797	2.268751	2.187755	C	-3.345772	-1.682911	1.228848

C	-3.685103	-3.622554	3.216366	O	3.318773	-1.272428	1.110427
H	-3.817504	-4.372680	3.989885	O	4.264425	-2.287510	-0.700088
C	-4.089054	-2.836756	0.976596	C	4.027176	-2.414273	1.651538
C	-4.256836	-3.805260	1.960993	C	4.300830	-3.270161	0.355964
H	-4.532224	-2.981693	-0.005494	C	3.205303	-4.289067	0.045522
H	-4.835743	-4.698300	1.747668	H	3.405595	-4.729040	-0.934325
C	2.453102	0.084464	-2.237816	H	3.178671	-5.090783	0.788980
C	3.283989	0.127449	-1.017304	H	2.225064	-3.812821	0.002528
C	-3.078756	-1.364845	-3.255440	C	5.662926	-3.951618	0.335666
H	-3.307404	-1.656931	-4.282694	H	5.775983	-4.509275	-0.597035
H	-4.032177	-1.202452	-2.739816	H	6.477370	-3.228491	0.397037
H	-2.551310	-0.405920	-3.283432	H	5.751911	-4.656332	1.168530
C	-0.136818	-2.837938	0.592032	C	3.146362	-3.086337	2.692246
H	0.198422	-3.769604	1.055824	H	2.168228	-3.340345	2.282488
H	0.730469	-2.186752	0.435972	H	3.623518	-3.998563	3.063385
H	-0.779541	-2.313041	1.295615	H	2.994617	-2.412608	3.539902
H	-2.452780	-3.837831	-4.165659	C	5.297037	-1.860583	2.296299
H	0.019509	-5.081653	-0.899816	H	5.920066	-1.342546	1.561628
B	3.615980	-1.181492	-0.246970	H	5.018249	-1.142776	3.072637

H	5.889033	-2.653956	2.759809
C	-3.001357	0.787430	0.501620
C	3.846129	1.232868	-0.454318
H	4.430009	1.118942	0.465657
C	3.849048	2.610824	-1.060911
C	2.520800	3.353672	-0.847438
C	5.011739	3.458579	-0.526203
H	3.987698	2.498575	-2.148307
C	2.542188	4.734219	-1.505859
H	2.355684	3.463261	0.232446
H	1.687834	2.755247	-1.235445
C	5.041084	4.853276	-1.156530
H	4.902583	3.555180	0.564580
H	5.961531	2.940406	-0.704006
C	3.704122	5.578457	-0.976190
H	1.589671	5.246307	-1.338368
H	2.647957	4.611729	-2.592984
H	5.857243	5.445423	-0.727504
H	5.253200	4.755689	-2.230206
H	3.730816	6.553352	-1.476197
H	3.542756	5.774543	0.093313
C	-0.988748	-5.951413	-3.270536
H	-1.751763	-6.672280	-2.956039
H	-1.046116	-5.864698	-4.358877
H	-0.012864	-6.372054	-3.013586
H	2.637053	0.928839	-2.914321
H	2.596087	-0.854076	-2.785281
Li	2.275702	0.417109	1.399730

Ib-Li

Imaginary Freq = -8.2517 cm^{-1}

Electronic Energy BS1 = -2859.84505997 Hartree

Electronic Energy BS2 = -4303.97406121 Hartree

Zero-point Energy Correction = 0.913105 Hartree

Thermal Correction to Enthalpy = 0.966688 Hartree

Thermal Correction to Free Energy = 0.824998 Hartree

Chemical symbol X, Y, Z

H	-3.293968	0.541535	0.876201

C	-2.222961	-1.314011	1.323445	C	-5.671388	-0.379804	0.591335
H	-2.634468	-2.325047	1.427804	C	-4.647473	-2.299323	-0.438089
C	-1.110603	-0.766569	-0.687869	C	-6.930415	-0.933512	0.368258
C	-2.832080	0.506483	-1.862670	H	-5.579346	0.591020	1.070494
C	-3.476323	-0.116089	-2.931115	C	-5.901587	-2.851858	-0.665202
C	-2.681869	1.896620	-1.864825	H	-3.753684	-2.824158	-0.766505
C	-3.972379	0.630011	-3.991794	C	-7.047492	-2.169186	-0.258803
H	-3.583972	-1.194591	-2.907302	H	-7.819012	-0.394518	0.680858
C	-3.180508	2.641819	-2.933545	H	-5.987812	-3.814035	-1.160443
C	-3.826520	2.013933	-3.990366	H	-8.028042	-2.599776	-0.435358
H	-4.470329	0.131051	-4.816462	N	-2.370326	-0.314254	-0.795588
H	-3.037480	3.716520	-2.933937	N	-1.000284	-1.386313	0.491364
H	-4.208459	2.606066	-4.815262	C	0.095397	-2.231947	0.852144
S	-1.875965	2.839064	-0.558589	C	0.044453	-3.579510	0.460952
O	-1.088391	3.854912	-1.315866	C	1.173138	-1.727276	1.586742
O	-0.889189	1.927145	0.101423	C	1.082808	-4.423604	0.842611
O	-2.921961	3.332221	0.327816	C	2.180574	-2.616963	1.962510
Cu	0.428914	-0.130654	-1.651102	C	2.154449	-3.962084	1.607302
C	-4.523039	-1.060296	0.195734	H	-1.883197	1.312726	2.158498

H	-1.307695	2.092954	4.411380	H	0.624283	0.353054	1.374425
C	-1.774029	0.587868	2.958627	H	1.058634	-5.465394	0.531610
C	-1.423252	1.029028	4.229954	H	3.023052	-2.232304	2.533078
C	-1.926218	-0.778009	2.707985	B	3.092194	2.090038	-0.218200
C	-1.207265	0.115278	5.258932	O	3.841917	2.298417	0.907355
H	-0.928572	0.463825	6.248609	O	2.235590	3.168513	-0.450775
C	-1.712805	-1.687678	3.743939	C	3.306937	3.443589	1.605388
C	-1.350485	-1.247196	5.013178	C	2.582682	4.238367	0.465306
H	-1.817070	-2.752324	3.550634	C	3.508060	5.180383	-0.301914
H	-1.183069	-1.967134	5.807996	H	2.988943	5.540636	-1.193616
C	2.046674	0.789940	-2.235072	H	3.785263	6.044276	0.307997
C	3.063762	0.839951	-1.129403	H	4.420251	4.667844	-0.619643
C	-1.095369	-4.104367	-0.373902	C	1.310137	4.957612	0.884272
H	-0.892023	-5.124400	-0.707252	H	0.856096	5.451446	0.021635
H	-2.034282	-4.124353	0.191300	H	0.571105	4.266838	1.293785
H	-1.257287	-3.482165	-1.259697	H	1.537547	5.718074	1.637758
C	1.293991	-0.277297	1.962788	C	4.456406	4.191377	2.264905
H	1.046201	-0.128910	3.019489	H	5.240287	4.435536	1.546268
H	2.320089	0.061415	1.797212	H	4.097491	5.117241	2.725343

H	4.895550	3.568598	3.048285
C	2.336954	2.921937	2.666695
H	1.475614	2.423174	2.214954
H	2.858130	2.196297	3.295974
H	1.972596	3.734622	3.301289
C	-3.161768	-0.454529	0.441129
H	2.380679	0.165951	-3.072725
H	1.910779	1.789363	-2.678586
C	3.906228	-0.163097	-0.799098
H	4.576303	-0.014132	0.048724
C	3.976214	-1.506109	-1.471818
C	4.685046	-1.474992	-2.840553
C	4.663836	-2.547918	-0.579915
H	2.938698	-1.841590	-1.648211
C	4.659373	-2.856069	-3.502925
H	5.725903	-1.156956	-2.688341
H	4.223637	-0.729412	-3.494668
C	4.637938	-3.939120	-1.213178
H	5.706771	-2.237501	-0.417013

H	4.180015	-2.565959	0.399504
C	5.284548	-3.926322	-2.602101
H	5.178846	-2.824146	-4.467287
H	3.615848	-3.127732	-3.715625
H	5.146776	-4.662483	-0.565117
H	3.592730	-4.269768	-1.294849
H	5.203868	-4.913437	-3.071239
H	6.357499	-3.716076	-2.491840
C	3.260156	-4.894191	2.030218
H	3.060498	-5.313267	3.022664
H	3.364805	-5.727992	1.330994
H	4.219979	-4.372053	2.078663
Li	0.542629	2.636002	-1.117762

II-*Z*,*R*-Li

Electronic Energy BS1 = -4127.84207477 Hartree

Electronic Energy BS2 = -5572.15579004 Hartree

Zero-point Energy Correction = 1.060519 Hartree

Thermal Correction to Enthalpy = 1.126051 Hartree

Thermal Correction to Free Energy = 0.959201 Hartree

Chemical symbol X, Y, Z

H	-4.130343	0.319746	-0.147757
C	-2.731236	0.070627	-1.825972
H	-2.528338	0.818726	-2.601004
C	-1.075990	-0.118670	-0.149981
C	-2.149962	0.951256	1.746967
C	-1.529263	2.133629	2.140357
C	-2.789250	0.161070	2.712753
C	-1.553139	2.535339	3.471376
H	-1.024472	2.726218	1.387043
C	-2.825062	0.570930	4.043588
C	-2.209933	1.760494	4.420239
H	-1.053308	3.452383	3.762973
H	-3.327374	-0.054798	4.772315
H	-2.236613	2.073095	5.458795
S	-3.641904	-1.359583	2.293596
O	-3.732909	-2.122747	3.580127
O	-2.725479	-2.164000	1.416414
O	-4.909369	-0.994977	1.671467
Cu	0.637916	-0.195262	0.814515
C	-3.490885	2.269915	-0.730630
C	-4.767591	2.784213	-0.521851
C	-2.452571	3.137711	-1.083710
C	-5.011784	4.148142	-0.671260
H	-5.572874	2.114305	-0.232930
C	-2.692567	4.498909	-1.223392
H	-1.448143	2.746368	-1.231685
C	-3.975274	5.006752	-1.019763
H	-6.011141	4.538181	-0.506435
H	-1.877966	5.167001	-1.485232
H	-4.161491	6.070486	-1.128625
N	-2.107302	0.560726	0.381048
N	-1.424094	-0.483330	-1.386452
C	1.638803	-1.198423	2.317079
C	0.411834	-1.809554	2.063978
C	-0.541142	-1.168449	-2.281176
C	0.364194	-0.409582	-3.035679

C	-0.592669	-2.563579	-2.394225	H	-0.545090	1.509544	-3.425819
C	1.264838	-1.074461	-3.863458	H	0.392881	1.424695	-1.922019
C	0.323277	-3.184966	-3.243519	C	-1.606967	-3.408730	-1.669531
C	1.264593	-2.464293	-3.973082	H	-2.398883	-3.717574	-2.360620
H	-4.622359	-1.586346	-0.595425	H	-1.136851	-4.316616	-1.277315
H	-5.955455	-3.341095	-1.660662	H	-2.093025	-2.871604	-0.853542
C	-4.521466	-1.747638	-1.663682	H	1.987163	-0.490538	-4.428164
C	-5.269743	-2.755315	-2.264602	H	0.300392	-4.269393	-3.327266
C	-3.636829	-0.982592	-2.426068	B	3.828438	0.484402	-0.553811
C	-5.135507	-3.018931	-3.625149	O	4.132342	-0.421924	0.445973
H	-5.717997	-3.809470	-4.088059	O	4.616068	0.289128	-1.668398
C	-3.516708	-1.243995	-3.792589	C	5.335049	-1.109587	0.058959
C	-4.254174	-2.260077	-4.390753	C	5.305779	-0.959669	-1.498465
H	-2.824364	-0.655919	-4.389695	C	4.458442	-2.033472	-2.185502
H	-4.143644	-2.455146	-5.452708	H	4.314977	-1.747829	-3.230699
C	1.728347	1.482904	0.659338	H	4.945173	-3.013110	-2.155233
C	2.740352	1.585141	-0.433979	H	3.468821	-2.112996	-1.725680
C	0.354811	1.090158	-2.960169	C	6.674621	-0.866998	-2.157615
H	1.225282	1.506524	-3.467781	H	6.555747	-0.763111	-3.239340

H	7.234772	-0.003684	-1.794640	C	0.921472	0.090788	4.370994
H	7.257716	-1.773176	-1.963510	C	3.505928	1.072675	4.660289
C	5.274739	-2.540847	0.570691	H	3.956188	-0.054712	2.878327
H	4.365751	-3.044254	0.239487	C	1.225697	0.955905	5.414713
H	6.141594	-3.111792	0.222672	H	-0.097296	-0.270181	4.269653
H	5.283336	-2.543161	1.664625	C	2.519280	1.448905	5.567930
C	6.504894	-0.366452	0.708084	H	4.515822	1.457920	4.763205
H	6.564680	0.663950	0.346893	H	0.445368	1.245181	6.112492
H	6.351392	-0.339078	1.790376	H	2.755029	2.122444	6.385920
H	7.458105	-0.864385	0.508696	H	1.027980	2.326118	0.626239
H	2.523837	-1.579714	1.813635	H	2.235072	1.531019	1.630103
C	0.187487	-3.033261	1.281506	C	2.792611	2.580900	-1.344983
H	-0.710289	-3.002708	0.676533	H	3.566923	2.542824	-2.112493
H	-0.457768	-1.549018	2.662590	C	1.866149	3.768992	-1.408548
C	-3.230084	0.791349	-0.547261	C	2.160515	4.809536	-0.311860
Cl	1.530610	-3.585467	0.280388	C	1.892928	4.449909	-2.783338
Cl	-0.269976	-4.476747	2.481685	H	0.834077	3.421957	-1.235881
C	1.906133	-0.297277	3.455345	C	1.150135	5.958577	-0.360734
C	3.202191	0.210696	3.613219	H	3.176413	5.201528	-0.458751

H	2.148133	4.323174	0.668388
C	0.900361	5.611568	-2.860789
H	2.910531	4.821778	-2.971713
H	1.677770	3.719723	-3.571715
C	1.131941	6.628719	-1.738518
H	1.373296	6.698812	0.415792
H	0.149425	5.560635	-0.135717
H	0.965173	6.104979	-3.837014
H	-0.118790	5.207069	-2.785307
H	0.367180	7.413352	-1.773027
H	2.098287	7.124593	-1.903647
C	2.262937	-3.178742	-4.847392
H	2.821106	-2.476994	-5.472516
H	2.986523	-3.730990	-4.238035
H	1.769114	-3.901283	-5.504322
Li	-2.449315	-3.432864	2.891552

TS$_{OA}$-*Z,R*-Li

Imaginary Freq = -196.9283 cm^{-1}

Electronic Energy BS1 = -4127.84080667 Hartree

Electronic Energy BS2 = -5572.15343302 Hartree

Zero-point Energy Correction = 1.059835 Hartree

Thermal Correction to Enthalpy = 1.125139 Hartree

Thermal Correction to Free Energy = 0.957859 Hartree

Chemical symbol X, Y, Z

H	-4.067186	0.022080	-0.085648
C	-2.710946	-0.300678	-1.780740
H	-2.559535	0.363066	-2.640510
C	-1.016784	-0.167334	-0.143369
C	-2.118987	1.088595	1.622980
C	-1.584937	2.355552	1.840509
C	-2.686807	0.390311	2.698905
C	-1.611414	2.926953	3.107876
H	-1.148819	2.881596	0.999599
C	-2.724523	0.969690	3.965131
C	-2.187919	2.237295	4.168010
H	-1.179471	3.909577	3.262156
H	-3.168338	0.412805	4.782414

H	-2.216647	2.680007	5.158068	C	1.789961	-0.754654	2.420184
S	-3.461228	-1.218750	2.506741	C	0.596528	-1.485578	2.311369
O	-3.478464	-1.810821	3.879485	C	-0.470720	-1.491406	-2.114442
O	-2.527672	-2.071557	1.701182	C	0.369220	-0.813768	-3.008835
O	-4.762944	-0.995051	1.885383	C	-0.450980	-2.888569	-2.014864
Cu	0.698935	-0.066792	0.821276	C	1.281012	-1.552085	-3.758349
C	-3.666444	1.937856	-0.922285	C	0.475257	-3.586131	-2.791031
C	-4.969226	2.347324	-0.650443	C	1.355737	-2.940286	-3.654155
C	-2.758314	2.855167	-1.460233	H	-4.488227	-1.905230	-0.328564
C	-5.368827	3.654572	-0.920682	H	-5.734180	-3.840663	-1.156722
H	-5.670014	1.640811	-0.214194	C	-4.392153	-2.183268	-1.373106
C	-3.152571	4.162267	-1.719110	C	-5.090117	-3.293752	-1.837891
H	-1.733045	2.548616	-1.659717	C	-3.560902	-1.466762	-2.236422
C	-4.461155	4.563495	-1.452379	C	-4.957117	-3.709007	-3.160089
H	-6.387089	3.962500	-0.705628	H	-5.499814	-4.579250	-3.515914
H	-2.438739	4.873420	-2.123040	C	-3.441472	-1.882201	-3.564262
H	-4.767229	5.584930	-1.654943	C	-4.127731	-3.000407	-4.025261
N	-2.068493	0.530939	0.315271	H	-2.790075	-1.332564	-4.239100
N	-1.367671	-0.723906	-1.303847	H	-4.018142	-3.314164	-5.058443

C	1.692972	1.633995	0.481502	H	5.074873	-2.953811	-1.942548
C	2.629903	1.662068	-0.677521	H	3.558921	-2.099872	-1.561791
C	0.273325	0.675494	-3.176330	C	6.665195	-0.726628	-2.258324
H	1.148387	1.059048	-3.701977	H	6.506415	-0.758517	-3.339408
H	-0.618578	0.954900	-3.750495	H	7.181650	0.204522	-2.019630
H	0.228283	1.176992	-2.208575	H	7.309180	-1.568071	-1.982827
C	-1.403123	-3.663554	-1.143104	C	5.454325	-2.147965	0.696213
H	-2.183177	-4.120880	-1.761346	H	4.566741	-2.736297	0.460767
H	-0.876750	-4.470951	-0.623622	H	6.341704	-2.707022	0.382597
H	-1.908815	-3.033497	-0.410271	H	5.498518	-2.022355	1.781948
H	1.951500	-1.026804	-4.433880	C	6.554909	0.093695	0.536382
H	0.507923	-4.670339	-2.708982	H	6.541541	1.076654	0.057418
B	3.792884	0.633534	-0.714580	H	6.433770	0.238369	1.613391
O	4.182722	-0.125593	0.372253	H	7.529700	-0.369635	0.359739
O	4.554418	0.358303	-1.828117	H	2.683526	-1.153640	1.946244
C	5.413226	-0.785365	0.021414	C	0.418136	-2.731053	1.632789
C	5.326315	-0.820970	-1.541029	H	-0.530259	-2.903723	1.146899
C	4.526149	-2.015250	-2.066353	H	-0.281784	-1.184643	2.875870
H	4.330536	-1.863762	-3.130973	C	-3.238406	0.528114	-0.584196

Cl	1.740588	-3.400507	0.723876	C	1.414681	4.115944	-3.311178
Cl	-0.042182	-4.212966	3.157320	H	0.524571	3.193211	-1.597080
C	2.034996	0.285903	3.434803	C	0.587846	5.826036	-1.057839
C	3.315408	0.848632	3.514880	H	2.684370	5.292016	-1.170159
C	1.040788	0.759464	4.299001	H	1.811063	4.461470	0.112471
C	3.596727	1.847180	4.440085	C	0.295072	5.143464	-3.483746
H	4.075891	0.511485	2.816835	H	2.377831	4.568364	-3.588116
C	1.322589	1.759938	5.220166	H	1.258040	3.274497	-3.994890
H	0.033031	0.359417	4.251201	C	0.442510	6.311982	-2.503691
C	2.601710	2.306560	5.298630	H	0.749604	6.677987	-0.388193
H	4.594729	2.272030	4.486116	H	-0.351344	5.349560	-0.738906
H	0.536621	2.113846	5.880622	H	0.273429	5.515109	-4.514169
H	2.819453	3.086489	6.021501	H	-0.667388	4.640593	-3.315630
H	0.924597	2.409385	0.402020	H	-0.411117	6.993855	-2.594132
H	2.241569	1.808148	1.411520	H	1.336103	6.890770	-2.774330
C	2.547338	2.534472	-1.704618	C	2.372580	-3.727561	-4.439789
H	3.284786	2.462450	-2.505247	H	2.829306	-3.119590	-5.225188
C	1.507855	3.615090	-1.863568	H	3.175710	-4.083323	-3.784947
C	1.727738	4.812929	-0.920481	H	1.918891	-4.605960	-4.908478

Li	-2.145239	-3.177995	3.336119

III-*Z*,*R*-Li

Electronic Energy BS1 = -4127.86109984 Hartree

Electronic Energy BS2 = -5572.17990253 Hartree

Zero-point Energy Correction = 1.060617 Hartree

Thermal Correction to Enthalpy = 1.127233 Hartree

Thermal Correction to Free Energy = 0.955488 Hartree

Chemical symbol X, Y, Z

H	3.769897	0.532687	-0.040200
C	2.379990	1.448143	-1.434080
H	2.222149	1.296509	-2.508769
C	0.832708	0.291861	-0.091902
C	2.151648	-1.624298	0.651758
C	1.804124	-2.870409	0.137753
C	2.718505	-1.546975	1.931476
C	1.968446	-4.024458	0.894562
H	1.409981	-2.919549	-0.870564
C	2.893525	-2.705540	2.684771
C	2.509567	-3.940523	2.172572
H	1.679093	-4.984379	0.479919
H	3.328350	-2.619246	3.674619
H	2.644607	-4.836519	2.769269
S	3.374247	-0.016324	2.605073
O	3.405154	-0.197522	4.079549
O	2.375542	1.050195	2.297161
O	4.674094	0.170890	1.957235
Cu	-0.746915	-0.280366	0.943093
C	3.826732	-0.675557	-1.770846
C	5.096851	-1.115902	-1.398044
C	3.289083	-1.090764	-2.991023
C	5.831016	-1.945271	-2.241533
H	5.502075	-0.806666	-0.438119
C	4.029295	-1.906778	-3.840326
H	2.289735	-0.775415	-3.285010
C	5.301614	-2.335280	-3.467024
H	6.818745	-2.280273	-1.941680
H	3.615452	-2.207426	-4.797655

H	5.876428	-2.971566	-4.132513	C	3.488844	5.010179	-2.092031
N	1.938684	-0.469731	-0.160802	H	2.344707	3.516014	-3.129953
N	1.047896	1.408801	-0.780968	H	3.354815	5.757129	-2.867890
C	-1.977394	-0.753824	2.437256	C	-1.627947	-1.816980	0.016666
C	-1.165432	0.310706	2.988572	C	-2.315118	-1.328790	-1.204025
C	0.047657	2.385008	-1.091934	C	-0.419295	1.105306	-3.236490
C	-0.694600	2.227447	-2.271687	H	-1.326802	0.831939	-3.778904
C	-0.128658	3.496478	-0.258322	H	0.333405	1.406287	-3.975973
C	-1.669926	3.173679	-2.573275	H	-0.055231	0.211366	-2.727068
C	-1.113201	4.421231	-0.608980	C	0.710836	3.738525	0.966969
C	-1.900689	4.272558	-1.746979	H	1.514188	4.443243	0.729309
H	3.921644	2.362380	0.700284	H	0.111717	4.184417	1.765675
H	4.959950	4.569176	0.934419	H	1.179189	2.829181	1.346198
C	3.815001	3.083679	-0.102995	H	-2.261013	3.047700	-3.477361
C	4.390715	4.342072	0.038943	H	-1.271254	5.278664	0.040826
C	3.079704	2.776331	-1.249849	B	-3.648165	-0.541581	-1.047834
C	4.226988	5.308320	-0.950284	O	-4.306552	-0.409428	0.151747
H	4.673260	6.290635	-0.831229	O	-4.321539	0.038403	-2.091210
C	2.924595	3.747634	-2.239860	C	-5.629836	0.089587	-0.143624

C	-5.416573	0.792051	-1.530138	H	-0.610870	2.364224	3.118652
C	-4.943760	2.240534	-1.406361	H	-0.342360	0.084013	3.661999
H	-4.646936	2.600319	-2.394718	C	3.073639	0.220047	-0.818640
H	-5.736819	2.889553	-1.024106	Cl	-2.576844	2.269011	1.737875
H	-4.070598	2.320015	-0.753129	Cl	0.621341	2.705062	5.166258
C	-6.607328	0.707623	-2.474341	C	-1.881047	-2.112311	3.010392
H	-6.369434	1.216067	-3.412160	C	-3.029281	-2.910491	3.065481
H	-6.864243	-0.327301	-2.705430	C	-0.667573	-2.646443	3.462995
H	-7.481188	1.197536	-2.033348	C	-2.972482	-4.202841	3.577732
C	-6.081521	1.010139	0.979181	H	-3.970320	-2.506936	2.701283
H	-5.368926	1.816632	1.152447	C	-0.611937	-3.936277	3.972418
H	-7.057232	1.446870	0.743933	H	0.246598	-2.062913	3.398510
H	-6.179069	0.440663	1.907337	C	-1.763931	-4.718666	4.035338
C	-6.548106	-1.131441	-0.225125	H	-3.873770	-4.806189	3.619361
H	-6.241351	-1.804302	-1.030950	H	0.337744	-4.332473	4.317321
H	-6.489182	-1.681369	0.717641	H	-1.717581	-5.725902	4.436798
H	-7.589000	-0.840190	-0.390007	H	-0.734854	-2.419621	-0.150782
H	-2.967969	-0.470640	2.083532	H	-2.309173	-2.349577	0.672789
C	-1.274023	1.625429	2.671840	C	-1.932361	-1.661556	-2.454139

H	-2.553268	-1.306779	-3.277594
C	-0.775386	-2.543353	-2.843231
C	-1.075150	-4.021805	-2.510556
C	-0.428172	-2.402982	-4.332438
H	0.109522	-2.236558	-2.263868
C	0.066867	-4.951145	-2.927128
H	-1.992923	-4.313704	-3.038946
H	-1.286037	-4.127703	-1.441723
C	0.712131	-3.336155	-4.743198
H	-1.324330	-2.637120	-4.924749
H	-0.167347	-1.362881	-4.560040
C	0.394662	-4.795708	-4.413457
H	-0.195768	-5.989336	-2.697344
H	0.964836	-4.715716	-2.337622
H	0.927350	-3.220232	-5.811122
H	1.620098	-3.047419	-4.200215
H	1.237460	-5.438811	-4.687832
H	-0.466355	-5.126860	-5.009889
C	-2.995343	5.259626	-2.059351
H	-3.257060	5.245126	-3.120784
H	-3.901961	5.020711	-1.491687
H	-2.701199	6.278366	-1.792766
Li	2.171242	1.443604	4.311794

II-*Z,S*-Li

Electronic Energy BS1 = -4127.84739327 Hartree

Electronic Energy BS2 = -5572.15933450 Hartree

Zero-point Energy Correction = 1.060632 Hartree

Thermal Correction to Enthalpy = 1.126413 Hartree

Thermal Correction to Free Energy = 0.958095 Hartree

Chemical symbol X, Y, Z			
C	-2.779708	-1.532287	0.092297
H	-3.357030	-1.697175	-0.816537
C	-3.247158	-0.238007	0.800936
H	-3.307999	-0.414356	1.882691
C	-1.035072	0.066624	0.029844
C	-0.510647	-2.104489	-0.912166
C	0.673087	-2.429700	-0.256857

C	-0.756485	-2.638028	-2.185634	H	-1.511387	-2.078692	2.465133
C	1.615529	-3.258990	-0.851367	C	-2.998930	-5.133360	2.431326
H	0.868350	-1.998168	0.717616	H	-4.302394	-5.857852	0.880195
C	0.170472	-3.505753	-2.761620	H	-1.657025	-4.153331	3.799765
C	1.354855	-3.812354	-2.101223	H	-3.053595	-6.045653	3.017101
H	2.548972	-3.453148	-0.332131	N	-1.388325	-1.192833	-0.270713
H	-0.028155	-3.898475	-3.752069	N	-2.106277	0.673043	0.554863
H	2.075064	-4.473906	-2.571445	C	1.766361	2.233638	-1.378400
S	-2.179455	-2.206916	-3.190643	C	0.480882	2.052525	-1.874762
O	-1.709626	-2.353679	-4.605007	C	0.049454	1.086764	-2.901983
O	-2.396367	-0.725725	-2.995626	H	-0.847329	0.539250	-2.624783
O	-3.313421	-3.031632	-2.800211	H	-0.288929	2.789565	-1.666219
Cu	0.723854	0.956800	-0.117423	H	2.568890	1.593439	-1.739287
C	-2.853456	-2.786111	0.929457	C	-2.070744	2.034891	0.987757
C	-3.623375	-3.858228	0.483221	C	-1.641002	2.323224	2.291978
C	-2.140502	-2.900219	2.125625	C	-2.423154	3.054708	0.095161
C	-3.700447	-5.027654	1.235694	C	-1.533878	3.656099	2.676557
H	-4.141045	-3.780679	-0.468106	C	-2.307767	4.377226	0.529335
C	-2.214175	-4.069373	2.873207	C	-1.858177	4.697652	1.807120

H	-4.422965	-0.283994	-1.722061	H	-2.540205	3.519900	-2.004257
H	-6.556005	0.737873	-2.381876	H	-2.644427	1.782290	-1.648998
C	-5.017239	0.234225	-0.975418	H	-1.174795	3.887307	3.676503
C	-6.215399	0.830531	-1.355442	H	-2.566077	5.177025	-0.161299
C	-4.568885	0.338819	0.343326	B	3.912798	-0.720947	0.447868
C	-6.970203	1.548460	-0.431142	O	4.388367	0.201673	-0.461905
H	-7.903078	2.014273	-0.733048	O	4.577756	-1.927157	0.341164
C	-5.333385	1.055792	1.264782	C	5.318138	-0.469886	-1.332950
C	-6.524648	1.663700	0.882394	C	5.736810	-1.716789	-0.479199
H	-4.985983	1.145636	2.290987	C	6.908546	-1.432285	0.463272
H	-7.105643	2.220908	1.610320	H	7.009046	-2.266678	1.161752
C	2.027123	0.821690	1.405112	H	7.849093	-1.320380	-0.083772
C	2.789380	-0.450293	1.487439	H	6.731648	-0.523990	1.046200
C	-1.304348	1.219148	3.256667	C	6.005564	-2.982094	-1.282431
H	-0.660347	1.585599	4.059182	H	6.279392	-3.795039	-0.604793
H	-2.208946	0.803221	3.716360	H	5.122257	-3.291238	-1.844593
H	-0.779648	0.405105	2.753698	H	6.832327	-2.828556	-1.983359
C	-2.924647	2.777199	-1.298433	C	6.452074	0.489137	-1.668650
H	-4.017519	2.829113	-1.323463	H	6.910431	0.897409	-0.766346

H	7.223970	-0.015928	-2.258196	H	2.714266	1.657433	1.244100
H	6.064853	1.323104	-2.261025	C	2.588913	-1.431499	2.397366
C	4.559892	-0.850707	-2.603407	H	1.460798	1.032168	2.319772
H	3.727079	-1.522824	-2.375345	H	3.215577	-2.322838	2.350603
H	4.151375	0.054689	-3.058984	C	1.591012	-1.389842	3.522999
H	5.216409	-1.334887	-3.332034	C	1.175055	-2.794026	3.981360
Cl	1.260561	-0.114913	-3.399587	C	2.138679	-0.576310	4.712868
Cl	-0.551848	1.944628	-4.484991	H	0.684058	-0.878408	3.168433
C	2.206431	3.425466	-0.624671	C	0.199468	-2.746344	5.160642
C	3.567850	3.561658	-0.329512	H	2.074886	-3.351225	4.279612
C	1.318175	4.403177	-0.159058	H	0.731649	-3.342823	3.141004
C	4.031480	4.654595	0.394880	C	1.159410	-0.533745	5.887771
H	4.253257	2.779735	-0.643610	H	3.081417	-1.035934	5.041136
C	1.782829	5.491397	0.567517	H	2.384562	0.435280	4.374078
H	0.251520	4.309334	-0.336452	C	0.768170	-1.944238	6.333439
C	3.142152	5.626300	0.843962	H	-0.053160	-3.762786	5.485558
H	5.090895	4.742111	0.615674	H	-0.739625	-2.274571	4.835269
H	1.076804	6.236642	0.922072	H	1.594475	0.024868	6.724115
H	3.502954	6.478277	1.411714	H	0.252210	0.007912	5.584722

H	0.041699	-1.901422	7.152608
H	1.657114	-2.458550	6.723729
C	-1.729100	6.132029	2.251121
H	-2.562353	6.417240	2.902521
H	-0.804613	6.285998	2.815375
H	-1.726580	6.815233	1.397681
Li	-1.572022	-0.411697	-4.776054

TS$_{OA}$-*Z*,*S*-Li

Imaginary Freq = -200.5961 cm^{-1}

Electronic Energy BS1 = -4127.84529809 Hartree

Electronic Energy BS2 = -5572.15605075 Hartree

Zero-point Energy Correction = 1.059727 Hartree

Thermal Correction to Enthalpy = 1.125255 Hartree

Thermal Correction to Free Energy = 0.957025 Hartree

Chemical symbol X, Y, Z

C	-2.799789	-1.479847	0.021402
H	-3.371082	-1.564922	-0.902341
C	-3.213757	-0.205175	0.793864
H	-3.243223	-0.422113	1.869949
C	-1.006298	0.057291	0.010342
C	-0.554732	-2.089030	-1.018701
C	0.607924	-2.521868	-0.389481
C	-0.820663	-2.501864	-2.332410
C	1.513289	-3.343803	-1.048749
H	0.815211	-2.177329	0.616605
C	0.065758	-3.365208	-2.973767
C	1.231061	-3.781603	-2.338837
H	2.434042	-3.624790	-0.546927
H	-0.145224	-3.664422	-3.993920
H	1.920996	-4.436110	-2.861309
S	-2.217972	-1.917708	-3.300796
O	-1.763022	-1.993865	-4.721975
O	-2.356536	-0.449048	-2.992123
O	-3.390858	-2.710642	-2.956516
Cu	0.763227	0.928320	-0.134885
C	-2.943748	-2.772533	0.785454
C	-3.756541	-3.781261	0.272644

C	-2.251490	-2.985643	1.979711	C	-2.302618	3.065004	0.041927
C	-3.895522	-4.985579	0.958488	C	-1.504359	3.691155	2.648196
H	-4.259009	-3.626870	-0.677504	C	-2.181951	4.391064	0.461985
C	-2.385920	-4.189596	2.660384	C	-1.779063	4.723943	1.752830
H	-1.590373	-2.213242	2.369416	H	-4.411817	-0.041676	-1.707545
C	-3.213168	-5.190084	2.152533	H	-6.559990	1.016916	-2.257358
H	-4.530771	-5.766039	0.551979	C	-5.000424	0.412054	-0.915451
H	-1.843423	-4.350191	3.585825	C	-6.207662	1.027211	-1.230786
H	-3.316398	-6.130081	2.685523	C	-4.537252	0.411910	0.402544
N	-1.389241	-1.186697	-0.307534	C	-6.958105	1.656766	-0.240688
N	-2.056360	0.685576	0.548907	H	-7.898942	2.136261	-0.492186
C	1.921612	2.233031	-1.233032	C	-5.296182	1.042272	1.389189
C	0.664194	2.115173	-1.843855	C	-6.498428	1.666401	1.072717
C	0.271064	1.104269	-2.775889	H	-4.938361	1.047689	2.415797
H	-0.719734	0.678936	-2.677029	H	-7.076376	2.154055	1.851218
H	-0.110071	2.852015	-1.656693	C	1.981276	0.737390	1.436960
H	2.727407	1.581821	-1.565256	C	2.694161	-0.563867	1.504185
C	-2.002481	2.052591	0.963948	C	-1.346417	1.263702	3.279155
C	-1.619123	2.353858	2.278769	H	-0.649862	1.605864	4.048211

H	-2.269325	0.949056	3.780959	H	5.173994	-3.379087	-1.766804
H	-0.911578	0.384455	2.801321	H	6.896749	-2.944782	-1.771386
C	-2.757215	2.777622	-1.366025	C	6.558392	0.368996	-1.391114
H	-3.844356	2.880541	-1.435763	H	6.958682	0.744248	-0.447767
H	-2.312768	3.485083	-2.072699	H	7.360625	-0.137200	-1.937428
H	-2.511884	1.764325	-1.686983	H	6.231087	1.225434	-1.987412
H	-1.181713	3.931228	3.658454	C	4.712447	-0.904889	-2.494443
H	-2.400459	5.184271	-0.249672	H	3.858141	-1.572128	-2.346021
B	3.860591	-0.840590	0.512896	H	4.348784	0.018741	-2.951074
O	4.409771	0.094653	-0.338521	H	5.412736	-1.377639	-3.188801
O	4.497641	-2.060220	0.412600	Cl	1.435856	-0.113409	-3.225270
C	5.385297	-0.574372	-1.163809	Cl	-0.298651	1.991825	-4.685094
C	5.717560	-1.852639	-0.318336	C	2.351136	3.401395	-0.442904
C	6.823418	-1.618291	0.713298	C	3.700732	3.501371	-0.083683
H	6.858157	-2.473293	1.392908	C	1.461567	4.386984	0.003670
H	7.802456	-1.511377	0.237619	C	4.153169	4.568963	0.684166
H	6.623285	-0.722397	1.307875	H	4.384236	2.713804	-0.388035
C	6.019776	-3.101203	-1.135238	C	1.915154	5.449842	0.773042
H	6.231176	-3.936697	-0.462768	H	0.402600	4.317805	-0.223153

C	3.263293	5.549346	1.112921
H	5.203018	4.630710	0.953463
H	1.209692	6.202429	1.112375
H	3.615018	6.381827	1.714208
H	2.693840	1.560421	1.346447
C	2.424126	-1.558029	2.380449
H	1.349992	0.926829	2.311390
H	3.027976	-2.464921	2.332675
C	1.385204	-1.522999	3.468865
C	0.955778	-2.933677	3.895565
C	1.893493	-0.725594	4.687475
H	0.492387	-1.005844	3.086726
C	-0.054137	-2.906056	5.046075
H	1.847720	-3.492766	4.212943
H	0.539936	-3.471995	3.034729
C	0.878144	-0.702676	5.831934
H	2.826328	-1.188650	5.037996
H	2.148662	0.291742	4.373540
C	0.478265	-2.120017	6.246277
H	-0.310775	-3.928338	5.347902
H	-0.986152	-2.434572	4.701563
H	1.286788	-0.154378	6.688031
H	-0.021188	-0.159498	5.509564
H	-0.272040	-2.089972	7.044013
H	1.356555	-2.637812	6.655437
C	-1.654554	6.163847	2.179915
H	-2.562183	6.498643	2.694005
H	-0.817098	6.299425	2.870182
H	-1.500746	6.822358	1.320704
Li	-1.574568	-0.023118	-4.823670

III-*Z*,*S*-Li

Electronic Energy BS1 = -4127.86685581 Hartree

Electronic Energy BS2 = -5572.18466315 Hartree

Zero-point Energy Correction = 1.061342 Hartree

Thermal Correction to Enthalpy = 1.127476 Hartree

Thermal Correction to Free Energy = 0.958804 Hartree

Chemical symbol X, Y, Z

C	-2.690651	-1.501824	0.245665	Cu	0.731197	0.962264	-0.427533
H	-3.350179	-1.566517	-0.619489	C	-2.779531	-2.801660	1.005205
C	-3.002573	-0.220637	1.054513	C	-3.541456	-3.841710	0.474957
H	-2.898449	-0.424188	2.127929	C	-2.085339	-2.999018	2.200534
C	-0.930530	0.025998	-0.021956	C	-3.626177	-5.059925	1.144840
C	-0.586978	-2.134785	-1.067719	H	-4.046418	-3.696124	-0.475470
C	0.577583	-2.693272	-0.555059	C	-2.168896	-4.215258	2.867974
C	-0.973339	-2.428688	-2.381927	H	-1.466165	-2.201884	2.607297
C	1.385640	-3.504498	-1.344355	C	-2.944097	-5.247280	2.341968
H	0.856309	-2.459958	0.463876	H	-4.222414	-5.863589	0.724873
C	-0.178072	-3.263421	-3.161239	H	-1.629872	-4.360378	3.798540
C	1.004212	-3.791660	-2.650510	H	-3.009105	-6.196630	2.864294
H	2.309163	-3.895415	-0.929889	N	-1.313297	-1.232963	-0.234197
H	-0.482954	-3.461694	-4.182675	N	-1.885568	0.667428	0.653646
H	1.622959	-4.426250	-3.276702	C	2.144514	2.271766	-1.137626
S	-2.467043	-1.770384	-3.130000	C	0.933120	2.483294	-1.860060
O	-2.229665	-1.788137	-4.597081	C	0.310544	1.519189	-2.615962
O	-2.534811	-0.338278	-2.701977	H	-0.658340	1.684228	-3.085726
O	-3.575584	-2.594307	-2.650126	H	0.317188	3.353151	-1.643591

H	2.860758	1.555107	-1.535497	C	2.580160	-0.623036	1.232737
C	-1.787688	2.054423	0.999637	C	-1.149462	1.380179	3.364209
C	-1.379041	2.410951	2.292065	H	-0.348025	1.692022	4.038866
C	-2.079069	3.032123	0.032069	H	-2.054005	1.237822	3.966950
C	-1.205719	3.761550	2.590966	H	-0.879563	0.409429	2.945600
C	-1.885574	4.369895	0.379766	C	-2.620407	2.705086	-1.333276
C	-1.444890	4.755003	1.645275	H	-3.698966	2.889848	-1.349948
H	-4.371375	0.131955	-1.320596	H	-2.178469	3.336406	-2.109428
H	-6.575213	1.184419	-1.624816	H	-2.471061	1.665107	-1.618804
C	-4.914834	0.500071	-0.454749	H	-0.866950	4.039011	3.586233
C	-6.154801	1.105556	-0.627451	H	-2.094119	5.132403	-0.367588
C	-4.362698	0.393330	0.824201	B	3.690507	-0.959034	0.191957
C	-6.851510	1.610855	0.467974	O	4.232087	-0.071296	-0.707330
H	-7.819098	2.082174	0.326839	O	4.270309	-2.202489	0.109155
C	-5.065643	0.900071	1.917025	C	5.133706	-0.808127	-1.566906
C	-6.305080	1.508054	1.743378	C	5.454860	-2.075974	-0.700496
H	-4.639261	0.816640	2.913911	C	6.628691	-1.868611	0.258304
H	-6.842639	1.897196	2.602213	H	6.665076	-2.706849	0.958341
C	1.853758	0.676345	1.197014	H	7.581165	-1.821608	-0.276729

H	6.507653	-0.948883	0.837851	H	4.708485	2.545662	-0.489557
C	5.644408	-3.360753	-1.493893	C	2.482292	5.179595	1.258785
H	5.849881	-4.187487	-0.809026	H	0.832782	4.232052	0.280957
H	4.751787	-3.609406	-2.070604	C	3.860195	5.186955	1.473025
H	6.491201	-3.268739	-2.181098	H	5.733532	4.235614	1.008892
C	6.331571	0.077078	-1.879571	H	1.846586	5.897830	1.767521
H	6.802322	0.453129	-0.969468	H	4.303771	5.918321	2.141083
H	7.078126	-0.476189	-2.457684	H	2.545070	1.515073	1.251539
H	6.009154	0.933731	-2.478065	C	2.393289	-1.550717	2.195990
C	4.374335	-1.139769	-2.849211	H	1.107480	0.782684	1.986558
H	3.493150	-1.753697	-2.640150	H	3.018440	-2.443559	2.155590
H	4.038366	-0.212034	-3.317837	C	1.451507	-1.483091	3.366186
H	5.012889	-1.670143	-3.560856	C	1.140076	-2.884768	3.910039
Cl	1.149165	0.138510	-3.247738	C	2.028067	-0.585299	4.480724
Cl	-1.860836	2.388516	-5.008457	H	0.504517	-1.033554	3.035054
C	2.713137	3.303578	-0.254326	C	0.241704	-2.836062	5.147797
C	4.091981	3.302965	-0.014746	H	2.086940	-3.377389	4.173196
C	1.911711	4.246074	0.405093	H	0.677632	-3.492786	3.123620
C	4.661811	4.243748	0.837398	C	1.131104	-0.552564	5.720409

H	3.019032	-0.969869	4.758283
H	2.184772	0.425781	4.089086
C	0.850420	-1.961640	6.245875
H	0.064586	-3.850364	5.522686
H	-0.740312	-2.425684	4.871703
H	1.594137	0.062709	6.499372
H	0.175791	-0.073664	5.466262
H	0.182809	-1.919228	7.112904
H	1.789983	-2.414869	6.589922
C	-1.237883	6.211939	1.970863
H	-2.194901	6.741388	2.021637
H	-0.733698	6.339399	2.932298
H	-0.636136	6.704435	1.200316
Li	-2.294441	0.265059	-4.65752

13. References

1. J. Kuang, S. Ma. *J. Org. Chem.* **2009**, *74*, 1763.
2. M. L. Cooke, K. Xu, B. Breit, *Angew. Chem. Int. Ed.* **2012**, *51*, 10876.
3. R. Y. Liu, Y. Yang, S. L. Buchwald, *Angew. Chem. Int. Ed.* **2016**, *55*, 14077.
4. Z. Liu, B. Breit, *Org. Lett.* **2018**, *20*, 300.
5. C. Li, Z. Yang, L. Wang, Y. Guo, Z. Huang, S. Ma, *Angew. Chem. Int. Ed.* **2020**, *59*, 6278.
6. L. Lu, Siu, C. J., Y. Lai, S. Lin, *J. Am. Chem. Soc.* **2020**, *142*, 21272.
7. M. S. Newman, P. K. Sujeeth, *J. Org. Chem.* **1978**, *43*, 4367.
8. M. Giannerini, M. Fañanás-Mastral, B. L. Feringa, *J. Am. Chem. Soc.* **2012**, *134*, 4108.
9. A. Chaves-Pouso, A. M. Álvarez-Constantino, M. Fañanás-Mastral, *Angew. Chem. Int. Ed.* **2022**, *61*, e202282361.
10. H. Clavier, L. Coutable, L. Toupet, J.-C. Guillemin, M. Mauduit, *J. Organomet. Chem.* **2005**, *690*, 5237.
11. B. A. B. Prasad, S.R. Gilbertson, *Org. Lett.* **2009**, *11*, 3710.
12. T. J. Gong, S. H. Yu, K. Li, X. Lu, B. Xiao, Y. Fu, *Chem. Asian J.* **2017**, *12*, 2884.
13. M. K. Brown, T. L. May, C. A. Baxter, A. H. Hoveyda, *Angew. Chem. Int. Ed.* **2007**, *46*, 1097.
14. F. Gao, J. L. Carr, A. H. Hoveyda, *J. Am. Chem. Soc.* **2014**, *136*,2149.
15. M. J. Frisch, G. W. Trucks, H. B. Schlegel, G. E. Scuseria, M. A. Robb, J. R. Cheeseman, G. Scalmani, V. Barone, G. A. Petersson, H. Nakatsuji, X. Li, M. Caricato, A. V. Marenich, J. Bloino, B. G. Janesko, R. Gomperts, B. Mennucci, H. P. Hratchian, J. V. Ortiz, A. F. Izmaylov, J. L. Sonnenberg, D. Williams-Young, F. Ding, F. Lipparini, F. Egidi, J. Goings, B. Peng, A. Petrone, T. Henderson, D. Ranasinghe, V. G. Zakrzewski, J. Gao, N. Rega, G. Zheng, W. Liang, M. Hada, M. Ehara, K. Toyota, R. Fukuda, J. Hasegawa, M. Ishida, T. Nakajima, Y. Honda, O. Kitao, H. Nakai, T. Vreven, K. Throssell, J. J. A. Montgomery, J. E. Peralta, F. Ogliaro, M. J. Bearpark, J. J. Heyd, E. N. Brothers, K. N. Kudin, V. N. Staroverov, T. A. Keith, R. Kobayashi, J. Normand, K. Raghavachari, A. P. Rendell, J. C. Burant, S. S. Iyengar, J. Tomasi, M. Cossi, J. M. Millam, M. Klene, C. Adamo, R. Cammi, J. W. Ochterski, R. L. Martin, K. Morokuma, O. Farkas, J. B. Foresman, D. J. Fox, *Gaussian 16, Revision B.01*, Gaussian.
16. Price, A. J. A.; Bryenton, K. R.; Johnson, E. R. *J. Chem. Phys.* **2021**, *154*, 230902.
17. J.-D. Chai, M. Head-Gordon, *Phys. Chem. Chem. Phys.* **2008**, *10*, 6615.
18. a) W. J. Hehre, R. Ditchfield, J. A. Pople, *J. Chem. Phys.* **1972**, *56*, 2257. b) M. M. Francl, W. J. Pietro, W. J. Hehre, J. S. Binkley, M. S. Gordon, D. J. DeFrees, J. A. Pople, *J. Chem. Phys.* **1982**, *77*, 3654.
19. D. Andrae, U. Häußermann, M. Dolg, H. Stoll, H. Preuß, *Theor. Chim. Acta* **1990**, *77*, 123.
20. A. W. Ehlers, M. Böhme, S. Dapprich, A. Gobbi, A. Höllwarth, V. Jonas, K. F. Köhler, R. Stegmann, A. Veldkamp, G. Frenking, *Chem. Phys. Lett.* **1993**, *208*, 111.
21. A. V. Marenich, C. J. Cramer, D. G. Truhlar, *J. Phys. Chem. B,* **2009**, *113*, 6378
22. a) F. Weigend, R. Ahlrichs, *Phys. Chem. Chem. Phys.* **2005**, *7*, 3297; b) D. Rappoport, F. Furche, *J. Chem. Phys.* **2010**, *133*, 134105.
23. C. Y. Legault, *CYLview20*, **2020** (http://www.cylview.org)
24. E. R. Johnson, S. Keinan, P. Mori-Sánchez, J. Contreras-García, A. J. Cohen, W. Yang *J. Am. Chem. Soc.* **2010**, *132*, 6498.
25. T. Lu, F. Chen *J. Comput. Chem.* **2012**, *33*, 580.
26. W. Humphrey, A. Dalke, K. Schulten *J. Molec. Graphics* **1996**, *14*, 33.
